# Carbonylative N-Heterocyclization via Nitrogen-Directed C–C Bond Activation of Nonactivated Cyclopropanes

**DOI:** 10.1021/jacs.2c02921

**Published:** 2022-06-17

**Authors:** Adam D. J. Calow, David Dailler, John F. Bower

**Affiliations:** † School of Chemistry, University of Bristol, Bristol, BS8 1TS, United Kingdom; ‡ Department of Chemistry, University of Liverpool, Crown Street, Liverpool, L69 7ZD, United Kingdom

## Abstract

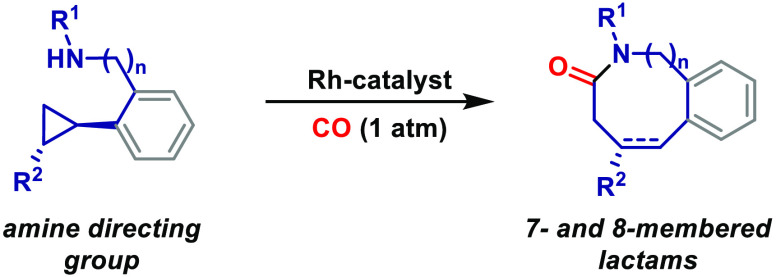

Under Rh-catalyzed conditions, secondary amines and anilines function as directing groups to facilitate regioselective C–C bond activation of nonactivated cyclopropanes. The resulting amino-stabilized rhodacycles undergo carbonylative C–N bond formation en route to challenging seven- and eight-membered lactams. The processes represent rare examples where C–C bond oxidative addition of nonactivated cyclopropanes is exploited in reaction design.

Processes enabled by the oxidative addition of C–C bonds to transition metals (termed here as “C–C bond activation”) are of emerging strategic importance.^1^ Predominant methodologies harness strained carbocycles to facilitate the C–C cleavage process, such that cyclopropane derivatives are commonly employed as initiating motifs (Scheme 1A).^2−5^ For example, C–C bond activations of alkylidene cyclopropanes,^2^ cyclopropenes,^3^ and vinyl cyclopropanes have found widespread use;^4,5^ in these cases, the fused, cyclic, or adjacent π-unsaturation predisposes the system to metal insertion. By contrast, the use of simple and more readily available nonactivated cyclopropanes is much rarer and is limited to just a handful of processes^6^ outside of simple reduction or isomerization reactions.^7^ Key issues include (a) the more challenging C–C bond activation process, (b) achieving regiocontrol, and (c) the instability of the incipient metallacycle, which is prone to deleterious β-hydride elimination (Scheme 1A, dashed box).

**Scheme 1:**
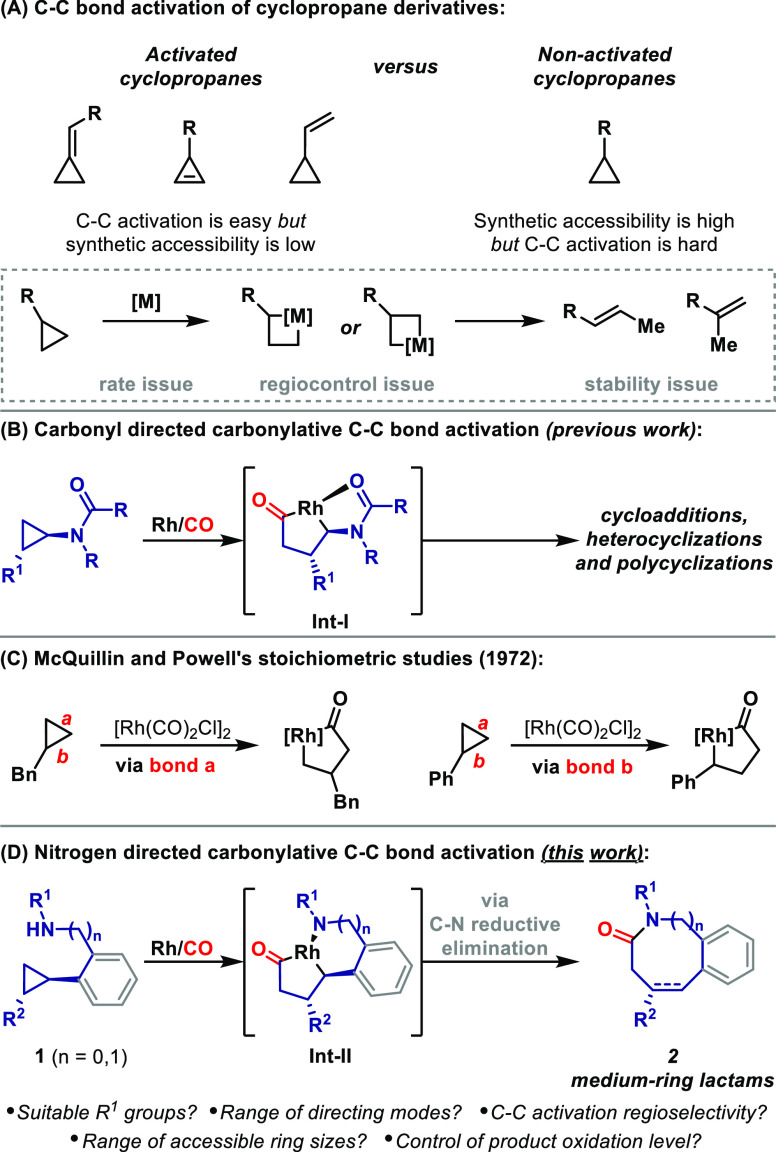
C–C Bond Activations of Cyclopropanes

We have previously reported a range of processes that are enabled by carbonyl-directed carbonylative C–C bond activation of aminocyclopropanes (Scheme 1B).^8−10^ Here, the directing group accelerates the rate of C–C bond activation and controls regioselectivity, whereas fast carbonylation of the rhodacyclobutane provides relatively stable rhodacyclopentanones (**Int-I**). Aminocyclopropanes are electronically privileged systems for these processes, and extension of our weak directing group strategy to completely nonactivated cyclopropanes has proven highly challenging.^9d^ Nevertheless, carbonylative C–C bond activations of nonactivated cyclopropanes are efficient in stoichiometric settings.^11^ For example, McQuillin and Powell reported distinct regiochemical outcomes for the carbonylative insertion of [Rh(CO)_2_Cl]_2_ into benzyl- and phenyl-substituted cyclopropanes (Scheme 1C).^11c^ To translate this reactivity into catalytic processes, we considered replacing the weak carbonyl-based directing group used in Scheme 1B with much stronger N-based variants (Scheme 1D). In this design, N-directed carbonylative C–C bond activation of **1** leads to more stable metallabicycles **Int-II**, which can then undergo C–N reductive elimination en route to medium ring lactams **2**. In this report, we outline our efforts in this area, which now allow diverse nonactivated cyclopropanes to be employed as initiating motifs for challenging carbonylative heterocyclizations. In broader terms, this work demonstrates how amines can be used to direct efficient C–C bond activation processes,^12,13^ thereby complementing recently reported amine-directed C–H functionalization reactions.^14^

Initial efforts to realize the processes depicted in Scheme 1D focused on accessing benzazepines by N-directed C–C bond activation of aryl-substituted cyclopropanes **1a**–**g** (Scheme 2A). Here, NH-metalation was expected to trigger N-directed carbonylative C–C bond activation and provide 5,5-metallabicycles **Int-IIa**. These should then undergo C–N reductive elimination to give alkyl-Rh(I) species **Int-IIIa**, from which (reversible) β-hydride elimination generates C2–C3 unsaturated products **2a**–**g**. Other mechanistic options leading to **Int-IIIa** are also potentially feasible.^15^*N*-Benzyl system **1a**, which was prepared via cross-coupling with cyclopropylboronic acid, was exposed to various Rh catalysts under an atmospheric pressure of CO. Ultimately, it was found that the combination of [Rh(cod)_2_]BARF (5 mol %) and dimethyl fumarate (100 mol %) in PhCN at 120 °C was optimal (see the SI); under these conditions, unsaturated system **2a** was isolated in 72% yield. The use of exogenous phosphine or arsine ligands^9d^ or neutral Rh complexes (e.g., [Rh(cod)Cl]_2_) resulted in substantially lower efficiencies. PhCN, a coordinating solvent that has been shown to stabilize Rh systems in other contexts,^9c^ was optimal, and less coordinating options, such as 1,2-dichlorobenzene, resulted in minimal conversion. The addition of dimethyl fumarate (100 mol %) was the most critical factor, leading to a cleaner reaction and an approximately 30% increase in yield. This additive may function as a π-bound ligand,^16^ but, perhaps more importantly, it also serves as the oxidant for the conversion of **1a** to **2a**; this was confirmed by ^1^H NMR and GCMS analysis of the crude reaction mixture, which revealed the stoichiometric formation of dimethyl succinate (see the SI). This presumably arises via a hydrometalation–protodemetalation sequence (Scheme 2A), with the latter step mediated by the proton released upon conversion of **1a** to **Int-IIa**.^15^

**Scheme 2:**
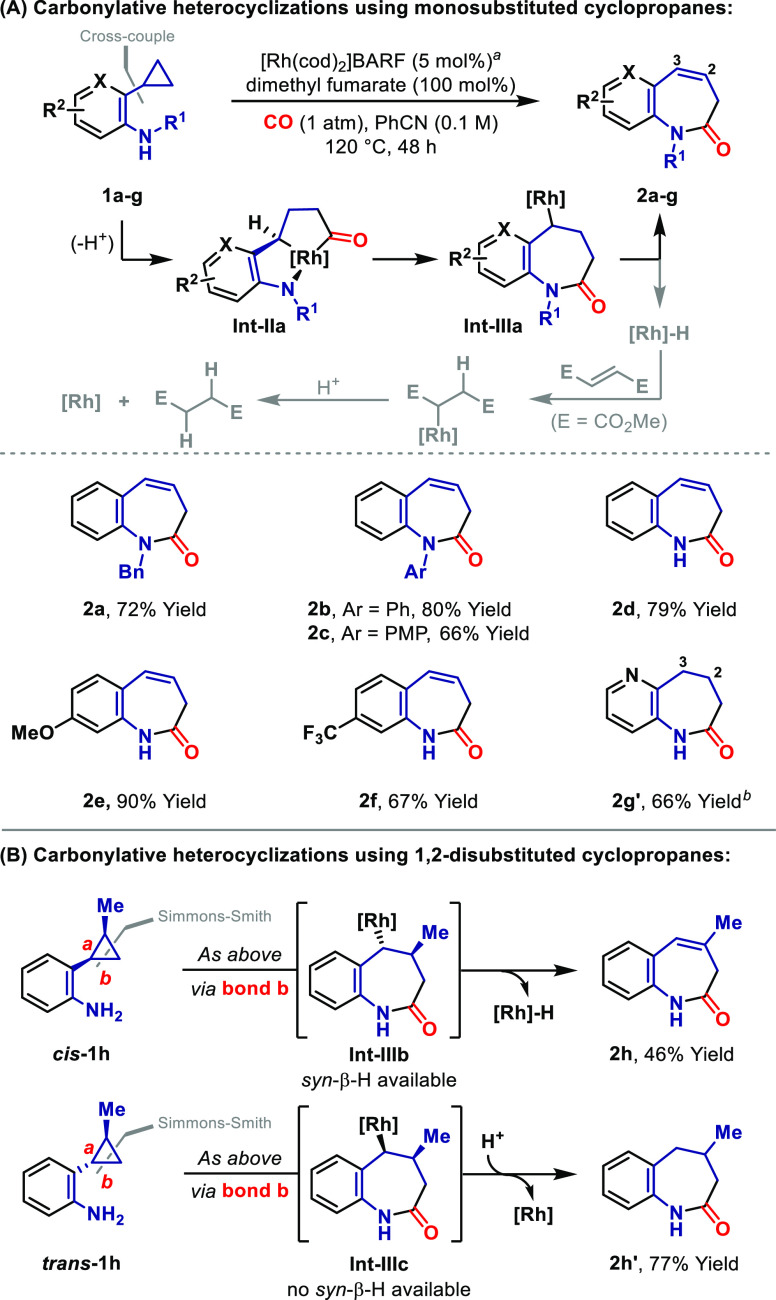
Benzazepines by N-Directed Carbonylative C–C Bond Activation BARF = tetrakis[3,5-bis(trifluoromethyl)phenyl]borate. Using [Rh] (7.5 mol %), AsPh_3_ (15 mol %) as an additive, and mesitylene as the solvent. 16% of C2–C3 unsaturated **2g** was also isolated (see the SI).

With optimized conditions in hand, the scope of the process was explored, focusing initially on the R^1^ group. Cyclizations of systems where R^1^ = aryl (**1b**,**c**) or H (**1d**) were similarly effective; these results show that the protocol tolerates an appreciably wide electronic and steric range for the directing N-center. The electronics of the aromatic unit also have minimal influence, such that cyclizations of electron-rich (**1e** to **2e**) and electron-poor systems (**1f** to **2f**) proceeded with similar levels of efficiency. Cyclization of pyridyl system **1g** did not occur under optimized conditions, perhaps because of the coordinating ability of the pyridyl nitrogen. Eventually, we established modified conditions where dimethyl fumarate was replaced by AsPh_3_ (15 mol %) and mesitylene was used as solvent. These conditions delivered **2g′**, which arises via protodemetalation of **Int-IIIa**, in 66% yield, alongside 16% yield of C2–C3 unsaturated system **2g**.^17^ The distinct oxidation level selectivity of this process is expected based on the absence of the dimethyl fumarate oxidant. Nevertheless, we have been unable to identify general conditions that allow a switch of product oxidation level.^18^ Interestingly, an alternate approach to this aspect is to program selectivity using the relative stereochemistry of 1,2-disubstituted cyclopropanes (Scheme 2B). For *cis***-1h**, N-directed rhodacycle formation and reductive elimination lead to **Int-IIIb**, where *syn*-β-hydride elimination can occur to provide unsaturated product **2h** in 46% yield. Conversely, for *trans***-1h**, reductive elimination generates diastereomeric intermediate **Int-IIIc**, where *syn*-β-hydride elimination is not possible, and so protodemetalation predominates to give saturated product **2h′**.^15^ Interestingly, for both *cis*- and *trans***-1h**, oxidative addition occurred at the more sterically accessible proximal C–C bond *b*; the selectivity of the former contrasts other C–C bond activation processes that use *cis*-1,2-disubstituted cyclopropanes.^8^

The substrates employed so far possess a degree of activation because the C–C bond that is cleaved is benzylic. The results in Scheme 2 build upon the stoichiometric studies outlined in Scheme 1C by offering unique examples where metallacycles derived from this insertion mode are integrated into productive catalysis.^11c^ Although the N-based unit plays a key role in these processes, the wider utility of this approach for achieving C–C bond activations of completely nonactivated cyclopropanes was uncertain. C–C oxidative additions involving such systems are extremely difficult and represent a frontier research challenge of the field. To probe the viability of the current strategy, we selected cyclopropane **1i**, where the electronics of the N-unit are similar to **1a** (Scheme 3A). Remarkably, exposure of **1i** to optimized conditions delivered azepine **2i** in 88% yield, albeit as a mixture of regioisomers favoring C2–C3 unsaturation. Isomerization is presumably mediated by the Rh-hydride released from **Int-IIId**, and further optimization studies indicated that suppression of this is challenging. Accordingly, we developed a telescoped protocol wherein xantphos (20 mol %) and H_2_ (1 atm) were added at the completion of the heterocyclization process to promote alkene reduction (Scheme 3B).^19^ This “in situ hydrogenation” protocol segues the Rh catalyst into a second productive process and enabled the isolation of saturated system **2i′** in 78% yield. Further studies demonstrated that these protocols can be used to convert a range of monosubstituted cyclopropanes **1j**–**p** to either unsaturated systems **2j**–**p** or saturated azepines **2j′**–**p′** with good levels of efficiency. Notably, bicyclic products can be accessed (**2p** and **2p′**), and, consistent with the proposed N-directed mechanism (cf. **1i** to **Int-IId**), bystander cyclopropanes remain intact (**2o** and **2o′**).

**Scheme 3:**
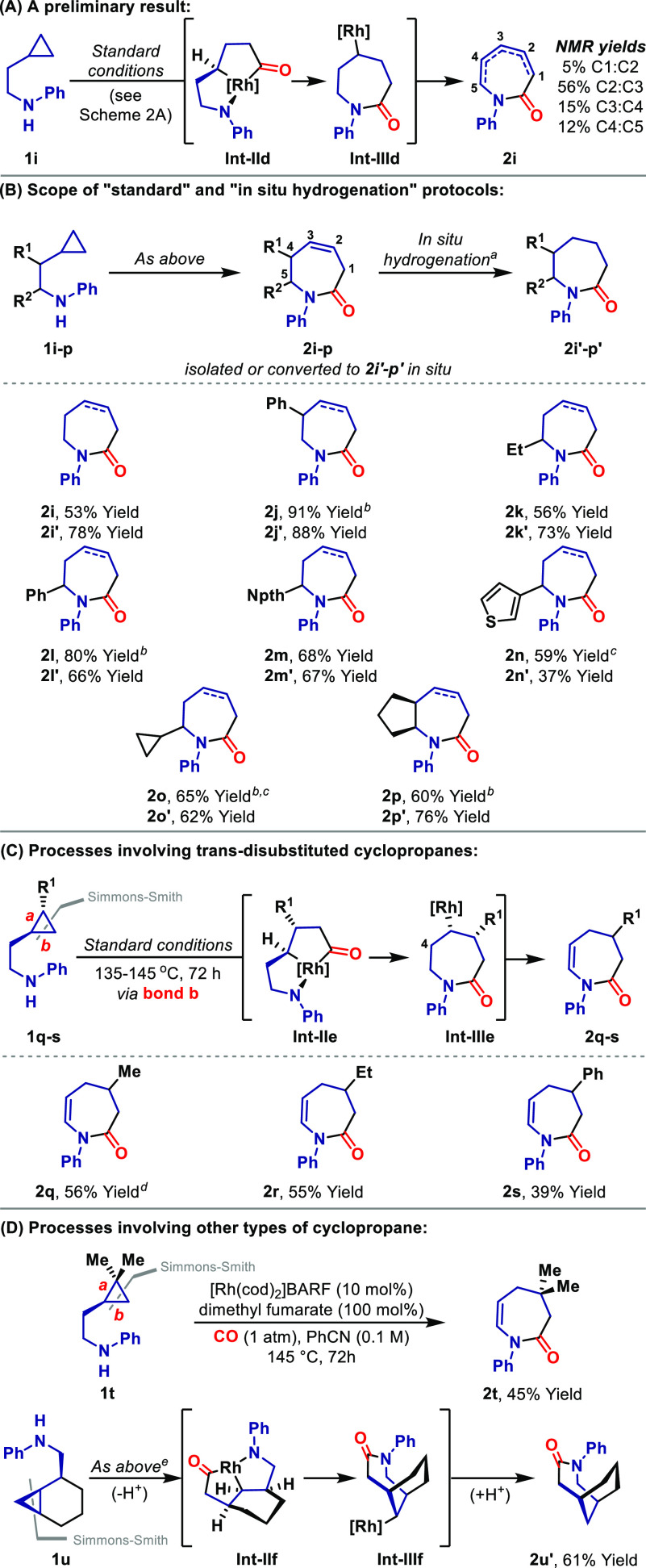
Azepines by N-Directed Carbonylative C–C Bond Activation After the carbonylation first step, xantphos (20 mol %) was added, and the mixture was heated under H_2_ (1 atm, 120 °C, 24–48 h); in some cases, a solvent swap to xylene or mesitylene was necessary (see the SI). Combined yield of a mixture of olefinic regioisomers (major isomer shown). Product ratios (C1–C2:C2–C3:C3–C4:C4-C5): **2j** (1:6:3.7:1.3), **2l** (0:8:1:0), **2o** (1:4.5:0:0), **2p** (1:1.7:0:0). The reaction temperature was 130 °C. **2q** was isolated in >99:1 er when enantioenriched **1q** (>99:1 er) was used (see the SI). 4-NO_2_C_6_H_4_CO_2_H (20 mol %) was used as an additive.

Under slightly more forcing conditions, the “standard” protocol extended to *trans*-1,2-disubstituted cyclopropanes **1q**–**s**, and these were converted, via oxidative addition of bond *b*, to cyclic enamides **2q**–**s** with useful levels of efficiency (Scheme 3C). In these cases, alkene isomerization was not problematic because the alkyl Rh(I) intermediate **Int-IIIe** is forced to undergo β-hydride elimination via C4, and this enforced directionality facilitates equilibration to give the observed and thermodynamically favored C4–C5 unsaturation. For **2s**, the non-benzylic C−C *bond b* of **1s** underwent cleavage, which contrasts the studies in Scheme 1C and highlights how the N-directing group enforces distinct regioselectivity. Even very hindered cyclopropanes participate (Scheme 3D); for example, trisubstituted system **1t** delivered unsaturated azepine **2t** in 44% yield. Similarly, bicyclic cyclopropane **1u** could be converted to the [4.3.1] ring of **2u′** in 61% yield, with the addition of 4-NO_2_C_6_H_4_CO_2_H proving beneficial in this case.^20^ Here, β-hydride elimination is not feasible at the stage of **Int-IIIf**, and so protodemetalation occurs instead to give the saturated product.^15^ These latter examples extend the state of the art significantly with respect to cyclopropane C–C bond activation scope. Notably, the starting materials are easily accessed by (directed) Simmons–Smith cyclopropanation of the corresponding alkene, and so the strategy appears to be well suited to target directed applications.^21^

A unifying feature of the processes described so far is the proposed intermediacy of N-stabilized 5,5-metallabicycles such as **Int-IIe**.^15^ The templating effect these provide, coupled with the relief of cyclopropane ring strain during their formation, facilitates ring closures that might otherwise be challenging.^22^ Accordingly, we sought to examine whether homologous intermediates (e.g., **Int-IIg**) might be harnessable because this would provide highly challenging eight-membered systems.^15^ To this end, a variety of electronically and sterically distinct N-directing units were evaluated, leading to the finding that *N*-benzhydryl system **1v** participates efficiently to provide target benzazocine **2v** in 61% yield (Scheme 4A).^23^ This was isolated as a single alkene regioisomer, favoring conjugation to the arene. The *N*-phenyl and *N*-benzyl analogues of **1v** were significantly less effective, suggesting that a strong N-directing group is required, but that this must be sufficiently sterically shielded to prevent saturation of the Rh center via polycoordination. As with earlier studies, the protocol is relatively insensitive to the electronics of the arene, such that **2w** and **2x** were generated with similar levels of efficiency. This 6,5-metallabicycle approach also extends to completely nonactivated cyclopropanes (Scheme 4B). Aniline-directed heterocyclization of **1y** provided **2y′** in 79% yield using the “in situ hydrogenation” protocol described earlier. This was necessary because the initial heterocyclization step generated **2y** (not depicted) as a 7:2 mixture of C3–C4 vs C4–C5 alkene regioisomers. The telescoped heterocyclization–reduction protocol extended smoothly to the formation of **2z′** and **2aa′**. In nondirected settings, alkyl-substituted cyclopropanes undergo preferential cleavage of the distal C–C bond;^7,9a^ the proximal selectivities observed in Scheme 3 and Scheme 4B offer strong support for an N-directed pathway.

**Scheme 4:**
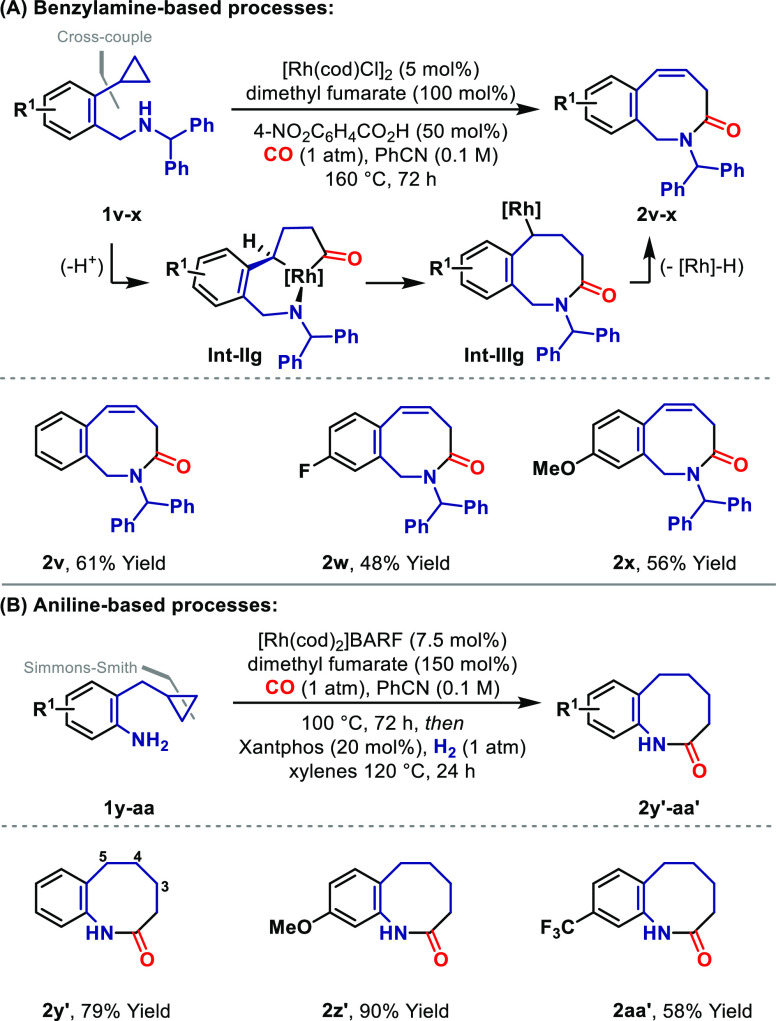
Azocines by N-Directed Carbonylative C–C Bond Activation

In summary, we outline a strategy that allows readily available nonactivated cyclopropanes to be harnessed efficiently in C–C bond activation processes. Our reaction design exploits secondary amines and anilines as strong directing groups for the carbonylative C–C bond insertion of Rh(I) systems. This allows the regiocontrolled generation of diverse amino-stabilized metallabicycles, which lead, via reductive elimination, to challenging seven- and eight-membered lactams. To the best of our knowledge, these studies encompass the first examples of catalytic processes where amines and anilines are used to direct C–C bond activation. Applications of this strong directing group strategy to other settings are currently being explored.

## Supporting Information Available

Experimental details, materials, methods, and characterization data, including copies of NMR spectra (PDF)

## References

[ref1] aSouillart L.; Cramer N. Catalytic C–C Bond Activations via Oxidative Addition to Transition Metals. Chem. Rev. 2015, 115, 9410 10.1021/acs.chemrev.5b00138. 26044343

[ref2] aBrandi A.; Cicchi S.; Cordero F. M.; Goti A. Progress in the Synthesis and Transformations of Alkylidenecyclopropanes and Alkylidenecyclobutanes. Chem. Rev. 2014, 114, 7317 10.1021/cr400686j. 24927495

[ref3] aLi C.; Zhang H.; Feng J.; Zhang Y.; Wang J. Rh(I)-Catalyzed Carbonylative Carbocyclization of Tethered Ene– and Yne–cyclopropenes. Org. Lett. 2010, 12, 3082 10.1021/ol101091r. 20536190

[ref4] aPellissier H. Recent Developments in the [5 + 2] Cycloaddition. Adv. Synth. Catal. 2011, 353, 189 10.1002/adsc.201000695.

[ref5] aMoran J.; Smith A. G.; Carris R. M.; Johnson J. S.; Krische M. J. Polarity Inversion of Donor–Acceptor Cyclopropanes: Disubstituted δ-Lactones via Enantioselective Iridium Catalysis. J. Am. Chem. Soc. 2011, 133, 18618 10.1021/ja2090993. 22026505 PMC3218199

[ref6] Under forcing conditions, electron neutral nonactivated cyclopropanes engage in carbonylative (3+1+2) cycloadditions with alkynes: Koga Y.; Narasaka K. Rhodium Catalyzed Transformation of 4-Pentynyl Cyclopropanes to Bicyclo[4.3.0]nonenones via Cleavage of Cyclopropane Ring. Chem. Lett. 1999, 28, 705 10.1246/cl.1999.705.

[ref7] aBart S. C.; Chirik P. J. Selective, Catalytic Carbon–Carbon Bond Activation and Functionalization Promoted by Late Transition Metal Catalysts. J. Am. Chem. Soc. 2003, 125, 886 10.1021/ja028912j. 12537484

[ref8] Review: Dalling A. G.; Bower J. F. Synthesis of Nitrogen Heterocycles via Directed Carbonylative C–C Bond Activation of Cyclopropanes. Chimia 2018, 72, 595 10.2533/chimia.2018.595. 30257733

[ref9] aShaw M. H.; Melikhova E. Y.; Kloer D. P.; Whittingham W. G.; Bower J. F. Directing Group Enhanced Carbonylative Ring Expansions of Amino-Substituted Cyclopropanes: Rhodium-Catalyzed Multicomponent Synthesis of N-Heterobicyclic Enones. J. Am. Chem. Soc. 2013, 135, 4992 10.1021/ja401936c. 23488745

[ref10] aShaw M. H.; Croft R. A.; Whittingham W. G.; Bower J. F. Modular Access to Substituted Azocanes via a Rhodium-Catalyzed Cycloaddition–Fragmentation Strategy. J. Am. Chem. Soc. 2015, 137, 8054 10.1021/jacs.5b05215. 26090897 PMC4508204

[ref11] aRoundhill D. M.; Lawson D. N.; Wilkinson G. New Complexes Derived from the Interaction of Dicarbonylchlororhodium(I) and Tris(triphenylphosphine)chlororhodium(I) with Cyclopropane, Butadiene, and Perfluorobutadiene. J. Chem. Soc. A 1968, 845 10.1039/j19680000845.

[ref12] aMurakami M.; Tsuruta T.; Ito Y. Lactone Formation by Rhodium-Catalyzed C–C Bond Cleavage of Cyclobutanone. Angew. Chem., Int. Ed. 2000, 39, 2484 10.1002/1521-3773(20000717)39:14<2484::AID-ANIE2484>3.0.CO;2-1. 10941110

[ref13] aSuggs J. W.; Jun C.-H. Metal-Catalyzed Alkyl Ketone to Ethyl Ketone Conversions in Chelating Ketones via Carbon-Carbon Bond Cleavage. J. Chem. Soc., Chem. Commun. 1985, 92.

[ref14] aHe C.; Whitehurst W. G.; Gaunt M. J. Palladium-Catalyzed C(sp^3^)–H Bond Functionalization of Aliphatic Amines. Chem. 2019, 5, 1031 10.1016/j.chempr.2018.12.017.

[ref15] Alternative mechanisms and supporting experiments are discussed in the SI.

[ref16] For the conversion of **1b** to **2b**, we also evaluated the use of maleic anhydride, but this led to 80% recovery of starting material. For the use of electron-deficient alkenes as additives in C–C bond activation processes, see ref (9b) and: Xia Y.; Wang J.; Dong G. Suzuki–Miyaura Coupling of Simple Ketones via Activation of Unstrained Carbon–Carbon Bonds. J. Am. Chem. Soc. 2018, 140, 5347 10.1021/jacs.8b02462. 29652498 PMC5963696

[ref17] 2-Vinyl pyridines are electronically predisposed to hydrorhodation, and this may facilitate protodemetallation by increasing access to **Int-IIIa**. For a discussion, see: Best D.; Lam H. W. C=N-Containing Azaarenes as Activating Groups in Enantioselective Catalysis. J. Org. Chem. 2014, 79, 831 10.1021/jo402414k. 24341407

[ref18] We have confirmed that the conditions used for **1g** to **2g′** are not efficient for the other examples given here.

[ref19] A solvent swap from PhCN to xylenes was necessary to prevent complications associated with hydrogenation of the former.

[ref20] In some cases, the addition of 4-NO_2_C_6_H_4_CO_2_H provided cleaner reactions. This additive may facilitate reduction of dimethyl fumarate (see Scheme 2A) or protodemetalation of the alkyl-Rh(I) intermediate (e.g., **Int-IIIf**). Both processes are otherwise dependent solely on the proton released during NH metalation. Another possibility is that a Rh-benzoate complex forms; however, the use of [Rh(cod)Cl]_2_/4-NO_2_C_6_H_4_CO_2_Ag as the precatalyst was not successful.

[ref21] aCharette A. B.; Beauchemin A. Simmons-Smith Cyclopropanation Reaction. Org. React. 2001, 58, 1 10.1002/0471264180.or058.01.

[ref22] For a discussion, see: Illuminati G.; Mandolini L. Ring Closure Reactions of Bifunctional Chain Molecules. Acc. Chem. Res. 1981, 14, 95 10.1021/ar00064a001.

[ref23] The use of [Rh(cod)Cl]_2_ is optimal for these systems. During early optimization we found that **1v** was converted predominantly to the corresponding β-methyl styrene using [Rh(cod)_2_]BARF.

