# Supplementary material for: Carbonylative N-Heterocyclization via Nitrogen-Directed C–C Bond Activation of Nonactivated Cyclopropanes
Source: J Am Chem Soc. 2022 Jun 17;144(25):11069–74. doi: 10.1021/jacs.2c02921 (PMC9248011; doi:10.1021/jacs.2c02921)
Supplement: Supplementary file 1 — ja2c02921_si_001.pdf [file ja2c02921_si_001.pdf]

Carbonylative N-Heterocyclization via Nitrogen-Directed C-C Bond Activation of Non-Activated Cyclopropanes

Adam D. J. Calow,[†] David Dailler,[‡] and John F. Bower[*,‡]

† School of Chemistry, University of Bristol, Bristol, BS8 1TS, United Kingdom

‡ Department of Chemistry, University of Liverpool, Crown Street, Liverpool, L69 7ZD, United Kingdom

Supporting Information

Table of Contents

General Experimental Details 2

Experimental Procedures and Data 3

General Procedures 3

Procedures and Characterization 6

Additional Studies and Mechanistic Considerations 49

^{1}H and ^{13}C NMR Spectra 56

General Experimental Details

Starting materials sourced from commercial suppliers were used as received unless otherwise stated. Dry solvents, where necessary, were obtained by distillation using standard procedures or by passage through a column of anhydrous alumina using equipment from Anhydrous Engineering based on the Grubb's design.[1] Petrol refers to the fraction of petroleum ether boiling in the range of 40-60 °C. The removal of solvents *in vacuo* was achieved using both a Büchi rotary evaporator (bath temperatures up to 45 °C) at a pressure of either 15 mmHg (diaphragm pump) or 0.1 mmHg (oil pump), as appropriate, and a high vacuum line at room temperature (r.t.). Reactions requiring anhydrous conditions were run under an atmosphere of dry nitrogen or argon; glassware, syringes and needles were either flame dried immediately prior to use or placed in an oven (200 °C) for at least 2 h and allowed to cool either in a desiccator or under an atmosphere of nitrogen or argon; liquid reagents, solutions or solvents were added via syringe through rubber septa; solid reagents were added via Schlenk type adapters. Commercially available Merck Kieselgel 60 F_{254} aluminum backed plates were used for TLC analysis. Visualization was achieved by either UV fluorescence, basic $KMnO_4$ solution and heat. Flash column chromatography (FCC) was performed using silica gel (Aldrich 40-63 μm, 230-400 mesh). The crude material was applied to the column as a solution in CH_2Cl_2 or by pre-adsorption onto silica, as appropriate. Melting points were determined using a Reichert melting point table and temperature controller and are uncorrected. Infra-red spectra were recorded in the range 4000-600 cm^{-1} on a Perkin Elmer Spectrum either as neat films or solids compressed onto a diamond window. Abbreviations used are: w (weak), m (medium) or s (strong). NMR spectra were recorded using either a Varian 400 MHz or Varian 500 MHz spectrometer. Chemical shifts (δ) are quoted in parts per million (ppm), coupling constants (*J*) are given in Hz to the nearest 0.5 Hz. Other abbreviations used are s (singlet), d (doublet), t (triplet), m (multiplet) and br. (broad). ^{1}H and ^{13}C NMR spectra were referenced to the appropriate residual solvent peak. ^{19}F spectra were referenced to CCl_3F as an external standard, ^{31}P spectra were referenced to H_3PO_4 as external standards. Assignments of ^{1}H NMR and ^{13}C NMR signals were made, where possible, using COSY, HMQC, HMBC, NOE and TOCSY experiments. Where mixtures of isomers (e.g. diastereomers and/or rotamers) have been characterized together, they are referred to as *A* and *B*. *Numbering systems for NMR signal assignments are specified on the structure and are not related to those used for the compound names*. Mass spectra were determined by the University of Bristol mass spectrometry service by either electron impact (ESI^+) or chemical ionization (CI^+) using a Fisons VG Analytical Autospec spectrometer.

Experimental Procedures and Data

General Procedures

General procedure A Cyclopropylmagnesium bromide solution

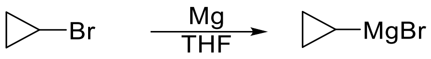

To a flame-dried flask, fitted with a magnetic stirrer bar, was added freshly prepared magnesium turnings (212 mg, 8.75 mmol). The magnesium turnings were heated under vacuum for 15 mins with vigorous stirring and the vessel was charged argon. To the flask was added anhydrous THF (10 mL) and bromocyclopropane (699 µL, 8.75 mmol). The suspension was stirred for 2 h. The resulting solution was utilized without any purification. Note: The reaction initiates upon stirring (< 10 min) and is sufficiently exothermic to avoid the need for external heating; the use of gas outlet is required due to the formation of gaseous side products.

General procedure B Synthesis of substituted non-benzo-fused aminocyclopropanes

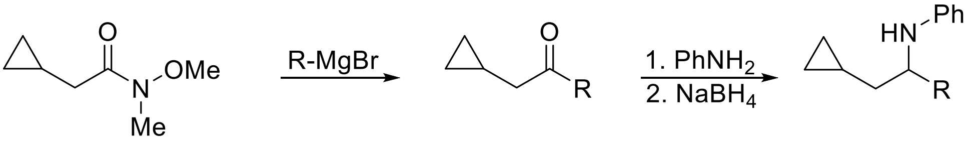

To a flame-dried flask was added 2-cyclopropyl-N-methoxy-N-methylacetamide (358 mg, 2.50 mmol) and anhydrous THF (10 mL) under argon. The resulting solution was cooled to -10 ºC. Aryl or alkyl Grignard reagent was added drop-wise over 10 min and the solution was warmed to r.t. over 3 h. H_2O (5 mL) was added slowly followed by the addition of 10% aq. HCl (2 mL). The resulting solution was vigorously stirred for 5 min, followed by the addition of EtOAc (50 mL). The resulting organic layer was washed with sat. aq. $NaHCO_3$ (15 mL), H_2O (3 × 10 mL) and brine (5 mL). The organic extracts were separated and dried over anhydrous $MgSO_4$. After filtration, the organic solvent was removed under reduced pressure to give the intermediate cyclopropylketone, which was utilized without additional purification. To the cyclopropyl ketone was added anhydrous toluene (2.5 mL), tetraisopropyl orthotitanate (740 µL, 2.5 mmol) and aniline (228 µL, 2.5 mmol). The solution was heated to reflux under argon for 18 h. Subsequently the solution was cooled to r.t. and concentrated in vacuo. To the crude material was added dichloromethane (1 mL) and MeOH (5 mL) followed by the portion-wise addition of $NaBH_4$ (150 mg, 4.0 mmol) over 5 min. The resulting suspension was stirred for 3 h. 10% aq. NaOH (5 mL) was added to the solution followed by stirring for 15 min. The solution was partitioned between EtOAc (70 mL) and the organic extracts were washed with H_2O (4 × 15 mL) and brine (15 mL). The organic layer was separated and dried over anhydrous $MgSO_4$. After filtration, the organic solvent was removed under reduced pressure to give the crude product. Purification was achieved by flash column chromatography (20:1, pentane/EtOAc).

General procedure C Standard catalytic conditions

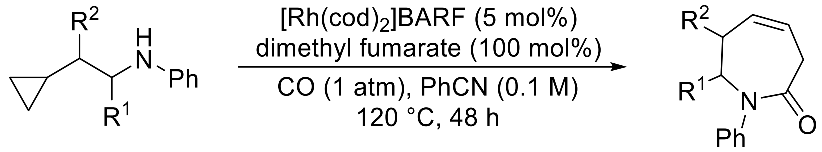

To a flame-dried reaction tube, fitted with a magnetic stirrer bar, was added $[Rh(cod)_2]BARF$ (5.9 mg, 5.0 µmol), dimethyl fumarate (14.4 mg, 0.10 mmol) and aminocyclopropane (0.10 mmol). The tube was fitted with a rubber septum and subjected to three argon/vacuum cycles. Anhydrous benzonitrile (1.0 mL) was added and the solution was subsequently sparged with carbon monoxide for *ca.* 20 seconds. The solution was heated under a carbon monoxide atmosphere (1 atm) at 120 °C for 48 h, with vigorous stirring throughout (>1000 rpm). The mixture was cooled to r.t. and concentrated *in vacuo*. Purification was achieved by flash column chromatography (10:1, pentane/EtOAc to 100% EtOAc). Note that control of the carbon monoxide sparge time can be important for optimal yields, with prolonged sparging (>20 seconds) sometimes resulting in gradually diminished yields. The starting amines were freshly prepared and it is important to ensure that all boron/aluminium by-products from previous reduction steps are removed via the basic work-ups described elsewhere.

General procedure D Standard catalytic & reductive strategy conditions

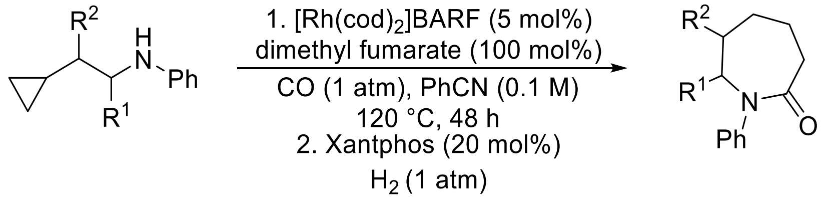

To a flame-dried reaction tube, fitted with a magnetic stirrer bar, was added $[Rh(cod)_2]BARF$ (5.9 mg, 5.0 µmol), dimethyl fumarate (14.4 mg, 0.10 mmol) and aminocyclopropane (0.10 mmol). The tube was fitted with a rubber septum and subjected to three argon/vacuum cycles. Anhydrous benzonitrile (1.0 mL) was added and the solution was subsequently sparged with carbon monoxide for *ca.* 20 seconds. The solution was heated under a carbon monoxide atmosphere (1 atm) at 120 °C for 48 h, with vigorous stirring throughout (>1000 rpm). The mixture was cooled to r.t. and the next step of the reaction was conducted in the remaining benzonitrile solution or concentrated *in vacuo*, followed by the addition of anhydrous xylenes or mesitylene (2 mL). To the solution was added xantphos (11.6 mg, 20.0 µmol) and the resulting solution was sparged with hydrogen for *ca.* 60 seconds. The solution was heated under a hydrogen atmosphere (1 atm) at 120 °C for 24 h. The mixture was cooled to r.t. and concentrated *in vacuo*. Purification was achieved by flash column chromatography.

Typical setup for the carbonylative *N*-heterocyclization reactions:

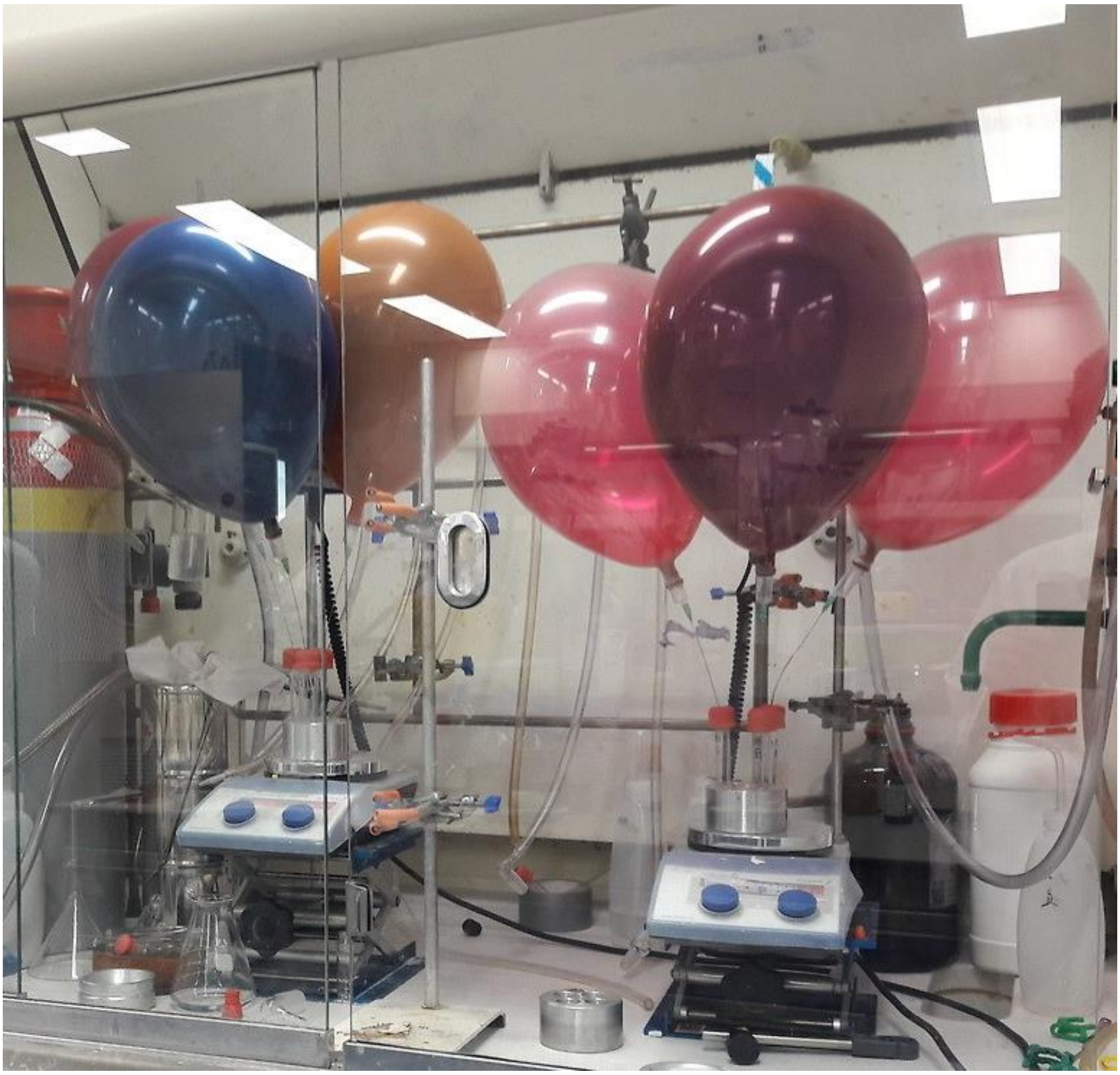

Procedures and Characterization

1-Cyclopropyl-2-nitrobenzene

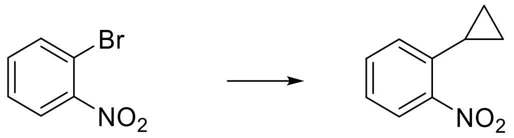

To a flame-dried reaction flask was added cyclopropylboronic acid (684 mg, 8.0 mmol), triphenylphosphine (105 mg, 0.40 mmol), tris(dibenzylideneacetone)dipalladium(0) (172 mg, 118 μmol), K_2CO_3 (1.1 g, 8.0 mmol), 1-bromo-2-nitrobenzene (808 mg, 4.0 mmol) and anhydrous toluene (30 mL). The resulting solution was sparged with argon for 15 min. After sparging, the solution was heated to reflux and vigorously stirred overnight. The solution was cooled, filtered through celite (washing with toluene) and concentrated *in vacuo*. The crude material was purified by flash column chromatography (10:1 to 5:1, hexane/EtOAc), which gave 1-cyclopropyl-2-nitrobenzene (0.52 g, 80%) as a pale yellow oil; ^{1}H NMR (400 MHz, $CDCl_3$): δ 7.79 (1H, dd, *J* = 8.0, 1.5 Hz), 7.55 – 7.42 (1H, m), 7.40 – 7.25 (1H, m), 7.25 – 7.09 (1H, m), 2.39 (1H, tt, *J* = 8.5, 5.5 Hz), 1.16 – 0.91 (2H, m), 0.82 – 0.61 (2H, m); ^{13}C NMR (101 MHz, $CDCl_3$): δ 151.3, 138.1, 132.7, 128.0, 126.5, 124.2, 12.6, 8.2; m/z (ESI$^+$) HRMS: Calculated for $C_9H_9NaNO_2$: 186.0525; Found $[M+Na]^+$: 186.0528. All physical and spectroscopic properties are consistent with those previously reported.[1]

2-Cyclopropylbenzaldehyde

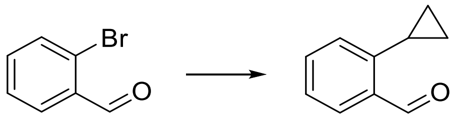

To a flame-dried reaction flask was added cyclopropylboronic acid (680 mg, 8.00 mmol), palladium-tetrakis(triphenylphosphine) (138 mg, 0.12 mmol), K_2CO_3 (1.1 g, 8.0 mmol), anhydrous xylenes (12 mL) and the resulting solution was vigorously stirred. To the stirring solution was added 2-bromobenzaldehyde (466 μL, 4.0 mmol) and the subsequent solution was sparged with argon for 15 min. After sparging, the solution was heated to 130 °C and vigorously stirred overnight. On cooling the solution was filtered through celite (washing with toluene) and concentrated *in vacuo*. The resulting crude material was purified by flash chromatography (15:1 to 10:1, hexane/EtOAc), which gave 2-cyclopropylbenzaldehyde (558 mg, 96%) as a pale yellow oil; ^{1}H NMR (400 MHz, $CDCl_3$): δ 10.61 (1H, s), 7.82 (1H, dd, *J* = 8.0, 1.5 Hz), 7.48 (1H, td, *J* = 8.0, 1.5 Hz), 7.32 (1H, td, *J* = 8.0, 1.5 Hz), 7.13 (1H, d, *J* = 8.0 Hz), 2.63 (1H, tt, *J* = 8.5, 5.5 Hz), 1.17 – 0.98 (2H, m), 0.84 – 0.70 (2H, m); ^{13}C NMR (101 MHz, $CDCl_3$): δ 192.9, 146.2, 135.0, 134.1, 130.2, 126.7, 126.2, 11.9, 8.6. All physical and spectroscopic properties are consistent with those previously reported.[2]

2-Cyclopropyl-*N*-methoxy-*N*-methylacetamide

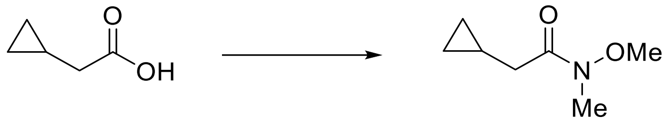

To a stirring solution of anhydrous dichloromethane (30 mL) was added cyclopropylacetic acid (2.79 mL, 30 mmol) followed by oxalyl chloride (3.1 mL, 35 mmol). Catalytic DMF was added (100 µL) and the resulting solution was stirred for 2 h. *N,O*-Dimethylhydroxylamine hydrochloride was added (3.9 g, 40 mmol) followed by the drop-wise addition of triethylamine (10 mL). The reaction was diluted with dichloromethane and washed with H_2O (50 mL), 10% aq. NaOH (3 × 15 mL), sat. aq. $NaHCO_3$ (20 mL), H_2O (50 mL) and brine (20 mL). The organic layer was separated and dried over anhydrous $MgSO_4$. Filtration and concentration *in vacuo* gave 2-cyclopropyl-*N*-methoxy-*N*-methylacetamide (4.29 g, >99%) as a yellow oil in a quantitative yield ; ν_{max} / cm^{-1}: 1660 (m), 1413 (m), 1390 (m), 1174 (m), 1009 (m); ^1H NMR (400 MHz, $CDCl_3$): δ 3.63 (3H, s), 3.15 (3H, s), 2.31 (2H, d, J = 7.0 Hz), 1.17 – 0.93 (1H, m), 0.63 – 0.41 (2H, m), 0.13 (2H, dt, J = 6.0, 5.0 Hz); ^{13}C NMR (101 MHz, $CDCl_3$): δ 174.3, 37.4, 32.2, 6.8, 4.5; m/z (ESI$^+$) HRMS: Calculated for $C_7H_{14}NO_2$: 144.1019; Found $[M+H]^+$: 144.1022.

***N*-Benzyl-2-cyclopropylaniline (1a)**

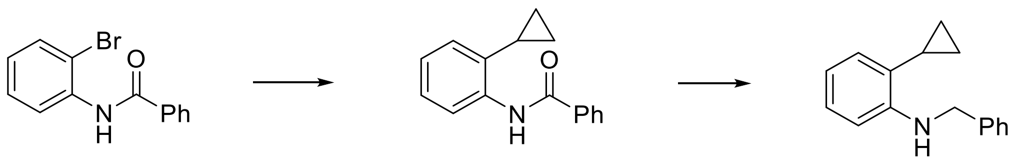

To a flame-dried reaction flask was added cyclopropylboronic acid (1.11 g, 12.9 mmol), triphenylphosphine (197 mg, 0.75 mmol), tris(dibenzylideneacetone)dipalladium (172 mg, 188 µmol), K_2CO_3 (1.8 g, 12.9 mmol), *N*-(2-bromophenyl)benzamide (2.1 g, 7.5 mmol) and anhydrous xylenes (60 mL). The resulting solution was sparged with argon for 15 min. After sparging, the solution was heated to 120 °C and vigorously stirred overnight. On cooling the solution was filtered through celite (washing with toluene) and concentrated *in vacuo*. The resulting solid was partially purified by trituration (10:1, hexane/Et_2O) and the resulting compound was utilized in the next step without additional purification. To the compound was added anhydrous THF and the solution was cooled to 0 °C under argon. Lithium aluminum hydride (569 mg, 15.0 mmol) was added slowly and the resulting suspension was stirred overnight, warming to room temperature. After stirring overnight, the solution was cooled to 0 °C. To the solution was slowly added H_2O (0.6 mL), 15% aq. NaOH (0.6 mL) and the solution was stirred for 15 min. H_2O (1.7 mL) was added followed by anhydrous $MgSO_4$ (2.0 g) and the suspension was stirred for 15 mins. The resulting precipitate was separated by filtration (washing with THF) and the organic filtrate was concentrated *n vacuo*. The crude material was purified by flash column chromatography (10:1 to 5:1, hexane/EtOAc), which gave *N*-benzyl-2-cyclopropylaniline (1.15 g, 69% over two steps) as a pale yellow oil; ν_{max} / cm^{-1} : 3436 (m), 1603 (m), 1507 (s), 1452 (m), 1026 (m); ^1H NMR (400 MHz,

$CDCl_3$): δ 7.49 – 7.25 (5H, m), 7.18 – 7.07 (2H, m), 6.70 (1H, dt, *J* = 8.4, 4.2 Hz), 6.62 (1H, d, *J* = 7.8 Hz), 4.79 – 4.63 (1H, br. s), 4.46 (2H, s), 1.67 (1H, tt, *J* = 8.5, 5.5 Hz), 1.02 – 0.80 (2H, m), 0.76 – 0.49 (2H, m); ^{13}C NMR (101 MHz, $CDCl_3$): δ 147.7, 139.9, 128.8, 128.5, 127.6, 127.5, 127.3, 126.5, 116.78, 109.8, 48.23, 11.45, 5.20; m/z (ESI^+) HRMS: Calculated for $C_{16}H_{18}N$: 224.1434; Found $[M+H]^+$: 224.1440.

2-Cyclopropyl-*N*-phenylaniline (1b)

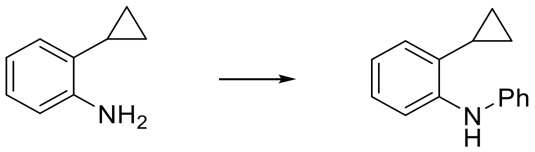

To a flame-dried reaction flask was added bromobenzene (96 µL, 0.9 mmol), SPhos (36.9 mg, 89.9 µmol), tris(dibenzylideneacetone)dipalladium(0) (20.6 mg, 22.5 µmol), sodium *tert*-butoxide (86.4 mg, 0.9 mmol), 2-cyclopropylaniline (133 mg, 1.0 mmol) and anhydrous toluene (2 mL). The resulting solution was sparged with argon for 5 min. After sparging, the solution was heated to 120 °C overnight in a sealed tube. The solution was cooled to r.t. and diluted with EtOAc (25 mL). The organic layer was washed with H_2O (3 × 10 mL), separated and subsequently dried over anhydrous $MgSO_4$. After filtration the organic layer was concentrated *in vacuo*. The crude material was purified by flash column chromatography (3:1 to 1:1, hexane/toluene), which gave 2-cyclopropyl-*N*-phenylaniline (188 mg, >99%) as a colorless oil; ν_{max} / cm^{-1} : 3411 (m), 3040 (s), 1592 (s), 1494 (s), 1026 (m); ^1H NMR (400 MHz, $CDCl_3$): δ 7.33 – 7.23 (3H, m), 7.17 – 7.06 (4H, m), 6.99 – 6.81 (2H, m), 6.16 – 5.91 (1H, br. s), 1.87 – 1.76 (1H, m), 1.02 – 0.89 (2H, m), 0.72 – 0.62 (2H, m); ^{13}C NMR (101 MHz, $CDCl_3$): δ 143.4, 142.8, 130.9, 129.3, 128.1, 126.8, 120.9, 120.6, 118.3, 116.1, 11.5, 5.8; m/z (ESI^+) HRMS: Calculated for $C_{15}H_{16}N$: 210.1277; Found $[M+H]^+$: 210.1267.

2-Cyclopropyl-*N*-(4-methoxyphenyl)aniline (1c)

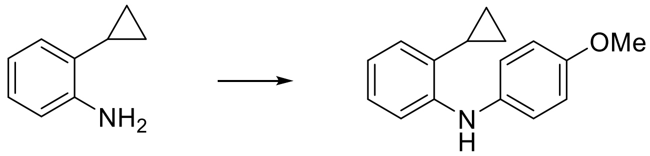

To a flame-dried reaction flask was added 4-bromoanisole (113 µL, 0.9 mmol), SPhos (36.9 mg, 89.9 µmol), tris(dibenzylideneacetone)dipalladium (20.6 mg, 22.5 µmol), sodium *tert*-butoxide (86.4 mg, 0.9 mmol), 2-cyclopropylaniline (133 mg, 1.0 mmol) and anhydrous toluene (2 mL). The resulting solution was sparged with argon for 5 min. After sparging, the solution was heated to 120 °C overnight in a sealed tube. The solution was cooled to r.t. and diluted with EtOAc (25 mL). The organic layer was washed with H_2O (3 × 10 mL), separated and subsequently dried over anhydrous $MgSO_4$. After filtration the organic layer was concentrated *in vacuo*. The crude material was purified by flash column chromatography (3:1 to 1:1, hexane/toluene), which gave 2-cyclopropyl-*N*-(4-

methoxyphenyl)anilinethe (168 mg, 78%) as a colorless solid; m.p.: 90 – 91 °C (EtOAc/pentane); ν_{max} / cm^{-1} : 3407 (m), 2963, 1578 (m), 1506 (s), 1452 (s), 1029 (m); ^{1}H NMR (400 MHz, $CDCl_3$): δ 7.19 – 7.06 (4H, m), 7.01 (1H, dd, *J* = 8.0, 1.0 Hz), 6.90 (2H, d, *J* = 9.0 Hz), 6.79 (1H, td, *J* = 7.5, 1.0 Hz), 6.15 – 5.79 (1H, br. s), 3.82 (3H, s), 1.78 (1H, tt, *J* = 8.5, 5.5 Hz), 1.04 – 0.90 (2H, m), 0.73 – 0.61 (2H, m); ^{13}C NMR (101 MHz, $CDCl_3$): δ 155.5, 145.1, 136.1, 128.7, 128.5, 127.1, 123.1, 119.1, 114.8, 113.5, 55.7, 11.6, 5.6; m/z (ESI^+) HRMS: Calculated for $C_{16}H_{18}NO$: 240.1383; Found $[M+H]^+$: 240.1386.

2-Cyclopropylaniline (1d)

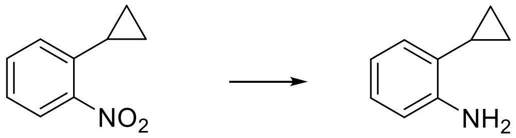

To a flask containing 1-cyclopropyl-2-nitrobenzene (500 mg, 3.1 mmol) was added Fe powder (0.52 g, 9.3 mmol) and EtOH (20 mL). aq. HCl (6 mL, 2M) was added and the suspension was heated to reflux for 2 h. On cooling, the solution was neutralized by the addition of 10% aq. NaOH. The solution was diluted with EtOAc (60 mL) and the organic layer was separated. The aqueous layer was extracted with EtOAc (3 × 30 mL) and the organic extracts were combined and dried over anhydrous $MgSO_4$. After filtration the organic layer was concentrated *in vacuo*. The crude oil was purified by flash column chromatography (15:1 to 5:1, hexane/EtOAc), which gave 2-cyclopropylaniline (328 mg, 79%) as a colorless oil; ν_{max} / cm^{-1}: 1614 (m), 1496 (m), 1454 (m), 1270, 1050 ; ^{1}H NMR (400 MHz, $CDCl_3$): δ 7.08 – 7.00 (2H, m), 6.74 – 6.65 (2H, m), 4.12 – 3.78 (2H, br. s), 1.69 (1H, tt, *J* = 8.5, 5.5 Hz), 0.96 – 0.84 (2H, m), 0.65 – 0.56 (2H, m); ^{13}C NMR (101 MHz, $CDCl_3$): δ 146.3, 128.4, 127.1, 126.8, 118.1, 114.5, 11.4, 5.0; m/z (ESI^+) HRMS: Calculated for $C_9H_{12}N$: 134.0964; Found $[M+H]^+$: 134.0962.

2-Cyclopropyl-5-methoxyaniline (1e)

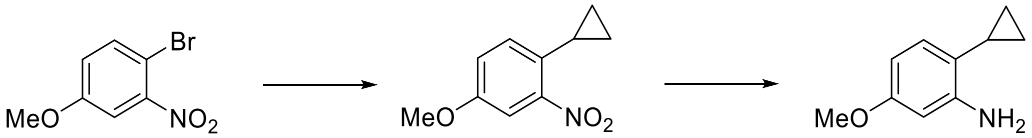

To a flame-dried reaction tube was added palladium(II) acetate (28 mg, 0.11 mmol), cyclopropylboronic acid (0.43 g, 5.0 mmol), tricyclohexylphosphine (70.1 mg, 0.25 mmol), tripotassium phosphate (1.6 g, 7.5 mmol), 1-bromo-4-methoxy-2-nitrobenzene (580 mg, 2.5 mmol) and anhydrous toluene (8 mL), The resulting suspension was sparged with argon for 15 and subsequently heated to 100 °C overnight with vigorous stirring. On cooling, 10% aq. NaOH (20 mL) was added and the organic products were extracted into toluene and washed with H_2O (3 × 15 mL) and brine (15 mL). The organic layer was dried over anhydrous $MgSO_4$, filtered and concentrated *in vacuo*. To the crude oil was added EtOH (3 mL). Raney nickel (*ca*. 266 mg, 3.11 mmol) was added to the solution followed by the drop-wise addition of hydrazine hydrate (294 µL, 9.43 mmol). The suspension was stirred vigorously for 3 h, after which the solution was decanted (washing with EtOH) and concentrated *in vacuo*. Purification was

achieved by flash column chromatography (10:1 to 1:1, petroleum ether 40-60 °C/EtOAc), which gave 2-cyclopropyl-5-methoxyaniline (343 mg, 84% over two steps) as an orange oil; ν_{max} / cm^{-1} : 3470 (m), 3376 (m), 1618 (m), 1579 (m), 1509 (s), 1319 (m), 1025 (m); ^{1}H NMR (400 MHz, $CDCl_3$): δ 6.95 (1H, d, *J* = 9.5 Hz), 6.35 – 6.18 (2H, m), 3.75 (3H, s), 1.68 – 1.52 (1H, m), 0.95 – 0.82 (2H, m), 0.64 – 0.48 (2H, m); ^{13}C NMR (101 MHz, $CDCl_3$): δ 159.2, 147.2, 129.5, 119.7, 103.2, 100.5, 55.2, 10.7, 4.9; m/z (ESI^+) HRMS: Calculated for $C_{10}H_{14}NO$: 164.1070; Found $[M+H]^+$: 164.1076.

2-Cyclopropyl-5-(trifluoromethyl)aniline (1f)

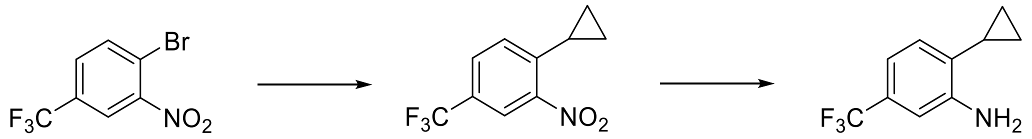

To a flame-dried reaction tube was added palladium(II) acetate (28 mg, 0.11 mmol), cyclopropylboronic acid (0.43 g, 5.0 mmol), tricyclohexylphosphine (70.1 mg, 0.25 mmol), tripotassium phosphate (1.6 g, 7.5 mmol), 1-bromo-2-nitro-4-(trifluoromethyl)benzene (675 mg, 2.5 mmol) and anhydrous toluene (8 mL), The resulting suspension was sparged with argon for 15 and subsequently heated to 100 °C overnight with vigorous stirring. On cooling, 10% aq. NaOH (20 mL) was added and the organic products were extracted into toluene and washed with H_2O (3 × 15 mL) and brine (15 mL). The organic layer was dried over anhydrous $MgSO_4$, filtered and concentrated *in vacuo*. To the crude oil was added EtOH (3 mL). Raney nickel (*ca*. 266 mg, 3.11 mmol) was added to the solution followed by the drop-wise addition of hydrazine hydrate (294 µL, 9.43 mmol). The suspension was stirred vigorously for 3 h, after which the solution was decanted (washing with EtOH) and concentrated *in vacuo*. The resulting oil was purified by flash column chromatography (toluene), which gave 2-cyclopropyl-5-(trifluoromethyl)aniline (433 mg, 86% over two steps) as a pale yellow oil; ν_{max} / cm^{-1} : 3486 (m), 3397 (m), 1624 (m), 1582(s), 1436 (m), 1333 (m); ^{1}H NMR (400 MHz, $CDCl_3$): δ 7.11 (1H, d, *J* = 8.0 Hz), 6.93 (1H, d, *J* = 8.0 Hz), 6.90 (1H, s), 4.39 – 3.99 (1H, br. s), 1.76 – 1.60 (1H, m), 1.01 – 0.83 (2H, m), 0.65 – 0.50 (2H, m); ^{13}C NMR (101 MHz, $CDCl_3$): δ 146.6, 130.4, 129.6 (q, *J* = 32.0 Hz), 128.8, 124.4 (q, *J* = 272.8 Hz), 114.7 (q, *J* = 3.9 Hz), 110.8 (q, *J* = 3.9 Hz), 11.5, 5.3; ^{19}F NMR (377 MHz, $CDCl_3$): δ -62.5; m/z (ESI^+) HRMS: Calculated for $C_{10}H_{11}F_3N$: 202.0838; Found $[M+H]^+$: 202.0837.

2-Cyclopropylpyridin-3-amine (1g)

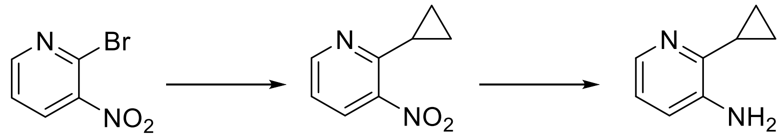

To a flame-dried reaction tube was added palladium(II) acetate (28 mg, 0.11 mmol), cyclopropylboronic acid (0.43 g, 5.0 mmol), triphenylphosphine (65 mg, 0.25 mmol), tripotassium phosphate (1.6 g, 7.5 mmol), 2-bromo-3-nitropyridine (508 mg, 2.5 mmol) and anhydrous toluene (8 mL), The resulting suspension was sparged with argon for 15 and subsequently heated to 100 °C overnight with vigorous

stirring. On cooling, 10% aq. NaOH (20 mL) was added and the organic products were extracted into toluene and washed with H_2O (3 × 15 mL) and brine (15 mL). The organic layer was dried over anhydrous $MgSO_4$, filtered and concentrated *in vacuo*. To the crude oil was added EtOH (3 mL). Raney nickel (*ca*. 266 mg, 3.11 mmol) was added to the solution followed by the drop-wise addition of hydrazine hydrate (294 µL, 9.43 mmol). The suspension was stirred vigorously for 3 h, after which the solution was decanted (washing with EtOH) and concentrated *in vacuo*. Purification was achieved by flash column chromatography (4:1 to 1:1, petroleum ether 40-60 °C/EtOAc), which gave 2-cyclopropylpyridin-3-amine (210 mg, 63% over two steps) as an colorless solid; m.p.: 84 – 86 °C (EtOAc/Hex); ν_{max} / cm^{-1} : 3385 (m), 3310 (m), 1644, 1586 (m), 1458 (s), 1452 (m), 1298 (m); ^{1}H NMR (400 MHz, $CDCl_3$): δ 7.93 (1H, t, *J* = 3.0 Hz), 6.96 – 6.89 (2H, m), 3.91 – 3.73 (2H, br. s), 1.85 (1H, tt, *J* = 8.0, 5.5 Hz), 1.04 – 0.93 (4H, m); ^{13}C NMR (101 MHz, $CDCl_3$): δ 147.1, 141.3, 139.3, 121.6, 121.3, 12.5, 6.5; m/z (ESI^+) HRMS: Calculated for $C_8H_{11}N_2$: 135.0917; Found $[M+H]^+$: 135.0922.

(*rac*)-(*cis*)-2-(2-Methylcyclopropyl)aniline (*cis*-1h)

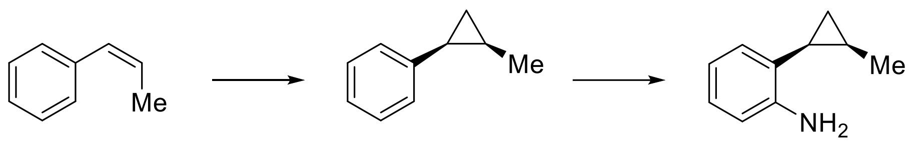

To a flame-dried flask was added anhydrous dichloromethane (20 mL), *cis*-1-phenyl-1-propene (649 µL, 5.0 mmol) and diiodomethane (2.0 mL, 25.0 mmol). The solution was cooled to -10 °C and stirred under argon. Diethyl zinc in hexane (25 mL, 1.0 M) was added dropwise over 10 min and the resulting solution was warmed to r.t. over 1 h. The reaction was further cooled to 0 °C and sat. aq. NaEDTA (10 mL) was added dropwise (Note: care should be taken when quenching excess diethyl zinc). The resulting suspension was diluted with EtOAc (50 mL) and washed with H_2O, brine and the organic layer was dried over anhydrous $MgSO_4$. After filtration, the solution was concentrated in vacuo to give the pure syn-(2-methylcyclopropyl)benzene as a colorless clear oil. The syn-(2-methylcyclopropyl)benzene (264 mg, 2.0 mmol) and acetic anhydride (2 mL) were combined and cooled to -42 °C. In a separate vessel, nitric acid 70% (100 µL, 3.23 mmol) was added to acetic anhydride (1 mL) and was subsequently stirred for 5 min at low temperature (-42 °C). Both solutions were combined and stirred for 5 min. Concentrated H_2SO_4 (25 µL) was added and the solution was stirred for 1 h, warming to r.t. over 30 mins. H_2O (3 mL) was added followed by 10% aq. NaOH (1 mL). The solution was diluted with toluene (100 mL) and subsequently washed with 10% aq. NaOH (1 × 10mL), H_2O (3 × 20 mL) and brine (1 × 20 mL). The organic layer was dried over anhydrous $MgSO_4$, filtered and concentrated *in vacuo* to give inseparable *o*/*p*-nitrated products in quantitative yield. To the resulting oil was added Fe powder (280 mg, 5.0 mmol), EtOH (8 mL) and acetic acid (2.5 mL). The resulting suspension was heated to reflux for 2 h. On cooling, the solution was neutralized by the addition of 10% aq. NaOH. The solution was diluted with EtOAc (25 mL) and the organic layer was separated. The aqueous layer was extracted with EtOAc (3 × 30 mL) and the organic extracts were combined and dried over anhydrous $MgSO_4$. After

filtration the organic layer was concentrated *in vacuo*. The resulting oil was purified by flash column chromatography (1:1 pentane/toluene), which (*rac*)-(*cis*)-2-(2-Methylcyclopropyl)aniline (71 mg, 24% over three steps) as a colorless clear oil; ^{1}H NMR (400 MHz, $CDCl_3$): 7.14 – 6.93 (2H, m), 6.86 – 6.62 (2H, m), 4.07 – 3.71 (2H, br. s), 1.83 – 1.69 (1H, m), 1.34 – 1.09 (1H, m), 1.02 (1H, td, J = 8.5, 4.5 Hz), 0.80 (3H, d, J = 6.0 Hz), 0.54 (1H, q, J = 5.5 Hz); ^{13}C NMR (101 MHz, $CDCl_3$): δ 146.7, 130.2, 127.2, 123.9, 118.1, 114.5, 17.4, 13.6, 11.3, 10.6. All physical and spectroscopic properties are consistent with those previously reported.[3]

(*rac*)-(*trans*)-2-(2-Methylcyclopropyl)aniline (*trans*-1h)

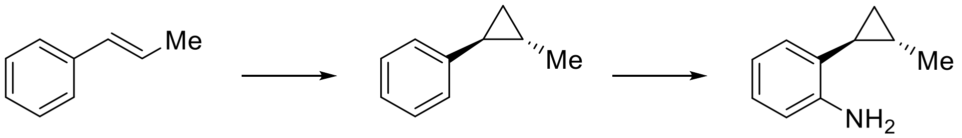

To a flame-dried flask was added anhydrous dichloromethane (20 mL), *trans*-1-phenyl-1-propene (649 µL, 5.0 mmol) and diiodomethane (2.0 mL, 25.0 mmol). The solution was cooled to -10 °C and stirred under argon. Diethyl zinc in hexane (25 mL, 1.0 M) was added dropwise over 10 min and the resulting solution was warmed to r.t. over 1 h. The reaction was further cooled to 0 °C and sat. aq. NaEDTA (10 mL) was added dropwise (Note: care should be taken when quenching excess diethyl zinc). The resulting suspension was diluted with EtOAc (50 mL) and washed with H2O, brine and the organic layer was dried over anhydrous MgSO4. After filtration, the solution was concentrated in vacuo to give the crude cyclopropane as off-colorless oil. The crude cyclopropane and acetic anhydride (2 mL) were combined and cooled to -42 °C. In a separate vessel, nitric acid 70% (183 µL, 5.92 mmol) was added to acetic anhydride (2 mL) and was subsequently stirred for 5 min at low temperature (-42 °C). Both solutions were combined and stirred for 5 min. Concentrated H_2SO_4 (25 µL) was added and the solution was stirred for 1 h, warming to r.t. over 30 mins. H_2O (3 mL) was added followed by 10% aq. NaOH (1 mL). The solution was diluted with toluene (100 mL) and subsequently washed with 10% aq. NaOH (1 × 10mL), H_2O (3 × 20 mL) and brine (1 × 20 mL). The organic layer was dried over anhydrous $MgSO_4$, filtered and concentrated *in vacuo* to give inseparable *o*/*p*-nitrated products in quantitative yield. The resulting mixture was added to a stirring solution of EtOH (3 mL). Raney nickel (*ca*. 266 mg, 3.11 mmol) was added to the solution followed by the drop-wise addition of hydrazine hydrate (294 µL, 9.43 mmol). The suspension was stirred vigorously for 3 h, after which the solution was decanted (washing with EtOH) and concentrated *in vacuo*. The resulting oil was purified by flash column chromatography (1:1 pentane/toluene), which gave (*rac*)-(*trans*)-2-(2-methylcyclopropyl)aniline (142 mg, 19% over three steps) as a colorless clear oil; ^{1}H NMR (400 MHz, $CDCl_3$): δ 7.10 – 6.93 (2H, m), 6.75 – 6.55 (2H, m), 3.96 – 3.76 (2H, br. s), 1.46 – 1.33 (1H, m), 1.26 (3H, d, J = 6.0 Hz), 1.03 – 0.93 (1H, m), 0.87 – 0.74 (1H, m), 0.74 – 0.57 (1H, m); ^{13}C NMR (101 MHz, $CDCl_3$): δ 146.2, 128.4, 127.2, 127.1, 118.3, 114.7, 20.3, 19.3, 13.7. All physical and spectroscopic properties are consistent with those previously reported.[3]

***N*-(2-Cyclopropylethyl)aniline (1i)**

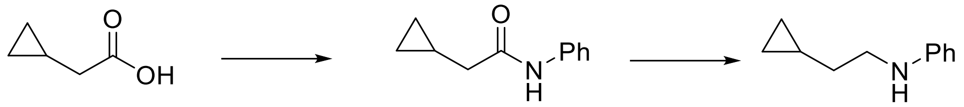

To a stirring solution of anhydrous dichloromethane (20 mL) was added cyclopropylacetic acid (930 µL, 10.0 mmol) and oxalyl chloride (986 µL, 11.5 mmol). DMF was added (25 µL) and the solution was stirred for 2 h. The solution was concentrated under reduced pressure and to the resulting mass was added anhydrous dichloromethane (30 mL). Aniline (0.90 mL, 9.9 mmol) was added slowly, resulting in the formation of a white precipitate. Triethylamine (2 mL) was added drop-wise, resulting in the formation of a clear a yellow solution, which was stirred for 1 h. The solution was diluted with EtOAc (80 mL) and washed with H_2O (30 mL), 10% aq. NaOH (3 × 15 mL), H_2O (50 mL) and brine (20 mL). The organic layer was separated, dried over anhydrous $MgSO_4$ and subsequently filtered. After concentrating under reduced pressure, the resulting mass was dissolved in THF (5 mL). To this solution was added borane in THF (10 mL, 1.0 M) and the solution was heated to reflux for 3 h. After cooling to r.t., 10% aq. NaOH (10 mL) was added slowly, and the mixture was further heated to reflux for 1 h. After cooling to r.t., the mixture was diluted with EtOAc (50 mL) and the organic layer was washed with H_2O (2 × 20 mL) and brine (20 mL). The organic layer was separated, dried over anhydrous $MgSO_4$ and filtered. After concentrating the crude material was purified by flash column chromatography (15:1 to 5:1, pentane/EtOAc), which gave *N*-(2-cyclopropylethyl)aniline (1.22 g, 76% over three steps) as a colorless clear oil; ν_{max} / cm^{-1} : 3410 (m), 1601 (m), 1504 (m), 1316 (m); ^1H NMR (400 MHz, $CDCl_3$): δ 7.20 – 7.16 (2H, m), δ 6.71 – 6.69 (1H, m), 6.74 – 6.61 (2H, m), 3.96 – 3.62 (1H, br. s), 3.21 (2H, t, *J* = 7.0 Hz), 1.53 (2H, q, *J* = 7.0 Hz), 0.82 – 0.69 (1H, m), 0.51 – 0.45 (2H, m), 0.13 – 0.07 (2H, m); ^{13}C NMR (101 MHz, $CDCl_3$): δ 148.6, 129.4, 117.3, 113.0, 44.3, 34.6, 8.9, 4.4; m/z (ESI^+) HRMS: Calculated for $C_{11}H_{16}N$: 162.1277; Found $[M+H]^+$: 162.1280.

***N*-(2-Cyclopropyl-2-phenylethyl)aniline (1j)**

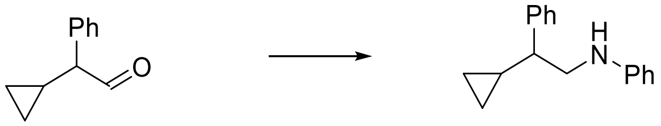

To a flame-dried flask was added 2-cyclopropyl-2-phenylacetaldehyde[4] (0.45 g, 2.6 mmol), 3 Å molecular sieve pellets (1.0 g), aniline (270 µL, 3.0 mmol) and anhydrous THF (6 mL). The solution was stirred for 2 h under argon and was subsequently filtered and concentrated under reduced pressure. MeOH (5 mL) was added followed by the portion-wise addition of $NaBH_4$ (190 mg, 5.0 mmol) over 15 min. The resulting suspension was stirred for 18 h. 10% aq. NaOH (25 mL) was added to the solution followed by stirring for 15 min. The organic products were extracted into EtOAc (3 × 15 mL). The organic extracts were dried over anhydrous $MgSO_4$, filtered and concentrated *in vacuo*. Purification was achieved by flash column chromatography (3:1, hexane/toluene to toluene), which gave *N*-(2-cyclopropyl-2-phenylethyl)aniline (305 mg, 49% over two steps) as a pale yellow oil; ν_{max} / cm^{-1} : 3412

(m), 1600 (s), 1504 (s), 1259 (m), 909 (m); ^{1}H NMR (400 MHz, $CDCl_3$): δ 7.36 – 7.24 (5H, m,), 7.16 (2H, t, J = 8.0 Hz), 6.69 (1H, t, J = 7.5 Hz), 6.58 (2H, d, J = 8.5 Hz), 3.78 – 3.59 (1H, m), 3.63 – 3.36 (2H, br. s), 2.23 – 2.08 (1H, m), 1.15 – 0.99 (1H, m), 0.75 – 0.61 (1H, m), 0.49 – 0.40 (1H, m), 0.40 – 0.29 (1H, m), 0.19 – -0.02 (1H, m); ^{13}C NMR (101 MHz, $CDCl_3$): δ 148.3, 143.3, 129.4, 128.7, 127.9, 126.8, 117.5, 113.2, 50.2, 49.9, 15.3, 5.6, 3.5; m/z (ESI$^+$) HRMS: Calculated for $C_{17}H_{20}N$: 238.1590; Found $[M+H]^+$: 238.12600.

***N*-(1-Cyclopropylbutan-2-yl)aniline (1k)**

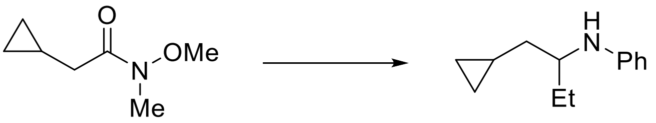

General procedure B: Ethylmagnesium bromide in diethyl ether solution was utilized (1.5 mL, 3.0 M). The crude mixture was purified by flash column chromatography (20:1 to 10:1 pentane/EtOAc), to yield *N*-(1-cyclopropylbutan-2-yl)aniline (74 mg, 16% over three steps) as a pale yellow oil; ν_{max} / cm^{-1}: 3404 (m), 1600 (s). 1503 (s). 1318 (m), 1277 (m); ^{1}H NMR (400 MHz, $CDCl_3$): δ 7.15 (2H, t, J = 7.5 Hz), 6.68 – 6.55 (3H, m), 3.63 – 3.46 (1H, br. s), 3.46 – 3.34 (1H, m), 1.75 – 1.62 (1H, m), 1.55 – 1.45 (2H, m), 1.39 – 1.29 (1H, m), 0.95 (3H, t, J = 7.5 Hz), 0.80 – 0.67 (1H, m), 0.51 – 0.37 (2H, m), 0.15 – -0.01 (2H, m); ^{13}C NMR (101 MHz, $CDCl_3$): δ 148.3, 129.4, 116.7, 113.1, 54.90, 39.4, 27.4, 10.4, 7.8, 4.8, 4.6; m/z (ESI$^+$) HRMS: Calculated for $C_{13}H_{20}N$: 190.1590; Found $[M+H]^+$: 190.1598.

***N*-(2-Cyclopropyl-1-phenylethyl)aniline (1l)**

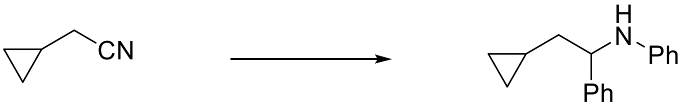

To a flame-dried flask was added anhydrous toluene (15 mL) and cyclopropylacetonitrile (460 µL, 5.0 mmol) under argon. To the solution was added phenylmagnesium bromide in diethyl ether (3.0 mL, 3.0 M) and the subsequent solution was heated to 100 °C overnight under argon. After cooling to r.t., H_2O (10 mL) was added followed by the addition of 10% aq. HCl (3 mL). The subsequent solution was vigorously stirred for 20 min. The solution was diluted with EtOAc (100 mL) and washed with H_2O (20 mL), 10% aq. NaOH (1 × 15 mL), sat. aq. $NaHCO_3$ (10 mL), H_2O (10 mL) and brine (5 mL). The organic layer was separated and dried over anhydrous $MgSO_4$. After filtration and concentrating under reduced pressure, the residual oil was partially purified using a silica gel plug (20:1 to 10:1, hexane/EtOAc) which gave 2-cyclopropyl-1-phenylethan-1-one as a pale-yellow oil (0.61 g, 76%). To a flame-dried flask was added 2-cyclopropyl-1-phenylethan-1-one (0.61, 3.8 mmol), 3 Å molecular sieve pellets (3.0 g), aniline (365 µL, 4.0 mmol) and anhydrous toluene (15 mL). The solution was heated to reflux for 24 h under argon. On cooling the solution was filtered and concentrated under reduced pressure. MeOH (10 mL) was added followed by the portion-wise addition of $NaBH_4$ (0.36 g, 9.5 mmol) over 15 min. The resulting suspension was stirred for 1.5 h. 10% aq. NaOH (25 mL) was

added to the solution followed by stirring for 15 min. The organic products were extracted into EtOAc (3 × 25 mL). The organic extracts were dried over anhydrous $MgSO_4$, filtered and concentrated *in vacuo*. Purification was achieved by flash column chromatography (25:1, hexane/EtOAc), which gave *N*-(2-cyclopropyl-1-phenylethyl)aniline (438 mg, 49%) as a yellow oil; ν_{max} / cm^{-1} : 3413 (m), 1600 (s), 1502 (s), 1315 (m), 1017; ^{1}H NMR (400 MHz, $CDCl_3$): δ 7.41 – 7.19 (5H, m), 7.09 (2H, t, *J* = 8.0 Hz), 6.64 (1H, t, *J* = 7.5 Hz), 6.53 (2H, d, *J* = 7.5 Hz), 4.42 (1H, t, *J* = 7.0 Hz), 4.35 – 4.24 (1H, br. s), 1.82 – 1.61 (2H, m), 0.79 – 0.61 (1H, m), 0.61 – 0.34 (2H, m), 0.23 – -0.01 (2H, m); ^{13}C NMR (101 MHz, $CDCl_3$): δ 147.7, 144.3, 129.2, 128.6, 127.0, 126.5, 117.3, 113.5, 59.1, 44.0, 8.26, 4.8, 4.6; m/z (ESI^+) HRMS: Calculated for $C_{17}H_{20}N$: 238.1590; Found $[M+H]^+$: 238.1596.

***N*-(2-Cyclopropyl-1-(naphthalen-2-yl)ethyl)aniline (1m)**

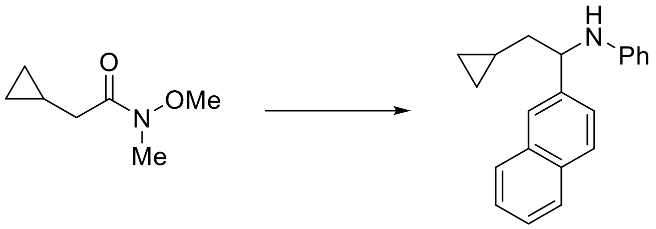

General procedure B: 2-Naphthylmagnesium bromide in THF solution was utilized (7.0 mL, 0.5 M). The crude mixture was purified by flash column chromatography (20:1 to 10:1 pentane/EtOAc), to yield *N*-(2-cyclopropyl-1-(naphthalen-2-yl)ethyl)aniline (156 mg, 22% over three steps) as a yellow solid; m.p.: 66 – 68 °C (EtOAc/Hex); ν_{max} / cm^{-1} : 3410 (m), 1599 (m), 1505 (m), 1320 (m), 1100 (m); ^{1}H NMR (400 MHz, $CDCl_3$): δ 7.90 – 7.77 (4H, m), 7.58 – 7.51 (1H, m), 7.50 – 7.39 (2H, m), 7.16 – 7.01 (2H, m), 6.64 (1H, t, *J* = 7.5 Hz), 6.58 (2H, d, *J* = 8.5 Hz), 4.59 (1H, t, *J* = 7.0 Hz), 4.51 – 4.31 (1H, m), 1.90 – 1.67 (2H, m), 0.84 – 0.67 (1H, m), 0.64 – 0.40 (2H, m), 0.20 – 0.03 (2H, m); ^{13}C NMR (101 MHz, $CDCl_3$): δ 147.7, 141.8, 133.7, 132.9, 129.2, 128.4, 128.0, 127.8, 126.1, 125.6, 125.1, 124.9, 117.4, 113.6, 59.3, 43.9, 8.3, 4.9, 4.6; m/z (ESI^+) HRMS: Calculated for $C_{21}H_{22}N$: 288.1747; Found $[M+H]^+$: 288.1752.

***N*-(2-Cyclopropyl-1-(thiophen-3-yl)ethyl)aniline (1n)**

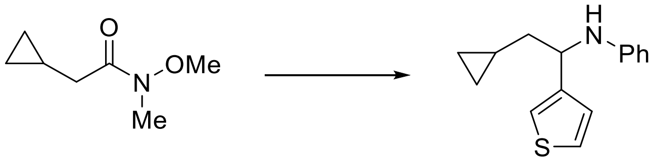

General procedure B: Thiophen-3-ylmagnesium bromide in THF solution was utilized (5.0 mL, 1.0 M). The crude mixture was purified by flash column chromatography (20:1 to 10:1, petroleum ether 40-60 °C/EtOAc), to yield *N*-(2-cyclopropyl-1-(thiophen-3-yl)ethyl)aniline (149 mg, 24% over three steps) as a pale brown oil; ν_{max} / cm^{-1} : 3408 (m), 1600 (m), 1501 (m), 1313 (m), 1179; ^{1}H NMR (400 MHz, $CDCl_3$): δ 7.28 – 7.27 (1H, m), 7.17 – 7.03 (4H, m), 6.67 (1H, t, *J* = 7.5 Hz), 6.58 (2H, d, *J* = 8.5 Hz), 4.57 (1H, t, *J* = 6.5 Hz), 4.23 – 4.10 (1H, br. s), 1.75 (2H, t, *J* = 7.0 Hz), 0.78 – 0.66 (1H, m), 0.59 – 0.36 (2H, m), 0.20 – 0.01 (2H, m); ^{13}C NMR (101 MHz, $CDCl_3$): δ 147.7, 145.7, 129.3, 126.4, 125.9,

120.7, 117.5, 113.5, 55.0, 42.8, 8.1, 4.7, 4.6; m/z (ESI$^+$) HRMS: Calculated for $C_{15}H_{18}NS$: 244.1154; Found $[M+H]^+$: 244.1151.

***N*-(1,2-Dicyclopropylethyl)aniline (1o)**

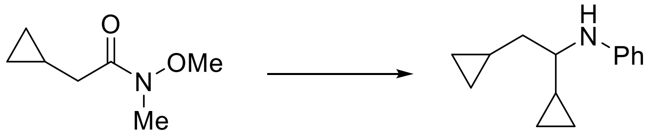

General procedure B: Cyclopropylmagnesium bromide solution was utilized; prepared according to the procedure outlined in **General procedure A**. The crude mixture was purified by flash column chromatography (20:1 pentane/EtOAc), to yield *N*-(1,2-dicyclopropylethyl)aniline (99 mg, 20% over three steps) as a pale yellow oil; ν_{max} / cm^{-1} : 3409 (m), 1601 (m), 1502 (m), 1318 (m), 1015 (m); ^1H NMR (400 MHz, $CDCl_3$): δ 7.20 – 7.07 (2H, m), 6.65 (1H, t, *J* = 7.0 Hz), 6.59 (2H, d, *J* = 8.0 Hz), 3.84 – 3.55 (1H, m), 2.93 (1H, q, *J* = 6.0 Hz), 1.53 (2H, t, *J* = 6.5 Hz), 1.04 – 0.91 (1H, m), 0.88 – 0.82 (1H, m), 0.61 – 0.37 (4H, m), 0.35 – 0.27 (2H, m), 0.11 – 0.06 (2H, m); ^{13}C NMR (101 MHz, $CDCl_3$): δ 148.4, 129.3, 116.9, 113.2, 57.7, 40.7, 16.5, 7.9, 4.9, 4.7, 3.8, 2.7; m/z (ESI$^+$) HRMS: Calculated for $C_{14}H_{20}N$: 202.1590; Found $[M+H]^+$: 202.1591.

(*rac*)-(*cis*)-*N*-(2-Cyclopropylcyclopentyl)aniline (1p)

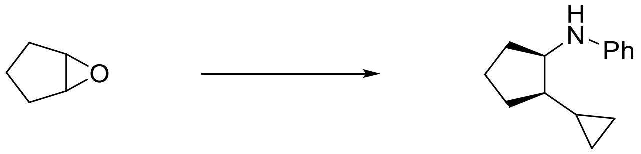

To a solution of cyclopropylmagnesium bromide (20 mL, as prepared in **General procedure A**) was added anhydrous cuprous bromide dimethyl sulfide complex (144 mg, 0.70 mmol) and the resulting solution was stirred for 15 min. Cyclopentene oxide (611 µL, 7.0 mmol) was subsequently added and the reaction was gently heated for 4 h. On cooling, the reaction was quenched by the addition of sat. aq. $NaHCO_3$ and extracted into EtOAc, followed by washing with H_2O (3x). The organic layer was separated and dried of anhydrous $MgSO_4$. Filtration and concentration *in vacuo* gave an off-colorless oil which is sufficiently pure to utilize without additional purification. To the crude product was added THF (20 mL), pyridine (2 mL) and methanesulfonyl chloride (618 µL, 8 mmol), with the drop-wise addition of triethylamine (200 µL). The reaction was stirred under argon overnight. The reaction was quenched by the addition of sat. aq. $NaHCO_3$ and extracted into EtOAc, followed by washing with H_2O (3x). The organic layer was separated and dried of anhydrous $MgSO_4$. Filtration and concentration *in vacuo* gives a crude brown oil. The product was partially purified by passing the crude oil through a silica gel plug (10:1 to 5:1, pentane/EtOAc). The enriched oil could be utilized without additional purification. To the resulting oil was added aniline (6.4 mL, 70 mmol) and K_2CO_3 (970 mg, 7.0 mmol). The suspension was heated to 70 °C and stirred for 48 h under argon. Upon cooling, the solution was

diluted with toluene (10 mL) and filtered. The solution was concentrated under high vacuum rotary evaporation until 1-2 mL of oil was remaining. Purification was achieved by flash column chromatography (2:1, petroleum ether 40-60 °C/toluene to toluene), which gave (*rac*)-(*cis*)-*N*-(2-cyclopropylcyclopentyl)aniline (462 mg, 33% over three steps) as a yellow oil; ν_{max} / cm^{-1} : 3421 (m), 1601 (m), 1504 (m), 1430, 1317 (m), 1016; ^{1}H NMR (400 MHz, $CDCl_3$): δ 7.20 – 7.11 (2H, m), 6.72 – 6.58 (3H, m), 4.19 – 3.99 (1H, br. s), 3.77 (1H, q, *J* = 6.5 Hz), 2.11 – 2.00 (1H, m), 1.84 – 1.71 (2H, m), 1.64 – 1.54 (3H, m), 1.47 – 1.37 (1H, m), 0.75 – 0.63 (1H, m), 0.51 – 0.36 (2H, m), 0.16 – 0.01 (2H, m); ^{13}C NMR (101 MHz, $CDCl_3$): δ 148.5, 129.3, 116.8, 113.2, 57.4, 47.7, 32.0, 29.6, 21.6, 10.8, 4.7, 2.8; m/z (ESI^+) HRMS: Calculated for $C_{14}H_{20}N$: 202.1590; Found $[M+H]^+$: 202.1594.

(*rac*)-(*trans*)-*N*-(2-(2-Methylcyclopropyl)ethyl)aniline (1q)

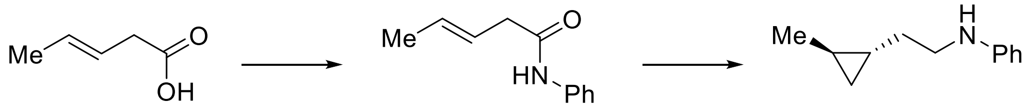

To a solution of anhydrous dichloromethane (20 mL) was added *trans*-pent-3-enoic acid (1.0 mL, 10 mmol), oxalyl chloride (1.1 mL, 12 mmol) and DMF (*ca.* 25 µL). The solution was stirred for 2 h at r.t. The resulting solution was concentrated *in vacuo* and to the residual crude mixture was added anhydrous dichloromethane (30 mL) and aniline (870 µL, 9.5 mmol). Triethylamine (2.0 mL, 14 mmol) was added drop-wise and the solution was stirred for 18 h. The resulting solution was diluted with dichloromethane (50 mL) and washed with H_2O (20 mL), 10% aq. NaOH (3 × 20 mL), sat. aq. $NaHCO_3$ (20 mL), H_2O (20 mL) and brine (10 mL). The organic layer was separated, dried over anhydrous $MgSO_4$ and subsequently filtered. The organic extracts were concentrated under reduced pressure to give *trans-N*-phenylpent-3-enamide as a pure white solid. In a separate flask was added *trans-N*-phenylpent-3-enamide (414 mg, 2.37 mmol) and anhydrous dichloromethane (10 mL). The resulting solution was cooled to 0 °C under argon. Diiodomethane (955 µL, 11.9 mmol) was added followed by the drop-wise addition of diethyl zinc in hexane (11.9 mL, 1.0 M). The reaction was stirred for 1 h followed by a further portion of diiodomethane (955 µL, 11.9 mmol) followed by the drop-wise addition of diethyl zinc in hexane (11.9 mL, 1.0 M). The resulting suspension was stirred for 2 h. The reaction was cooled to -10 °C and sat. aq. NaEDTA (10 mL) was added dropwise (Note: care should be taken when quenching excess diethyl zinc). The resulting suspension was diluted with EtOAc (60 mL) and washed with H_2O, brine and the organic layer was dried over anhydrous $MgSO_4$. After filtration, the solution was concentrated in vacuo to give the crude cyclopropane as off-colorless solid. To the crude solid was added anhydrous THF (10 mL) under argon. To this solution was added borane in THF (5 mL, 1.0 M) and the solution was heated to reflux for 2 h. After cooling to r.t., 10% aq. NaOH (10 mL) was added slowly, and the mixture was further heated to reflux for 1.5 h. After cooling to r.t., the mixture was diluted with EtOAc (50 mL) and the organic layer was washed with H_2O (2 × 20 mL) and brine (20 mL). The organic layer was separated, dried over anhydrous $MgSO_4$ and filtered. After concentrating

the crude material was purified by flash column chromatography (20:1 to 10:1, petroleum ether 40-60 °C/EtOAc), which gave (*rac*)-(*trans*)-*N*-(2-(2-methylcyclopropyl)ethyl)aniline (211 mg, 51% over four steps) as a colorless clear oil; ν_{max} / cm^{-1} : 1603 (m), 1506 (m), 1263 (m); ^{1}H NMR (400 MHz, $CDCl_3$): δ 7.22 – 7.12 (2H, m), 6.74 – 6.66 (1H, m), 6.66 – 6.59 (2H, m), 4.01 – 3.46 (1H, br. s), 3.30 – 3.12 (2H, m), 1.61 – 1.41 (2H, m), 1.04 (3H, d, *J* = 5.9 Hz), 0.54 – 0.41 (2H, m), 0.30 – 0.18 (2H, m); ^{13}C NMR (101 MHz, $CDCl_3$): δ 148.7, 129.4, 117.3, 112.9, 44.3, 34.1, 19.1, 17.7, 12.9, 12.7; m/z (ESI^+) HRMS: Calculated for $C_{12}H_{18}N$: 176.1434; Found $[M+H]^+$: 176.1442.

N-(2-((1R,2S)-2-methylcyclopropyl)ethyl)aniline (1q)

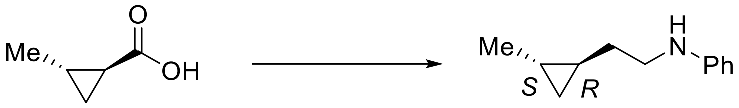

A solution of (1*S*,2*S*)-2-methylcyclopropane-1-carboxylic acid (2.5 g, 25 mmol) (prepared according to the literature procedure from (*R*)-propylene oxide)[5] in dry Et_2O (75 mL) was added dropwise to a solution of lithium aluminium hydride (949 mg, 25 mmol) at 0 °C. After stirring for 2 hours at room temperature, the solution was cooled down to 0 °C and carefully quenched with a 10% aq. NaOH (25 mL). The resulting solution was extracted with Et_2O (3 x 100 mL) and the combined organic layers were washed with H_2O (100 mL) and brine (100 mL). The organic layer was dried over anhydrous $MgSO_4$, filtered and **carefully** concentrated under reduced pressure to afford ((1*S*,2*S*)-2-methylcyclopropyl)methanol a colourless oil. Due to the volatility of this intermediate, the material was carried forward with remaining solvent (Et_2O). For information: ^{1}H NMR (500 MHz, $CDCl_3$) δ 3.46 – 3.38 (m, 2H), 1.05 (d, *J* = 5.9 Hz, 3H), 0.85 – 0.78 (m, 1H), 0.67 – 0.59 (m, 1H), 0.36 (m, 1H), 0.26 (m, 1H). Assuming quantitative conversion to ((1*S*,2*S*)-2-methylcyclopropyl)methanol, PPh_3 (8.54 g, 32.5 mmol) was added portionwise to a solution of ((1*S*,2*S*)-2-methylcyclopropyl)methanol and CBr_4 (8.98 g, 27 mmol) in dry dichloromethane (100 mL) at 0 °C over 1 h. After an additional 20 min, the mixture was diluted with hexane (100 mL). The precipitate was filtered and the filtrate was evaporated in vacuo. The precipitation procedure was repeated several times until no further precipitate was observed. The resulting crude was dissolved in dry DMSO (48 mL) and added dropwise to a mixture of NaCN (1.22 g, 25 mmol) in DMSO (48 mL). The reaction mixture was stirred at 120 °C overnight. The reaction mixture was cooled and diluted with Et_2O (250 mL) and water (100 mL). The organic layer was collected, washed with water (3 x 100 mL) and brine (3 x 100 mL), dried over $MgSO_4$, filtered and **carefully** concentrated under reduced pressure to afford 2-((1*R*,2*S*)-2-methylcyclopropyl)acetonitrile a colourless oil. Due to the volatility of this intermediate, the material was carried forward with remaining solvent (Et_2O) and residual trace of triphenylphosphine oxide. For information: ^{1}H NMR (500 MHz, $CDCl_3$) δ 2.37 (d, *J* = 6.3 Hz, 2H), 1.08 (dd, *J* = 5.8, 1.4 Hz, 3H), 0.79 – 0.70 (m, 2H), 0.49 (m, 1H), 0.44 – 0.38 (m, 1H). Assuming quantitative conversion to 2-((1*R*,2*S*)-2-methylcyclopropyl)acetonitrile, a solution of 2-((1*R*,2*S*)-2-methylcyclopropyl)acetonitrile and NaOH

(5.3 g, 133 mmol) in water (65 mL) was refluxed overnight. The resulting crude mixture was cooled down to 0 °C and carefully acidified to pH = 1 using a 4M HCl solution. The aqueous layer was extracted with Et_2O (3 x 100 mL), dried over $MgSO_4$, filtered and **carefully** concentrated under reduced pressure to afford 2-((1*R*,2*S*)-2-methylcyclopropyl)acetic acid (538 mg) as colourless oil. The product was engaged in the next step without further purification. For information: ^{1}H NMR (500 MHz, $CDCl_3$) δ 2.34 – 2.20 (m, 2H), 1.05 (d, *J* = 6.0 Hz, 3H), 0.74 (m, 1H), 0.62 – 0.53 (m, 1H), 0.32 (m, 2H). To a solution of anhydrous dichloromethane (9 mL) was added 2-((1*R*,2*S*)-2-methylcyclopropyl)acetic acid (538 mg, 4.71 mmol), oxalyl chloride (0.48 mL, 5.7 mmol) and DMF (approx. 10 µL). The solution was stirred for 2 h at r.t.. The resulting solution was concentrated *in vacuo* and to the residual crude mixture was added anhydrous dichloromethane (15 mL) and aniline (409 µL, 4.47 mmol). Triethylamine (941 µL, 6.77 mmol) was added dropwise and the solution was stirred for 18 h. The resulting solution was diluted with dichloromethane (25 mL) and washed with H_2O (10 mL), 10% aq. NaOH (3 × 10 mL), sat. aq. $NaHCO_3$ (10 mL), H_2O (10 mL) and brine (5 mL). The organic layer was separated, dried over anhydrous $MgSO_4$ and subsequently filtered. The organic extracts were concentrated under reduced pressure to give 2-((1*R*,2*S*)-2-methylcyclopropyl)-N-phenylacetamide as a white solid. To the crude solid was added anhydrous THF (5 mL) under argon. To this solution was added borane in THF (14 mL, 1.0 M) and the solution was heated to reflux overnight. After cooling to r.t., 10% aq. NaOH (30 mL) was added slowly, and the mixture was further heated to reflux for 3 h. After cooling to r.t., the mixture was diluted with EtOAc (50 mL) and the organic layer was washed with H_2O (2 × 20 mL) and brine (20 mL). The organic layer was separated, dried over anhydrous $MgSO_4$ and filtered. After concentrating, the crude material was purified by flash column chromatography (20:1 to 10:1, Hexane/EtOAc), which gave *N*-(2-((1*R*,2*S*)-2-methylcyclopropyl)ethyl)aniline (235 mg, 5.4% over 7 steps, 66% per steps, 99% *e.e*) as a colorless clear oil. The analytical data were in accordance with the racemic **1q**. **$[\alpha]^{26}_{D}$** +46 (c = 0.25, $CHCl_3$).The enantiopurity of this compound was determined by chiral SFC (Chiralpak IE, isocratic CO_2-MeCN 99.5:0.5, 2.0 mL/min, 40 °C, 140 bar) against a racemic standard; tR (major – 10.34 min and tR (minor) – 9.46 min.

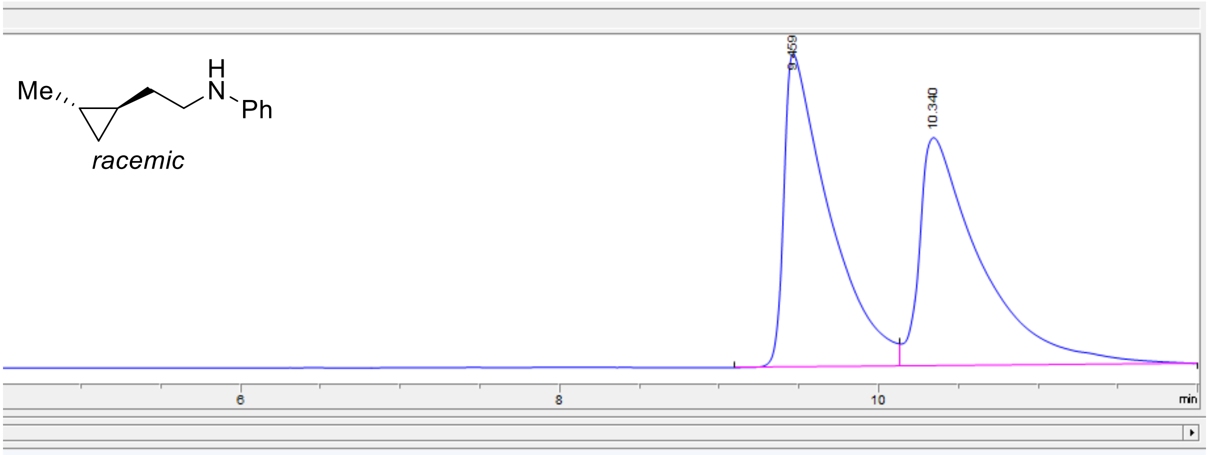

#	Time	Area	Height	Width	Area%	Symmetry
1	9.459	13172.9	663.2	0.2757	50.409	0.232
2	10.34	12959.3	482.8	0.3692	49.591	0.272

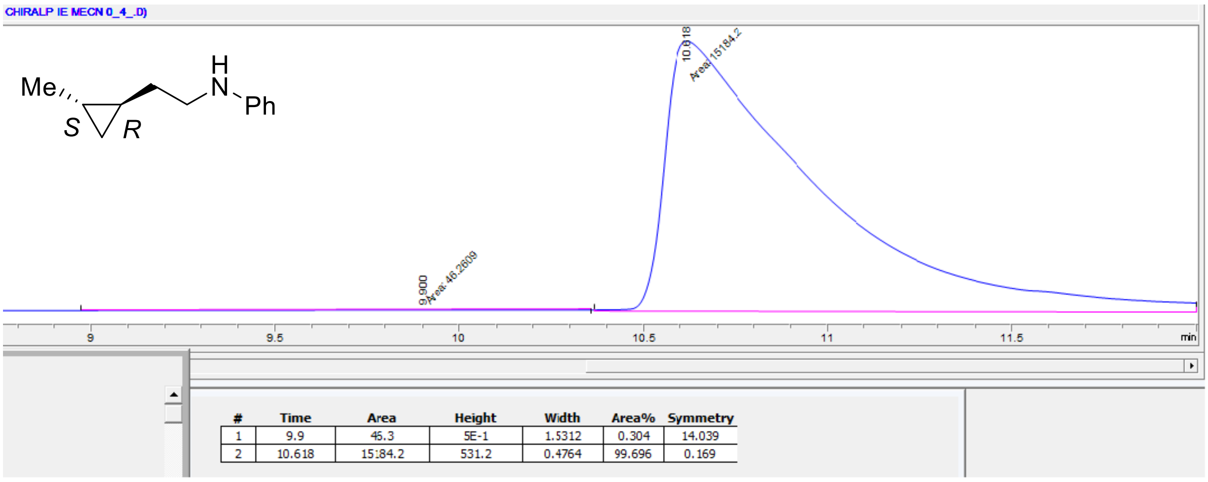

#	Time	Area	Height	Width	Area%	Symmetry
1	9.9	46.3	5E-1	1.5312	0.304	14.039
2	10.618	15184.2	531.2	0.4764	99.696	0.169

(*rac*)-(*trans*)-*N*-(2-(2-Ethylcyclopropyl)ethyl)aniline (1r)

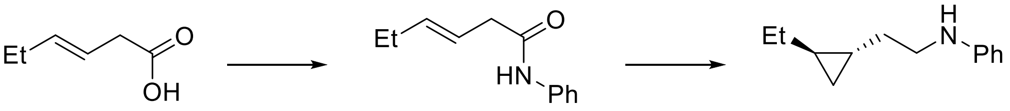

To a solution of anhydrous dichloromethane (20 mL) was added *trans*-hex-3-enoic acid (1.2 mL, 10 mmol), oxalyl chloride (1.1 mL, 12 mmol) and DMF (*ca.* 25 µL). The solution was stirred for 2 h at r.t. The resulting solution was concentrated *in vacuo* and to the residual crude mixture was added anhydrous dichloromethane (30 mL) and aniline (870 µL, 9.5 mmol). Triethylamine (2.0 mL, 14 mmol) was added drop-wise and the solution was stirred for 18 h. The resulting solution was diluted with dichloromethane (50 mL) and washed with H_2O (20 mL), 10% aq. NaOH (3 × 20 mL), sat. aq. $NaHCO_3$ (20 mL), H_2O (20 mL) and brine (10 mL). The organic layer was separated, dried over anhydrous $MgSO_4$ and subsequently filtered. The organic extracts were concentrated under reduced pressure to give *trans*-*N*-phenylhex-3-enamide as a pure white solid. In a separate flask was added *trans*-*N*-phenylhex-3-enamide

(227 mg, 1.2 mmol) and anhydrous dichloromethane (7 mL). The resulting solution was cooled to 0 °C under argon. Diiodomethane (484 µL, 6.0 mmol) was added followed by the drop-wise addition of diethyl zinc in hexane (6.0 mL, 1.0 M). The reaction was stirred for 1 h followed by a further portion of diiodomethane (484 µL, 6.0 mmol) followed by the drop-wise addition of diethyl zinc in hexane (6.0 mL, 1.0 M). The resulting suspension was stirred for 2 h. The reaction was cooled to -10 °C and sat. aq. NaEDTA (10 mL) was added dropwise (Note: care should be taken when quenching excess diethyl zinc). The resulting suspension was diluted with EtOAc (50 mL) and washed with H_2O, brine and the organic layer was dried over anhydrous $MgSO_4$. After filtration, the solution was concentrated in vacuo to give the crude cyclopropane as off-colorless solid. To the crude solid was added anhydrous THF (3 mL) under argon. To this solution was added borane in THF (3 mL, 1.0 M) and the solution was heated to reflux for 3 h. After cooling to r.t., 10% aq. NaOH (10 mL) was added slowly, and the mixture was further heated to reflux for 1.5 h. After cooling to r.t., the mixture was diluted with EtOAc (50 mL) and the organic layer was washed with H_2O (2 × 20 mL) and brine (20 mL). The organic layer was separated, dried over anhydrous $MgSO_4$ and filtered. After concentrating the crude material was purified by flash column chromatography (20:1, pentane/EtOAc), which gave (*rac*)-(*trans*)-*N*-(2-(2-ethylcyclopropyl)ethyl)aniline (178 mg, 78% over four steps) as a colorless clear oil; ν_{max} / cm^{-1} : 3414 (m), 1602 (m), 1505 (m), 1319 (m), 1178 (m); ^{1}H NMR (400 MHz, $CDCl_3$): δ 7.22 – 7.10 (2H, m), 6.69 (1H, t, *J* = 7.3 Hz), 6.61 (2H, d, *J* = 7.6 Hz), 3.80 – 3.64 (1H, br. s), 3.19 (2H, t, *J* = 7.5 Hz), 1.60 – 1.48 (2H, m), 1.36 – 1.13 (2H, m), 0.97 (3H, t, *J* = 7.5 Hz), 0.56 – 0.40 (2H, m), 0.34 – 0.21 (2H, m); ^{13}C NMR (101 MHz, $CDCl_3$): δ 148.7, 129.4, 117.3, 112.9, 44.3, 34.2, 27.3, 20.7, 16.4, 13.9, 11.5; m/z (ESI^+) HRMS: Calculated for $C_{13}H_{20}N$: 190.1590; Found $[M+H]^+$: 190.1594.

(*rac*)-(*trans*)-*N*-(2-(2-Phenylcyclopropyl)ethyl)aniline (1s)

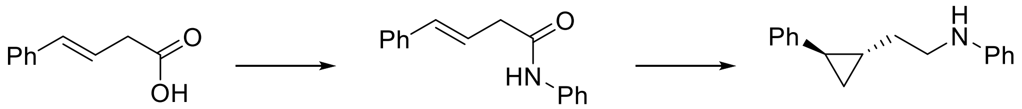

To a solution of anhydrous dichloromethane (20 mL) was added *trans*-4-phenylbut-3-enoic acid (1.0 g mL, 6.2 mmol), oxalyl chloride (0.61 mL, 7.0 mmol) and DMF (*ca.* 25 µL). The solution was stirred for 2 h at r.t. The resulting solution was concentrated *in vacuo* and to the residual crude mixture was added anhydrous dichloromethane (20 mL) and aniline (547 µL, 6.0 mmol). Triethylamine (1.0 mL, 7.0 mmol) was added drop-wise and the solution was stirred for 2 h. The resulting solution was diluted with dichloromethane (50 mL) and washed with H_2O (20 mL), 10% aq. NaOH (3 × 20 mL), sat. aq. $NaHCO_3$ (20 mL), H_2O (20 mL) and brine (10 mL). The organic layer was separated, dried over anhydrous $MgSO_4$ and subsequently filtered. The organic extracts were concentrated under reduced pressure to give *trans*-*N*,4-diphenylbut-3-enamide as a pure yellow solid in quantitative yield. To a separate flame-dried flask was added anhydrous dichloromethane (10 mL) and diethyl zinc in hexane (10 mL, 1.0 M) and the resulting solution was cooled to 0 °C. Trifluoroacetic acid (771 µL, 10.0 mmol)

in dichloromethane (10 mL) was added drop-wise over 5 min and the solution stirred for 15 min resulting in a viscous suspension. Diiodomethane (807 µL, 10.0 mmol) was added and the solution was stirred for 15 min leading to a colorless clear solution. *trans*-*N*,4-Diphenylbut-3-enamide (1.42 g, 6.0 mmol) was added and the solution was warmed to r.t. over 1 h. The reaction was cooled to 0 °C and sat. aq. NaEDTA (10 mL) was added dropwise (Note: care should be taken when quenching excess diethyl zinc). The resulting suspension was diluted with EtOAc (70 mL) and washed with H_2O, brine and the organic layer was dried over anhydrous $MgSO_4$. After filtration, the solution was concentrated in vacuo to give the crude product. To the crude product was added anhydrous THF (15 mL) under argon. To this solution was added borane in THF (10 mL, 1.0 M) and the solution was heated to reflux for 3 h. After cooling to r.t., 10% aq. NaOH (15 mL) was added slowly, and the mixture was further heated to reflux for 1 h. After cooling to r.t., the mixture was diluted with EtOAc (70 mL) and the organic layer was washed with H_2O (2 × 20 mL) and brine (20 mL). The organic layer was separated, dried over anhydrous $MgSO_4$ and filtered. After concentrating the crude material was purified by flash column chromatography (20:1 to 10:1, petroleum ether 40-60 °C/EtOAc), which gave (*rac*)-(*trans*)-*N*-(2-(2-phenylcyclopropyl)ethyl)aniline (614 mg, 43% over four steps) as a colorless clear oil; ν_{max} / cm^{-1} : 3411 (m), 1601 (m), 1504 (m), 1319 (m); ^{1}H NMR (400 MHz, $CDCl_3$): δ 7.30 – 7.03 (7H, m), 6.70 (1H, t, *J* = 7.5 Hz), 6.65 – 6.58 (2H, m), 3.98 – 3.52 (1H, br. s), 3.38 – 3.17 (2H, m), 1.79 – 1.71 (2H, m), 1.20 – 1.04 (1H, m), 1.04 – 0.92 (1H, m), 0.89 – 0.75 (1H, m); ^{13}C NMR (101 MHz, $CDCl_3$): δ 148.4, 143.4, 129.4, 128.5, 125.7, 125.6, 117.4, 113.0, 44.0, 34.2, 23.2, 21.7, 15.9; m/z (ESI^+) HRMS: Calculated for $C_{17}H_{20}N$: 238.1590; Found $[M+H]^+$: 238.1597.

***N*-(2-(2,2-Dimethylcyclopropyl)ethyl)aniline (1t)**

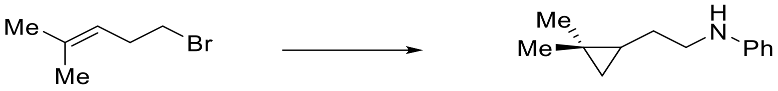

To a separate flame-dried flask was added anhydrous dichloromethane (10 mL) and diethyl zinc in hexane (10 mL, 1.0 M) and the resulting solution was cooled to 0 °C. Trifluoroacetic acid (771 µL, 10.0 mmol) in dichloromethane (10 mL) was added drop-wise over 5 min and the solution stirred for 15 min resulting in a viscous suspension. Diiodomethane (807 µL, 10.0 mmol) was added and the solution was stirred for 15 min leading to a colorless clear solution. 5-Bromo-2-methyl-2-pentene (540 µL, 4.0 mmol) was added and the solution was warmed to r.t. over 1 h. The reaction was cooled to 0 °C and sat. aq. NaEDTA (10 mL) was added dropwise (Note: care should be taken when quenching excess diethyl zinc). The resulting suspension was diluted with EtOAc (70 mL) and washed with H_2O, brine and the organic layer was dried over anhydrous $MgSO_4$. After filtration, the solution was concentrated *in vacuo* to give cyclopropane product. To the product was added DMF (10 mL), aniline (913 µL, 10.0 mmol) and K_2CO_3 (552 mg, 4.0 mmol). The suspension was heated to 60 °C for 18 h. After cooling to r.t., the mixture was diluted with EtOAc (4 mL) and the organic layer was washed with H_2O (3 × 20 mL) and

brine (20 mL). The organic layer was separated, dried over anhydrous $MgSO_4$ and filtered. After concentrating the crude material was purified by flash column chromatography (40:1 to 20:1, hexane/EtOAc), which gave *N*-(2-(2,2-dimethylcyclopropyl)ethyl) (502 mg, 66% over two steps) as a pale yellow oil; ν_{max} / cm^{-1} : 3414 (m), 1602 (m), 1504 (m), 1319 (m); ^1H NMR (400 MHz, $CDCl_3$): δ 7.20 – 7.12 (2H, m), 6.76 – 6.58 (3H, m), 3.78 – 3.60 (1H, br. s), 3.18 (2H, t, *J* = 7.0 Hz), 1.80 – 1.64 (1H, m), 1.64 – 1.45 (2H, m), 1.07 (3H, s), 1.05 (3H, s), 0.62 – 0.50 (1H, m), 0.45 (1H, dd, *J* = 8.5, 4.0 Hz), 0.07 – -0.14 (1H, m); ^{13}C NMR (101 MHz, $CDCl_3$): δ 148.7, 129.4, 117.3, 112.9, 44.6, 29.8, 27.6, 22.5, 20.3, 19.8, 15.4; m/z (ESI^+) HRMS: Calculated for $C_{13}H_{20}N$: 190.1590; Found $[M+H]^+$: 190.1598.

(*rac*)-*N*-((Bicyclo[4.1.0]heptan-2-yl)methyl)aniline (1u)

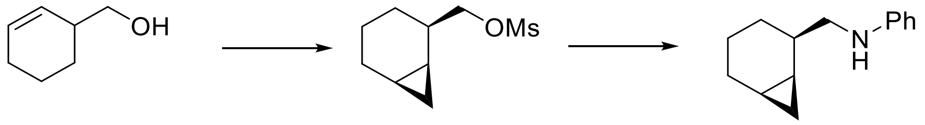

To a flame-dried flask was added anhydrous dichloromethane (10 mL) and diethyl zinc in hexane (10 mL, 1.0 M) and the resulting solution was cooled to 0 ºC. Trifluoroacetic acid (771 μL, 10.0 mmol) in dichloromethane (10 mL) was added drop-wise over 5 min and the solution stirred for 15 min resulting in a viscous suspension. Diiodomethane (807 μL, 10.0 mmol) was added and the solution was stirred for 15 min leading to a colorless clear solution. (Cyclohex-2-en-1-yl)methanol (560 mg, 5.0 mmol) was added and the solution was warmed to r.t. over 1 h. The reaction was cooled to 0 ºC and sat. aq. NaEDTA (10 mL) was added dropwise (Note: care should be taken when quenching excess diethyl zinc). The resulting suspension was diluted with EtOAc (70 mL) and washed with H_2O, brine and the organic layer was dried over anhydrous $MgSO_4$. After filtration, the solution was concentrated in vacuo to give the crude cyclopropane as off-colorless oil. To the crude oil was added anhydrous dichloromethane (25 mL) and methanesulfonyl chloride (774 μL, 10.0 mmol). Triethylamine (1.40 mL, 10.0 mmol) was added drop-wise and the solution was stirred for 2 h. The resulting suspension was diluted with EtOAc (100 mL) and washed with H_2O, brine and the organic layer was dried over anhydrous $MgSO_4$. After filtration, the solution was concentrated in vacuo to give the mesylated product. To the mesylated product was added aniline (7 mL) and K_2CO_3 (690 mg, 5.0 mmol). The resulting solution was heated to 100 ºC for 18 h with vigorous stirring. On cooling, the solution was diluted with toluene (10 mL) and filtered. The excess aniline was removed under high vacuum rotary evaporation. Purification was achieved by flash column chromatography (1:1, petroleum ether 40-60 ºC/toluene to toluene), which gave (*rac*)-*N*-((bicyclo[4.1.0]heptan-2-yl)methyl)aniline (200 mg, 20% over three steps) as a yellow oil; ν_{max} / cm^{-1} : 3418 (m), 1601 (m), 1504 (m), 1319 (m), 745 (m); ^1H NMR (400 MHz, $CDCl_3$): δ 7.24 – 7.08 (2H, m), 6.78 – 6.51 (3H, m), 3.98 – 3.64 (1H, br. s), 3.12 – 2.99 (2H, m), 2.25 – 2.12 (1H, m), 2.04 – 1.89 (1H, m,), 1.64 – 1.47 (1H, m), 1.45 – 1.31 (2H, m, 1H), 1.27 – 1.07 (1H, m), 1.04 – 0.92 (2H, m), 0.71 – 0.68 (1H, m), 0.58 – 0.45 (1H, m), 0.04 (1H, q, *J* = 5.0 Hz); ^{13}C NMR (101 MHz,

$CDCl_3$): δ 148.8, 129.4, 117.1, 113.0, 50.2, 32.9, 24.8, 24.1, 22.1, 13.7, 9.7, 7.7; m/z (ESI^+) HRMS: Calculated for $C_{14}H_{20}N$: 202.1590; Found $[M+H]^+$: 202.1597.

***N*-(2-Cyclopropylbenzyl)-1,1-diphenylmethanamine (1v)**

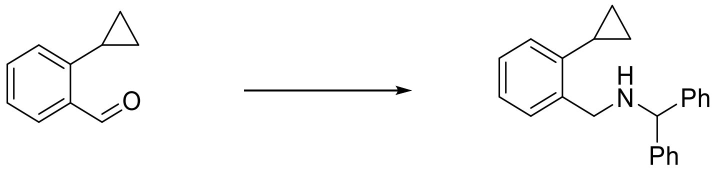

To a flame-dried flask containing 3 Å molecular sieve pellets (1.0 g) was added anhydrous THF (6.0 mL), benzhydrylamine (690 µL, 4.0 mmol) and 2-cyclopropylbenzaldehyde (428 mg, 2.93 mmol). The resulting solution was stirred at room temperature for 4.5 h, after which the solution was filtered and concentrated *in vacuo*. To the resulting crude oil was added MeOH (8 mL). $NaBH_4$ (304 mg, 8.0 mmol) was added portion-wise to with vigorous stirring over 15 min. After 2 h, 10% aq. NaOH (25 mL) was added to the solution followed by stirring for 15 min. The organic products were extracted into EtOAc (3 × 25 mL). The organic extracts were dried over anhydrous $MgSO_4$, filtered and concentrated *in vacuo*. Purification was achieved by flash column chromatography (3:1, hexane/toluene to toluene), which gave *N*-(2-cyclopropylbenzyl)-1,1-diphenylmethanamine (428 mg, 53%) as a colorless clear oil; ν_{max} / cm^{-1} : 1601, 1491 (m), 1451 (m); ^1H NMR (400 MHz, $CDCl_3$): δ 7.51 – 7.40 (4H, m), 7.35 – 7.27 (5H, m), 7.24 – 7.14 (4H, m), 7.05 – 6.94 (1H, m), 4.91 (1H, s), 3.92 (2H, s), 2.05 – 1.90 (1H, m), 0.93 – 0.73 (2H, m), 0.69 – 0.55 (2H, m); ^{13}C NMR (101 MHz, $CDCl_3$): δ 144.2, 141.5, 139.6, 128.9, 128.6, 127.5, 127.3, 127.2, 125.8, 125.5, 67.2, 49.9, 12.7, 7.4; m/z (ESI^+) HRMS: Calculated for $C_{23}H_{24}N$: 314.1903; Found $[M+H]^+$: 314.1913.

***N*-(2-Cyclopropyl-5-fluorobenzyl)-1,1-diphenylmethanamine (1w)**

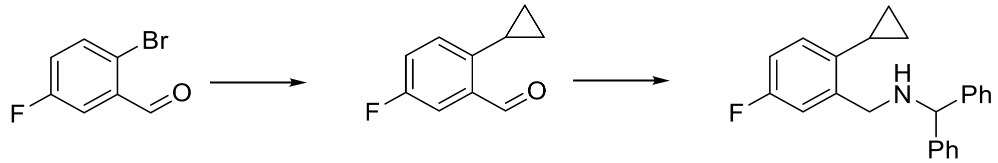

To a flame-dried reaction flask was added cyclopropylboronic acid (506 mg, 5.88 mmol), palladium-tetrakis(triphenylphosphine) (102 mg, 88.3 µmol), K_2CO_3 (0.81 g, 5.88 mmol), anhydrous toluene (15 mL) and the resulting solution was vigorously stirred. To the stirring solution was added 2-bromo-5-fluorobenzaldehyde (597 µL, 2.94 mmol) and the subsequent solution was sparged with argon for 15 min. After sparging, the solution was heated to reflux overnight. On cooling the solution was filtered through celite and diluted with toluene (50 mL). The resulting toluene solution was washed with 10% aq. NaOH (25 mL), H_2O (3 × 25 mL), brine (10 mL). The organic extracts were dried over anhydrous $MgSO_4$, filtered and concentrated *in vacuo* to give an off-colorless oil (crude 2-cyclopropyl-5-fluorobenzaldehyde), which was utilized without additional purification. To a flame-dried flask containing 3 Å molecular sieve pellets (1.0 g) was added anhydrous toluene (6.0 mL), benzhydrylamine

(517 µL, 3.0 mmol) and crude 2-cyclopropyl-5-fluorobenzaldehyde. The resulting solution was stirred at room temperature for 4.5 h, after which the solution was filtered and concentrated *in vacuo*. To the resulting crude oil was added MeOH (4 mL). $NaBH_4$ (281 mg, 7.4 mmol) was added portion-wise to with vigorous stirring over 15 min. After 2 h, 10% aq. NaOH (25 mL) was added and the solution was subsequently stirred for 15 min. The organic products were extracted into EtOAc (3 × 25 mL). The organic extracts were dried over anhydrous $MgSO_4$, filtered and concentrated *in vacuo*. Purification was achieved by flash column chromatography (20:1 to 5:1, pentane/EtOAc to toluene), which gave *N*-(2-Cyclopropyl-5-fluorobenzyl)-1,1-diphenylmethanamine (0.63 g, 65%) as a pale yellow oil; ν_{max} / cm^{-1}: 1608, 1588 (m), 1492 (m), 1452 (m), 1027 (m); ^1H NMR (400 MHz, $CDCl_3$): δ 7.47 – 7.42 (4H, m), 7.34 – 7.28 (4H, m), 7.25 – 7.20 (2H, m), 7.13 (1H, dd, *J* = 10.0, 3.0 Hz), 6.96 (1H, dd, *J* = 8.5, 6.0 Hz), 6.85 (1H, td, *J* = 8.5, 3.0 Hz), 4.91 (1H, s), 3.90 (2H, s), 1.93 – 1.81 (1H, m), 0.90 – 0.77 (2H, m), 0.61 – 0.48 (2H, m); ^{13}C NMR (101 MHz, $CDCl_3$): δ 161.4 (d, *J* = 244.4 Hz), 144.1, 142.2 (d, *J* = 6.6 Hz), 136.7 (d, *J* = 3.1 Hz), 128.7, 127.6 (d, *J* = 8.0 Hz), 127.5, 127.3, 115.2 (d, *J* = 18.7 Hz), 113.4 (d, *J* = 21.0 Hz), 49.2, 12.3, 7.0; ^{19}F NMR (377 MHz, $CDCl_3$): δ -117.5; m/z (ESI^+) HRMS: Calculated for $C_{23}H_{23}FN$: 332.1809; Found $[M+H]^+$: 332.1817.

***N*-(2-Cyclopropyl-5-methoxybenzyl)-1,1-diphenylmethanamine (1x)**

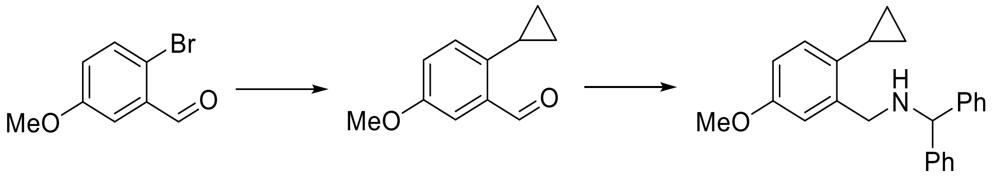

To a flame-dried reaction flask was added cyclopropylboronic acid (506 mg, 5.88 mmol), palladium-tetrakis(triphenylphosphine) (102 mg, 88.3 µmol), K_2CO_3 (0.81 g, 5.88 mmol), anhydrous toluene (15 mL) and the resulting solution was vigorously stirred. To the stirring solution was added 2-bromo-5-methoxybenzaldehyde (632 µL, 2.94 mmol) and the subsequent solution was sparged with argon for 15 min. After sparging, the solution was heated to reflux overnight. On cooling the solution was filtered through celite and diluted with toluene (50 mL). The resulting toluene solution was washed with 10% aq. NaOH (25 mL), H_2O (3 × 25 mL), brine (10 mL). The organic extracts were dried over anhydrous $MgSO_4$, filtered and concentrated *in vacuo* to give an off-colorless oil (crude 2-cyclopropyl-5-methoxybenzaldehyde), which was utilized without additional purification. To a flame-dried flask containing 3 Å molecular sieve pellets (1.0 g) was added anhydrous toluene (6.0 mL), benzhydrylamine (517 µL, 3.0 mmol) and crude 2-cyclopropyl-5-methoxybenzaldehyde. The resulting solution was stirred at room temperature for 4.5 h, after which the solution was filtered and concentrated *in vacuo*. To the resulting crude oil was added MeOH (4 mL). $NaBH_4$ (281 mg, 7.4 mmol) was added portion-wise to with vigorous stirring over 15 min. After 2 h, NaOH 10% (25 mL) was added and the solution was subsequently stirred for 15 min. The organic products were extracted into EtOAc (3 × 25 mL). The organic extracts were dried over anhydrous $MgSO_4$, filtered and concentrated *in vacuo*. Purification

was achieved by flash column chromatography (20:1 to 5:1, pentane/EtOAc to toluene), which gave *N*-(2-cyclopropyl-5-methoxybenzyl)-1,1-diphenylmethanamine (1.02 g, >99%) as a colorless clear oil; ν_{max} / cm^{-1} : 1604 (m), 1497 (m), 1452 (m), 1029 (m); ^1H NMR (400 MHz, $CDCl_3$): δ 7.49 – 7.42 (4H, m), 7.34 – 7.27 (4H, m), 7.25 – 7.18 (2H, m), 6.94 (2H, d, *J* = 11.5 Hz), 6.72 (1H, dd, *J* = 8.5, 3.0 Hz), 4.91 (1H, s), 3.90 (2H, s), 3.79 (3H, s), 1.84 (1H, td, *J* = 8.5, 4.5 Hz), 0.84 – 0.73 (2H, m), 0.59 – 0.50 (2H, m); ^{13}C NMR (101 MHz, $CDCl_3$): δ 157.9, 144.2, 141.2, 133.4, 128.6, 127.5, 127.3, 127.2, 114.7, 111.9, 67.1, 55.4, 49.7, 12.2, 6.9; m/z (ESI^+) HRMS: Calculated for $C_{24}H_{26}NO$: 344.2009; Found $[M+H]^+$: 344.2014.

Benzylcyclopropane[6]

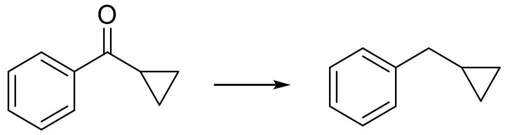

To a solution of ethylene glycol (25 mL) was added cyclopropyl phenyl ketone (4.73 mL, 34.2 mmol), hydrazine hydrate (3.32 mL, 68.4 mmol), potassium hydroxide (3.84 g, 68.4 mmol) and the resulting mixture was heated to 120 °C overnight. On cooling, the solution was diluted with hexane (250 mL) and H_2O (100 mL). The organic layer was washed with H_2O (3 × 50 mL) and the resulting organic layer was concentrated *in vacuo*. The resulting oil was purified by flash column chromatography (30:1 hexane/EtOAc), which gave benzylcyclopropane (3.30 g, 73%) as a colorless clear oil; ^1H NMR (300 MHz, $CDCl_3$): δ 7.36 – 7.07 (5H, m), 2.56 (2H, d, *J* = 7.0 Hz), 1.12 – 0.90 (1H, m), 0.60 – 0.43 (2H, m), 0.28 – 0.13 (2H, m); ^{13}C NMR (76 MHz, $CDCl_3$): δ 142.8, 128.6, 128.4, 125.72, 36.9, 36.2, 10.9, 4.7. All physical and spectroscopic properties are consistent with those previously reported.[7]

2-(Cyclopropylmethyl)aniline[8] **(1y)**

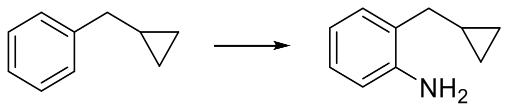

Benzyl cyclopropane (651 mg, 4.93 mmol) and acetic anhydride (2 mL) were combined and cooled to -42 °C. In a separate vessel, nitric acid 70% (183 µL, 5.92 mmol) was added to acetic anhydride (2 mL) and was subsequently stirred for 5 min at low temperature (-42 °C). Both solutions were combined and stirred for 5 min. Concentrated H_2SO_4 (25 µL) was added and the solution was stirred for 1 h, warming to r.t. over 30 mins. H_2O (3 mL) was added followed by 10% aq. NaOH (1 mL). The solution was diluted with toluene (100 mL) and subsequently washed with 10% aq. NaOH (1 × 10mL), H_2O (3 × 20 mL) and brine (1 × 20 mL). The organic layer was dried over anhydrous $MgSO_4$, filtered and concentrated *in vacuo* to give inseparable *o*/*p*-nitrated products in quantitative yield. The resulting mixture was added to a stirring solution of EtOH (3 mL). Raney nickel (*ca*. 266 mg, 3.11 mmol) was added to the solution followed by the drop-wise addition of hydrazine hydrate (294 µL, 9.43 mmol). The suspension was stirred vigorously for 3 h, after which the solution was decanted (washing with

EtOH) and concentrated *in vacuo*. The resulting oil was purified by flash column chromatography (1:1 hexane/toluene), which gave 2-(cyclopropylmethyl)aniline (234 mg, 32% over two steps) as a pale yellow oil; ^{1}H NMR (300 MHz, $CDCl_3$): δ 7.22 (1H, d, *J* = 7.5 Hz), 7.06 – 7.03 (1H, m), 6.76 (1H, dd, *J* = 7.5, 1.0 Hz), 6.69 (1H, dd, *J* = 7.5, 1.0 Hz), 3.77 – 3.52 (2H, br. s), 2.46 (2H, d, *J* =6.5 Hz), 1.10 – 0.95 (1H, m), 0.61 – 0.54 (2H, m), 0.22 – 0.17 (2H, m); ^{13}C NMR (76 MHz, $CDCl_3$): δ 144.3, 129.4, 127.1, 126.2, 118.8, 115.5, 35.5, 9.5, 4.9. All physical and spectroscopic properties are consistent with those previously reported.[7]

2-(Cyclopropylmethyl)-5-methoxyaniline (1z)

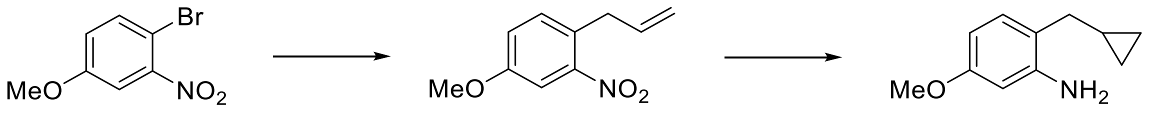

To a flame-dried reaction flask was added 1-bromo-4-methoxy-2-nitrobenzene (928 mg, 4.0 mmol), triphenylphosphine (105 mg, 0.4 mmol), tris(dibenzylideneacetone)dipalladium(0) (92 mg, 0.10 mmol), lithium chloride (470 mg, 11.0 mmol), allyltributylstannane (1.4 mL, 4.4 mmol) and anhydrous toluene (30 mL) under argon. The resulting solution was sparged with argon for 15 min. After sparging, the solution was heated to 110 °C and vigorously stirred overnight. After cooling, H_2O (20 mL) and 10% aq. NaOH (20 mL) were added sequentially, and the resulting solution was stirred for 15 min. The solution was partitioned in between toluene (50 mL) and the organic layer was extracted with toluene (3 × 30 mL). The organic layer was dried over anhydrous $MgSO_4$, filtered and concentrated under reduced pressure. The crude residue was filtered through a short silica plug (20:1 to 15:1, petroleum ether 40-60 °C /EtOAc) giving the allyl compound as a yellow oil in quantitative yield, which was utilized without additional purification. To a separate flame-dried flask was added anhydrous dichloromethane (20 mL) and diethyl zinc in hexane (20 mL, 1.0 M) and the resulting solution was cooled to 0 °C. Trifluoroacetic acid (1.54 mL, 20.0 mmol) in dichloromethane (10 mL) was added drop-wise over 5 min and the solution stirred for 15 min resulting in a viscous suspension. Diiodomethane (1.6 mL, 20.0 mmol) was added and the solution was stirred for 15 min leading to a colorless clear solution. 1-allyl-4-methoxy-2-nitrobenzene (772 mg, 4.0 mmol) was added and the solution was warmed to r.t. over 1 h. The reaction was cooled to 0 °C and sat. aq. NaEDTA (20 mL) was added dropwise (Note: care should be taken when quenching excess diethyl zinc). The resulting suspension was diluted with EtOAc (100mL) and washed with H_2O, brine and the organic layer was dried over anhydrous $MgSO_4$. After filtration, the solution was concentrated *in vacuo* to give 1-(cyclopropylmethyl)-4-methoxy-2-nitrobenzene. To a flask containing 1-(cyclopropylmethyl)-4-methoxy-2-nitrobenzene was added Fe powder (0.56 g, 10 mmol) and EtOH (5 mL). Acetic acid (4 mL) was added and the suspension was heated to 70 °C overnight. On cooling, the solution was concentrated under reduced pressure. The resulting residue was taken in EtOAc, and the organic layer was washed with 10% aq. NaOH (3 × 25 mL), H_2O (10 ml) and brine (10 mL). The organic extracts were and dried

over anhydrous $MgSO_4$. After filtration the organic layer was concentrated *in vacuo* purified by flash column chromatography (15:1 to 2:1, hexane/EtOAc), which gave 2-(cyclopropylmethyl)-5-methoxyaniline (235 mg, 33% over three steps) as an orange solid; m.p.: 45 – 47 °C (EtOAc/Hex); ν_{max} / cm^{-1} : 3466 (m), 3378 (m), 1614 (m), 1583 (m), 1508 (m), 1208 (m); ^1H NMR (400 MHz, $CDCl_3$): δ 7.09 (1H, d, *J* = 8.5 Hz), 6.37 – 6.19 (2H, m), 3.88 – 3.70 (2H, br. s) 3.76 (3H, s), 2.40 (2H, d, *J* = 6.5 Hz), 1.06 – 0.89 (1H, m), 0.65 – 0.39 (2H, m), 0.28 – 0.01 (m, 2H); ^{13}C NMR (101 MHz, $CDCl_3$): δ 159.2, 145.4, 130.2, 118.8, 103.9, 101.5, 55.3, 34.8, 9.9, 4.9; m/z (ESI^+) HRMS: Calculated for $C_{11}H_{16}NO$: 178.1226; Found $[M+H]^+$: 178.1233.

2-(Cyclopropylmethyl)-5-(trifluoromethyl)aniline (1aa)

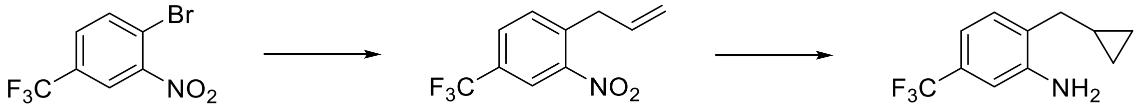

To a flame-dried reaction flask was added 1-bromo-2-nitro-4-(trifluoromethyl)benzene (613 µL, 4.0 mmol), triphenylphosphine (105 mg, 0.4 mmol), tris(dibenzylideneacetone)dipalladium(0) (92 mg, 0.10 mmol), lithium chloride (470 mg, 11.0 mmol), allyltributylstannane (1.4 mL, 4.4 mmol) and anhydrous toluene (30 mL) under argon. The resulting solution was sparged with argon for 15 min. After sparging, the solution was heated to 110 °C and vigorously stirred overnight. After cooling, H_2O (20 mL) and 10% aq. NaOH (20 mL) were added sequentially, and the resulting solution was stirred for 15 min. The solution was partitioned in between toluene (50 mL) and the organic layer was extracted with toluene (3 × 30 mL). The organic layer was dried over anhydrous $MgSO_4$, filtered and concentrated under reduced pressure. The crude residue was filtered through a short silica plug (20:1 to 15:1, petroleum ether 40-60 °C /EtOAc) giving the allyl compound as a yellow oil in quantitative yield, which was utilized without additional purification. To a separate flame-dried flask was added anhydrous dichloromethane (20 mL) and diethyl zinc in hexane (20 mL, 1.0 M) and the resulting solution was cooled to 0 °C. Trifluoroacetic acid (1.54 mL, 20.0 mmol) in dichloromethane (10 mL) was added drop-wise over 5 min and the solution stirred for 15 min resulting in a viscous suspension. Diiodomethane (1.6 mL, 20.0 mmol) was added and the solution was stirred for 15 min leading to a colorless clear solution. 1-allyl-2-nitro-4-(trifluoromethyl)benzene (924 mg, 4.0 mmol) was added and the solution was warmed to r.t. over 3 h. The reaction was cooled to 0 °C and sat. aq. NaEDTA (20 mL) was added dropwise (Note: care should be taken when quenching excess diethyl zinc). The resulting suspension was diluted with EtOAc (100mL) and washed with H_2O, brine and the organic layer was dried over anhydrous $MgSO_4$. After filtration, the solution was concentrated *in vacuo* to give 1-(cyclopropylmethyl)-2-nitro-4-(trifluoromethyl)benzene. To a flask containing 1-(cyclopropylmethyl)-2-nitro-4-(trifluoromethyl)benzene was added Fe powder (0.56 g, 10 mmol) and EtOH (5 mL). Acetic acid (4 mL) was added and the suspension was heated to 70 °C overnight. On cooling, the solution was concentrated under reduced pressure. The resulting residue was taken in EtOAc, and the organic layer

was washed with 10% aq. NaOH (3 × 25 mL), H_2O (10 ml) and brine (10 mL). The organic extracts were and dried over anhydrous $MgSO_4$. After filtration the organic layer was concentrated *in vacuo* purified by flash column chromatography (15:1 to 2:1, hexane/EtOAc), which gave 2-(cyclopropylmethyl)-5-(trifluoromethyl)aniline (118 mg, 14% ove three steps) as a yellow oil; ν_{max} / cm^{-1} 3398 (m), 3080 (m), 1626 (m), 1585 (m), 1434 (m), 1330 (m); ^1H NMR (400 MHz, $CDCl_3$): δ 7.31 (1H, d, *J* = 8.0 Hz), 6.99 (1H, d, *J* = 8.0 Hz), 6.89 (1H, s), 3.93 – 3.70 (2H, br. s), 2.46 (2H, d, *J* = 6.5 Hz), 1.07 – 0.96 (1H, m), 0.66 – 0.49 (2H, m), 0.28 – 0.14 (2H, m); ^{13}C NMR (101 MHz, $CDCl_3$): δ 144.6, 129.7, 129.6, 129.5 (q, *J* = 32.1 Hz), 124.5 (q, *J* = 271.9 Hz), 115.2 (q, *J* = 3.9 Hz), 111.6 (q, *J* = 3.9 Hz), 35.4, 9.2, 5.0; ^{19}F NMR (377 MHz, $CDCl_3$): δ -62.5. m/z (ESI^+) HRMS: Calculated for $C_{11}H_{13}F_3N$: 216.0995; Found $[M+H]^+$: 216.1000.

1-Benzyl-1,3-dihydro-2*H*-benzo[*b*]azepin-2-one (2a)

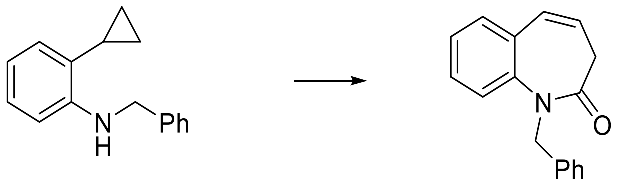

General procedure C: *N*-Benzyl-2-cyclopropylaniline (22.3 mg, 0.10 mmol) was employed. The crude mixture was purified by flash column chromatography (10:1 to 5:1, hexane/EtOAc), to yield 1-benzyl-1,3-dihydro-2*H*-benzo[*b*]azepin-2-one (17.9 mg, 72%) as a colorless clear oil; ν_{max} / cm^{-1} : 1663 (s), 1598 (m), 1448 (m), 1367 (m); ^1H NMR (400 MHz, $CDCl_3$): δ 7.37 – 7.06 (9H, m), 6.75 (1H, d, *J* = 9.5 Hz), 6.15 (1H, dt, *J* = 9.5, 7.0 Hz), 5.10 (2H, s), 3.30 – 2.72 (2H, br. s); ^{13}C NMR (101 MHz, $CDCl_3$): δ 170.6, 141.0, 137.8, 131.9, 130.2, 129.4, 128.5, 128.1, 127.2, 127.1, 127.0, 124.7, 123.1, 52.5, 36.0; m/z (ESI^+) HRMS: Calculated for $C_{17}H_{16}NO$: 250.1226; Found $[M+H]^+$: 250.1229.

1-Phenyl-1,3-dihydro-2*H*-benzo[*b*]azepin-2-one (2b)

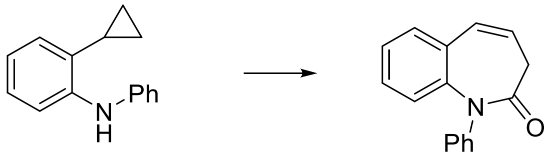

General procedure C: 2-Cyclopropyl-*N*-phenylaniline (20.9 mg, 0.10 mmol) was employed. The crude mixture was purified by flash column chromatography (10:1 to 5:1, hexane/EtOAc), to yield 1-phenyl-1,3-dihydro-2*H*-benzo[*b*]azepin-2-one (18.8 mg, 80%) as a colorless solid; m.p.: 155 – 156 °C (EtOAc/Hex); ν_{max} / cm^{-1} : 1675 (s), 1486 (m), 1334 (m), 1111 (m); ^1H NMR (400 MHz, $CDCl_3$): δ 7.40 – 7.25 (4H, m), 7.19 – 7.09 (4H, m), 6.94 – 6.85 (2H, m), 6.20 (1H, dt, *J* = 9.5, 7.0 Hz), 3.08 (2H, d, *J* = 7.0 Hz); ^{13}C NMR (101 MHz, $CDCl_3$): δ 170.0, 142.9, 141.5, 131.7, 130.2, 129.3, 129.2, 128.4, 127.9, 127.2, 127.1, 126.1, 124.6, 36.4; m/z (ESI^+) HRMS: Calculated for $C_{16}H_{14}NO$: 236.1070; Found $[M+H]^+$: 236.1062.

1-(4-Methoxyphenyl)-1,3-dihydro-2*H*-benzo[*b*]azepin-2-one (2c)

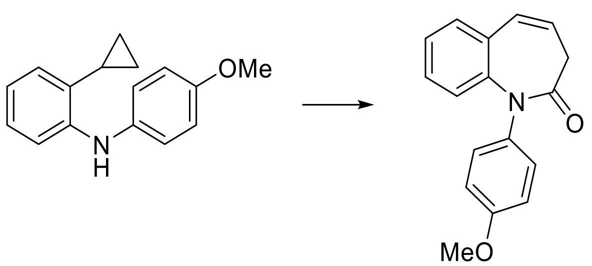

General procedure C: 2-Cyclopropyl-*N*-(4-methoxyphenyl)aniline (23.9 mg, 0.10 mmol) was employed. The crude mixture was purified by flash column chromatography (10:1 to 5:1, hexane/EtOAc), to yield 1-(4-methoxyphenyl)-1,3-dihydro-2*H*-benzo[*b*]azepin-2-one (17.5 mg, 66%) as a pale yellow solid; m.p.: 101 – 102 °C (EtOAc/Hex); ν_{max} / cm^{-1} : 1668 (s), 1507 (s), 1247 (s); ^1H NMR (400 MHz, $CDCl_3$): δ 7.33 – 7.28 (1H, m), 7.19 – 7.08 (2H, m), 7.08 – 7.01 (2H, m), 6.96 – 6.82 (4H, m), 6.19 (1H, dt, *J* = 9.5, 7.0 Hz), 3.80 (3H, s), 3.07 (2H, d, *J* = 7.0 Hz); ^{13}C NMR (101 MHz, $CDCl_3$): δ 170.1, 158.4, 141.8, 135.7, 131.4, 130.2, 129.4, 129.2, 127.8, 127.2, 125.9, 124.4, 114.5, 55.6, 36.3; m/z (ESI^+) HRMS: Calculated for $C_{17}H_{16}NO_2$: 266.1176; Found $[M+H]^+$: 266.1173.

1,3-Dihydro-2*H*-benzo[*b*]azepin-2-one (2d)

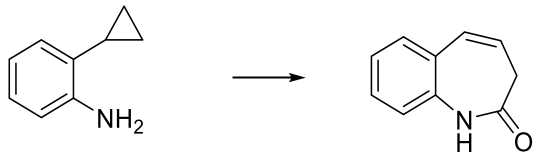

General procedure C: 2-Cyclopropylaniline (13.3 mg, 0.10 mmol) was employed. The crude mixture was purified by flash column chromatography (5:1 to 1:1, pentane/EtOAc), to yield 1,3-dihydro-2*H*-benzo[*b*]azepin-2-one (17.6 mg, 79%) as a yellow solid; m.p.: 137 – 138 °C (EtOAc/Hex); ν_{max} / cm^{-1} : 3192 (m), 1684 (s), 1639 (s), 1358 (s), 1240 (m), 1163 (m); ^1H NMR (400 MHz, $CDCl_3$): δ 9.01 – 8.73 (1H, br. s), 7.34 – 7.25 (2H, m), 7.17 – 7.08, 6.76 (1H, d, *J* = 10.0 Hz), 6.04 – 5.96 (1H, m), 2.94 (2H, d, *J* = 7.0 Hz); ^{13}C NMR (101 MHz, $CDCl_3$): δ 172.6, 135.9, 130.8, 130.2, 129.0, 128.5, 124.4, 124.1, 121.7, 35.5; m/z (ESI^+) HRMS: Calculated for $C_{10}H_{10}NO$: 160.0757; Found $[M+H]^+$: 160.0759.

8-Methoxy-1,3-dihydro-2*H*-benzo[*b*]azepin-2-one (2e)

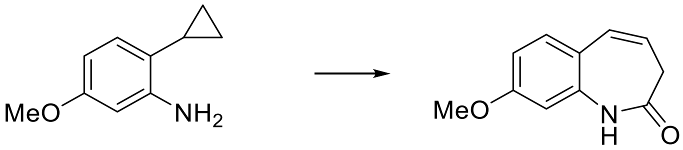

General procedure C: 2-Cyclopropyl-5-methoxyaniline (16.3 mg, 0.10 mmol) was employed. The crude mixture was purified by flash column chromatography (10:1 to 1:1, pentane/EtOAc), to yield 8-Methoxy-1,3-dihydro-2*H*-benzo[*b*]azepin-2-one (17.0 mg, 90%) as a pale yellow solid; m.p.: 127 – 129 °C (DCM/Hex); ν_{max} / cm^{-1} : 3209 (m), 1672 (s), 1615 (m), 1572 (m), 1342 (m), 1217 (m); ^1H NMR (400 MHz, $CDCl_3$): δ 8.59 – 8.36 (1H, br. s), 7.20 (1H, d, *J* = 8.5 Hz), 6.73 (1H, dd, *J* = 8.5, 2.5 Hz),

6.68 (1H, d, J = 10.0 Hz), 6.56 (1H, d, J = 2.5 Hz), 5.87 (1H, dt, J = 10.0, 7.0 Hz), 3.83 (3H, s), 2.92 (2H, d, J = 7.0 Hz); ^{13}C NMR (101 MHz, $CDCl_3$): δ 172.1, 159.7, 137.0, 131.4, 130.4, 122.2, 122.1, 111.1, 106.0, 55.6, 35.5; m/z (ESI^+) HRMS: Calculated for $C_{11}H_{12}NO_2$: 190.0863; Found $[M+H]^+$: 190.0863.

8-(Trifluoromethyl)-1,3-dihydro-2*H*-benzo[*b*]azepin-2-one (2f)

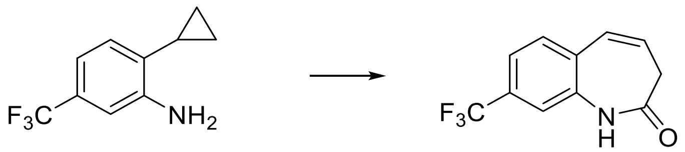

General procedure C: 2-Cyclopropyl-5-(trifluoromethyl)aniline (20.1 mg, 0.10 mmol) was employed. The crude mixture was purified by flash column chromatography (10:1 to 1:1, pentane/EtOAc), to yield 8-(Trifluoromethyl)-1,3-dihydro-2*H*-benzo[*b*]azepin-2-one (15.1 mg, 67%) as a colorless solid; m.p.: 176 – 178 °C (EtOAc/Hex); ν_{max} / cm^{-1} : 3208 (m), 3099 (m), 1678 (s), 1573, 1419 (m), 1313 (m); ^{1}H NMR (400 MHz, $CDCl_3$): δ 8.92 – 8.77 (1H, m), 7.49 – 7.31 (3H, m), 6.80 (1H, d, J = 10.0 Hz), 6.13 (1H, dt, J = 10.0, 7.0 Hz), 2.98 (2H, d, J = 7.0 Hz); ^{13}C NMR (101 MHz, $CDCl_3$): δ 172.3, 136.0, 132.0, 131.0, 130.5 (q, J = 33.0 Hz), 130.0, 126.8, 123.7 (q, J = 273.2 Hz), 120.6 (q, J = 3.7 Hz), 118.8 (q, J = 4.0 Hz), 35.6; ^{19}F NMR (377 MHz, $CDCl_3$): δ -62.6; m/z (ESI^+) HRMS: Calculated for C11H9F_3NO: 228.0631; Found $[M+H]^+$: 228.0634.

5,7,8,9-Tetrahydro-6*H*-pyrido[3,2-*b*]azepin-6-one (2g')

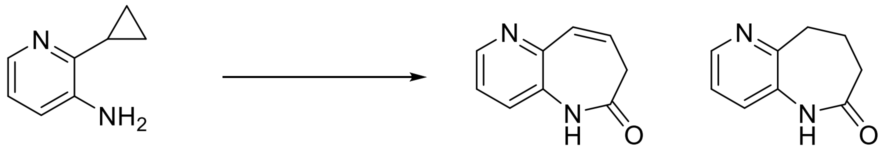

2g **2g'**

To a flame-dried reaction tube, fitted with a magnetic stirrer bar, was added $[Rh(cod)_2]$BARF (8.9 mg, 7.5 µmol), triphenylarsine (4.6 mg, 15 µmol), dimethyl fumarate (14.4 mg, 0.10 mmol) and 2-cyclopropylpyridin-3-amine (13.4 mg, 0.10 mmol). The tube was fitted with a rubber septum and subjected to three argon/vacuum cycles. Anhydrous mesitylene (1.0 mL) was added and the solution was subsequently sparged with carbon monoxide for *ca.* 20 seconds. The solution was heated to 120 °C under a carbon monoxide atmosphere (1 atm) for 48 h, with vigorous stirring throughout (>1000 rpm). The mixture was cooled to r.t. and concentrated *in vacuo*. Purification was achieved by flash column chromatography (10:1, Hexane/EtOAc to 100% EtOAc to 20:1, EtOAc/MeOH) which gave 5,7,8,9-tetrahydro-6*H*-pyrido[3,2-*b*]azepin-6-one (10.7 mg, 66%) as a pale yellow solid and 5,7-dihydro-6H-pyrido[3,2-*b*]azepin-6-one (2.5 mg, 16%) as a pale yellow oil: **2g'**; m.p.: 164 – 165°C (CH_2Cl_2/Hex); ν_{max} / cm^{-1} : 3202 (m), 1677 (s), 1450 (m); ^{1}H NMR (400 MHz, $CDCl_3$): δ 8.36 (1H, dd, J = 5.0, 1.5 Hz), 7.68 – 7.56 (1H, br. s), 7.27 (1H, dd, J = 8.0, 1.5 Hz), 7.20 (1H, dd, J = 8.0, 5.0 Hz), 3.07 (2H, t, J = 7.0 Hz), 2.46 – 2.32 (4H, m); ^{13}C NMR (101 MHz, $CDCl_3$): δ 174.7, 154.9, 146.3, 134.1, 129.0, 122.6,

33.4, 33.1, 27.4; m/z (ESI$^+$) HRMS: Calculated for $C_9H_{11}N_2O$: 163.0866; Found $[M+H]^+$: 163.0860; Characteristic peaks for **2g**: ^{1}H NMR (400 MHz, $CDCl_3$): δ 8.48 (1H, dd, *J* = 4.5, 1.5 Hz), 8.46 – 8.33 (1H, br. s), 7.41 – 7.35 (1H, m), 7.29 – 7.24 (2H, m), 6.97 (1H, d, *J* = 10.0 Hz), 6.25 (1H, dt, *J* = 10.0, 7.0 Hz), 3.01 (2H, d, *J* = 7.0 Hz); ^{13}C NMR (101 MHz, $CDCl_3$): δ 172.1, 158.1, 145.6, 132.4, 129.2, 127.7, 122.8, 35.65. All physical and spectroscopic properties are consistent with those previously reported.[9]

4-Methyl-1,3-dihydro-2*H*-benzo[*b*]azepin-2-one (2h)

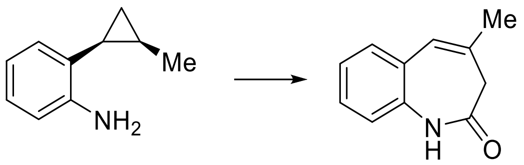

General procedure C: (*rac*)-(*cis*)-2-(2-methylcyclo-propyl)aniline (14.7 mg, 0.10 mmol) was employed. The crude mixture was purified by flash column chromatography (10:1 to 1:1, pentane/EtOAc), to yield 4-methyl-1,3-dihydro-2*H*-benzo[*b*]azepin-2-one (8.0 mg, 46%) as a colorless clear oil; ν_{max} / cm^{-1} : 1667 (s), 1605, 1575 (m), 1373 (m), 752 (s); ^{1}H NMR (400 MHz, $CDCl_3$): δ 8.37 – 8.12 (1H, br. s), 7.26 – 7.22 (2H, m), 7.19 – 7.10 (1H, m), 7.02 (1H, d, *J* = 16.1 Hz), 6.51 (1H, s), 2.87 (2H, s), 2.11 (3H, s); ^{13}C NMR (101 MHz, $CDCl_3$): δ 171.3, 135.3, 135.0, 129.9, 129.7, 127.6, 124.9, 124.1, 121.5, 40.8, 25.1; m/z (ESI$^+$) HRMS: Calculated for $C_{11}H_{12}NO$: 174.0913; Found $[M+H]^+$: 174.0915.

4-Methyl-1,3,4,5-tetrahydro-2*H*-benzo[*b*]azepin-2-one (2h')

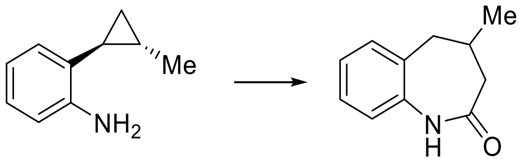

General procedure C: (*rac*)-(*cis*)-2-(2-methylcyclopropyl)aniline (14.7 mg, 0.10 mmol) was employed. The crude mixture was purified by flash column chromatography (10:1 to 1:1, pentane/EtOAc), to yield the 4-methyl-1,3,4,5-tetrahydro-2*H*-benzo[*b*]azepin-2-one (13.5 mg, 77%) as a colorless solid; m.p.: 110 – 112 °C (EtOAc/Hex); ν_{max} / cm^{-1} : 1666 (s), 1606, 1490 (m), 1376 (m); ^{1}H NMR (400 MHz, $CDCl_3$): δ 7.79 – 7.62 (1H, br. s), 7.26 – 7.08 (3H, m), 7.05 – 6.92 (1H, m), 2.95 (1H, dd, *J* = 13.5, 7.0 Hz), 2.62 (1H, dq, *J* = 13.5, 7.0 Hz), 2.51 – 2.35 (2H, m), 2.01 (1H, dd, *J* = 12.5, 7.0 Hz), 1.09 (3H, d, *J* = 7.0 Hz); ^{13}C NMR (101 MHz, $CDCl_3$): 174.4, 137.9, 133.4, 130.6, 127.6, 125.6, 121.8, 40.5, 38.4, 35.7, 21.3; m/z (ESI$^+$) HRMS: Calculated for $C_{11}H_{14}NO$: 176.1070; Found $[M+H]^+$: 176.1067.

1-Phenyl-1,3,6,7-tetrahydro-2*H*-azepin-2-one (2i)

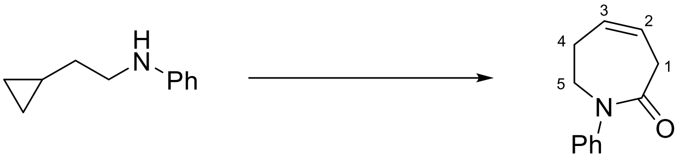

General procedure C: *N*-(2-cyclopropylethyl)aniline (32.2 mg, 0.20 mmol) was employed. The crude mixture was purified by flash column chromatography (10:1 to 2:1, pentane/EtOAc), to yield the major compound phenyl-1,3,6,7-tetrahydro-2*H*-azepin-2-one (19.8 mg, 53%) as a colorless clear oil; ν_{max} / cm^{-1} : 1641 (s), 1593 (m), 1492 (m), 1174 (m). ^1H NMR (400 MHz, $CDCl_3$): δ 7.48 – 7.35 (2H, m), 7.28 – 7.19 (3H, m), 5.81 – 5.64 (2H, m), 4.04 – 3.91 (2H, m), 3.47 – 3.38 (2H, m), 2.52 – 2.37 (2H, m). ^{13}C NMR (101 MHz, $CDCl_3$): δ 173.1, 143.2, 129.4, 129.0, 126.8, 126.7, 121.1, 49.64, 36.7, 29.3. m/z (ESI^+) HRMS: Calculated for $C_{12}H_{14}NO$: 188.1070; Found $[M+H]^+$: 188.1069; Isomer C4-C5 (4.1 mg, 11%) was isolated as a colorless clear oil; ^1H NMR (400 MHz, $CDCl_3$): δ 7.41 – 7.18 (5H, m), 6.08 (1H, dt, *J* = 8.5, 1.3 Hz), 5.64 (1H, dt, *J* = 8.5, 6.5 Hz), 2.73 – 2.65 (2H, m), 2.38 – 2.27 (3H, m), 2.21 (2H, qd, *J* = 7.0, 1.5 Hz); ^{13}C NMR (101 MHz, $CDCl_3$): δ 174.3, 141.1, 131.2, 129.0, 126.8, 126.2, 119.6, 36.2, 28.6, 25.0. All physical and spectroscopic properties are consistent with those previously reported.[10] Isomer C3-C4 and C1-C2 (3.7 mg, 10%) were isolated as an inseparable mixture (3:1, respectively) of alkene regioisomers; characteristic olefinic C-H peaks: Isomer C3-C4; ^1H NMR (400 MHz, $CDCl_3$): δ 5.89 – 5.83 (2H, m); Isomer C1-C2; ^1H NMR (400 MHz, $CDCl_3$): δ 6.32 (1H, dt, *J* = 12.0, 5.5 Hz), 6.12 (1H, dt, *J* = 12.0, 1.5 Hz).

1-Phenylazepan-2-one (2i')

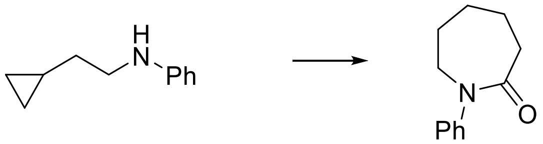

General procedure D: *N*-(2-Cyclopropylethyl)aniline (16.1 mg, 0.10 mmol) was employed. Solvent kept as benzonitrile after the initial step carbonylative step. The crude mixture was purified by flash column chromatography (10:1 to 2:1, pentane/EtOAc), to yield 1-phenylazepan-2-one (13.8 mg, 73%) as a colorless clear oil; ν_{max} / cm^{-1} : 1652 (s), 1594 (m), 1493 (m), 1407 (m), 1215 (m); ^1H NMR (400 MHz, $CDCl_3$): 7.40 – 7.34 (2H, m), 7.25 – 7.17 (3H, m), 3.81 – 3.69 (2H, br. m), 2.75 – 2.64 (2H, br. m), 1.90 – 1.76 (6H, br. m); ^{13}C NMR (101 MHz, $CDCl_3$): δ 175.7, 144.7, 129.2, 126.6, 126.4, 53.2, 37.8, 30.0, 29.1, 23.7; m/z (ESI^+) HRMS: Calculated for $C_{12}H_{16}NO$: 190.1226; Found $[M+H]^+$: 190.1236.

1,6-Diphenyl-1,3,6,7-tetrahydro-2*H*-azepin-2-one (2j)

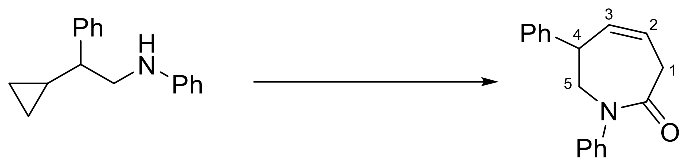

General procedure C: *N*-(2-Cyclopropyl-2-phenylethyl)aniline (23.7 mg, 0.10 mmol) was employed. The crude mixture was purified by flash column chromatography (10:1 to 3:1, pentane/EtOAc), to yield the unsaturated lactam (23.9 mg, 91%) as a mixture of alkene regioisomers (ratio: 1, C1-C2; 6, C2-C3; 3.7, C3-C4; 1.3, C4-C5). 1,6-diphenyl-1,3,6,7-tetrahydro-2*H*-azepin-2-one could be separated after multiple chromatographic purifications as a colorless clear oil; ν_{max} / cm^{-1} : 1669 (s), 1596 (m), 1493 (m), 1407 (m); ^{1}H NMR (400 MHz, $CDCl_3$): δ 7.38 – 7.19 (8H, m), 7.14 – 7.07 (2H, m), 5.98 – 5.89 (1H, m), 5.87 – 5.78 (1H, m), 4.31 – 4.18 (1H, m), 3.93 – 3.75 (3H, m), 3.28 (1H, dd, *J* = 16.0, 7.5 Hz); ^{13}C NMR (101 MHz, $CDCl_3$): δ 172.9, 143.3, 141.1, 132.3, 129.3, 128.9, 128.2, 127.4, 126.8, 126.6, 121.9, 56.7, 45.8, 36.7; m/z (ESI$^+$) HRMS: Calculated for $C_{18}H_{18}NO$: 264.1383; Found $[M+H]^+$: 264.1395. Isomers C4-C5, C3-C4 and C1-C2 were identified by comparative ^{1}H NMR analysis of the title compound; characteristic olefinic C-H peaks: Isomer C4-C5; ^{1}H NMR (400 MHz, $CDCl_3$): δ 6.38 (1H, s); δ Isomer C3-C4; ^{1}H NMR (400 MHz, $CDCl_3$): δ 5.98 (1H, t, *J* = 4.2 Hz); Isomer C1-C2; ^{1}H NMR (400 MHz, $CDCl_3$): δ 6.46 (1H, dt, *J* = 11.5, 5.5 Hz), 6.20 (1H, d, *J* = 12.0 Hz).

1,6-Diphenylazepan-2-one (2j’)

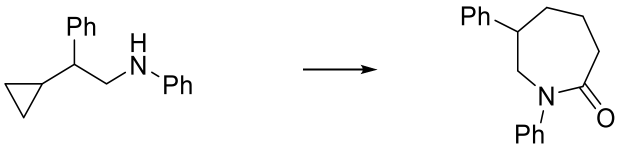

General procedure D: *N*-(2-Cyclopropyl-2-phenylethyl)aniline (23.7 mg, 0.10 mmol) was employed. Solvent was switched to xylenes (2 mL) after the initial step carbonylative step. The crude mixture was purified by flash column chromatography (10:1 to 2:1, pentane/EtOAc), to yield 1,6-diphenylazepan-2-one (23.2 mg, 88%) as a colorless clear oil; ν_{max} / cm^{-1} : 1656 (s), 1596 (m), 1493 (m), 1410 (m); ^{1}H NMR (400 MHz, $CDCl_3$): δ 7.39 – 7.13 (10H, m), 4.20 (1H, dd, *J* = 15.0, 9.9 Hz), 3.67 (1H, dt, *J* = 15.0, 1.5 Hz), 3.03 – 2.95 (1H, m), 2.94 – 2.82 (1H, m), 2.77 (1H, dd, *J* = 14.1, 7.5 Hz), 2.30 – 2.19 (1H, m), 2.19 – 2.08 (1H, m), 1.98 – 1.82 (2H, m); ^{13}C NMR (101 MHz, $CDCl_3$): δ 175.4, 144.7, 144.5, 129.3, 128.9, 126.9, 126.7, 126.7, 126.4, 58.8, 46.4, 38.4, 37.7, 23.8; m/z (ESI$^+$) HRMS: Calculated for $C_{18}H_{20}NO$: 266.1539; Found $[M+H]^+$: 266.1541.

7-Ethyl-1-phenyl-1,3,6,7-tetrahydro-2*H*-azepin-2-one (2k)

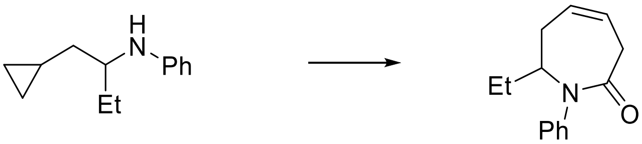

General procedure C: *N*-(1-Cyclopropylbutan-2-yl)aniline (18.9 mg, 0.10 mmol) was employed. The crude mixture was purified by flash column chromatography (10:1 to 2:1, pentane/EtOAc), to yield 7-ethyl-1-phenyl-1,3,6,7-tetrahydro-2*H*-azepin-2-one (12.0 mg, 56%) as a pale yellow oil; ν_{max} / cm^{-1} : 2971, 1644 (s), 1594 (m), 1404 (m), 1356 (m); ^{1}H NMR (400 MHz, $CDCl_3$): δ 7.40 – 7.35 (2H, m), 7.32 – 7.27 (1H, m), 7.14 (2H, d, *J* = 19.2 Hz), 5.83 – 5.57 (2H, m), 4.17 – 4.05 (1H, m), 3.77 – 3.63 (1H, m), 3.25 – 3.08 (1H, m), 2.57 – 2.32 (2H, m), 1.71 – 1.62 (1H, m), 1.33 – 1.16 (1H, m), 0.93 (3H, t, *J* = 7.4 Hz); ^{13}C NMR (101 MHz, $CDCl_3$): δ 173.5, 141.0, 129.1, 129.0, 128.5, 127.5, 121.3, 59.7, 37.4, 33.8, 26.7, 12.0; m/z (ESI^+) HRMS: Calculated for $C_{14}H_{18}NO$: 216.1383; Found $[M+H]^+$: 216.1386.

7-Ethyl-1-phenylazepan-2-one (2k')

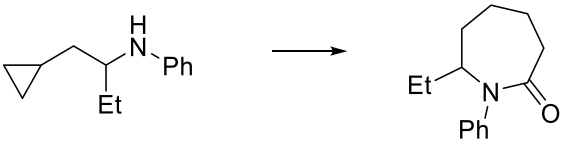

General procedure D: *N*-(1-Cyclopropylbutan-2-yl)aniline (18.9 mg, 0.10 mmol) was employed. Solvent kept as benzonitrile after the initial step carbonylative step. The crude mixture was purified by flash column chromatography (10:1 to 2:1, pentane/EtOAc), to yield 7-ethyl-1-phenylazepan-2-one (15.8 mg, 73%) as a yellow oil; ν_{max} / cm^{-1} : 1645 (s), 1594 (m), 1494 (m), 1393 (m), 1231 (m); ^{1}H NMR (400 MHz, $CDCl_3$): δ 7.45 – 7.25 (3H, m), 7.20 – 7.07 (2H, m), 3.76 – 3.60 (1H, m), 2.84 – 2.61 (2H, m), 1.95 – 1.47 (8H, m), 0.90 (3H, t, *J* = 7.4 Hz); ^{13}C NMR (101 MHz, $CDCl_3$): δ 176.5, 144.0, 130.2, 128.0, 127.0, 63.2, 37.6, 32.3, 26.0, 25.3, 23.4, 11.9; m/z (ESI^+) HRMS: Calculated for $C_{14}H_{19}NNaO$: 240.1359; Found $[M+H]^+$: 240.1363.

1,7-Diphenyl-1,3,6,7-tetrahydro-2*H*-azepin-2-one (2l)

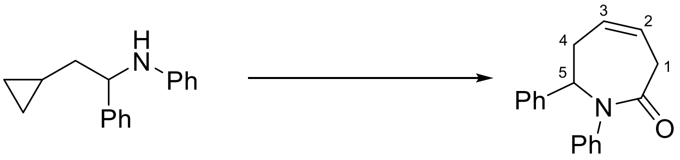

General procedure C: *N*-(2-Cyclopropyl-1-phenylethyl)aniline (23.7 mg, 0.1 mmol) was employed. The crude mixture was purified by flash column chromatography (10:1 to 3:1, pentane/EtOAc), to yield 1,7-diphenyl-1,3,6,7-tetrahydro-2*H*-azepin-2-one (21.0 mg, 80%) as a mixture of alkene regioisomers (ratio: 0, C1-C2; 8, C2-C3; 1, C3-C4; 0, C4-C5); 1,7-diphenyl-1,3,6,7-tetrahydro-2*H*-azepin-2-one could be separated after multiple chromatographic purifications as a colorless clear oil; ν_{max} / cm^{-1} : 1649

(s), 1595 (m), 1494 (m), 1298 (m); ^{1}H NMR (400 MHz, $CDCl_3$): δ 7.36 – 7.16 (10H, m), 5.93 – 5.85 (1H, m), 5.70 – 5.58 (1H, m), 5.31 – 5.24 (1H, m), 3.36 – 3.23 (1H, m), 3.14 – 3.02 (1H, m), 2.96 – 2.79 (2H, m); ^{13}C NMR (101 MHz, $CDCl_3$): δ 173.1, 143.1, 139.4, 129.3, 128.7, 127.9, 127.8, 127.4, 127.1, 127.1, 121.7, 61.7, 37.8, 31.3; m/z (ESI^+) HRMS: Calculated for $C_{18}H_{18}NO$: 264.1383; Found $[M+H]^+$: 264.1388. Isomer C3-C4 was identified by comparative ^{1}H NMR analysis of the title compound; characteristic olefinic C-H peaks: Isomer C3-C4; ^{1}H NMR (400 MHz, $CDCl_3$): δ 6.18 – 6.07 (1H, m), 5.62 – 5.56 (1H, m).

1,7-Diphenylazepan-2-one (2l')

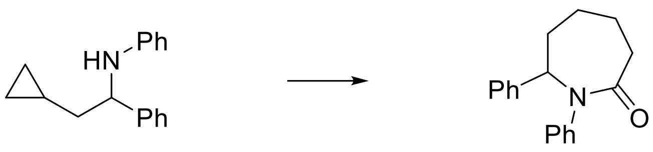

General procedure D: *N*-(2-Cyclopropyl-1-phenylethyl)aniline (23.7 mg, 0.10 mmol) was employed. Solvent kept as benzonitrile after the initial step carbonylative step. The crude mixture was purified by flash column chromatography (10:1 to 2:1, pentane/EtOAc), to yield 1,7-diphenylazepan-2-one (17.6 mg, 66%) as a yellow oil; ν_{max} / cm^{-1} : 1646 (s), 1595 (m), 1423 (m), 1225 (m); ^{1}H NMR (400 MHz, $CDCl_3$): δ 7.41 – 7.18 (10H, m), 5.13 (1H, dd, *J* = 6.5, 3.0 Hz), 2.72 – 2.58 (2H, m), 2.54 – 2.39 (1H, m), 2.31 – 2.17 (1H, m), 1.90 – 1.69 (4H, m); ^{13}C NMR (101 MHz, $CDCl_3$): δ 176.2, 145.5, 139.7, 129.3, 129.1, 127.1, 126.8, 126.7, 126.6, 64.9, 38.2, 32.6, 24.4, 23.6; m/z (ESI^+) HRMS: Calculated for $C_{18}H_{20}NO$: 266.1539; Found $[M+H]^+$: 266.153.

7-(Naphthalen-2-yl)-1-phenyl-1,3,6,7-tetrahydro-2*H*-azepin-2-one (2m)

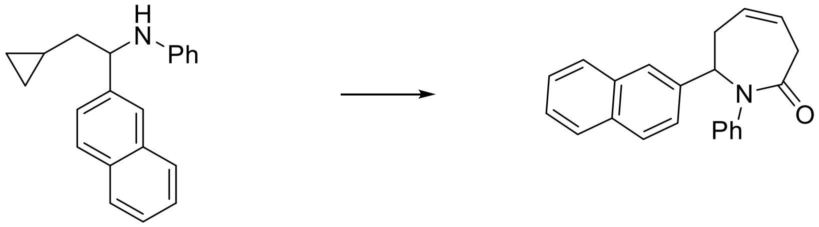

General procedure C: *N*-(2-Cyclopropyl-1-(naphthalen-2-yl)ethyl)aniline (28.7 mg, 0.10 mmol) was employed. The crude mixture was purified by flash column chromatography (10:1 to 2:1, hexane/EtOAc), to yield 7-(naphthalen-2-yl)-1-phenyl-1,3,6,7-tetrahydro-2*H*-azepin-2-one (21.3 mg, 68%) as a yellow solid; m.p.: 133 – 136 °C (EtOAc/Hex); ν_{max} / cm^{-1} : 1646 (s), 1595 (m), 1421 (m), 1274 (m), 906 (s); ^{1}H NMR (400 MHz, $CDCl_3$): δ 7.86 – 7.77 (4H, m), 7.56 – 7.43 (3H, m), 7.40 – 7.29 (2H, m), 7.28 – 7.19 (3H, m), 6.00 – 5.87 (1H, m), 5.72 – 5.58 (1H, m), 5.46 – 5.31 (1H, m), 3.35 – 3.12 (2H, m), 3.01 – 2.83 (2H, m); ^{13}C NMR (101 MHz, $CDCl_3$): δ 173.1, 143.3, 136.8, 133.2, 132.7, 129.3, 128.6, 128.2, 127.7, 127.7, 127.3, 127.1, 126.5, 126.4, 125.7, 125.0, 121.8, 61.9, 37.9, 31.3; m/z (ESI^+) HRMS: Calculated for $C_{22}H_{19}NNaO$: 336.1359; Found $[M+Na]^+$: 336.1382.

7-(Naphthalen-2-yl)-1-phenylazepan-2-one (2m’)

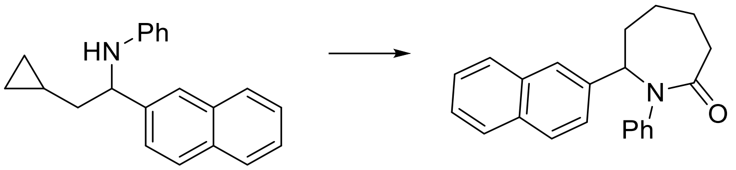

General procedure D: *N*-(2-Cyclopropyl-1-(naphthalen-2-yl)ethyl)aniline (28.7 mg, 0.10 mmol) was employed. Solvent was switched to xylenes (2 mL) after the initial step carbonylative step. The crude mixture was purified by flash column chromatography (10:1 to 3:1, pentane/EtOAc), to yield 7-(naphthalen-2-yl)-1-phenylazepan-2-one as a yellow solid (21.1 mg, 67%); m.p.: 88 – 90 °C (EtOAc/Hex); ν_{max} / cm^{-1} : 1646 (s), 1595 (m), 1494 (m), 1438 (m), 1423 (m); ^{1}H NMR (400 MHz, $CDCl_3$): δ 7.91 – 7.83 (4H, m), 7.55 – 7.47 (2H, m), 7.44 (1H, dd, *J* = 8.6, 1.7 Hz), 7.37 – 7.28 (4H, m), 7.25 – 7.19 (1H, m), 5.37 – 5.16 (1H, m), 2.82 – 2.71 (1H, m), 2.70 – 2.60 (1H, m), 2.59 – 2.48 (1H, m), 2.37 – 2.23 (1H, m), 1.97 – 1.84 (1H, m), 1.83 – 1.68 (3H, m); ^{13}C NMR (101 MHz, $CDCl_3$): δ 176.3, 145.6, 137.2, 133.6, 132.5, 129.3, 128.9, 128.1, 127.7, 126.9, 126.7, 126.6, 126.3, 125.5, 124.5, 65.2, 38.3, 32.8, 24.5, 23.6; m/z (ESI$^+$) HRMS: Calculated for $C_{22}H_{22}NO$: 316.1696; Found $[M+H]^+$: 316.1708.

1-Phenyl-7-(thiophen-3-yl)-1,3,6,7-tetrahydro-2*H*-azepin-2-one (2n)

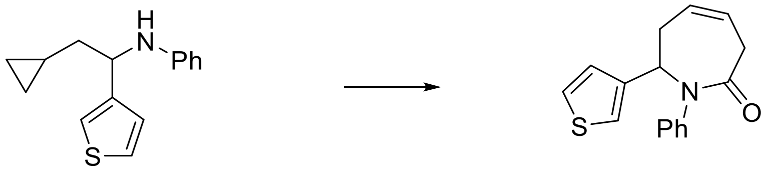

General procedure C: *N*-(2-Cyclopropyl-1-(thiophen-3-yl)ethyl)aniline (24.3 mg, 0.10 mmol) was employed. Modified conditions: the reaction was conducted at 130 ºC for 48 h. The crude mixture was purified by flash column chromatography (10:1 to 2:1, pentane/EtOAc), to yield 1-phenyl-7-(thiophen-3-yl)-1,3,6,7-tetrahydro-2*H*-azepin-2-one (15.9 mg, 59%) as a dark brown oil; ν_{max} / cm^{-1} : 1661 (s), 1652 (s), 1596 (m), 1493 (m), 1422 (m), 1119 (m); ^{1}H NMR (400 MHz, $CDCl_3$): δ 7.38 – 7.33 (2H, m), 7.30 – 7.27 (1H, m), 7.25 – 7.12 (4H, m), 7.04 (1H, dd, *J* = 5.0, 1.5 Hz), 5.88 – 5.83 (1H, m), 5.68 – 5.61 (1H, m), 5.22 (1H, t, *J* = 4.5 Hz), 3.31 – 3.22 (1H, m), 3.07 – 2.82 (3H, m); ^{13}C NMR (101 MHz, $CDCl_3$): δ 173.0, 143.2, 140.9, 129.4, 127.7, 127.2, 127.1, 127.0, 126.3, 122.2, 121.7, 58.6, 37.7, 32.5; m/z (ESI$^+$) HRMS: Calculated for $C_{16}H_{16}NOS$: 270.0947; Found $[M+H]^+$: 270.0953.

1-Phenyl-7-(thiophen-3-yl)azepan-2-one (2n’)

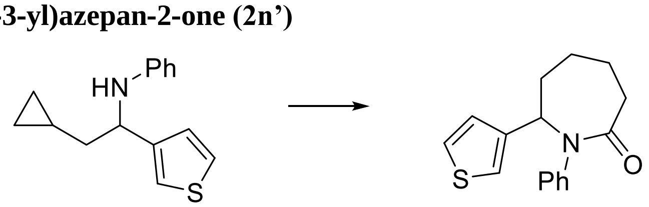

To a flame-dried reaction tube, fitted with a magnetic stirrer bar, was added [Rh(cod)$_2$]BARF (5.9 mg, 5.0 μmol), dimethyl fumarate (14.4 mg, 0.10 mmol) and *N*-(2-Cyclopropyl-1-(thiophen-3-yl)ethyl)aniline (24.3 mg, 0.10 mmol). The tube was fitted with a rubber septum and subjected to three argon/vacuum cycles. Anhydrous benzonitrile (1.0 mL) was added and the solution was subsequently sparged with carbon monoxide for *ca.* 20 seconds. The solution was heated under a carbon monoxide atmosphere (1 atm) at 130 °C for 48 h, with vigorous stirring throughout (>1000 rpm). The mixture was cooled to r.t. and concentrated *in vacuo*, followed by the addition of anhydrous mesitylene (2 mL). To the solution was added xantphos (11.6 mg, 20.0 μmol) and the resulting solution was sparged with hydrogen for *ca.* 60 seconds. The solution was heated under a hydrogen atmosphere (1 atm) at 120 °C for 24 h. The mixture was cooled to r.t. and concentrated *in vacuo*. Purification was achieved by flash column chromatography (10:1 to 2:1, pentane/EtOAc), to yield 1-phenyl-7-(thiophen-3-yl)azepan-2-one as a yellow oil (10.0 mg, 37%); ν_{max} / cm^{-1} : 1652 (s), 1595 (m), 1492 (m), 1422 (m), 1298 (m), 1163 (m); ^{1}H NMR (400 MHz, CDCl$_3$): δ 7.39 – 7.32 (2H, m), 7.28 – 7.17 (4H, m), 7.00 (1H, dd, *J* = 5.0, 1.2 Hz), 5.06 (1H, ddd, *J* = 5.6, 3.0, 1.4 Hz), 2.71 – 2.42 (3H, m), 2.31 – 2.13 (1H, m), 1.91 – 1.65 (2H, m); ^{13}C NMR (101 MHz, CDCl$_3$): δ 176.1, 145.4, 141.6, 129.3, 127.0, 126.9, 126.6, 126.5, 121.4, 62.7, 38.3, 33.3, 24.8, 23.5; m/z (ESI$^+$) HRMS: Calculated for $C_{16}H_{18}NOS$: 272.1104; Found [M+H]$^+$: 272.1106. A small impurity (5-10%) was observed in the aliphatic region of the ^{1}H NMR spectrum, and we were unable to remove this.

7-Cyclopropyl-1-phenyl-1,3,6,7-tetrahydro-2*H*-azepin-2-one (2o)

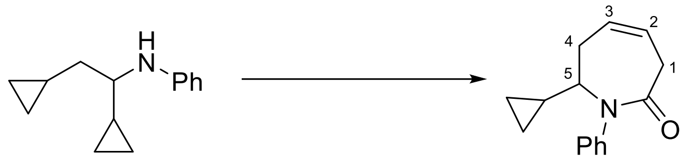

General procedure C: *N*-(1,2-Dicyclopropylethyl)aniline (20.1 mg, 0.1 mmol) was employed. Modified conditions: the reaction was conducted at 130 °C for 48 h. The crude mixture was purified by flash column chromatography (10:1 to 2:1, pentane/EtOAc), to yield 7-cyclopropyl-1-phenyl-1,3,6,7-tetrahydro-2*H*-azepin-2-one (21.0 mg, 80%) as a mixture of alkene regioisomers (ratio: 1, C1-C2; 4.5, C2-C3; 0, C3-C4; 0, C4-C5); 7-cyclopropyl-1-phenyl-1,3,6,7-tetrahydro-2*H*-azepin-2-one could be separated after multiple chromatographic purifications as a colorless clear oil; ν_{max} / cm^{-1} : 1663 (s), 1646 (s), 1595 (m), 1495 (m), 1408 (m), 1372 (m), 1181; ^{1}H NMR (400 MHz, CDCl$_3$): δ 7.42 – 7.28 (3H, m), 7.23 – 7.17 (2H, m), 5.85 – 5.48 (2H, m), 3.80 – 3.55 (1H, m), 3.48 – 3.29 (1H, m), 3.08 (1H, dd, *J* = 16.5, 7.0 Hz), 2.68 – 2.41 (2H, m), 0.64 – 0.52 (1H, m), 0.50 – 0.35 (3H, m), 0.31 – 0.17 (1H, m); ^{13}C NMR (101 MHz, CDCl$_3$): δ 173.4, 140.4, 129.6, 129.0, 128.9, 127.7, 121.3, 63.9, 36.7, 35.4, 15.3, 6.0; m/z (ESI$^+$) HRMS: Calculated for $C_{15}H_{18}NO$: 228.1383; Found [M+H]$^+$: 228.1393; Isomer C1-C2 was identified by comparative ^{1}H NMR analysis of the title compound; characteristic olefinic C-H peaks: Isomer C1-C2; ^{1}H NMR (400 MHz, CDCl$_3$): δ 6.29 – 6.18 (1H, m), 6.08 – 6.03 (1H, m).

7-Cyclopropyl-1-phenylazepan-2-one (2o')

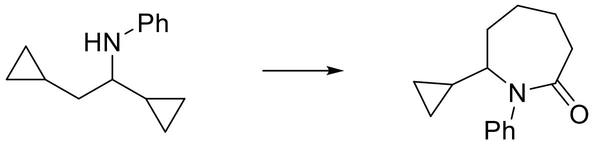

To a flame-dried reaction tube, fitted with a magnetic stirrer bar, was added [Rh(cod)$_2$]BARF (5.9 mg, 5.0 µmol), dimethyl fumarate (14.4 mg, 0.10 mmol) and *N*-(1,2-dicyclopropylethyl)aniline (20.1 mg, 0.10 mmol). The tube was fitted with a rubber septum and subjected to three argon/vacuum cycles. Anhydrous benzonitrile (1.0 mL) was added and the solution was subsequently sparged with carbon monoxide for *ca.* 20 seconds. The solution was heated under a carbon monoxide atmosphere (1 atm) at 130 °C for 48 h, with vigorous stirring throughout (>1000 rpm). The mixture was cooled to r.t. and concentrated *in vacuo*, followed by the addition of anhydrous mesitylene (2 mL). To the solution was added xantphos (11.6 mg, 20.0 µmol) and the resulting solution was sparged with hydrogen for *ca.* 60 seconds. The solution was heated under a hydrogen atmosphere (1 atm) at 120 °C for 24 h. The mixture was cooled to r.t. and concentrated *in vacuo*. Purification was achieved by flash column chromatography (10:1 to 3:1, pentane/EtOAc), to yield 7-cyclopropyl-1-phenylazepan-2-one (10.0 mg, 62%) as a colorless clear oil; ν_{max} / cm^{-1} : 1648 (s), 1595 (m), 1494 (m), 1386 (m), 1203; ^{1}H NMR (400 MHz, CDCl$_3$): δ 7.40 – 7.33 (2H, m), 7.30 – 7.26 (1H, m), 7.20 – 7.15 (2H, m), 2.98 – 2.88 (1H, m), 2.78 – 2.64 (2H, m), 2.08 – 1.91 (3H, m), 1.88 – 1.66 (3H, m), 0.79 – 0.67 (1H, br. m), 0.59 – 0.47 (1H, m), 0.45 – 0.35 (1H, m), 0.35 – 0.24 (1H, m), 0.22 – 0.10 (1H, m); ^{13}C NMR (101 MHz, CDCl$_3$): δ 175.6, 142.9, 129.0, 128.8, 127.2, 67.3, 37.4, 34.6, 27.5, 23.4, 15.4, 6.3, 5.3; m/z (ESI$^+$) HRMS: Calculated for $C_{15}H_{20}NO$: 230.1539; Found [M+H]$^+$: 230.1547.

(*rac*)-(*cis*)-1-Phenyl-3,5a,6,7,8,8a-hexahydrocyclopenta[*b*]azepin-2(1*H*)-one (2p)

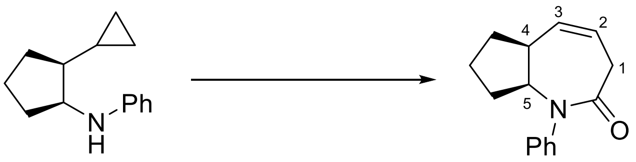

General procedure C: (*rac*)-(*cis*)-*N*-(2-cyclopropylcyclopentyl)aniline (20.1 mg, 0.1 mmol) was employed. The crude mixture was purified by flash column chromatography (10:1 to 3:1, hexane/EtOAc), to yield the unsaturated lactam (*rac*)-(*cis*)-1-phenyl-3,5a,6,7,8,8a-hexahydrocyclopenta[*b*]azepin-2(1*H*)-one (13.6 mg, 60%) as a mixture of alkene regioisomers (ratio: 1, C1-C2; 1.7, C2-C3; 0, C3-C4; 0, C4-C5); Isomers C1-C2 and C2-C3 could not be separated after multiple chromatographic purifications. Isomers C1-C2 and C2-C3 were identified by 2D NMR; Isomer C2-C3; ^{1}H NMR (400 MHz, CDCl$_3$) δ 7.35 – 7.05 (m, 5H), 5.72 – 5.63 (m, 2H), 4.29 (q, J = 6.2 Hz, 1H), 3.44 – 3.34 (m, 1H), 3.19 – 3.08 (m, 1H), 2.79 – 2.70 (m, 1H), 1.90 – 1.76 (m, 2H), 1.62 – 1.49 (m, 2H), 1.46 – 1.22 (m, 2H); Characteristic peaks ^{13}C NMR (101 MHz, CDCl$_3$): δ 170.3, 132.9, 120.6, 62.9, 44.1, 36.6; Isomer C1-C2; ^{1}H NMR (400 MHz, CDCl$_3$) δ 7.35 – 7.05 (m, 5H), 6.43 (ddd, J = 11.0,

7.8, 6.1 Hz, 1H), 6.04 (dd, J = 11.0, 1.5 Hz, 1H), 4.00 (td, J = 9.4, 7.9 Hz, 1H), 2.79 – 2.70 (m, 1H), 2.57 – 2.50 (m, 1H), 2.16 (ddd, J = 13.4, 7.8, 5.0 Hz, 1H), 2.03 – 1.95 (m, 1H), 1.90 – 1.76 (m, 3H), 1.62 – 1.49 (m, 1H), 1.46 – 1.22 (m, 1H); Characteristic peaks ^{13}C NMR (101 MHz, $CDCl_3$): δ173.9, 139.7, 130.1, 66.3, 47.1, 29.8; m/z (ESI$^+$) HRMS: Calculated for isomers C1-C2 and C2-C3, $C_{15}H_{18}NO$: 228.1383; Found $[M+H]^+$: 228.1387.

(*rac*)-(*cis*)-1-Phenyloctahydrocyclopenta[*b*]azepin-2(1*H*)-one 2p'

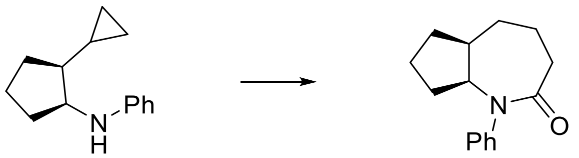

General procedure D: (*rac*)-(*cis*)-*N*-(2-cyclopropylcyclopentyl)aniline (20.1 mg, 0.10 mmol) was employed. Solvent was switched to xylenes (2 mL) after the initial step carbonylative step. The crude mixture was purified by flash column chromatography (10:1 to 2:1, pentane/EtOAc), to yield (*rac*)-(*cis*)-1-phenyloctahydrocyclopenta[*b*]azepin-2(1*H*)-one (17.4 mg, 76%) as a pale brown oil; ν_{max} / cm^{-1}: 1656 (s), 1595, 1383 (m), 1181; ^{1}H NMR (400 MHz, $CDCl_3$): δ 7.42 – 7.32 (2H, m), 7.32 – 7.27 (1H, m), 7.09 (2H, d, J = 16.3 Hz), 4.36 (1H, q, J = 8.5 Hz), 2.80 – 2.70 (1H, m), 2.59 – 2.46 (1H, m), 2.20 – 2.04 (1H, m), 1.93 – 1.65 (6H, m), 1.61 – 1.52 (1H, m), 1.46 – 1.17 (3H, m); ^{13}C NMR (101 MHz, $CDCl_3$): δ 174.4, 142.3, 129.3, 129.2, 127.2, 62.8, 41.6, 34.4, 33.1, 32.9, 27.6, 24.3, 21.3; m/z (ESI$^+$) HRMS: Calculated for $C_{15}H_{19}NNaO$: 252.1359; Found $[M+Na]^+$: 252.1349.

4-Methyl-1-phenyl-1,3,4,5-tetrahydro-2*H*-azepin-2-one (2q)

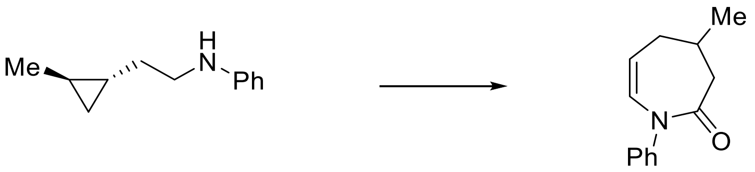

To a flame-dried reaction tube, fitted with a magnetic stirrer bar, was added $[Rh(cod)_2]$BARF (8.9 mg, 7.5 µmol), (*rac*)-(*trans*)-*N*-(2-(2-methylcyclopropyl)ethyl)aniline (17.5 mg, 0.10 mmol) and dimethyl fumarate (14.4 mg, 0.10 mmol). The tube was fitted with a rubber septum and subjected to three argon/vacuum cycles. Anhydrous benzonitrile (1.0 mL) was added and the solution was subsequently sparged with carbon monoxide for *ca.* 20 seconds. The solution was heated under a carbon monoxide atmosphere (1 atm) at 135 °C for 72 h, with vigorous stirring throughout (>1000 rpm). The mixture was cooled to r.t. and concentrated *in vacuo*. Purification was achieved by flash column chromatography (20:1 to 5:1, pentane/EtOAc) to give 4-methyl-1-phenyl-1,3,4,5-tetrahydro-2*H*-azepin-2-one (11.2 mg, 56%) as an pale yellow oil; ν_{max} / cm^{-1} : 1669 (s), 1640 (m), 1595 (m), 1494 (m), 1185 (m); ^{1}H NMR (400 MHz, $CDCl_3$): δ 7.43 – 7.36 (2H, m), 7.29 – 7.23 (3H, m), 6.14 (1H, dt, J = 8.0, 1.0 Hz), 5.75 (1H, dt, J = 8.0, 7.0 Hz), 2.80 – 2.72 (1H, m), 2.72 – 2.61 (1H, m), 2.50 – 2.39 (2H, m), 2.09 – 1.93 (1H, m), 1.12 (3H, d, J = 6.7 Hz); ^{13}C NMR (101 MHz, $CDCl_3$): δ 173.4, 140.8, 131.5, 129.0, 126.7, 126.0,

119.8, 43.7, 37.6, 32.5, 21.7; m/z (ESI$^+$) HRMS: Calculated for $C_{13}H_{16}NO$: 202.1232; Found [M+H]$^+$: 202.1226. Analysis of the crude mixture by ^{1}H NMR indicated that the product was formed with >10:1 regioselectivity.

(*R*)-4-Methyl-1-phenyl-1,3,4,5-tetrahydro-2*H*-azepin-2-one (2q)

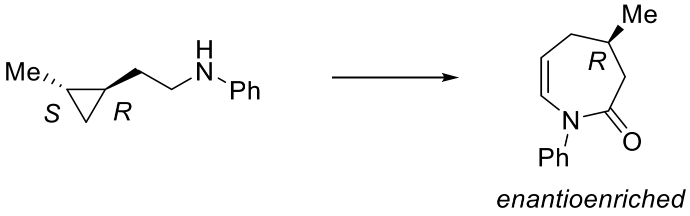

To a flame-dried reaction tube, fitted with a magnetic stirrer bar, was added [Rh(cod)$_2$]BARF (8.9 mg, 7.5 μmol), *N*-(2-((1*R*,2*S*)-2-methylcyclopropyl)ethyl)aniline (17.5 mg, 0.10 mmol) and dimethyl fumarate (14.4 mg, 0.10 mmol). The tube was fitted with a rubber septum and subjected to three argon/vacuum cycles. Anhydrous benzonitrile (1.0 mL) was added and the solution was subsequently sparged with carbon monoxide for *ca.* 20 seconds. The solution was heated under a carbon monoxide atmosphere (1 atm) at 135 °C for 72 h, with vigorous stirring throughout (>1000 rpm). The mixture was cooled to r.t. and concentrated *in vacuo*. Purification was achieved by flash column chromatography (20:1 to 5:1, pentane/EtOAc) to give (*R*)-4-methyl-1-phenyl-1,3,4,5-tetrahydro-2*H*-azepin-2-one (11 mg, 55%, 99% *e.e*) as a pale yellow oil; The analytical data are in accordance with the **2q**. **[α]^{26}$_D$** -50.7 (c = 0.25, $CHCl_3$). The enantiopurity of this compound was determined by chiral SFC (Chiralpak IE, isocratic CO_2-MeCN 90:10, 2.0 mL/min, 40 °C, 140 bar) against a racemic standard; tR (major – 16.5 min and tR (minor) – 15.2 min.

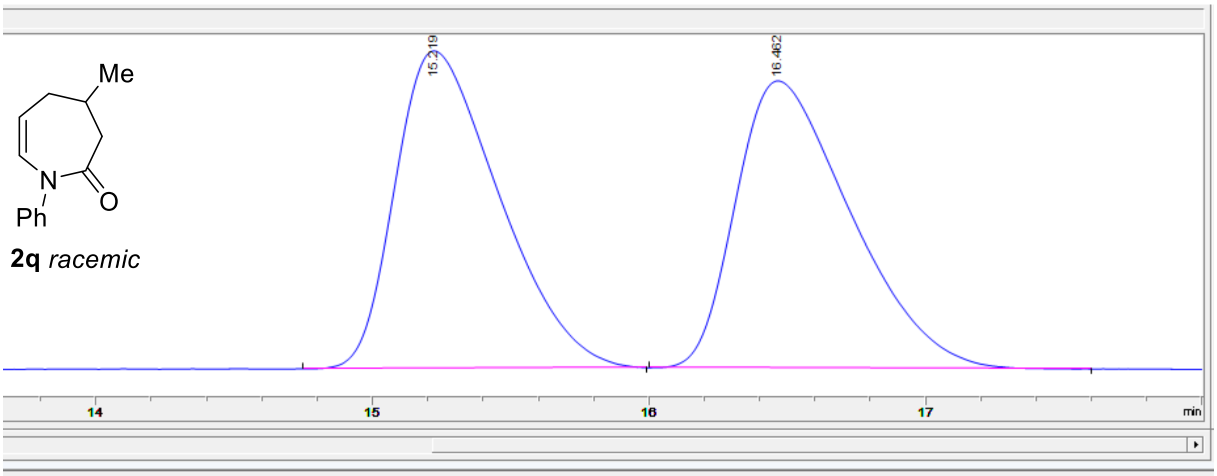

#	Time	Area	Height	Width	Area%	Symmetry
1	15.219	3639.3	139.2	0.3991	50.024	0.571
2	16.462	3635.8	125.9	0.437	49.976	0.556

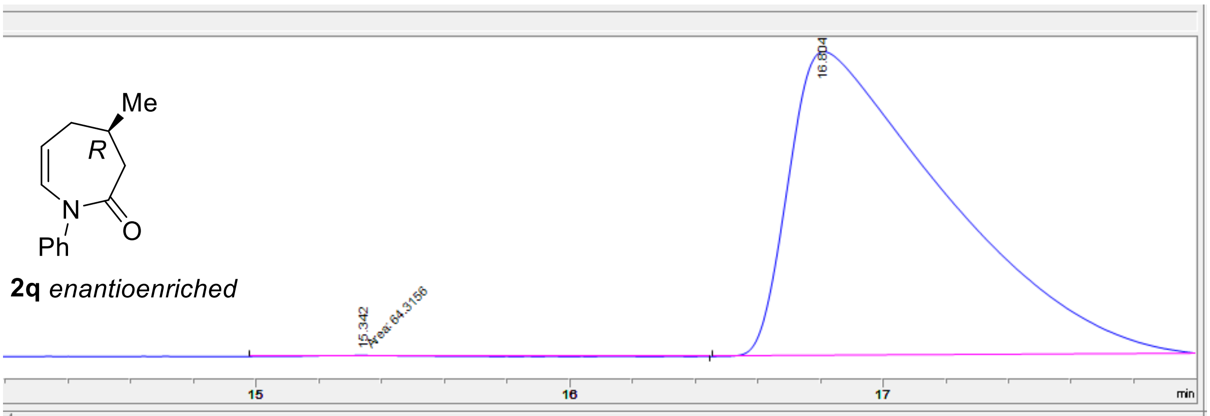

#	Time	Area	Height	Width	Area%	Symmetry
1	15.342	64.3	1.7	0.6404	0.521	0.521
2	16.804	12271.4	354.2	0.4762	99.479	0.266

4-Ethyl-1-phenyl-1,3,4,5-tetrahydro-2*H*-azepin-2-one (2r)

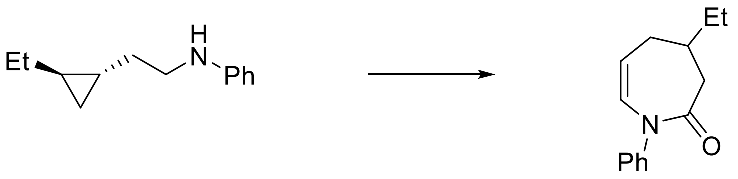

To a flame-dried reaction tube, fitted with a magnetic stirrer bar, was added [Rh(cod)$_2$]BARF (8.9 mg, 7.5 μmol), (*rac*)-(*trans*)-*N*-(2-(2-ethylcyclopropyl)ethyl)aniline (18.9 mg, 0.10 mmol) and dimethyl fumarate (14.4 mg, 0.10 mmol). The tube was fitted with a rubber septum and subjected to three argon/vacuum cycles. Anhydrous benzonitrile (1.0 mL) was added and the solution was subsequently sparged with carbon monoxide for *ca.* 20 seconds. The solution was heated under a carbon monoxide atmosphere (1 atm) at 135 °C for 72 h, with vigorous stirring throughout (>1000 rpm). The mixture was cooled to r.t. and concentrated *in vacuo*. Purification was achieved by flash column chromatography (20:1 to 5:1, pentane/EtOAc) to give 4-ethyl-1-phenyl-1,3,4,5-tetrahydro-2*H*-azepin-2-one (11.8 mg, 55%) as an pale yellow oil; ν_{max} / cm^{-1} : 1674 (s), 1642, 1596 (m), 1495 (m), 1285 (m), 1169; ^{1}H NMR (400 MHz, CDCl$_3$): δ 7.44 – 7.34 (2H, m), 7.26 – 7.18 (3H, m), 6.11 (1H, d, *J* = 8.0 Hz), 5.79 – 5.65 (1H, m), 2.72 (1H, dd, *J* = 11.9, 5.6 Hz), 2.54 – 2.30 (3H, m), 2.12 – 1.94 (1H, m), 1.51 – 1.40 (2H, m), 0.94 (3H, t, *J* = 7.4 Hz); ^{13}C NMR (101 MHz, CDCl$_3$): δ 173.6, 140.7, 131.5, 128.9, 126.6, 126.0, 119.90, 44.5, 41.8, 30.2, 28.8, 11.9; m/z (ESI^{+}) HRMS: Calculated for $C_{14}H_{18}NO$: 216.1383; Found [M+H]$^{+}$: 216.1381. Analysis of the crude mixture by ^{1}H NMR indicated that the product was formed with >10:1 regioselectivity.

1,4-Diphenyl-1,3,4,5-tetrahydro-2*H*-azepin-2-one (2s)

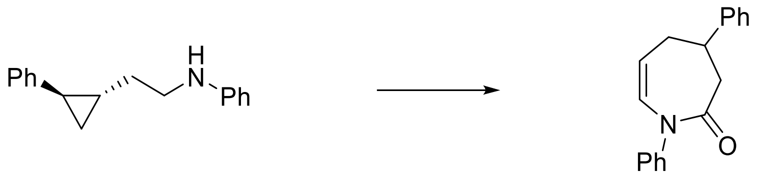

To a flame-dried reaction tube, fitted with a magnetic stirrer bar, was added [Rh(cod)$_2$]BARF (8.9 mg, 7.5 µmol), (*rac*)-(*trans*)-*N*-(2-(2-phenylcyclopropyl)ethyl)aniline (23.7 mg, 0.10 mmol) and dimethyl fumarate (14.4 mg, 0.10 mmol). The tube was fitted with a rubber septum and subjected to three argon/vacuum cycles. Anhydrous benzonitrile (1.0 mL) was added and the solution was subsequently sparged with carbon monoxide for *ca.* 20 seconds. The solution was heated under a carbon monoxide atmosphere (1 atm) at 135 °C for 72 h, with vigorous stirring throughout (>1000 rpm). The mixture was cooled to r.t. and concentrated *in vacuo*. Purification was achieved by flash column chromatography (20:1 to 5:1, pentane/EtOAc) to give 1,4-diphenyl-1,3,4,5-tetrahydro-2*H*-azepin-2-one (10.3 mg, 39%) as a pale yellow oil; ν_{max} / cm^{-1} : 1668 (s), 1493 (m), 1344 (m), 1158 (m); ^{1}H NMR (400 MHz, CDCl$_3$): δ 7.46 – 7.19 (10, m), 6.22 (1H, d, *J* = 8.0 Hz), 5.93 – 5.75 (1H, m), 3.90 – 3.65 (1H, m), 3.14 – 2.90 (2H, m), 2.81 – 2.62 (1H, m), 2.52 – 2.34 (1H, m); ^{13}C NMR (101 MHz, CDCl$_3$): δ 172.8, 145.1, 140.7, 131.8, 129.0, 128.8, 126.9, 126.9, 126.7, 126.1, 119. 48.3, 43.2, 32.8; m/z (ESI$^+$) HRMS: Calculated for $C_{18}H_{18}NO$: 264.1383; Found [M+H]$^+$: 264.1394. Although complex, analysis of the crude mixture by ^{1}H NMR indicated that the product was formed with >4:1 regioselectivity.

4,4-Dimethyl-1-phenyl-1,3,4,5-tetrahydro-2*H*-azepin-2-one (2t)

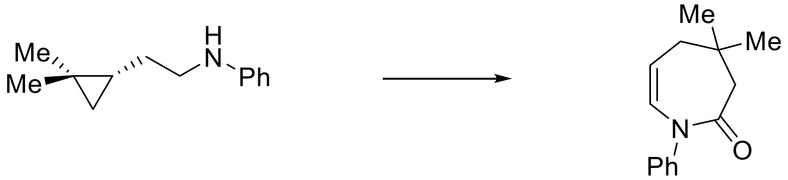

To a flame-dried reaction tube, fitted with a magnetic stirrer bar, was added [Rh(cod)$_2$]BARF (23.6 mg, 20.0 µmol), *N*-(2-(2,2-dimethylcyclopropyl)ethyl)aniline (37.8 mg, 0.20 mmol) and dimethyl fumarate (28.8 mg, 0.20 mmol). The tube was fitted with a rubber septum and subjected to three argon/vacuum cycles. Anhydrous benzonitrile (2.0 mL) was added and the solution was subsequently sparged with carbon monoxide for *ca.* 20 seconds. The solution was heated under a carbon monoxide atmosphere (1 atm) at 145 °C for 72 h, with vigorous stirring throughout (>1000 rpm). The mixture was cooled to r.t. and concentrated *in vacuo*. Purification was achieved by flash column chromatography (15:1 to 10:1, pentane/EtOAc) to give 4,4-dimethyl-1-phenyl-1,3,4,5-tetrahydro-2*H*-azepin-2-one (18.9 mg, 44%) as a pale yellow oil; ν_{max} / cm^{-1} : 1736 (w), 1671 (s), 1640 (m), 1495 (m), 1287 (m); ^{1}H NMR (400 MHz, CDCl$_3$): δ 7.41 – 7.33 (2H, m), 7.26 – 7.18 (3H, m), 6.15 (1H, dt, *J* = 7.5, 1.0 Hz), 5.83 (1H, q, *J* = 7.5 Hz), 2.46 (2H, s), 2.09 (2H, dd, *J* = 7.5, 1.0 Hz), 1.12 (6H, s); ^{13}C NMR (101 MHz, CDCl$_3$): δ 173.0,

140.4, 132.0, 128.9, 126.6, 125.8, 120.9, 49.6, 44.7, 38.4, 29.1; m/z (ESI$^+$) HRMS: Calculated for $C_{14}H_{18}NO$: 216.1383; Found [M+H]$^+$: 216.1390.

(*rac*)-3-Phenyl-3-azabicyclo[4.3.1]decan-4-one (2u')

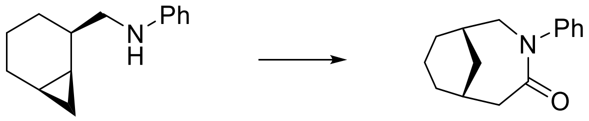

To a flame-dried reaction tube, fitted with a magnetic stirrer bar, was added [Rh(cod)$_2$]BARF (11.8 mg, 10.0 µmol), (*rac*)-N-((bicyclo[4.1.0]heptan-2-yl)methyl)aniline (20.1 mg, 0.10 mmol), 4-nitrobenzoic acid (3.3 mg, 20 µmol) and dimethyl fumarate (14.4 mg, 0.10 mmol). The tube was fitted with a rubber septum and subjected to three argon/vacuum cycles. Anhydrous benzonitrile (1.0 mL) was added and the solution was subsequently sparged with carbon monoxide for *ca.* 20 seconds. The solution was heated under a carbon monoxide atmosphere (1 atm) at 145 °C for 72 h, with vigorous stirring throughout (>1000 rpm). The mixture was cooled to r.t. and concentrated *in vacuo*. Purification was achieved by flash column chromatography (10:1 to 1:1, hexane/EtOAc) to give (*rac*)-3-phenyl-3-azabicyclo[4.3.1]decan-4-one (13.9 mg, 61%) as a pale yellow oil; ν_{max} / cm^{-1} : 1662 (s), 1596 (m), 1494 (m), 1391 (m), 1022 (m); ^{1}H NMR (400 MHz, $CDCl_3$): δ 7.42 – 7.31 (2H, m), 7.27 – 7.15 (3H, m), 3.92 (1H, dd, *J* = 15.5, 9.3 Hz), 3.67 (1H, dd, *J* = 15.6, 7.9 Hz), 2.83 (1H, dd, *J* = 14.0, 8.0 Hz), 2.62 (1H, dd, *J* = 14.0, 8.6 Hz), 2.36 – 2.25 (2H, m), 2.08 – 1.93 (1H, m), 1.91 – 1.81 (1H, m), 1.74 – 1.53 (6H, m); ^{13}C NMR (101 MHz, $CDCl_3$): δ 173.4, 144.2, 128.9, 126.0, 125.5, 54.3, 39.4, 32.9, 29.8, 29.3, 27.8, 25.8, 15.5; m/z (ESI$^+$) HRMS: Calculated for $C_{15}H_{20}NNaO$: 252.1359; Found [M+Na]$^+$: 252.1366.

(*Z*)-2-Benzhydryl-1,4-dihydrobenzo[*c*]azocin-3(2*H*)-one (2v)

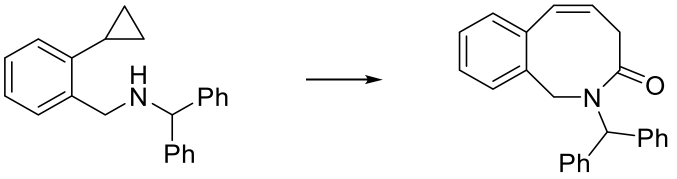

To a flame-dried reaction tube, fitted with a magnetic stirrer bar, was added [Rh(cod)Cl]$_2$ (2.5 mg, 5.0 µmol), dimethyl fumarate (14.4 mg, 0.10 mmol), 4-nitrobenzoic acid (8.4 mg, 50.0 µmol) and *N*-(2-cyclopropylbenzyl)-1,1-diphenylmethanamine (31.3 mg, 0.10 mmol). The tube was fitted with a rubber septum and subjected to three argon/vacuum cycles. Anhydrous benzonitrile (1.0 mL) was added and the solution was subsequently sparged with carbon monoxide for *ca.* 20 seconds. The solution was heated under a carbon monoxide atmosphere (1 atm) at 160 °C for 72 h, with vigorous stirring throughout (>1000 rpm). The mixture was cooled to r.t. and concentrated *in vacuo*. Purification was achieved by flash column chromatography (10:1 to 4:1, petroleum ether 40:60/EtOAc), to yield (*Z*)-2-benzhydryl-1,4-dihydrobenzo[*c*]azocin-3(2*H*)-one (20.7 mg, 61%) as a yellow oil; ν_{max} / cm^{-1} : 1641 (s), 1627 (m), 1595 (m), 1495 (m), 1165 (m); ^{1}H NMR (400 MHz, $CDCl_3$): δ 7.43 – 7.27 (11H, m), 7.23 (1H, d, *J* = 7.4 Hz), 7.14 (1H, d, *J* = 7.5 Hz), 7.06 (1H, t, *J* = 7.6 Hz), 6.89 (1H, d, *J* = 10.5 Hz), 6.25

(1H, d, J = 8.0 Hz), 6.05 (1H, dt, J = 10.5, 8.0 Hz), 4.29 (2H, s), 3.12 (2H, d, J = 8.0 Hz); ^{13}C NMR (101 MHz, $CDCl_3$): δ 168.6, 139.5, 138.5, 134.3, 133.8, 130.5, 129.4, 128.67, 128.2, 128.0, 127.7, 127.6, 124.6, 63.0, 48.7, 39.0; m/z (ESI$^+$) HRMS: Calculated for $C_{24}H_{22}NO$: 340.1696; Found $[M+H]^+$: 340.1709.

(*Z*)-2-Benzhydryl-9-fluoro-1,4-dihydrobenzo[*c*]azocin-3(2*H*)-one (2w)

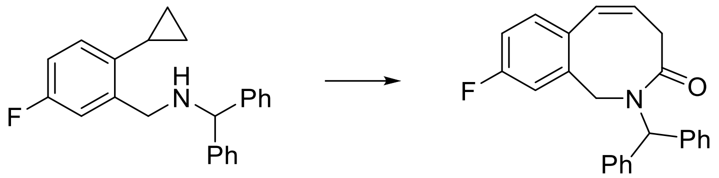

To a flame-dried reaction tube, fitted with a magnetic stirrer bar, was added $[Rh(cod)Cl]_2$ (2.5 mg, 5.0 µmol), dimethyl fumarate (14.4 mg, 0.10 mmol), 4-nitrobenzoic acid (8.4 mg, 50.0 µmol) and *N*-(2-cyclopropyl-5-fluorobenzyl)-1,1-diphenylmethanamine (33.1 mg, 0.10 mmol). The tube was fitted with a rubber septum and subjected to three argon/vacuum cycles. Anhydrous benzonitrile (1.0 mL) was added and the solution was subsequently sparged with carbon monoxide for *ca.* 20 seconds. The solution was heated under a carbon monoxide atmosphere (1 atm) at 160 °C for 72 h, with vigorous stirring throughout (>1000 rpm). The mixture was cooled to r.t. and concentrated *in vacuo*. Purification was achieved by flash column chromatography (10:1 to 4:1, petroleum ether 40:60/EtOAc), to yield (*Z*)-2-benzhydryl-9-fluoro-1,4-dihydrobenzo[*c*]azocin-3(2*H*)-one (17.1 mg, 48%) as a yellow oil; ν_{max} / cm^{-1} : 1642 (s), 1626 (m), 1494 (m), 1154 (m), 843 (m); ^{1}H NMR (400 MHz, $CDCl_3$): 7.43 – 7.34 (6H, m), 7.31 – 7.27 (5H, m), 7.10 (1H, d, J = 14.2 Hz), 6.93 (1H, td, J = 8.5, 2.5 Hz), 6.82 (1H, d, J = 10.5 Hz), 6.05 (1H, dt, J = 10.5, 8.0 Hz), 5.75 (1H, dd, J = 9.5, 2.5 Hz), 4.30 (2H, s), 3.12 (2H, d, J = 8.0 Hz); ^{13}C NMR (101 MHz, $CDCl_3$): δ 168.4, 162.1 (d, J = 248.8 Hz), 139.3, 136.4 (d, J = 7.3 Hz), 134.4 (d, J = 3.2 Hz), 132.8, 129.4 (d, J = 8.2 Hz), 129.2, 128.8, 127.9, 124.8, 117.0 (d, J = 21.8 Hz), 115.4 (d, J = 21.8 Hz), 63.0, 48.2, 39.0; ^{19}F NMR (377 MHz, $CDCl_3$): δ -113.6; m/z (ESI$^+$) HRMS: Calculated for $C_{24}H_{21}FNO$: 358.1602; Found $[M+H]^+$: 358.1608.

(*Z*)-2-Benzhydryl-9-methoxy-1,4-dihydrobenzo[*c*]azocin-3(2*H*)-one (2x)

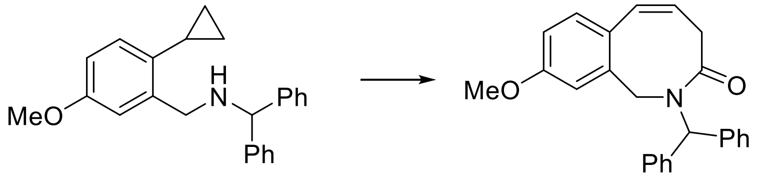

To a flame-dried reaction tube, fitted with a magnetic stirrer bar, was added $[Rh(cod)Cl]_2$ (2.5 mg, 5.0 µmol), dimethyl fumarate (14.4 mg, 0.10 mmol), 4-nitrobenzoic acid (8.4 mg, 50.0 µmol) and *N*-(2-cyclopropyl-5-methoxybenzyl)-1,1-diphenylmethanamine (34.3 mg, 0.10 mmol). The tube was fitted with a rubber septum and subjected to three argon/vacuum cycles. Anhydrous benzonitrile (1.0 mL) was added and the solution was subsequently sparged with carbon monoxide for *ca.* 20 seconds. The solution was heated under a carbon monoxide atmosphere (1 atm) at 160 °C for 72 h, with vigorous

stirring throughout (>1000 rpm). The mixture was cooled to r.t. and concentrated *in vacuo*. Purification was achieved by flash column chromatography (10:1 to 4:1, petroleum ether 40:60/EtOAc), to yield (*Z*)-2-benzhydryl-9-methoxy-1,4-dihydrobenzo[*c*]azocin-3(2*H*)-one (20.7 mg, 56%) as a yellow solid; m.p.: 179 – 180 °C (hexane/IPA); ν_{max} / cm^{-1} : 1642 (s), 1605 (m), 1495 (m), 1459 (m), 1241 (m); ^{1}H NMR (400 MHz, $CDCl_3$): δ 7.43 – 7.24 (11H, m), 7.04 (1H, d, *J* = 8.5 Hz), 7.81 (1H, d, *J* = 10.0 Hz), 6.77 (1H, dd, *J* = 8.5, 3.0 Hz), 5.99 (1H, dt, *J* = 10.0, 8.0 Hz), 5.67 (1H, d, *J* = 3.0 Hz), 4.31 (2H, s), 3.56 (3H, s), 3.13 (2H, dd, *J* = 8.0, 1.0 Hz); ^{13}C NMR (101 MHz, $CDCl_3$): δ 168.7, 159.2, 139.6, 135.7, 133.5, 131.2, 129.5, 128.9, 128.7, 127.7, 123.6, 115.1, 114.4, 62.9, 55.5, 48.6, 39.1; m/z (ESI^+) HRMS: Calculated for $C_{25}H_{24}NO_2$: 370.1802; Found $[M+H]^+$: 370.1818.

(*Z*)-3,6-Dihydrobenzo[*b*]azocin-2(1*H*)-one (2y)

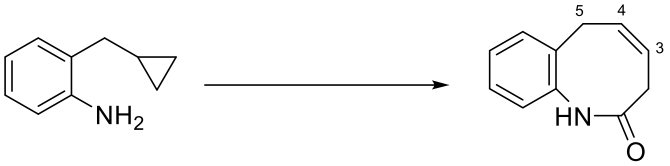

To a flame-dried reaction tube, fitted with a magnetic stirrer bar, was added $[Rh(cod)_2]BARF$ (17.8 mg, 15.0 µmol), dimethyl fumarate (43.2 mg, 0.30 mmol) and 2-(cyclopropylmethyl)aniline (20.1 mg, 0.20 mmol). The tube was fitted with a rubber septum and subjected to three argon/vacuum cycles. Anhydrous benzonitrile (2.0 mL) was added and the solution was subsequently sparged with carbon monoxide for *ca.* 20 seconds. The solution was heated under a carbon monoxide atmosphere (1 atm) at 100 °C for 72 h, with vigorous stirring throughout (>1000 rpm). The mixture was cooled to r.t. and concentrated *in vacuo*. Purification was achieved by flash column chromatography (10:1 to 2:1, pentane/EtOAc), to yield (*Z*)-3,6-dihydrobenzo[*b*]azocin-2(1*H*)-one as a pale brown oil (24.6 mg, 71%) as a mixture of alkene regioisomers (ratio: 7, C3-C4; 2, C4-C5); A sufficient quantity of isomer C3-C4 was obtained which allowed for full characterization; ν_{max} / cm^{-1} : 3178 (m), 3057, 1661 (s), 1645 (m), 1595 (m), 1494 (m), 1166 (m); ^{1}H NMR (400 MHz, $CDCl_3$): δ 7.79 – 7.59 (1H, br. s), 7.34 – 7.24 (3H, m), 7.14 (1H, d, *J* = 7.5 Hz), 5.99 – 5.85 (1H, m), 5.73 – 5.62 (1H, m), 3.46 (2H, d, *J* = 5.0 Hz), 2.82 (2H, d, *J* = 6.5 Hz); ^{13}C NMR (101 MHz, $CDCl_3$): δ 171.7, 136.8, 134.0, 131.0, 129.5, 128.9, 127.9, 125.6, 123.4, 33.8, 32.2; m/z (ESI^+) HRMS: Calculated for $C_{11}H_{11}NO$: 174.0913; Found $[M+H]^+$: 174.0919.

3,4,5,6-Tetrahydrobenzo[*b*]azocin-2(1*H*)-one (2y')

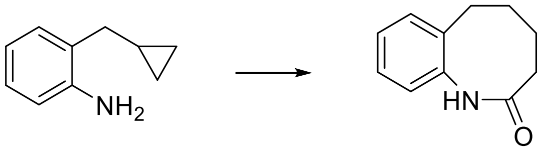

To a flame-dried reaction tube, fitted with a magnetic stirrer bar, was added $[Rh(cod)_2]BARF$ (17.8 mg, 15.0 µmol), dimethyl fumarate (43.2 mg, 0.30 mmol) and 2-(cyclopropylmethyl)aniline (20.1 mg, 0.20

mmol). The tube was fitted with a rubber septum and subjected to three argon/vacuum cycles. Anhydrous benzonitrile (2.0 mL) was added and the solution was subsequently sparged with carbon monoxide for *ca.* 20 seconds. The solution was heated under a carbon monoxide atmosphere (1 atm) at 100 °C for 72 h, with vigorous stirring throughout (>1000 rpm). The mixture was cooled to r.t. and concentrated *in vacuo*, followed by the addition of anhydrous xylenes (2 mL). To the solution was added xantphos (23.2 mg, 40.0 μmol) and the resulting solution was sparged with hydrogen for *ca.* 60 seconds. The solution was heated under a hydrogen atmosphere (1 atm) at 120 °C for 24 h. The mixture was cooled to r.t. and concentrated *in vacuo*. Purification was achieved by flash column chromatography (10:1 to 2:1, pentane/EtOAc), to yield 3,4,5,6-tetrahydrobenzo[*b*]azocin-2(1*H*)-one (27.9 mg, 79%) as a pale brown solid; m.p.: 153 – 154 °C (EtOAc/Hex); ν_{max} / cm^{-1} : 2931 (m), 1655 (s), 1580 (m), 1493 (m); ^1H NMR (400 MHz, $CDCl_3$): δ 7.92 – 7.62 (1H, br. s), 7.30 – 7.25 (2H, m), 7.24 – 7.18 (1H, m), 7.10 – 7.05 (1H, m), 2.90 – 2.49 (2H, br. m), 2.38 – 2.00 (3H, br. m), 2.00 – 1.72 (3H, br. m); ^{13}C NMR (101 MHz, $CDCl_3$): δ 177.0, 140.1, 135.9, 131.1, 128.0, 127.1, 125.3, 32.6, 31.3, 29.7, 25.0. All physical and spectroscopic properties are consistent with those previously reported.[11]

9-Methoxy-3,4,5,6-tetrahydrobenzo[*b*]azocin-2(1*H*)-one (2z')

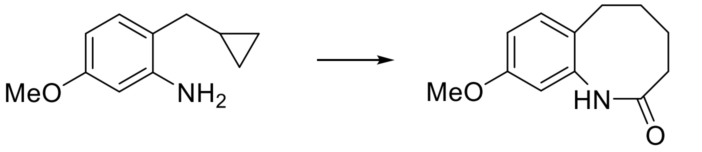

To a flame-dried reaction tube, fitted with a magnetic stirrer bar, was added $[Rh(cod)_2]BARF$ (17.8 mg, 15.0 μmol), dimethyl fumarate (43.2 mg, 0.30 mmol) and 2-(cyclopropylmethyl)-5-methoxyaniline (35.4 mg, 0.20 mmol). The tube was fitted with a rubber septum and subjected to three argon/vacuum cycles. Anhydrous benzonitrile (2.0 mL) was added and the solution was subsequently sparged with carbon monoxide for *ca.* 20 seconds. The solution was heated under a carbon monoxide atmosphere (1 atm) at 100 °C for 72 h, with vigorous stirring throughout (>1000 rpm). The mixture was cooled to r.t. and concentrated *in vacuo*, followed by the addition of anhydrous xylenes (4 mL). To the solution was added xantphos (23.2 mg, 40.0 μmol) and the resulting solution was sparged with hydrogen for *ca.* 60 seconds. The solution was heated under a hydrogen atmosphere (1 atm) at 120 °C for 24 h. The mixture was cooled to r.t. and concentrated *in vacuo*. Purification was achieved by flash column chromatography (10:1 to 1:1, pentane/EtOAc), to yield 9-methoxy-3,4,5,6-tetrahydrobenzo[*b*]azocin-2(1*H*)-one (36.9 mg, 90%) as a pale yellow solid; m.p.: 147 – 148 °C (EtOAc/Hex); ν_{max} / cm^{-1} : 3059 (m) 2933 (m), 1652 (s), 1611 (m), 1285 (m), 1047 (m); ^1H NMR (400 MHz, $CDCl_3$): 7.65 – 7.54 (1H, br. s), 7.17 (1H, d, *J* = 8.5 Hz), 6.82 (1H, dd, *J* = 8.5, 2.7 Hz), 6.60 (1H, s), 3.79 (3H, s), 2.80 – 2.46 (2H, br. m), 2.34 – 2.01 (3H, br. m), 1.95 – 1.73 (3H, br. m); ^{13}C NMR (101 MHz, $CDCl_3$): δ 176.9, 158.5, 136.6, 132.2, 131.8, 114.1, 110.3, 55.6, 32.7, 30.5, 29.8, 25.0; m/z (ESI^+) HRMS: Calculated for $C_{12}H_{16}NO_2$: 206.1176; Found $[M+H]^+$: 206.1174.

9-(Trifluoromethyl)-3,4,5,6-tetrahydrobenzo[*b*]azocin-2(1*H*)-one (2aa')

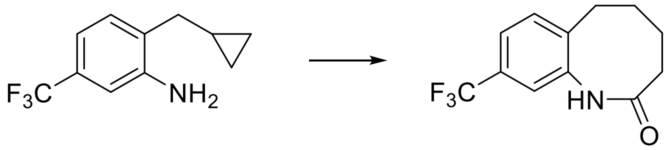

To a flame-dried reaction tube, fitted with a magnetic stirrer bar, was added [Rh(cod)$_2$]BARF (17.8 mg, 15.0 µmol) and dimethyl fumarate (43.2 mg, 0.30 mmol). To the tube was added 2-(cyclopropylmethyl)-5-(trifluoromethyl)aniline (43.0 mg, 0.20 mmol) and the reaction tube was fitted with a rubber septum and subjected to three argon/vacuum cycles. Anhydrous benzonitrile (2.0 mL) was added and the solution was subsequently sparged with carbon monoxide for *ca.* 20 seconds. The solution was heated under a carbon monoxide atmosphere (1 atm) at 100 °C for 72 h, with vigorous stirring throughout (>1000 rpm). The mixture was cooled to r.t. and concentrated *in vacuo*, followed by the addition of anhydrous xylenes (4 mL). To the solution was added xantphos (23.2 mg, 40.0 µmol) and the resulting solution was sparged with hydrogen for *ca.* 60 seconds. The solution was heated under a hydrogen atmosphere (1 atm) at 120 °C for 24 h. The mixture was cooled to r.t. and concentrated *in vacuo*. Purification was achieved by flash column chromatography (10:1 to 1:1, pentane/EtOAc), to yield 9-(trifluoromethyl)-3,4,5,6-tetrahydrobenzo[*b*]azocin-2(1*H*)-one (28.2 mg, 58%) as a colorless solid; m.p.: 157– 159 °C (EtOAc/Hex); ν_{max} / cm^{-1} : 3185 (m), 1658 (s), 1618 (m), 1397 (m), 1169 (m); ^{1}H NMR (400 MHz, CDCl$_3$): δ 8.10 – 7.88 (1H, br. s), 7.51 (1H, d, *J* = 8.0 Hz), 7.42 (1H, d, *J* = 8.0 Hz), 7.36 (1H, s), 2.99 – 2.55 (2H, br. m), 2.28 – 1.49 (6H, br. m); ^{13}C NMR (101 MHz, CDCl$_3$): δ 177.1, 144.0, 136.6, 131.7, 129.7 (q, *J* = 33.0 Hz), 125.1, 124.5 (q, *J* = 3.7 Hz), 122.4 (q, *J* = 3.8 Hz), 32.7, 31.3, 29.5, 24.8; ^{19}F NMR (377 MHz, CDCl$_3$): δ -62.5; m/z (ESI$^+$) HRMS: Calculated for $C_{12}H_{13}F_3NO$: 244.0944; Found [M+H]$^+$: 244.0949.

Additional Studies and Mechanistic Considerations

- **The role of the dimethyl fumarate additive.**

Dimethyl fumarate may function as π-bound ligand, but it also serves as the oxidant, as evidenced by studies on the conversion of **1d** to **2d**. Analysis of the crude reaction mixture shows the formation of stoichiometric amounts of dimethyl succinate which was additionally confirmed by GCMS.

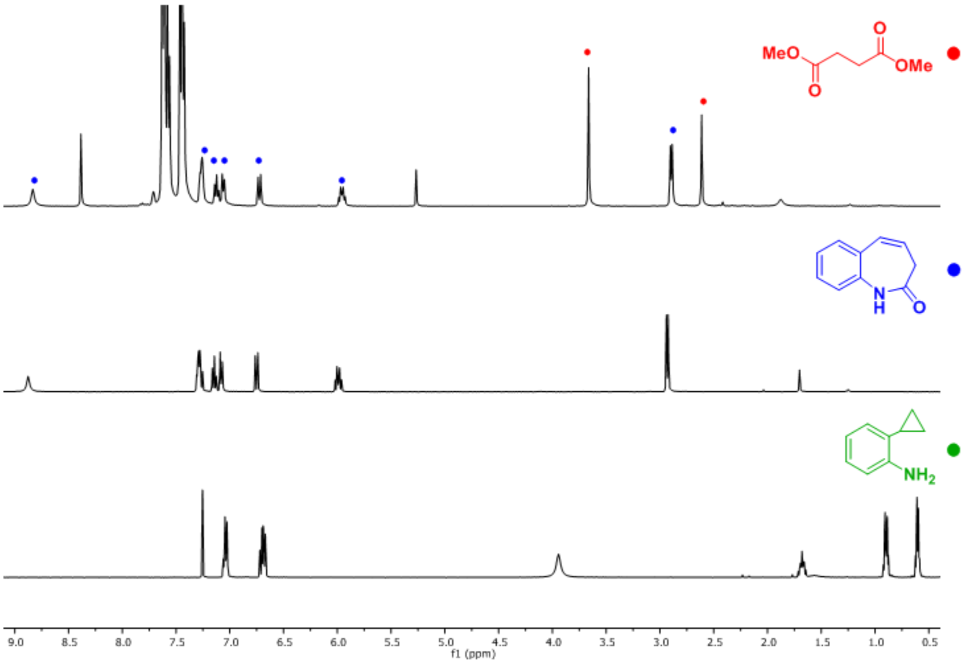

(Top): ^{1}H NMR Spectrum of the crude reaction mixture after concentration *in vacuo*. (Middle): ^{1}H NMR Spectrum of the product lactam **2d**. (Bottom): ^{1}H NMR Spectrum of the starting aminocyclopropane **1d**.

To probe the role of dimethyl fumarate further we conducted a deuterium labelling study:

***Deuterio*-1b** was prepared by repeatedly dissolving **1b** in MeOD-d_4 and concentrating the resulting solution *in vacuo*. 87% deuterium incorporation was measured by ^{1}H NMR analysis. When ***deuterio*-1b** was cyclized under standard conditions (80% yield), 34% deuterium transfer (30% incorporation) to the dimethyl succinate byproduct (68% yield) was observed. This broadly supports the dimethyl fumarate reduction mechanism proposed in the main paper. Incomplete deuterium transfer is likely due to adventitious water.

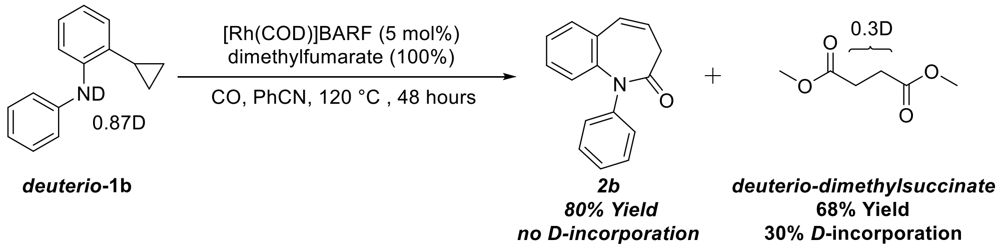

General procedure C: 2-Cyclopropyl-*N*-phenylaniline-*d* (21 mg, 0.10 mmol) was employed. The crude mixture was purified by flash column chromatography (10:1 to 5:1, hexane/EtOAc), to yield 1-phenyl-1,3-dihydro-2*H*-benzo[*b*]azepin-2-one (18.8 mg, 80%) as a colorless solid (^{1}H NMR analysis did not show any deuterium incorporation in the product **2b**) and dimethyl succinate (10 mg, 68%) as a colourless oil. ^{1}H NMR analysis of the latter showed 30% deuterium incorporation (34% transfer) and data are in accordance with the literature.[12] Data for ***deuterio*-dimethylsuccinate**: ^{1}H NMR ($CDCl_3$, 500 MHz): δ 3.70 (6H, s, C1-H), 2.64 (3.7H, s, C2-H); ^{2}H NMR ($CHCl_3$, 500 MHz): δ 2.60 (0.3D, br. s, C2-D).

- **The role of the N-directing group.**

To confirm the role of the N-directing group we performed three insertion experiments to probe the regioselectivity of C-C oxidative addition. These experiments used [Rh(cod)$_2$]BARF/PPh_3, which is a system we have previously found suitable for these types of studies.[13] In the absence of directing groups, we have previously confirmed that non-benzylic mono-substituted cyclopropanes undergo preferential cleavage of the less hindered C-C bond.[13] The selective clevage of the proximal and more hindered C-C bond in ***Experiments 1-3*** is therefore indicative of a directed oxidative addition pathway, with ***Experiments 2 and 3***, which involve non-benzylic systems, offering especially strong support.

Experiment 1

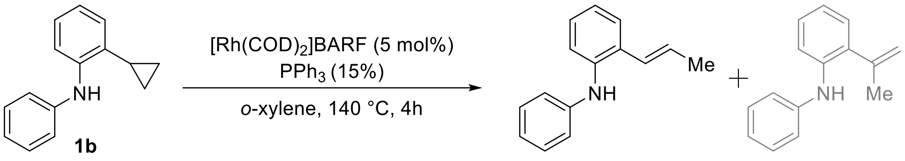

To a flame-dried reaction tube, fitted with a magnetic stirrer bar, was added [Rh(cod)$_2$]BARF (5.9 mg, 5 μmol), 2-cyclopropyl-*N*-phenylaniline (18.9 mg, 0.10 mmol) and PPh_3 (3.93 mg, 15 μmol). The tube was fitted with a rubber septum and subjected to three argon/vacuum cycles. Anhydrous *o*-xylene (1.0 mL) was added. The reaction tube was sealed with a Teflon cap and the solution was subsequently heated at 140 °C for 4 h, with vigorous stirring throughout (>1000 rpm). The mixture was cooled to r.t. and concentrated *in vacuo*. Purification was achieved by flash column chromatography (20:1 to 10:1, pentane/EtOAc) to give (*E*)-N-phenyl-2-(prop-1-en-1-yl)aniline (15.1 mg, 72%) as an colorless oil. The alternate regioisomer was not observed; ν_{max} / cm-1 : 3395 (m), 3020 (m), 1921 (w), 1653 (s), 1592 (s), 1541 (s), 1496 (m), 1297 (m), 966 (m), 743 (m), 692 (m); ^{1}H NMR (500 MHz, $CDCl_3$): δ 7.42 (dd, *J* = 7.7, 1.6 Hz, 1H), 7.32 – 7.23 (m, 3H), 7.18 (td, *J* = 7.7, 1.6 Hz, 1H), 7.06 – 6.96 (m, 3H), 6.95 – 6.89 (m, 1H), 6.56 (dd, *J* = 15.7, 1.9 Hz, 1H), 6.18 (dq, *J* = 15.7, 6.7 Hz, 1H), 5.54 (s, 1H), 1.92 (dd, *J* = 6.6, 1.8 Hz, 3H); 13C NMR (101 MHz, $CDCl_3$): δ144.3, 139.7, 130.2, 129.4, 128.7, 127.8, 127.5, 126.8,

122.5, 120.5, 119.7, 117.4, 19.1; m/z (ESI+) HRMS: Calculated for $C_{15}H_{16}N$: 210.1283; Found [M+H]+: 210.1276.

Experiment 2

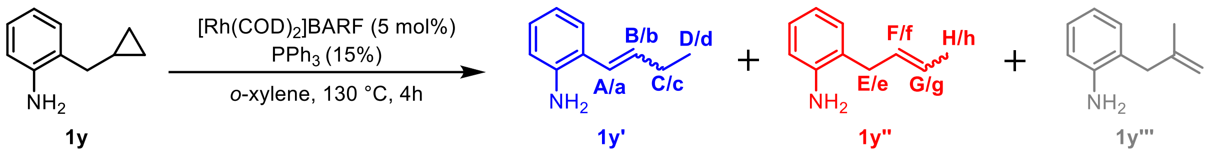

To a flame-dried reaction tube, fitted with a magnetic stirrer bar, was added [Rh(cod)$_2$]BARF (5.9 mg, 5 μmol), 2-(cyclopropylmethyl)aniline (14.7 mg, 0.10 mmol) and PPh_3 (3.93 mg, 15 μmol). The tube was fitted with a rubber septum and subjected to three argon/vacuum cycles. Anhydrous *o*-xylene (1.0 mL) was added. The reaction tube was sealed with a Teflon cap and the solution was subsequently heated at 130 °C for 4 h, with vigorous stirring throughout (>1000 rpm). The mixture was cooled to r.t. and concentrated *in vacuo*. The crude was dissolved in $CDCl_3$ (2 mL) followed by the addition of trichloroethylene (9 μL, 0.1 mmol). Analysis of the crude by ^{1}H NMR showed full conversion of **1y**, 80% yield for **1y'** (ratio **(*E*)-1y'**:**(*Z*)-1y'** = 85:15) and 15% yield for **1y''** (ratio **(*Z*)-1y''**:**(*E*)-1y''** = 2/1). **1y'''** was not observed.[14]

Major isomer (*E*)-1y': ^{1}H NMR (500 MHz, $CDCl_3$): δ 7.23 (dd, *J* = 7.7, 1.5 Hz, 1H), 7.05 (td, *J* = 7.6, 1.5 Hz, 1H), 6.75 (td, *J* = 7.7, 1.5 Hz, 1H), 6.68 (dd, *J* = 7.7, 1.5 Hz, 1H), 6.40 (dt, *J* = 15.6, 1.8 Hz, 1H, **A**), 6.12 (dt, *J* = 15.6, 6.5 Hz, 1H, **B**), 2.25 (qdd, *J* = 7.5, 6.5, 1.8 Hz, 1H, **C**), 1.11 (t, *J* = 7.5 Hz, 1H, **D**).

Minor isomer (*Z*)-1y': *(characteristic signals only)* ^{1}H NMR (500 MHz, $CDCl_3$): δ 6.30 – 6.24 (m, 1H, **a**), 5.80 (dt, *J* = 11.2, 7.4 Hz, 1H, **b**), 2.23 – 2.15 (m, 2H, **c**), 1.03 (t, *J* = 7.5 Hz, 3H, **d**).[15]

Major isomer (*Z*)-1y'': *(characteristic signals only)* ^{1}H NMR (500 MHz, $CDCl_3$): δ 3.26 (d, *J* = 4.8 Hz, 2H, **E**), 1.72 (d, *J* = 4.8 Hz, 3H, **H**).

Minor isomer (*E*)-1y'': *(characteristic signals only)* ^{1}H NMR (500 MHz, $CDCl_3$): 3.32 (d, *J* = 6.8 Hz, 2H, **e**), 1.78 (d, *J* = 6.9 Hz, 3H, **h**).[16]

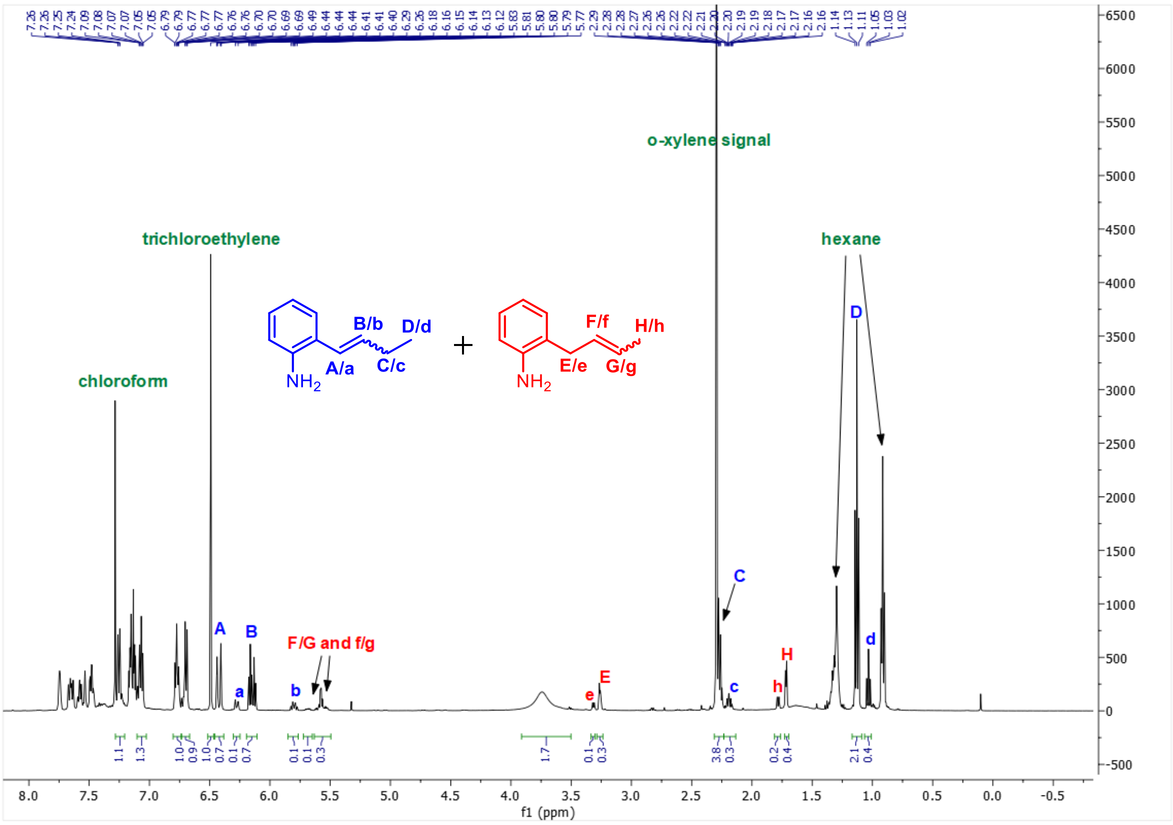

Experiment 3

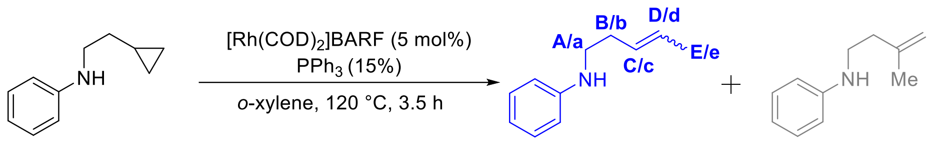

1i **1i'** **1i"**

To a flame-dried reaction tube, fitted with a magnetic stirrer bar, was added [Rh(cod)$_2$]BARF (5.9 mg, 5 µmol), *N*-(2-cyclopropylethyl)aniline (13.5 mg, 0.10 mmol) and PPh_3 (3.93 mg, 15 µmol). The tube was fitted with a rubber septum and subjected to three argon/vacuum cycles. Anhydrous *o*-xylene (1.0 mL) was added. The reaction tube was sealed with a Teflon cap and the solution was subsequently heated at 120 °C for 3.5 h, with vigorous stirring throughout (>1000 rpm). The mixture was cooled to r.t. and concentrated *in vacuo*. NMR analysis against an internal standard showed 50% conversion to **1i'** and 10% remaining **1i**. Regioisomer **1i''** was not observed. Purification was achieved by flash column chromatography (20:1 to 10:1, Hexane/Et_2O) to give *N*-(pent-3-en-1-yl)aniline **1i'** (ratio **(*E*)-1i'/(*Z*)-1i'** = 75:25) (5.5 mg, 41%) with remaining **1i** (highlighted in the ^{1}H NMR spectrum) as a colorless oil.[17]

Major isomer (*E*)-1i’: ^{1}H NMR (500 MHz, $CDCl_3$): δ 7.18 (m, 2H), 6.75 – 6.68 (m, 1H), 6.61 (m, 2H), 5.60 – 5.49 (m, 1H, **C/D**), 5.48 – 5.39 (m, 1H, **C/D**), 3.13 (t, *J* = 6.7 Hz, 2H, **A**), 2.31 (tdd, *J* = 6.7, 6.3, 1.3 Hz, 2H, **B**), 1.69 (dd, *J* = 6.3, 1.4 Hz, 3H, **E**).[17]

Minor isomer (*Z*)-1i’: *(characteristic signals only)* ^{1}H NMR (500 MHz, $CDCl_3$): δ 3.19 (t, *J* = 6.9 Hz, 2H, **a**), 2.41 (q, *J* = 6.9 Hz, 2H, **b**), 1.66 (d, *J* = 6.7 Hz, 3H, **e**).

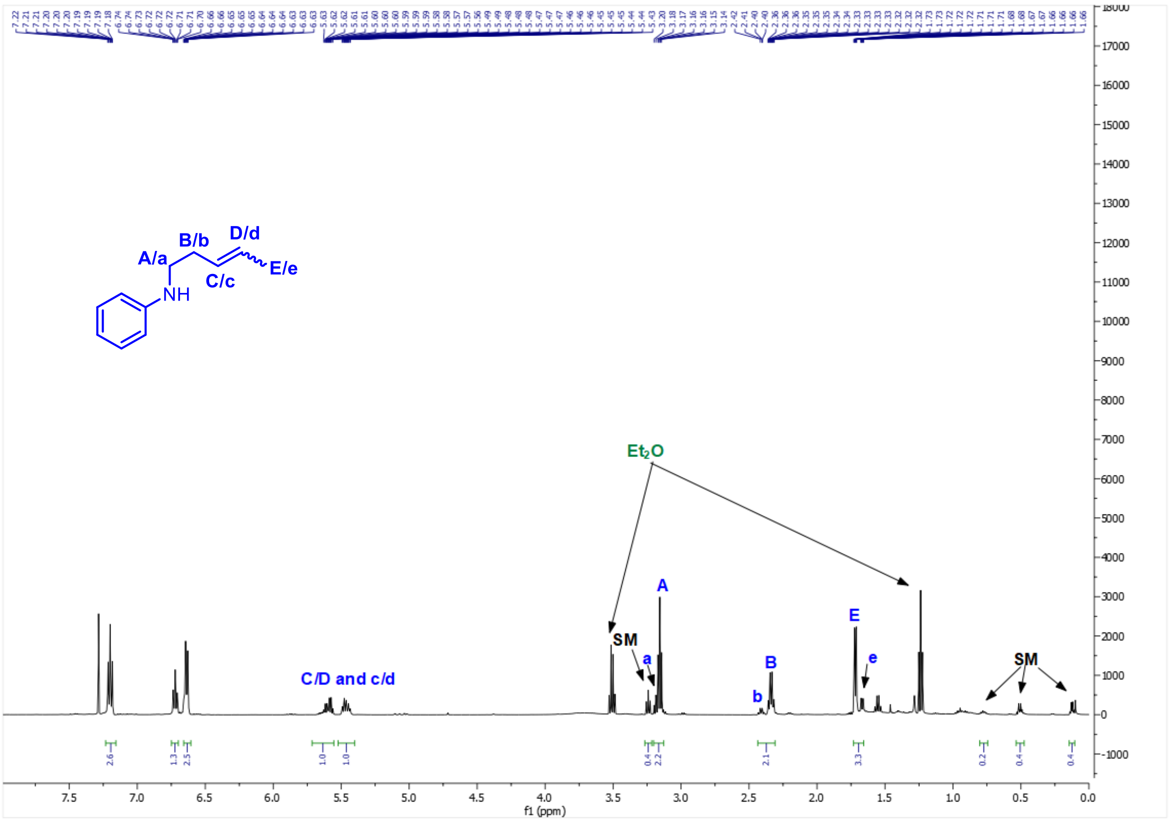

- **Selected optimization results for the transformation of 1a to 2a.**

Table 1 Benzazepines by N-directed carbonylative C-C bond activation optimization.

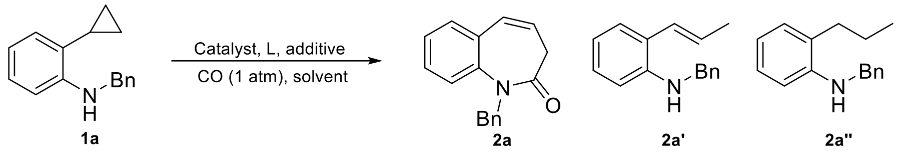

Experiment	Catalyst Rh(I)	Ligand / Additive (mol%)	Solvent	T (°C)	t (h)	Conversion (%)[a] [I.Y. (%)]			
						1a	2a	2a'	2a''
1	[Rh(cod)$_2$]BARF (5)	-	PhCN	100	18	84	16	0	0
2	[Rh(cod)$_2$]BARF (5)	-	PhCN	120	18	44	33	0	13
3	[Rh(cod)$_2$]BARF (5)	-	PhCN	120	48	38	42	0	12
4	[Rh(cod)$_2$]BARF (5)	Dimethyl fumarate (100)	PhCN	120	48	0	73 [72]	trace	trace
5	[Rh(cod)$_2$]BARF (5)	Diisobutyl fumarate (100)	PhCN	120	48	0	72	10	trace
6	[Rh(cod)$_2$]BARF (5)	BzOH (15%)	PhCN	120	48	33	52	0	14
7	[Rh(cod)$_2$]BARF(5)	Dimethyl fumarate (100) BzOH (15%)	PhCN	120	48	22	72	7	0
8	[Rh(cod)$_2$]BARF (5)	AsPh$_3$ (10)	PhCN	120	18	88	8	0	0
9	[Rh(cod)$_2$]BARF (5)	P(3,5-(CF$_3$)$_2$(C$_6$H$_3$))$_3$ (10)	PhCN	120	18	93	6	0	0

Reactions were performed on a 0.1 mmol scale (0.1 M). [a] *In situ* ^{1}H NMR analysis using 1,4-dinitrobenzene as an internal standard, with conversions calculated with respect to the internal standard.

- **Alternate mechanistic options.**

The studies outlined above show that the N-unit functions as a directing group and CO is not required to effect C-C bond oxidative addition. Nevertheless, alternate mechanisms to the one presented in the main paper (**A** to **B** to **C** to **D** to **E** to **F**) are also possible, and are depicted below using a representative generalized substrate (Scheme S1A). For example, following NH metallation to **B**, carbonylation of the N-[Rh] bond could occur to provide **G**, which then undergoes C-C oxidative addition to provide **I**. This requires 6- or 7-membered oxidative addition transition states, which are likely to be more demanding than the 5- or 6-membered variants invoked for **B** to **C**. Additionally, competitive conversion of **G** to N-formyl product **H** (in the presence of a proton source) or a urea (not depicted) might be expected,[18] but side products of this type were not observed during our studies. Finally, if the conversion of **B** to **G** did occur, then the bystander cyclopropane in substrate **1o** in the main paper would be expected to undergo cleavage (5-membered transition state), which is not what we observed (Scheme S1B). **I** could alternatively form via carbonylation of the N-[Rh] bond of **C**. This pathway cannot be discounted although we favor carbonylation to provide **D** because this (a) directly relieves ring strain and (b) allows fast capture of the otherwise unstable rhodacyclobutane,[19] which is prone to rapid β-hydride elimination. Outside of β-hydride elimination triggered isomerization/reduction processes, the productive use of rhodacyclobutanes is predominantly limited to processes that involve carbonylation to the corresponding rhodacyclopentanone (see the main paper). Potential side products of β-hydride elimination from **I** are not depicted and were not observed. We note that β-hydride elimination arguments may not be valid in certain cases due to conformational or configurational constraints (e.g.

1u to **2u'**). If **I** does form, then two possible C-C reductive elimination pathways become feasible, and we have not observed side products related to intermediate **J**. Note that C-N reductive eliminations related to **D** to **E** have precedent from our earlier work.[20]

(A) Possible mechanisms for the carbonylative heterocyclization processes:

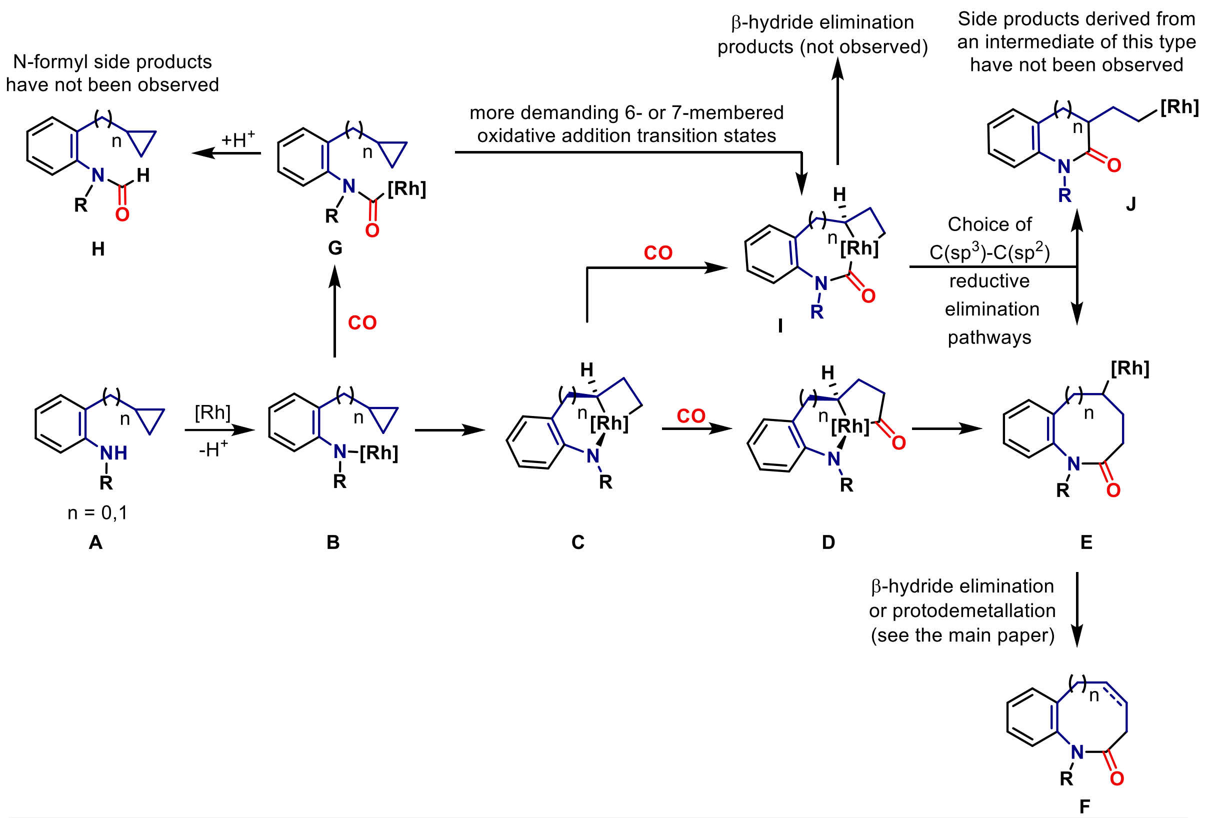

(B) Specific considerations for substrate 1o:

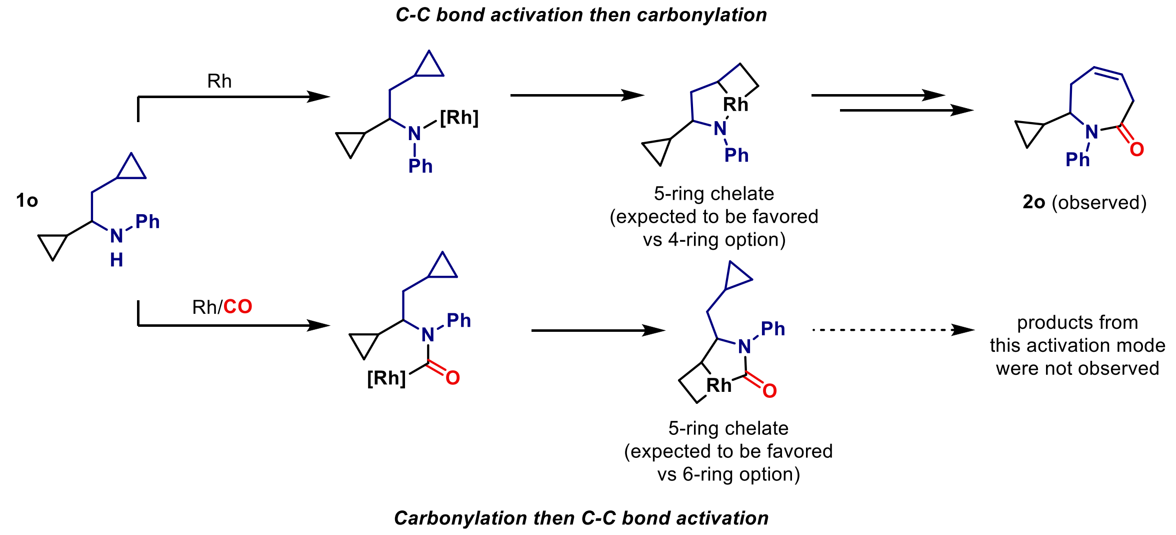

Scheme S1. Mechanistic considerations.

^{1}H and ^{13}C NMR Spectra

1-Cyclopropyl-2-nitrobenzene

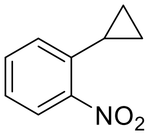

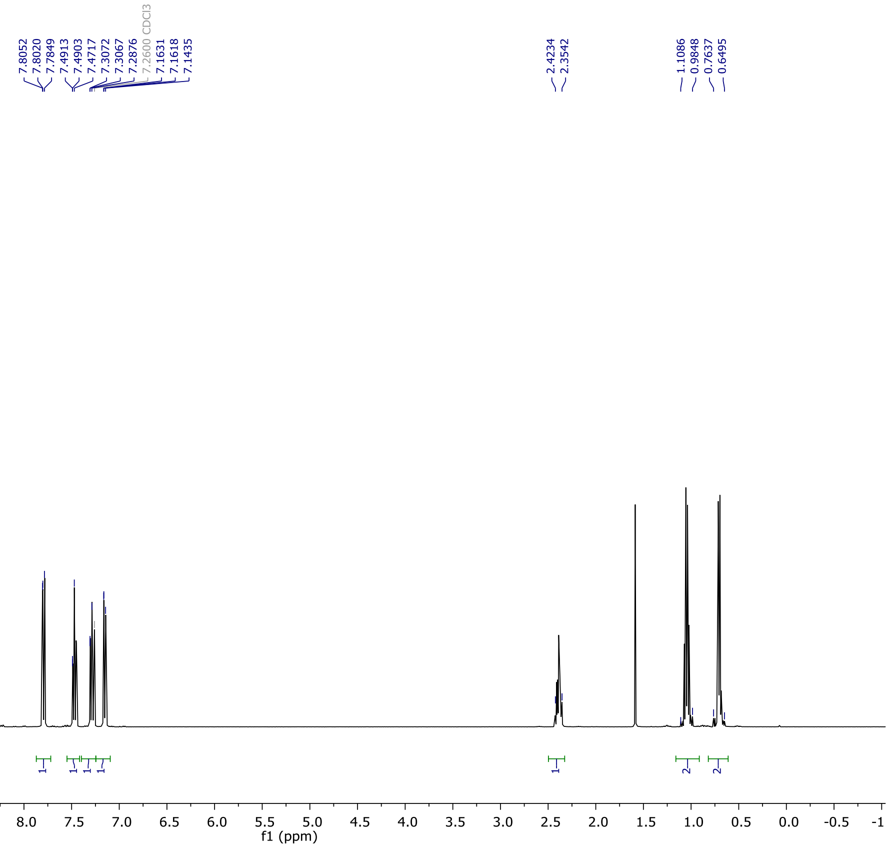

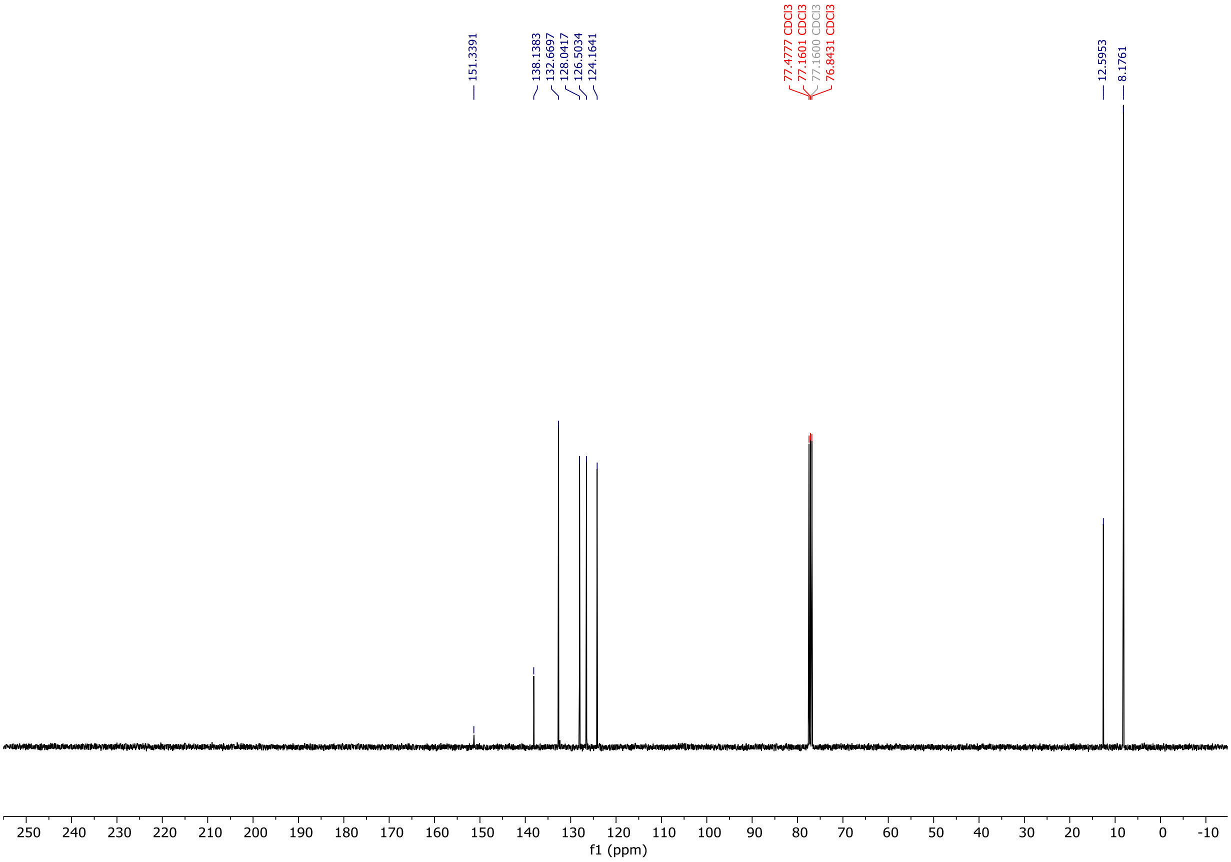

2-Cyclopropylbenzaldehyde

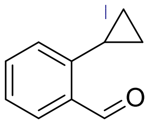

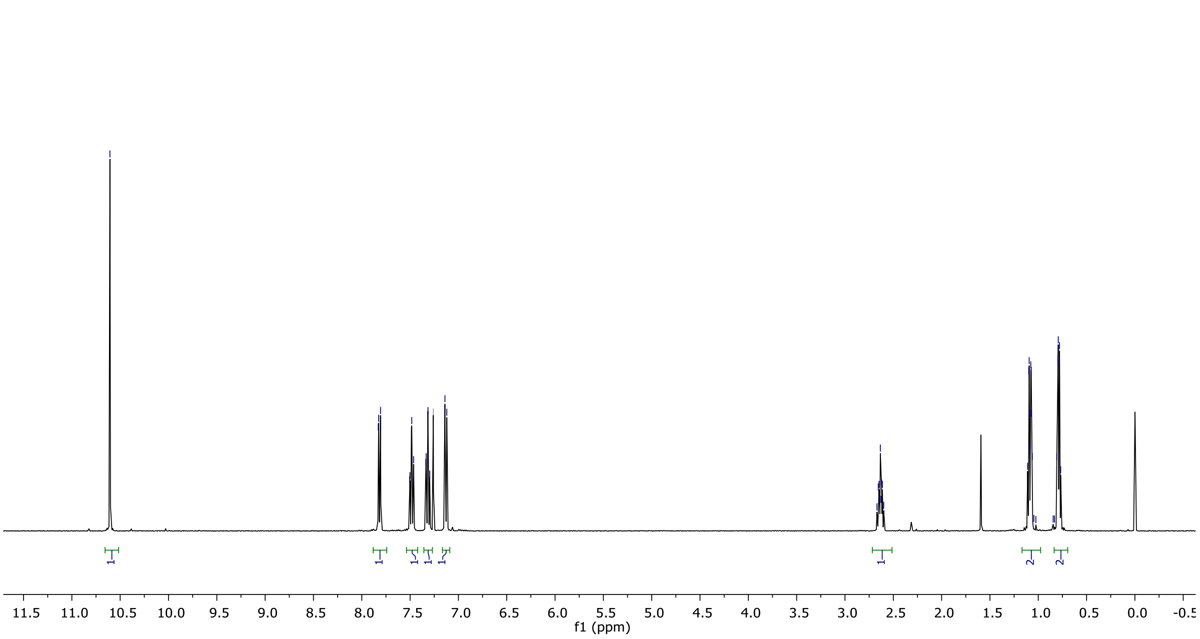

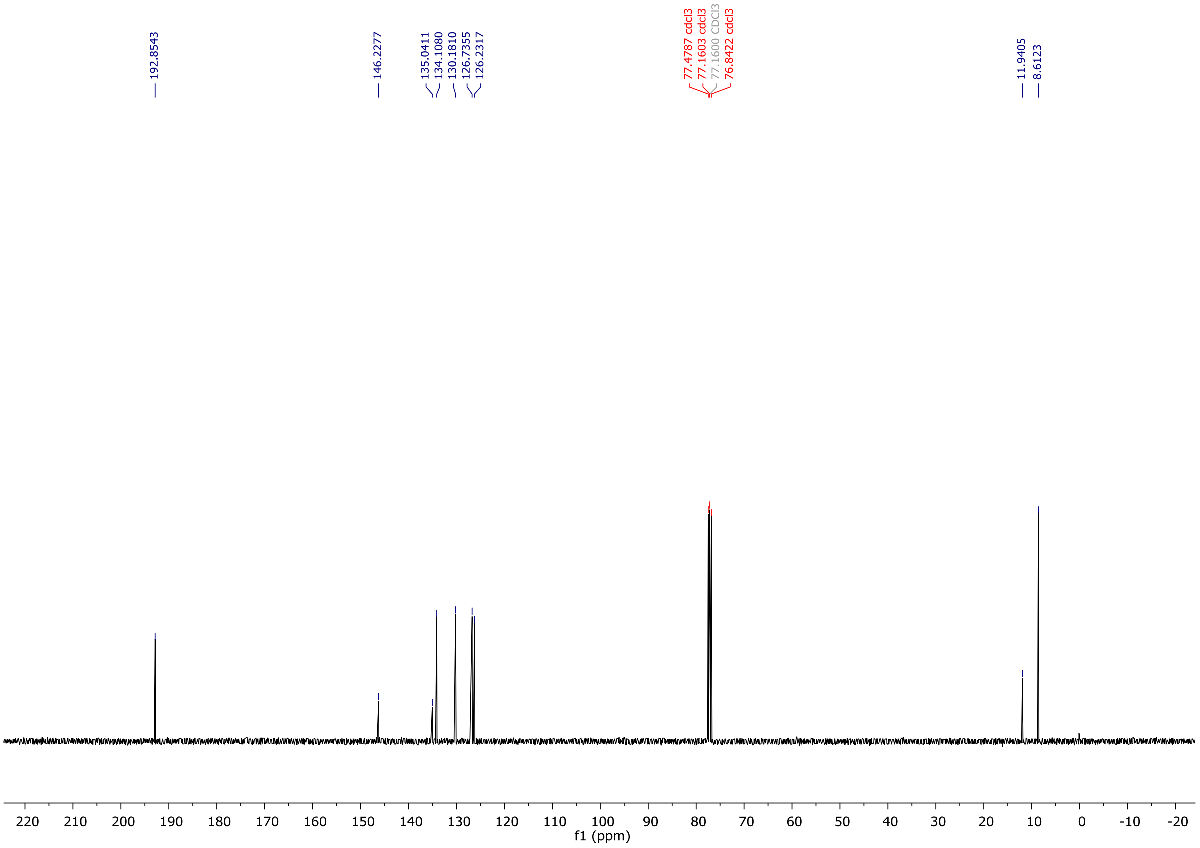

2-Cyclopropyl-*N*-methoxy-*N*-methylacetamide

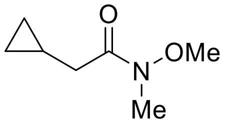

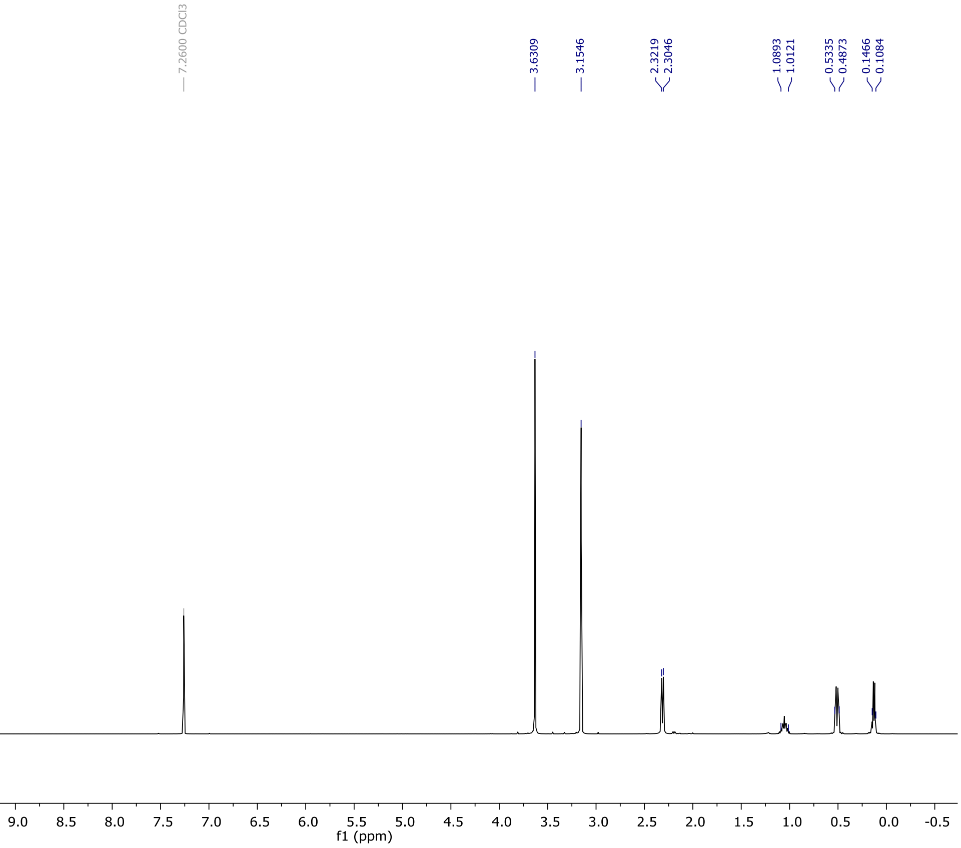

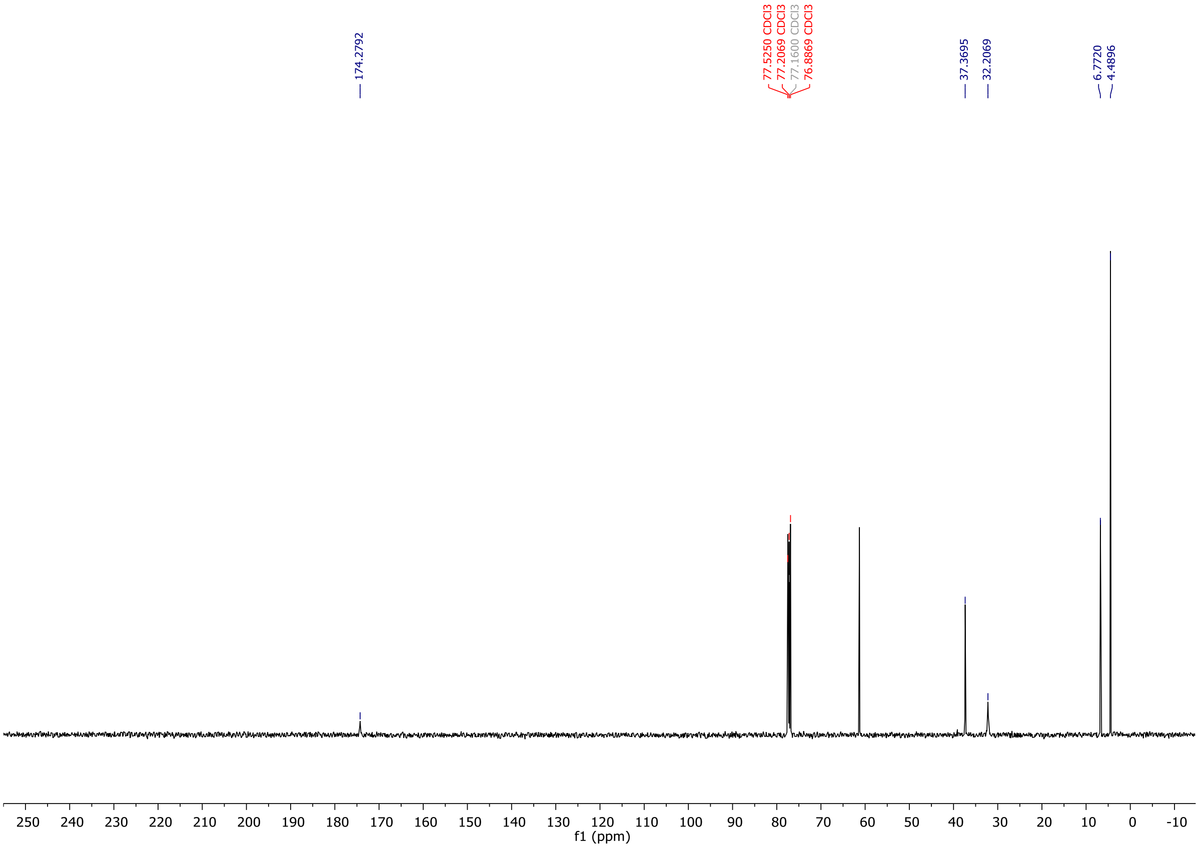

N-Benzyl-2-cyclopropylaniline (1a)

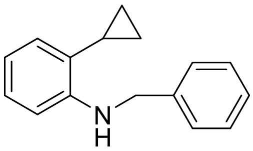

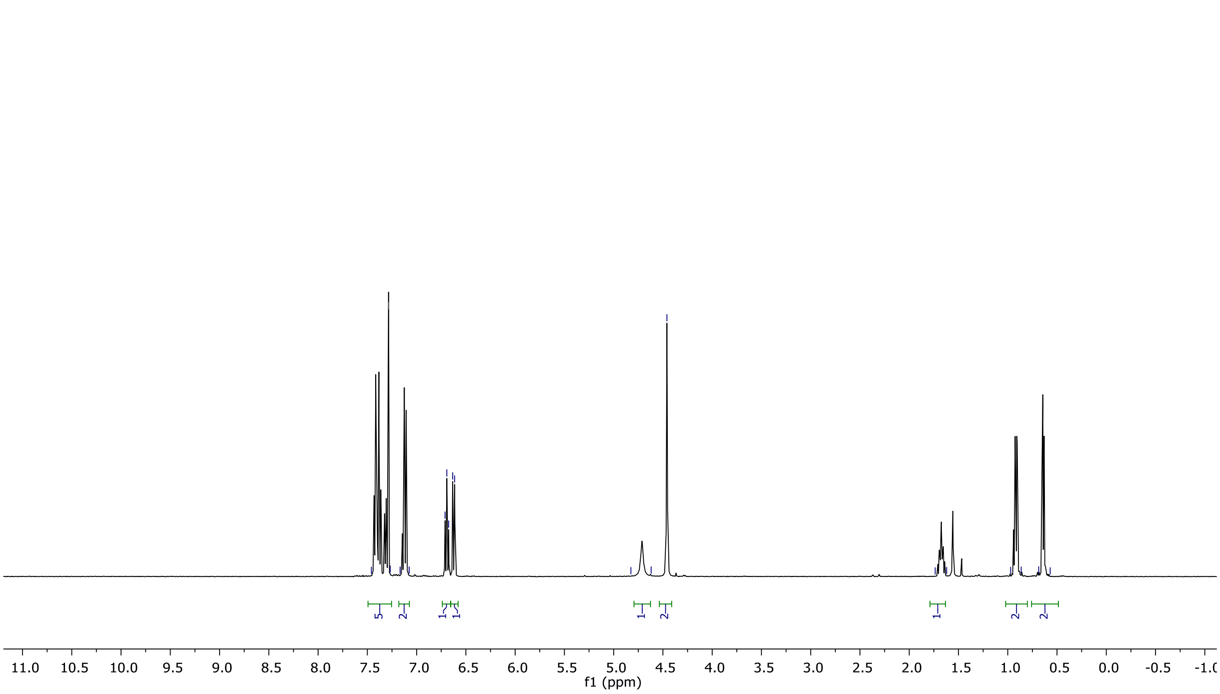

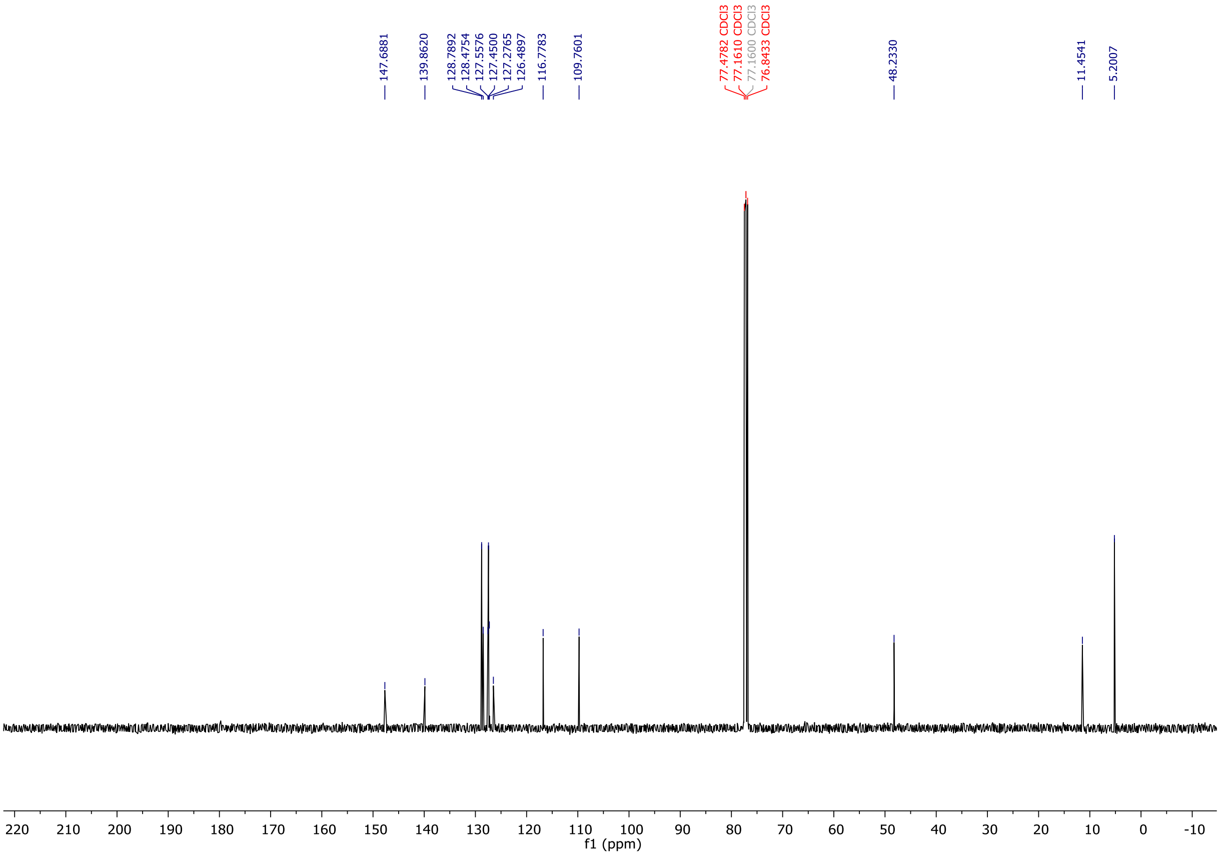

2-Cyclopropyl-*N*-phenylaniline (1b)

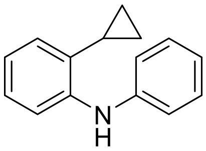

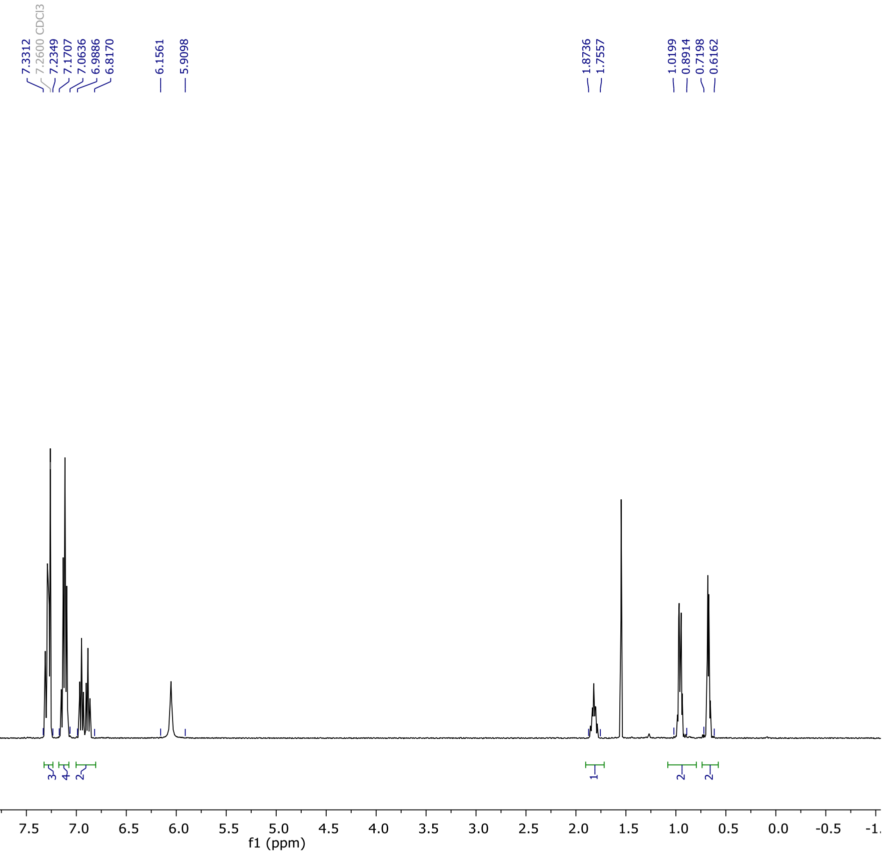

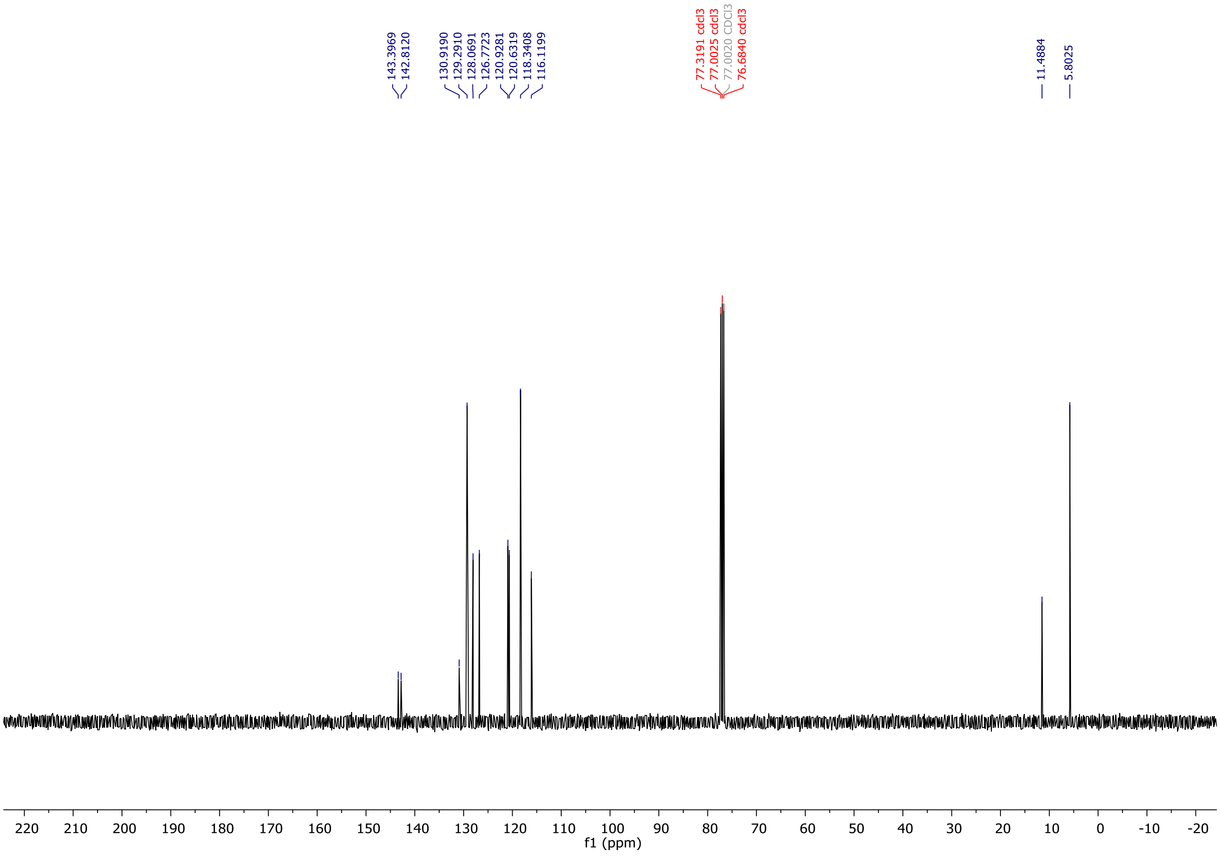

2-Cyclopropyl-*N*-(4-methoxyphenyl)aniline (1c)

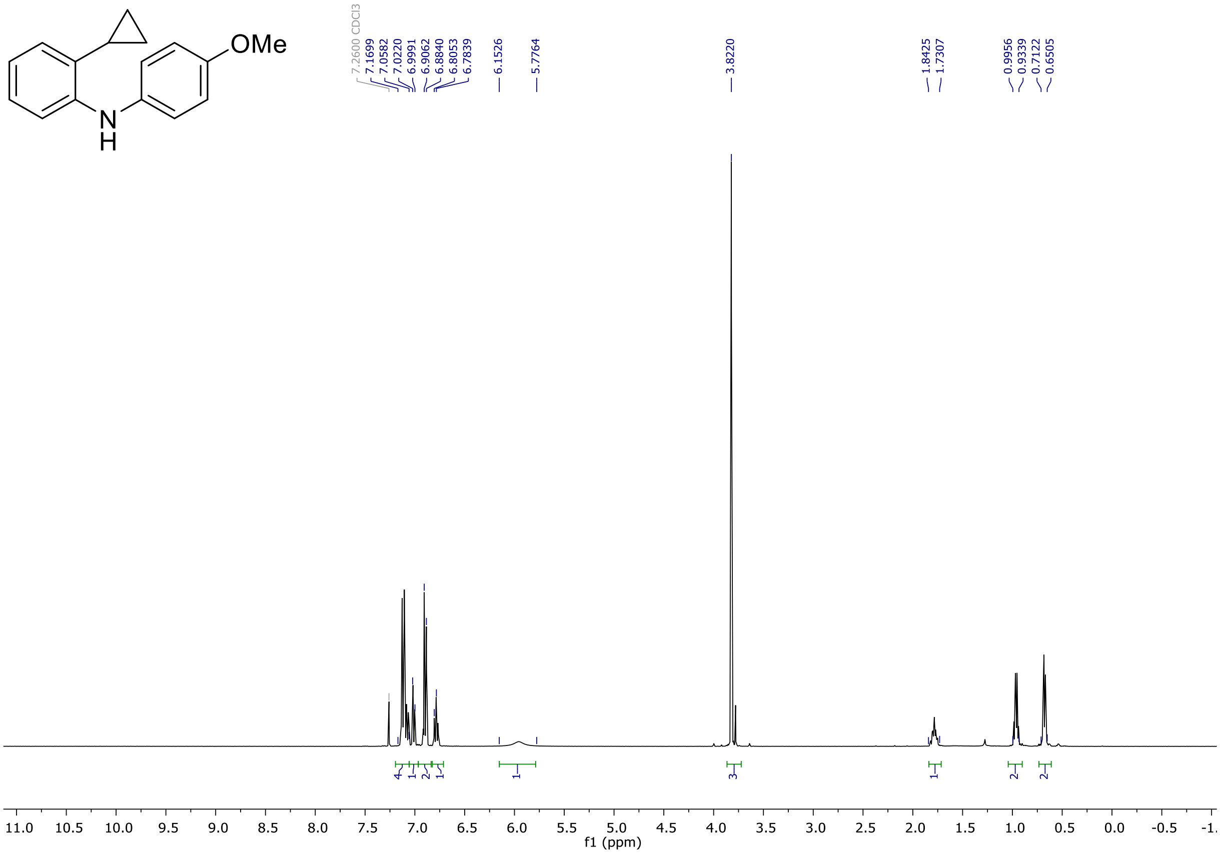

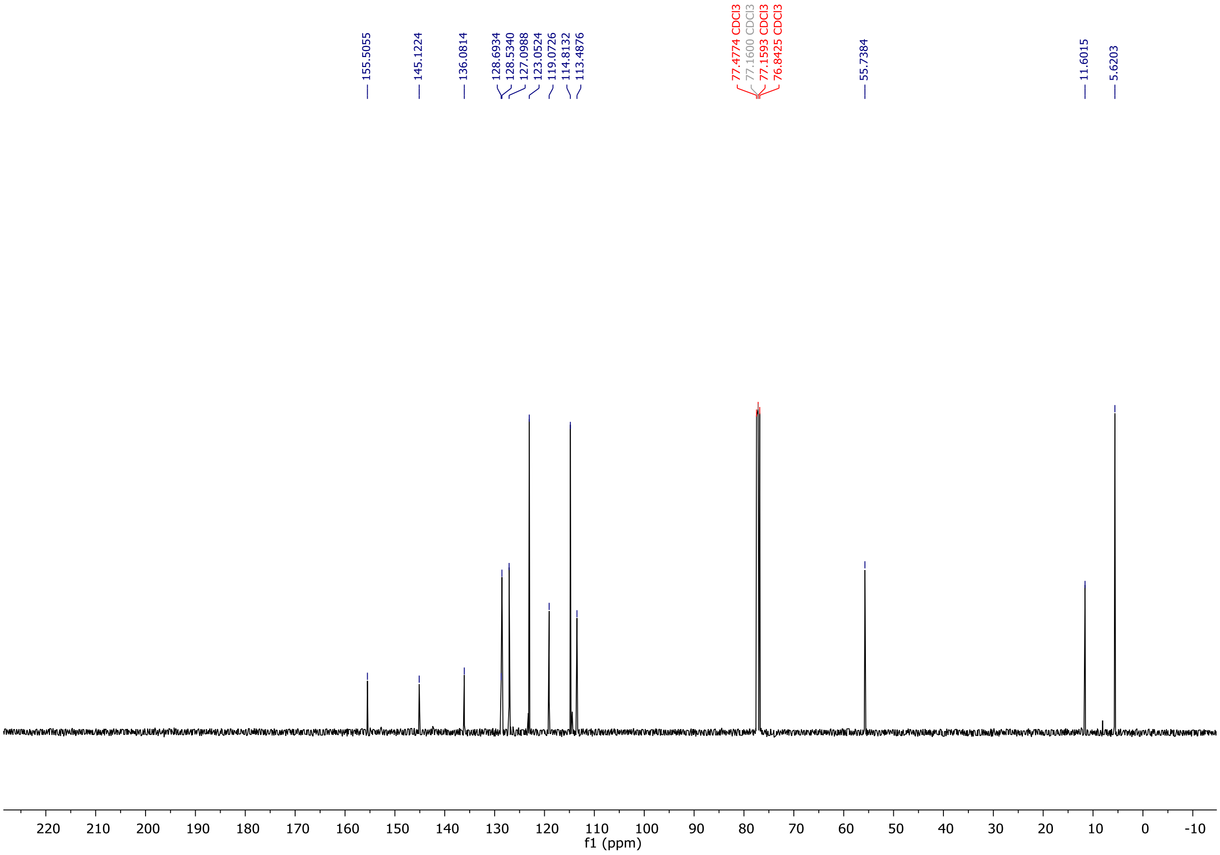

2-Cyclopropylaniline (1d)

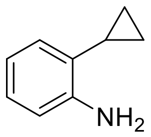

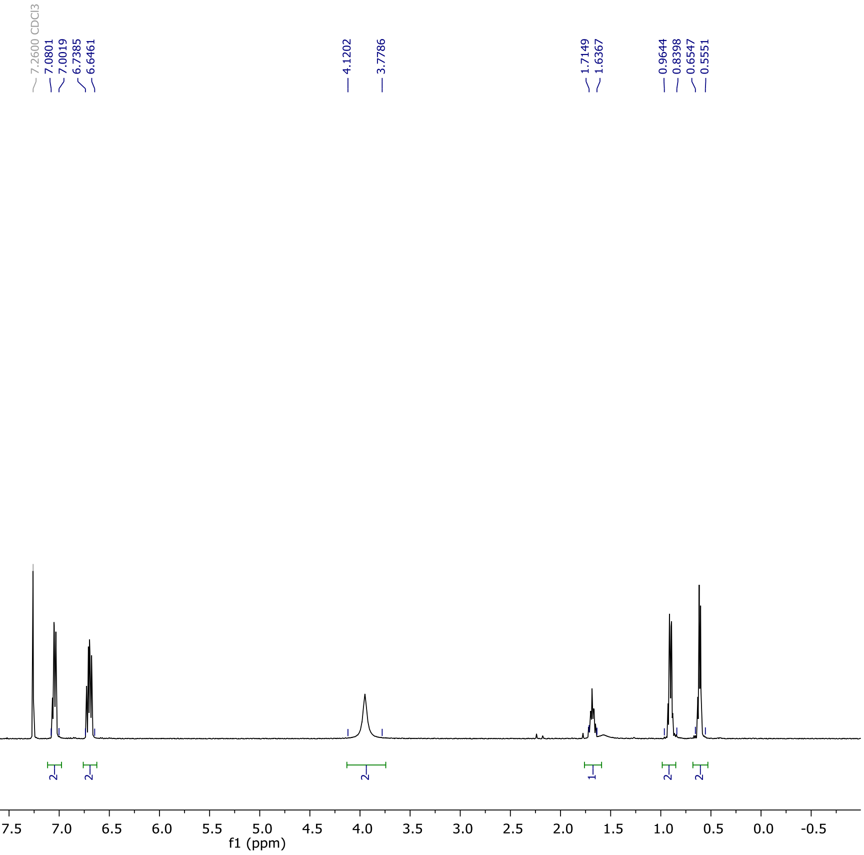

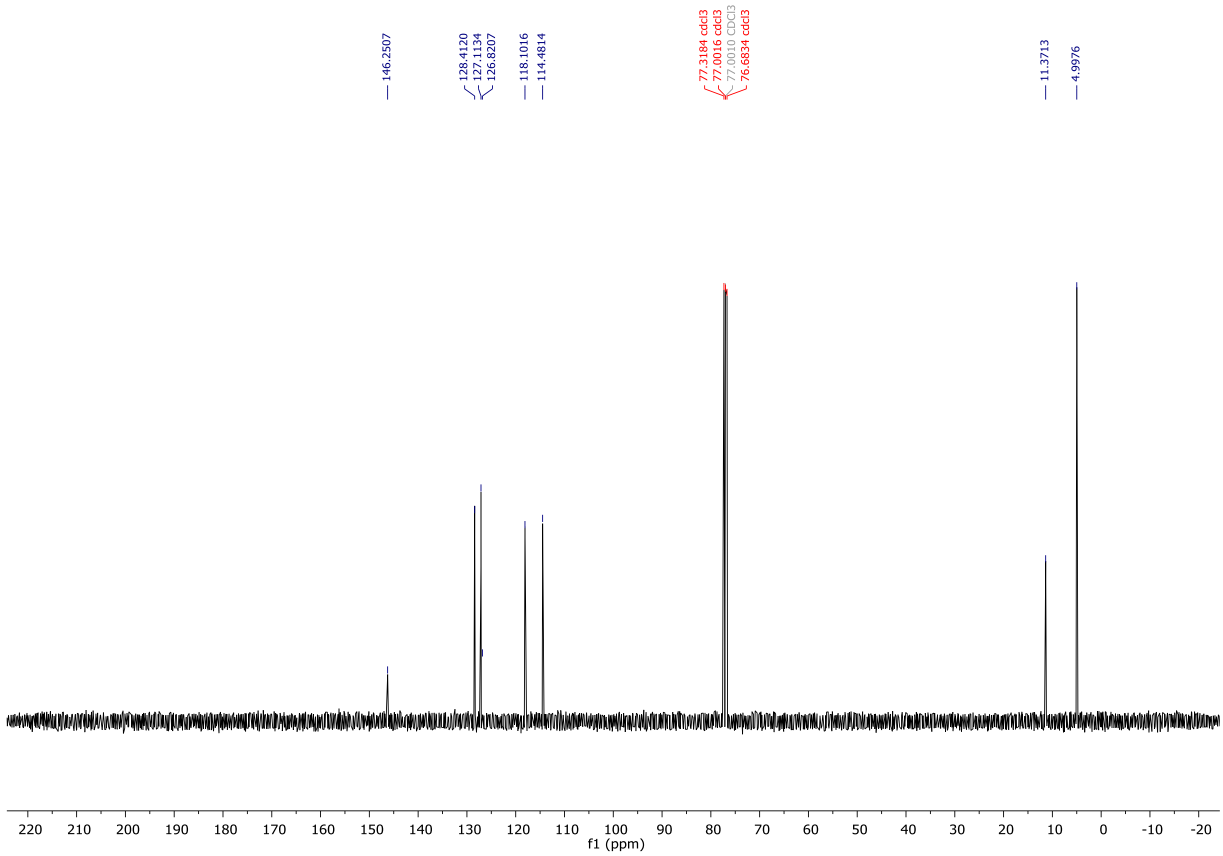

2-Cyclopropyl-5-methoxyaniline (1e)

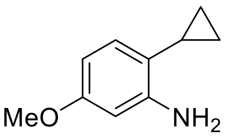

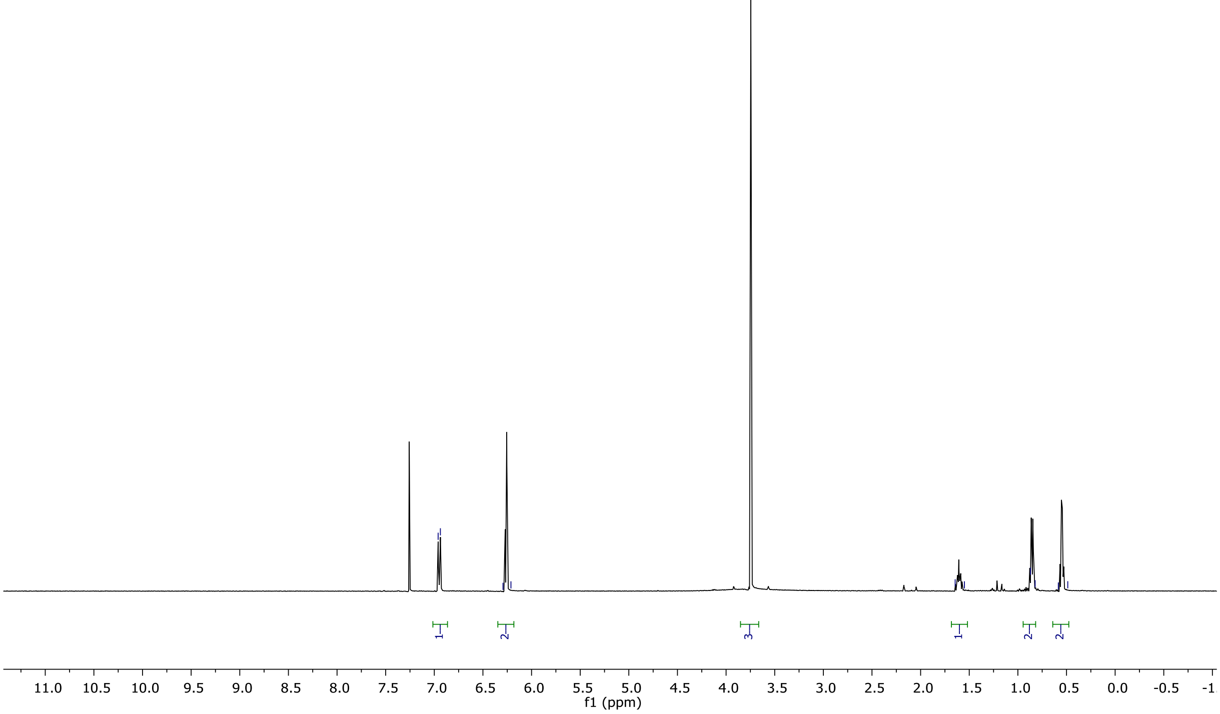

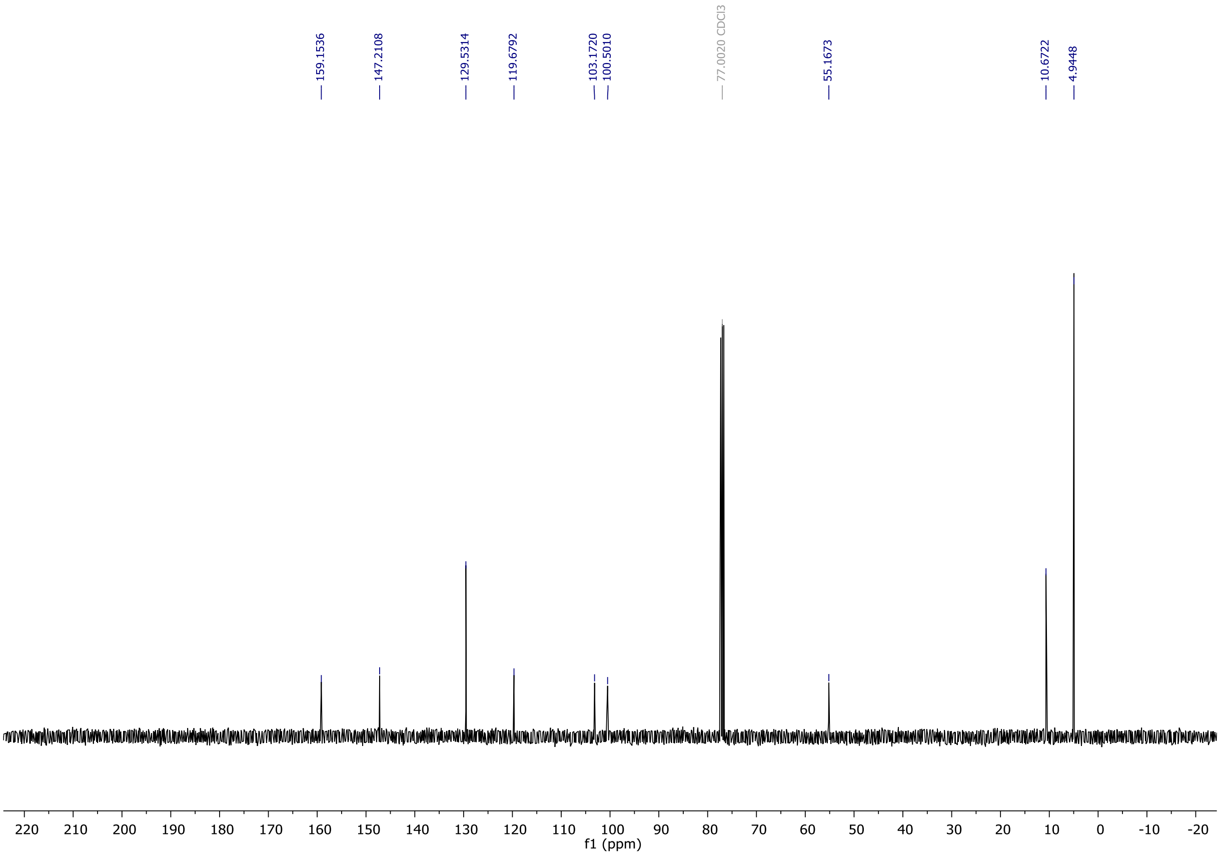

2-Cyclopropyl-5-(trifluoromethyl)aniline (1f)

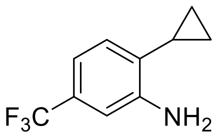

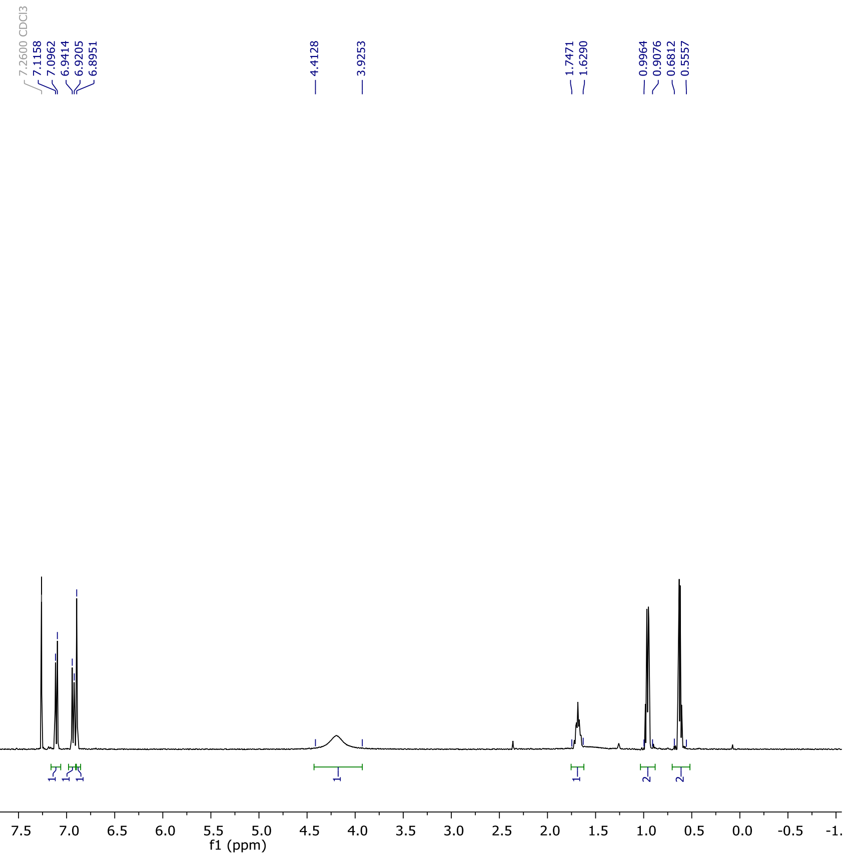

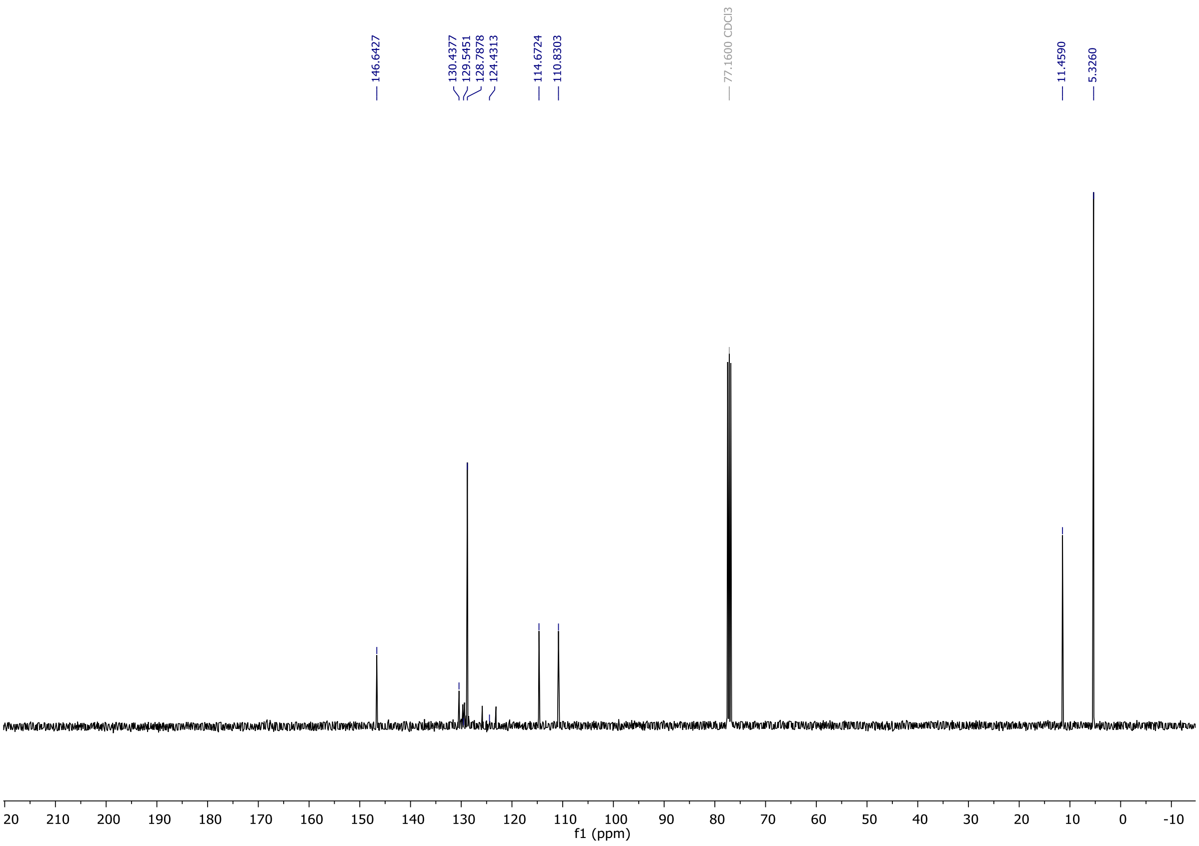

2-Cyclopropylpyridin-3-amine (1g)

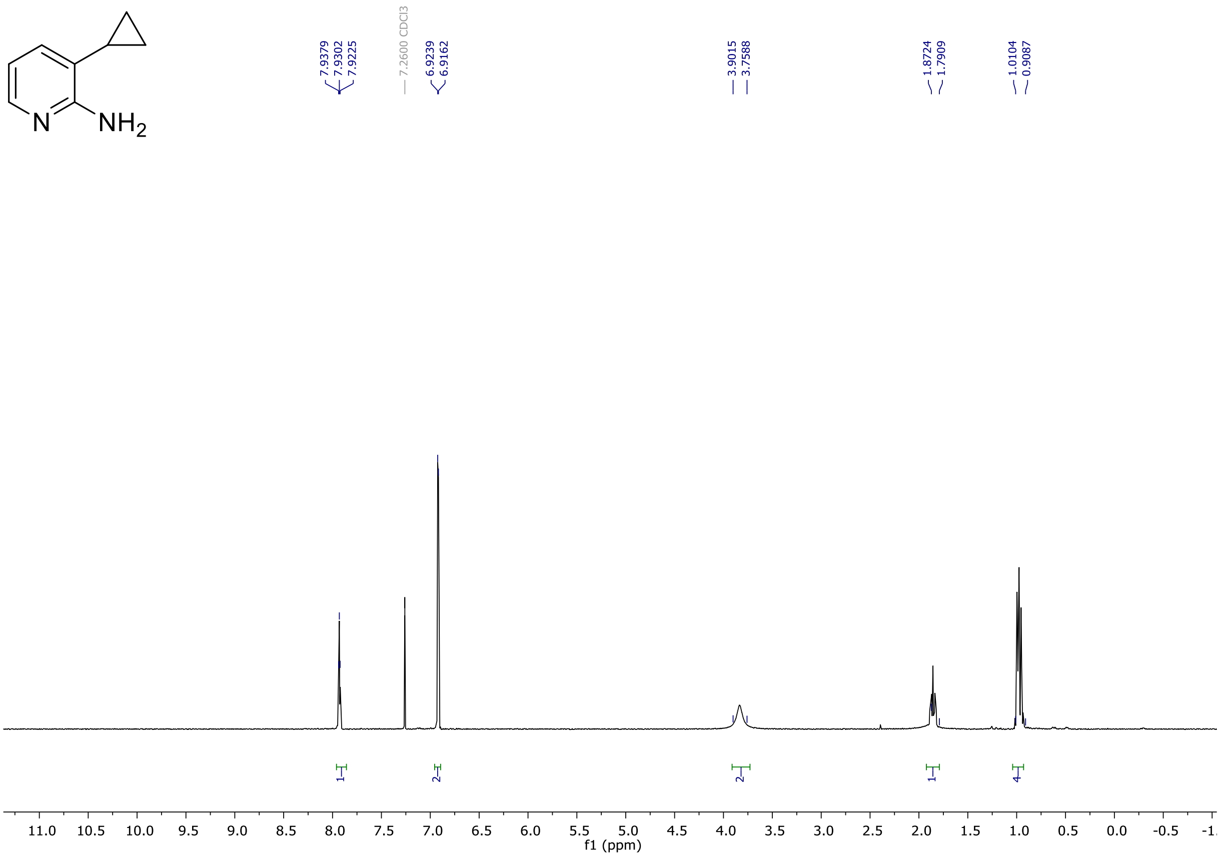

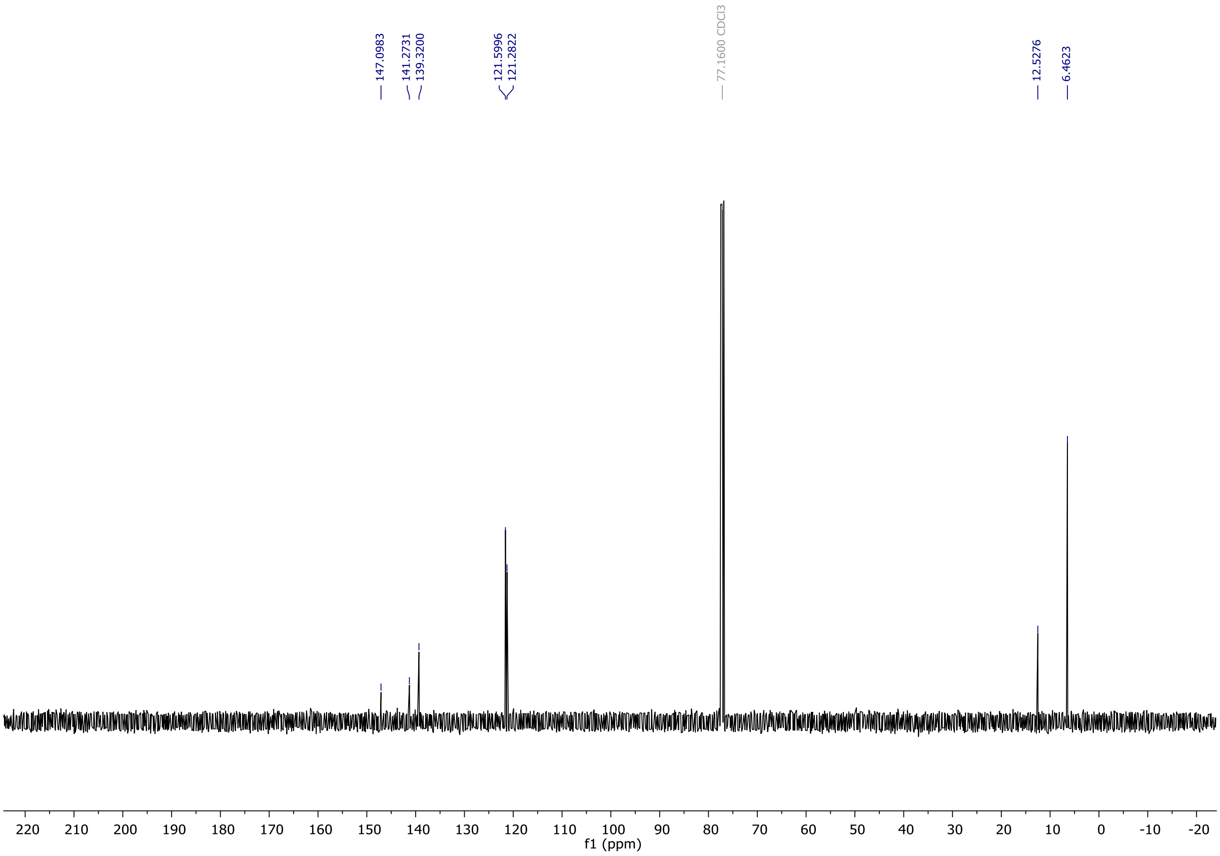

(*rac*)-(*cis*)-2-(2-Methylcyclopropyl)aniline (*cis*-1h)

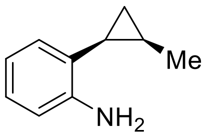

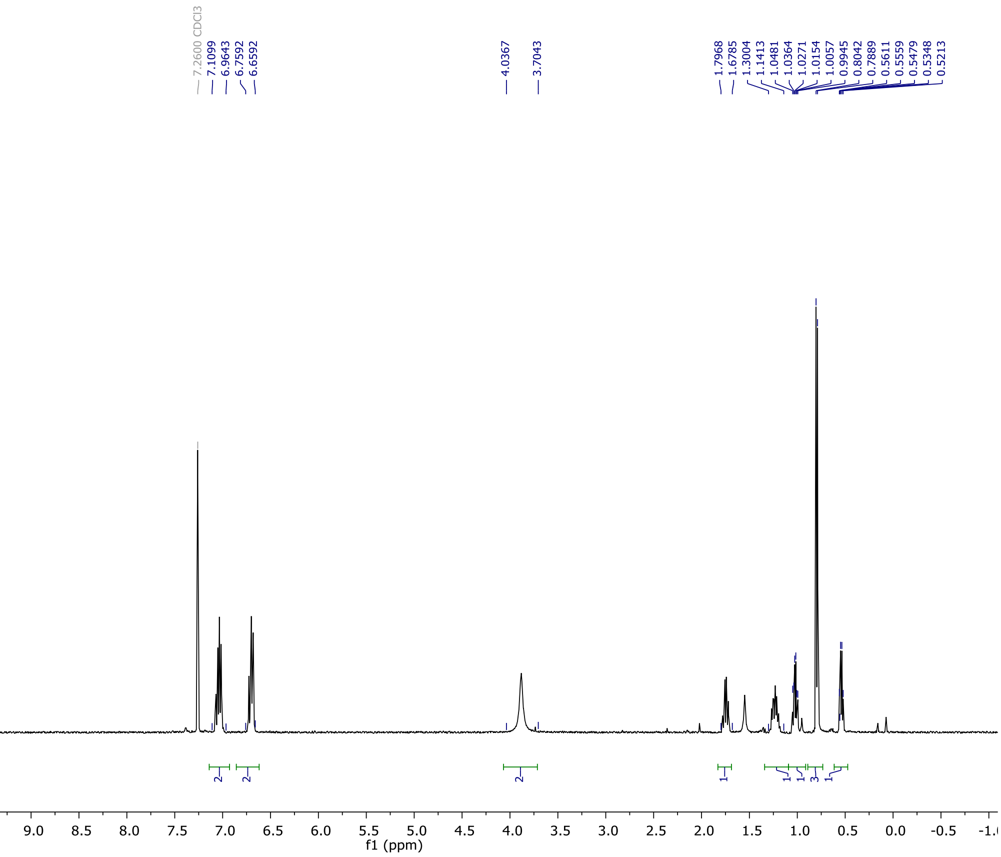

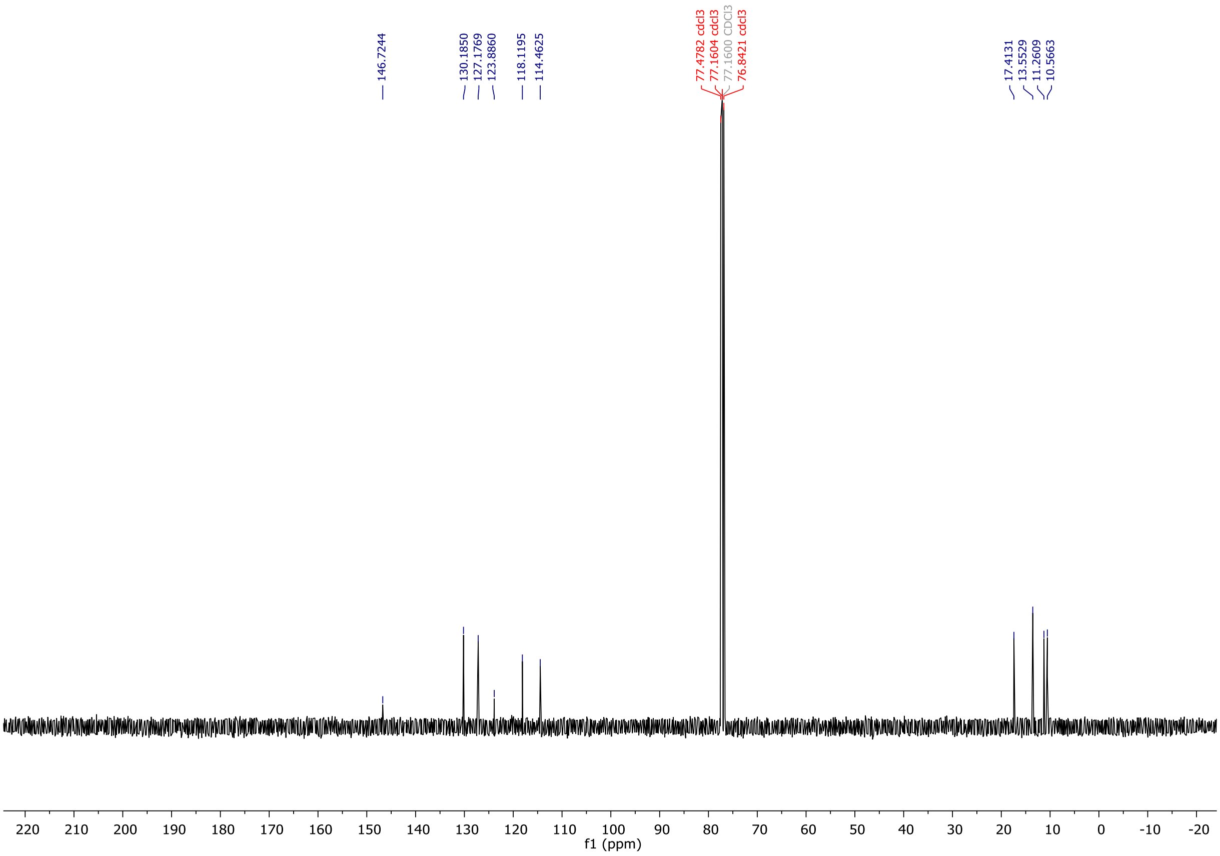

(*rac*)-(*trans*)-2-(2-Methylcyclopropyl)aniline (*trans*-1h)

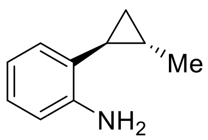

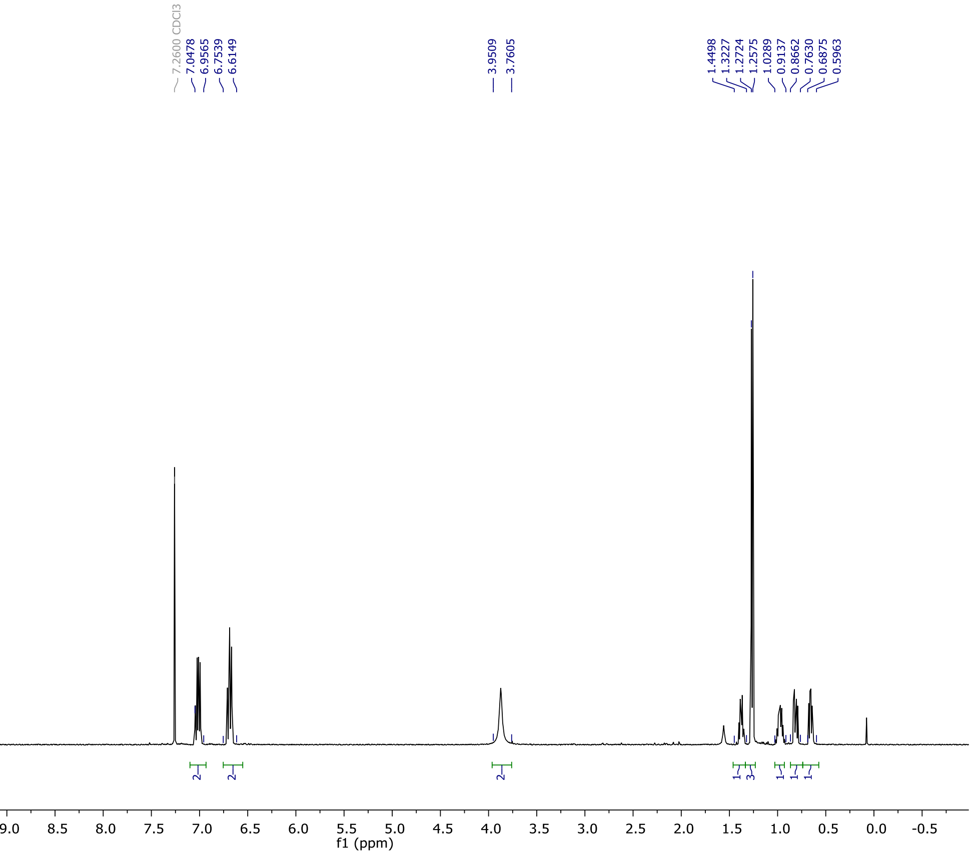

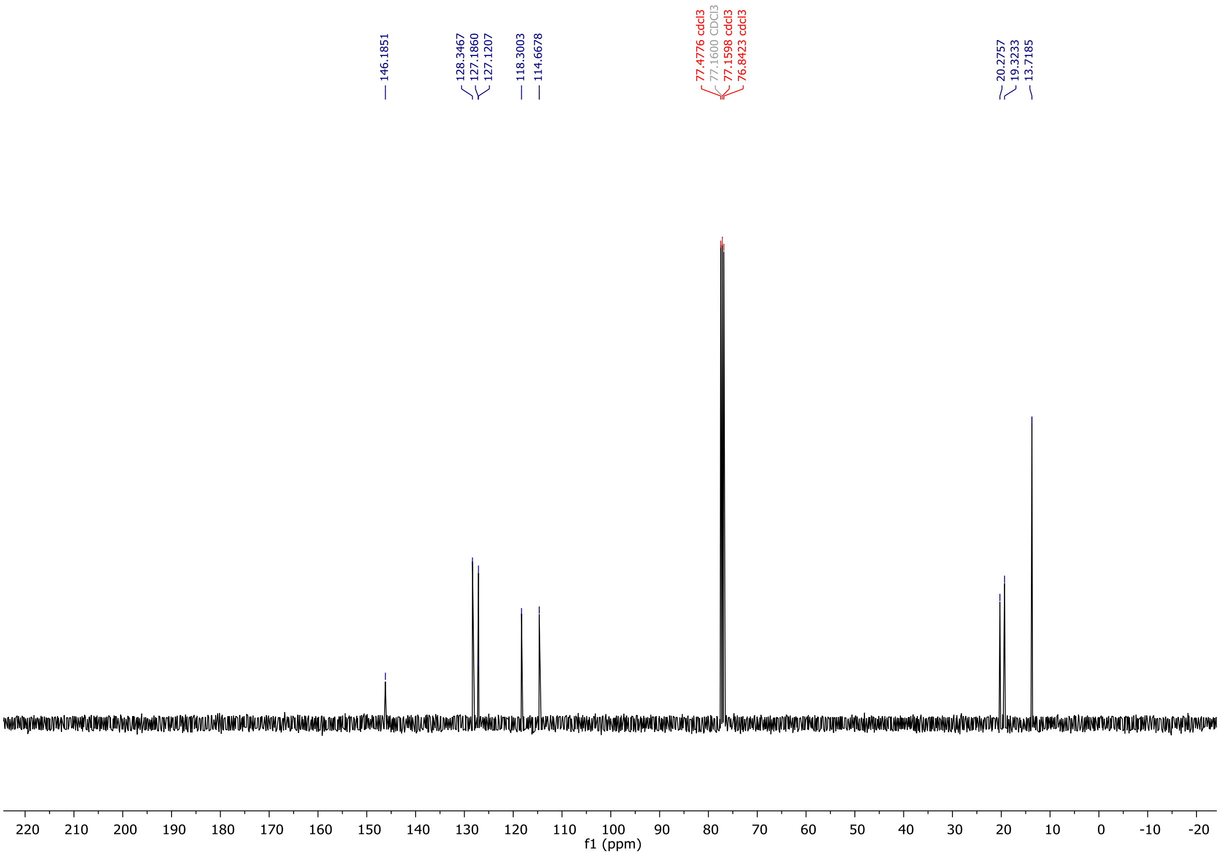

N-(2-Cyclopropylethyl)aniline (1i)

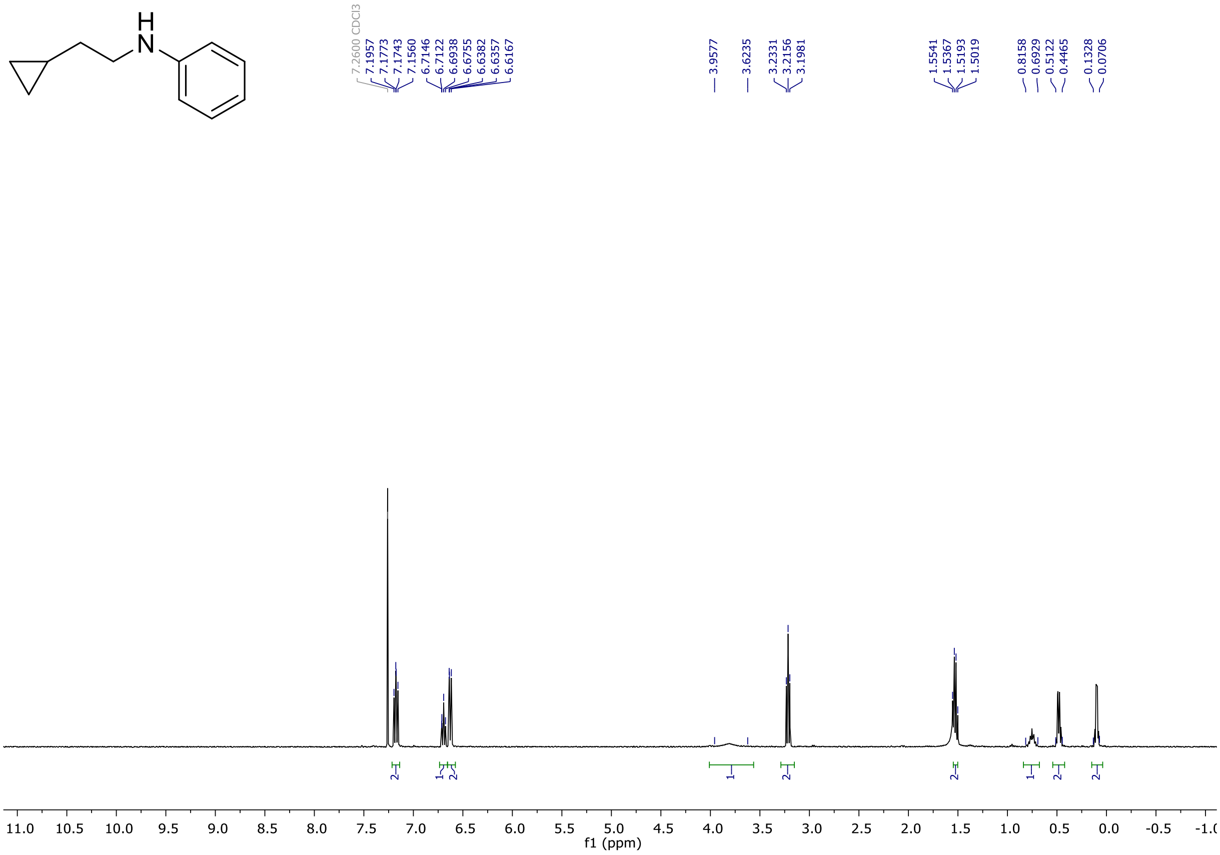

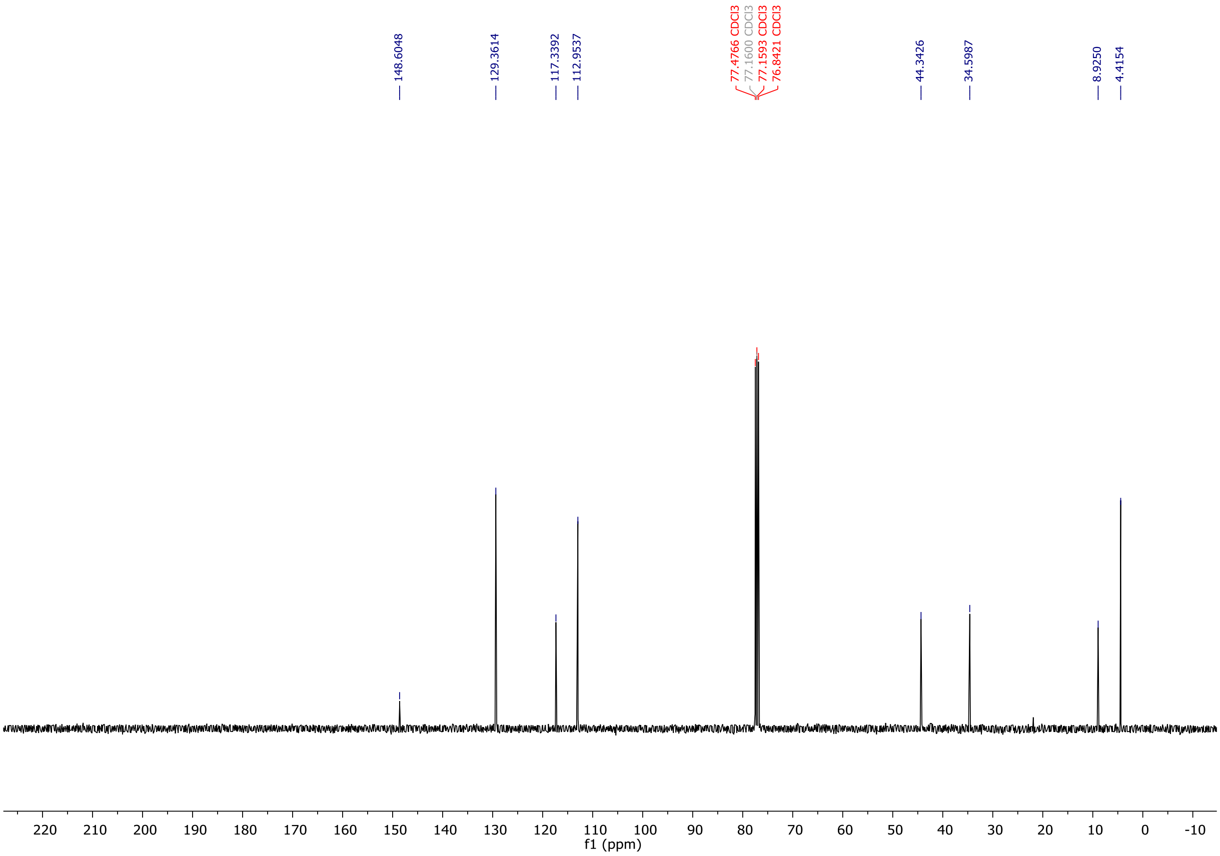

N-(2-Cyclopropyl-2-phenylethyl)aniline (1j)

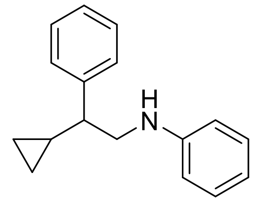

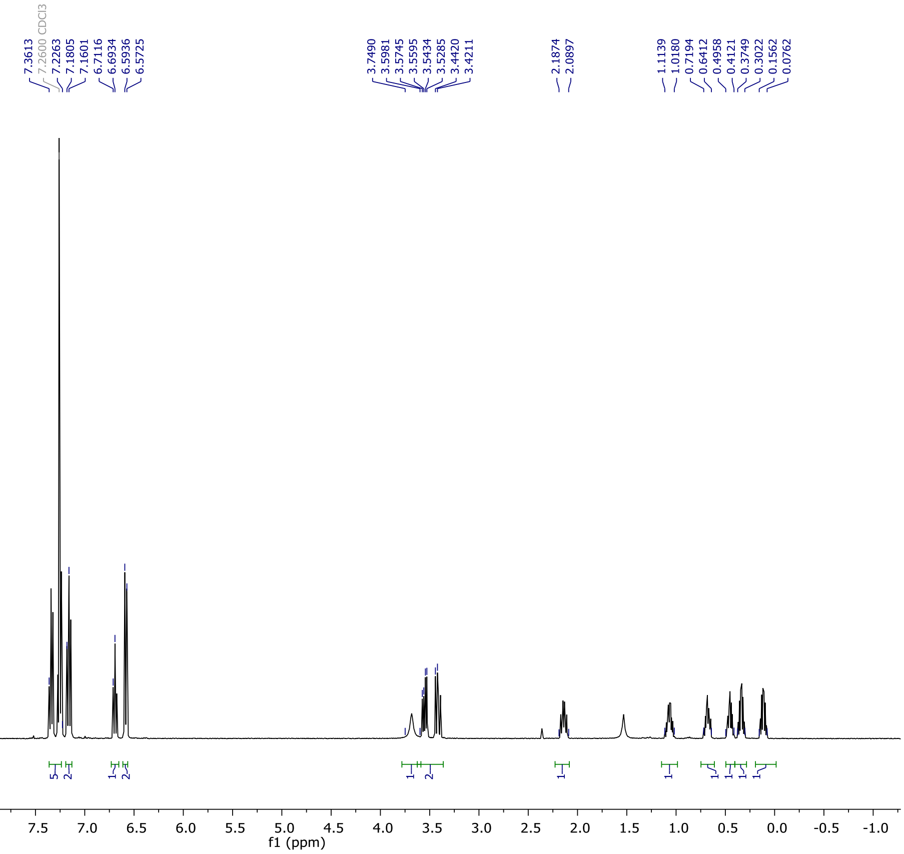

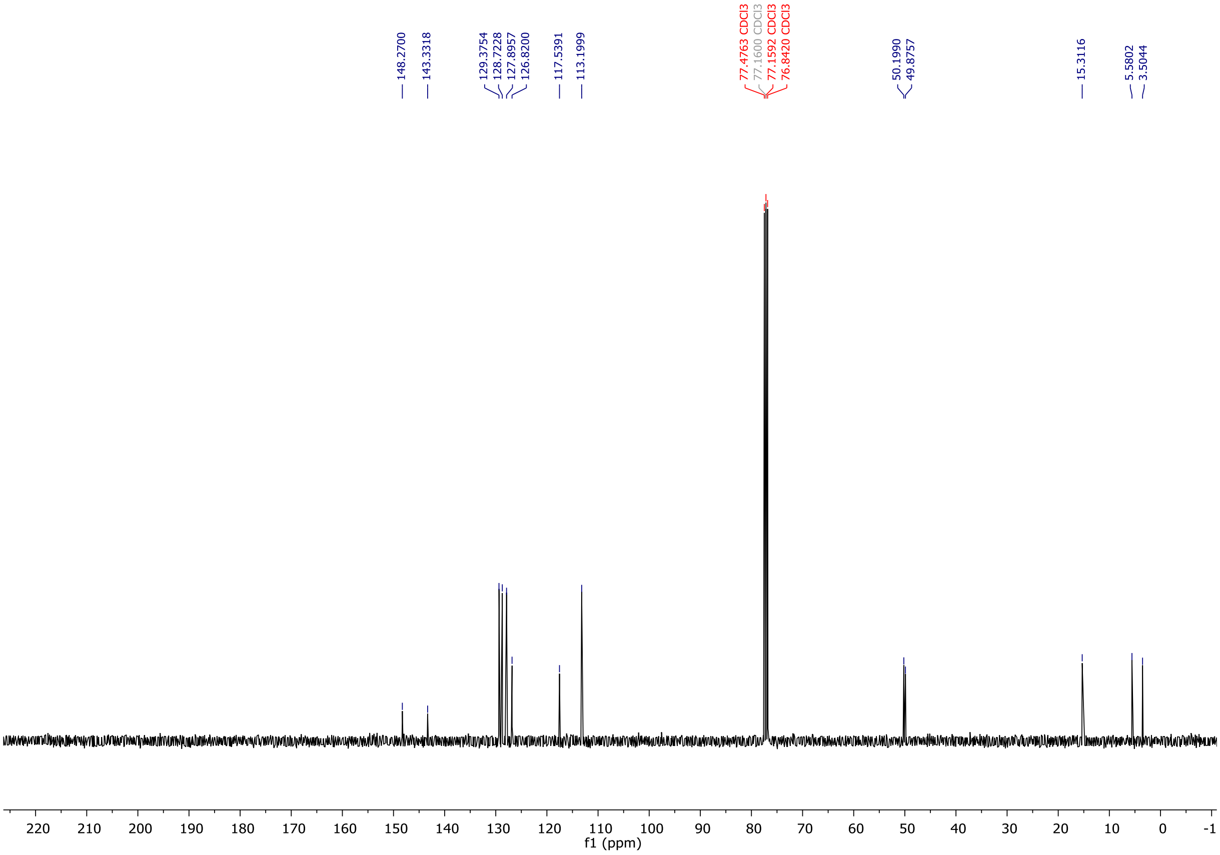

N-(1-Cyclopropylbutan-2-yl)aniline (1k)

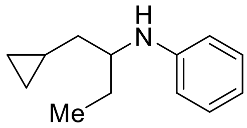

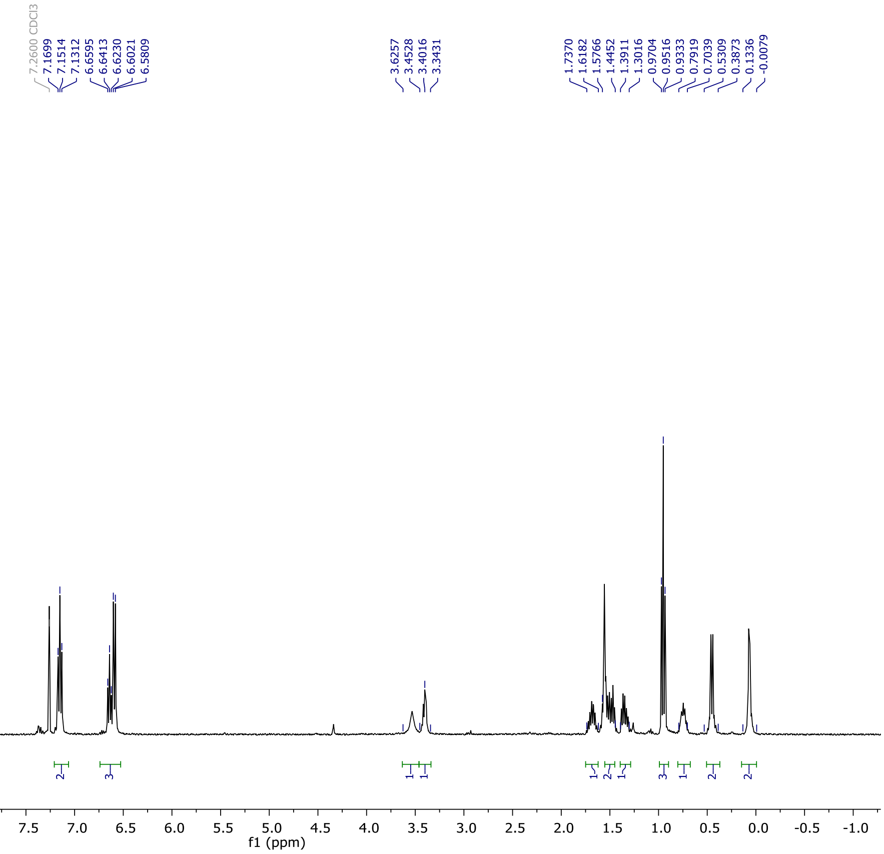

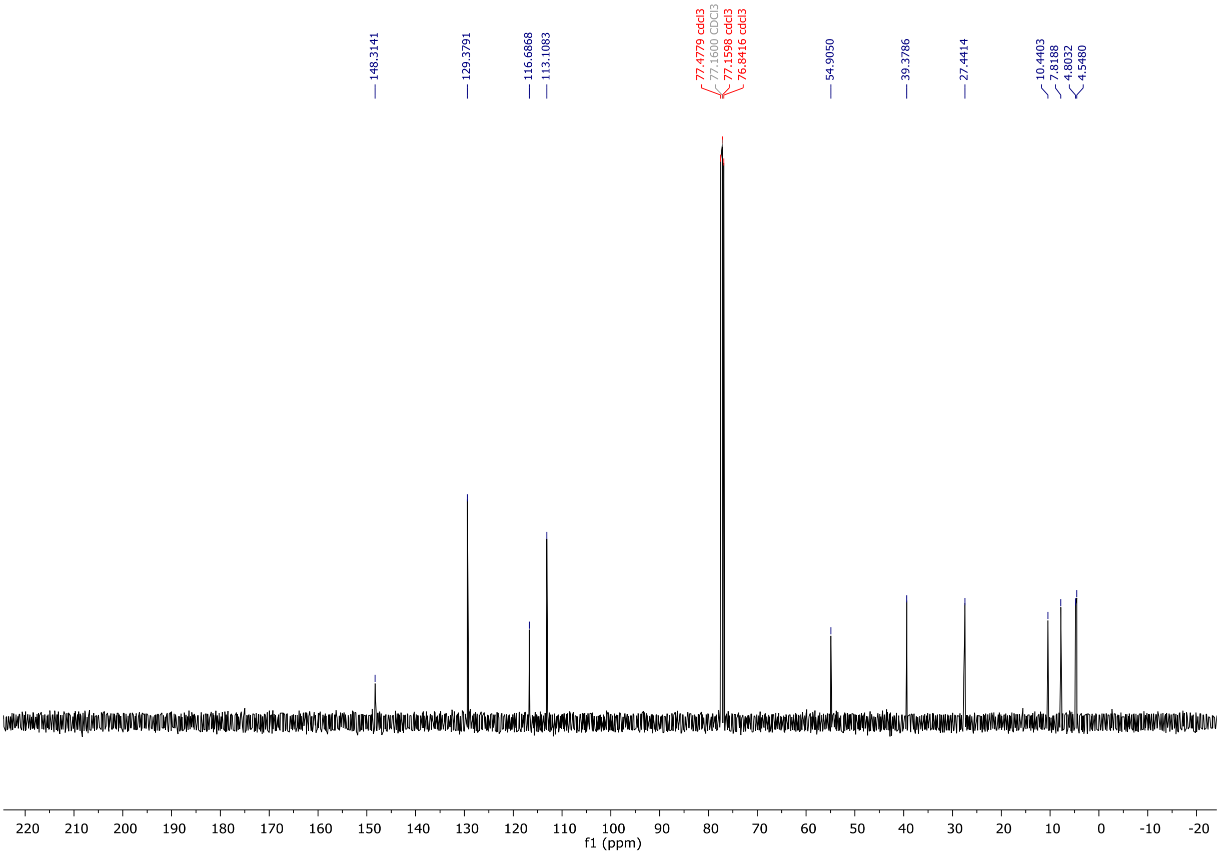

N-(2-Cyclopropyl-1-phenylethyl)aniline (1l)

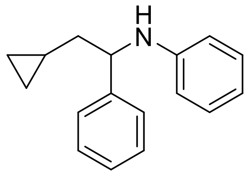

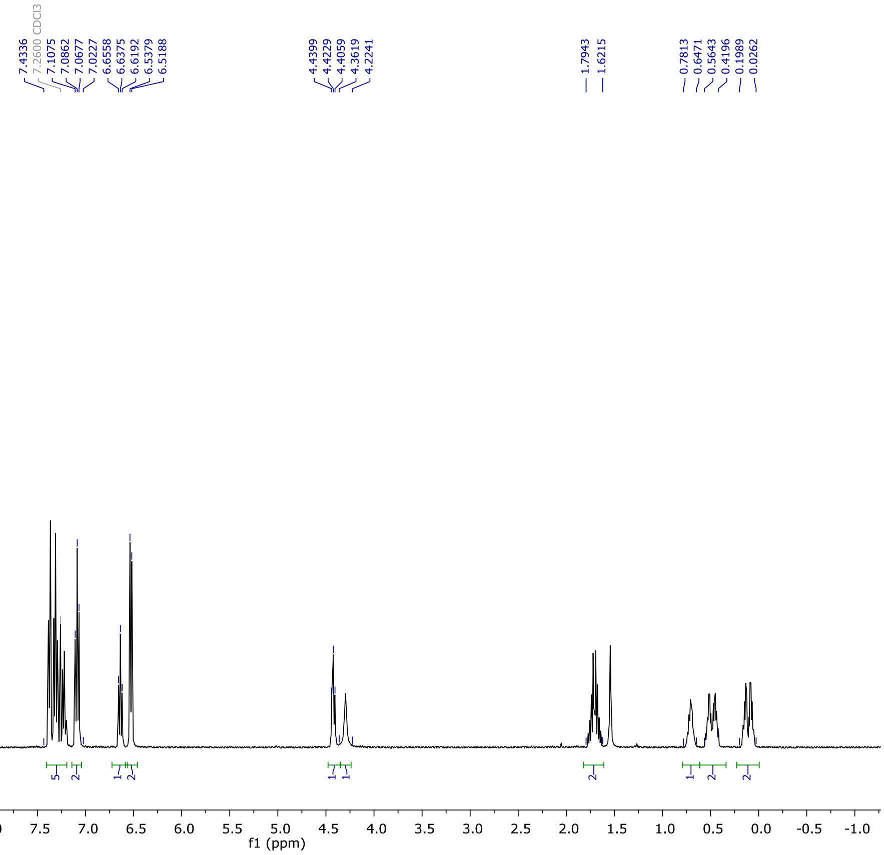

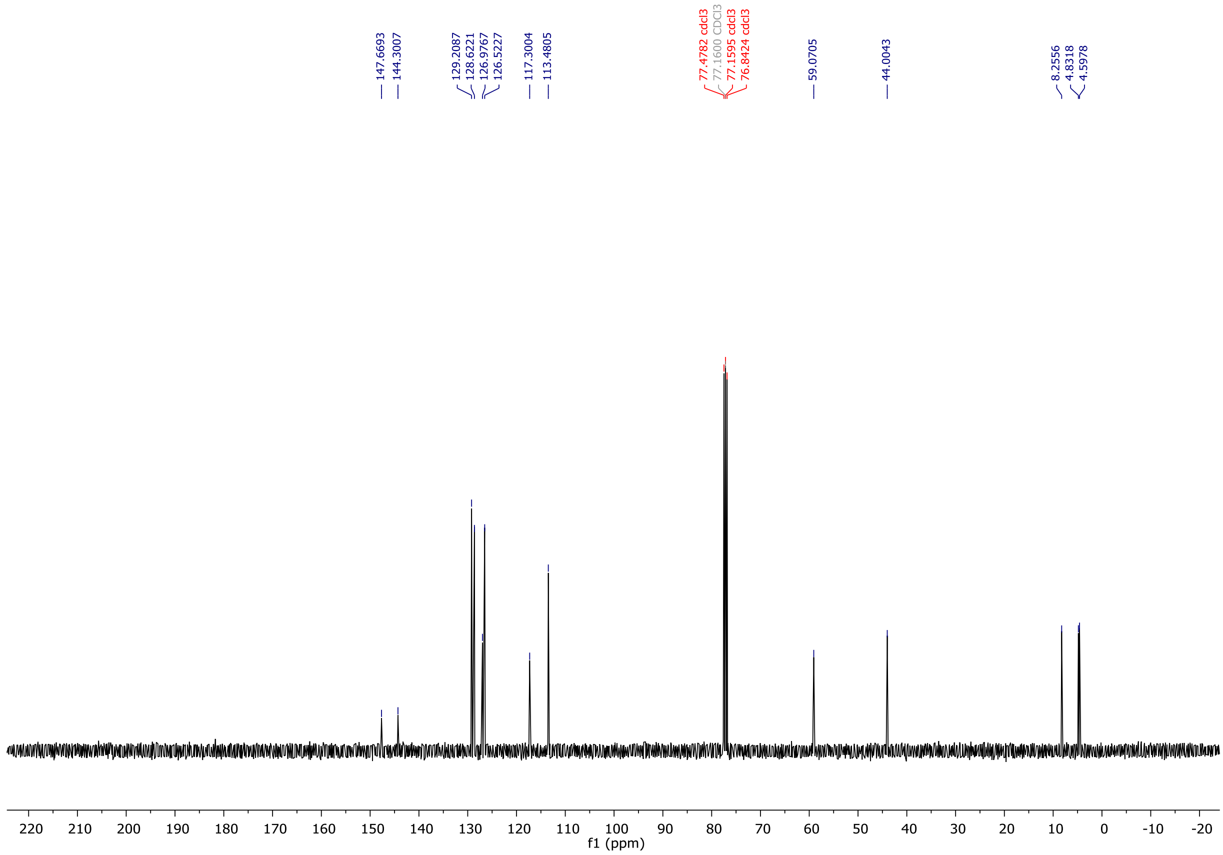

N-(2-Cyclopropyl-1-(naphthalen-2-yl)ethyl)aniline (1m)

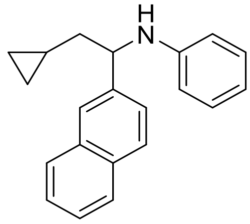

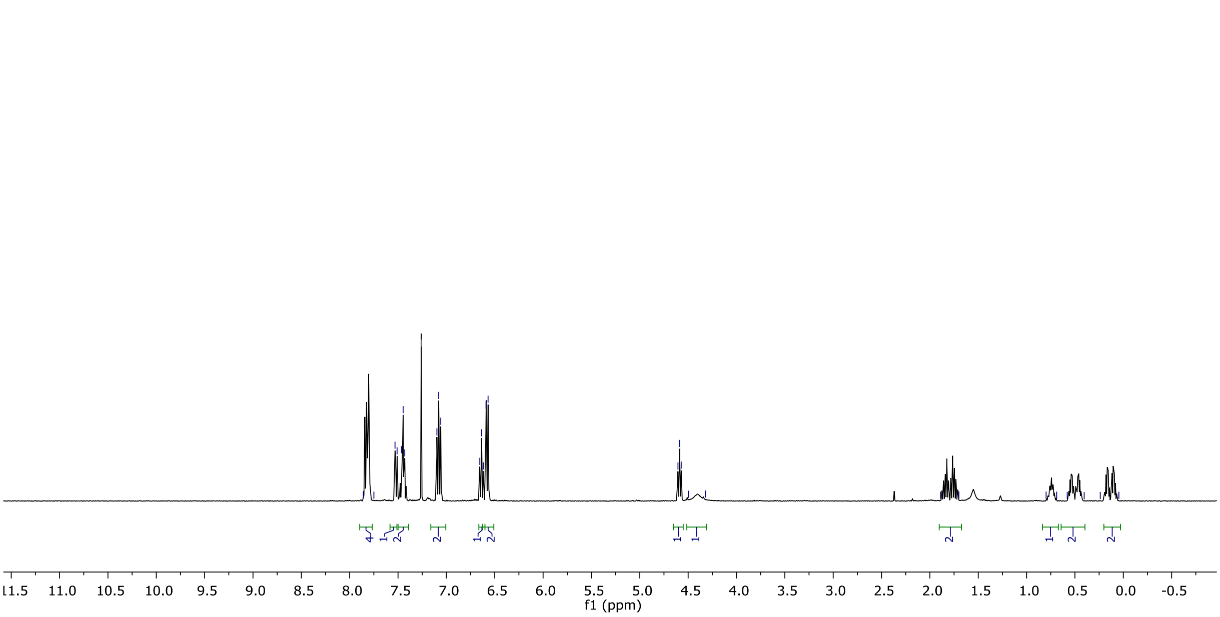

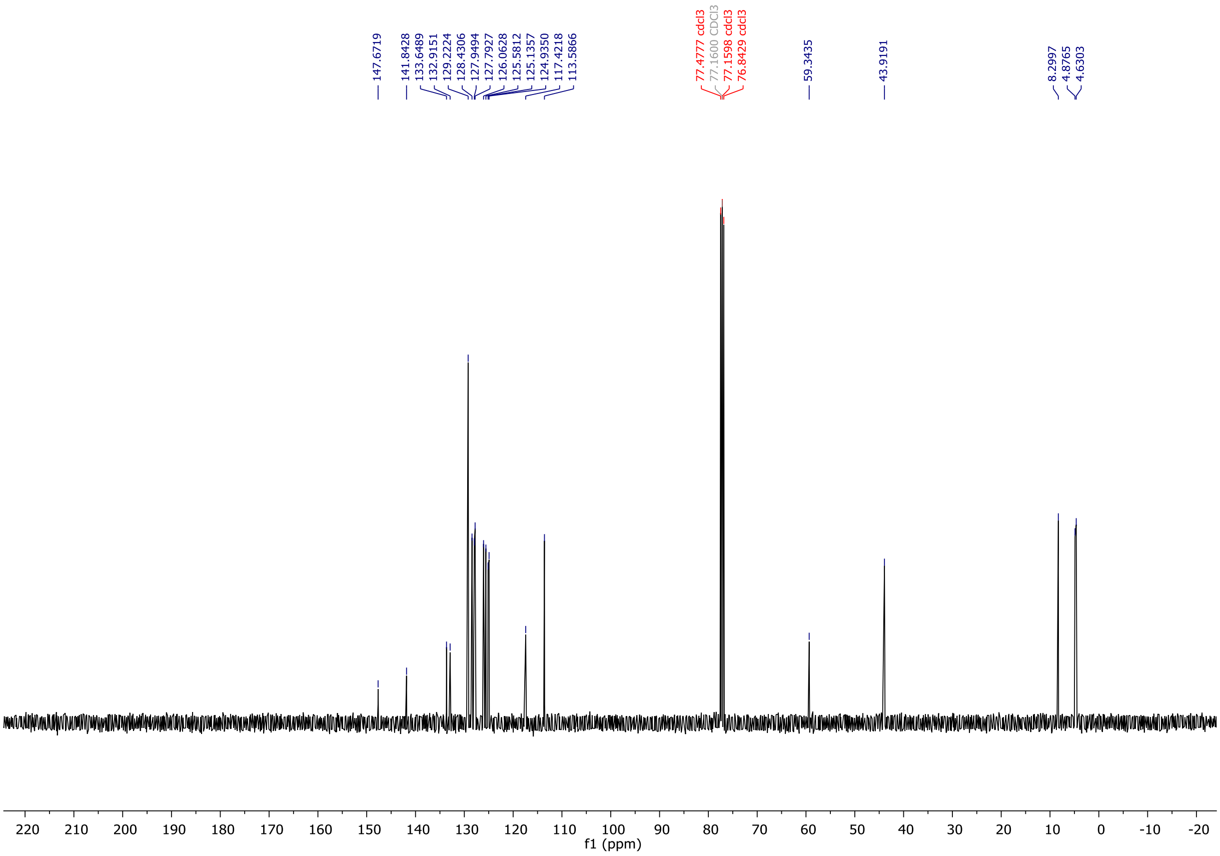

N-(2-Cyclopropyl-1-(thiophen-3-yl)ethyl)aniline (1n)

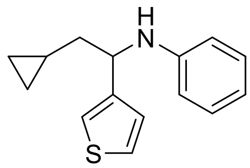

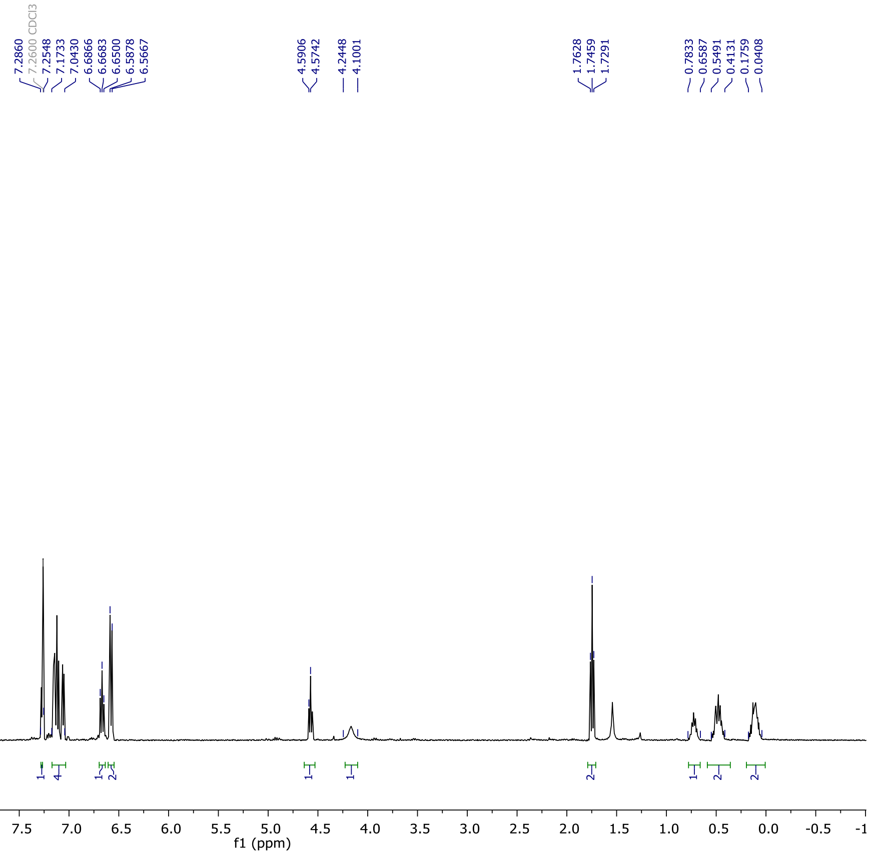

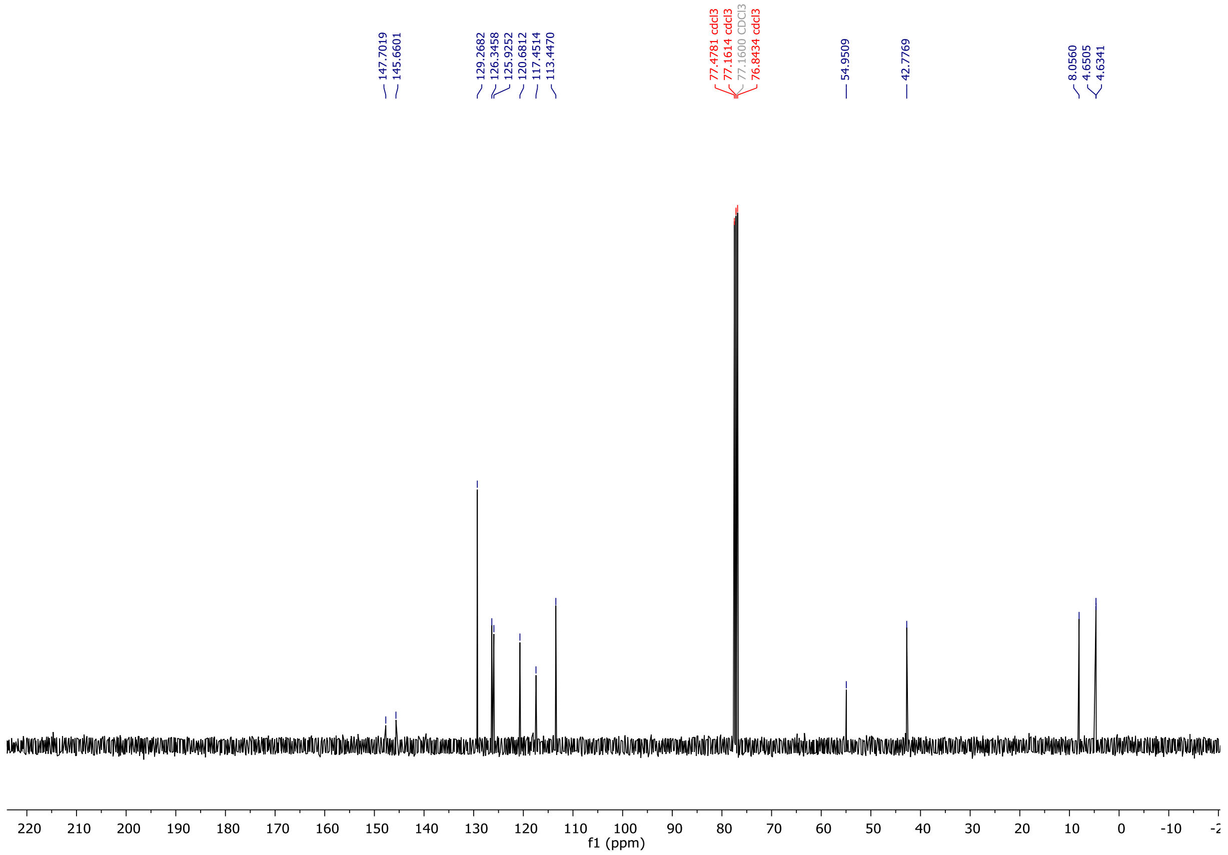

N-(1,2-Dicyclopropylethyl)aniline (1o)

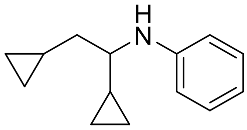

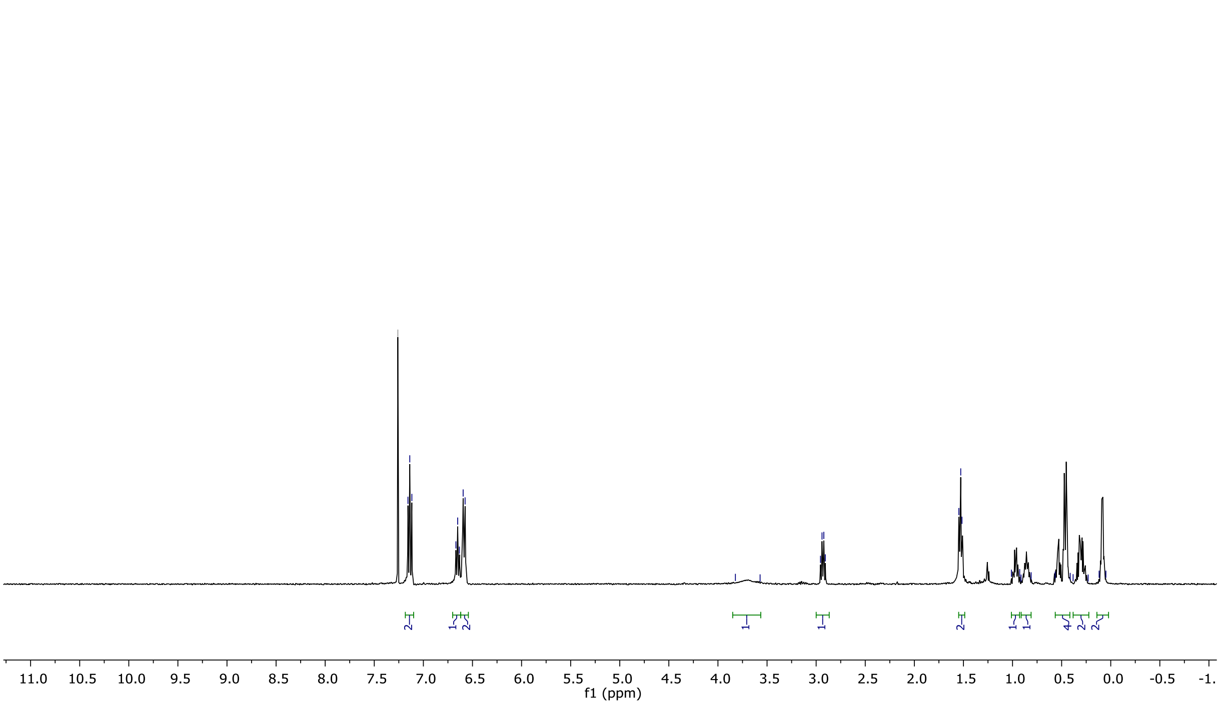

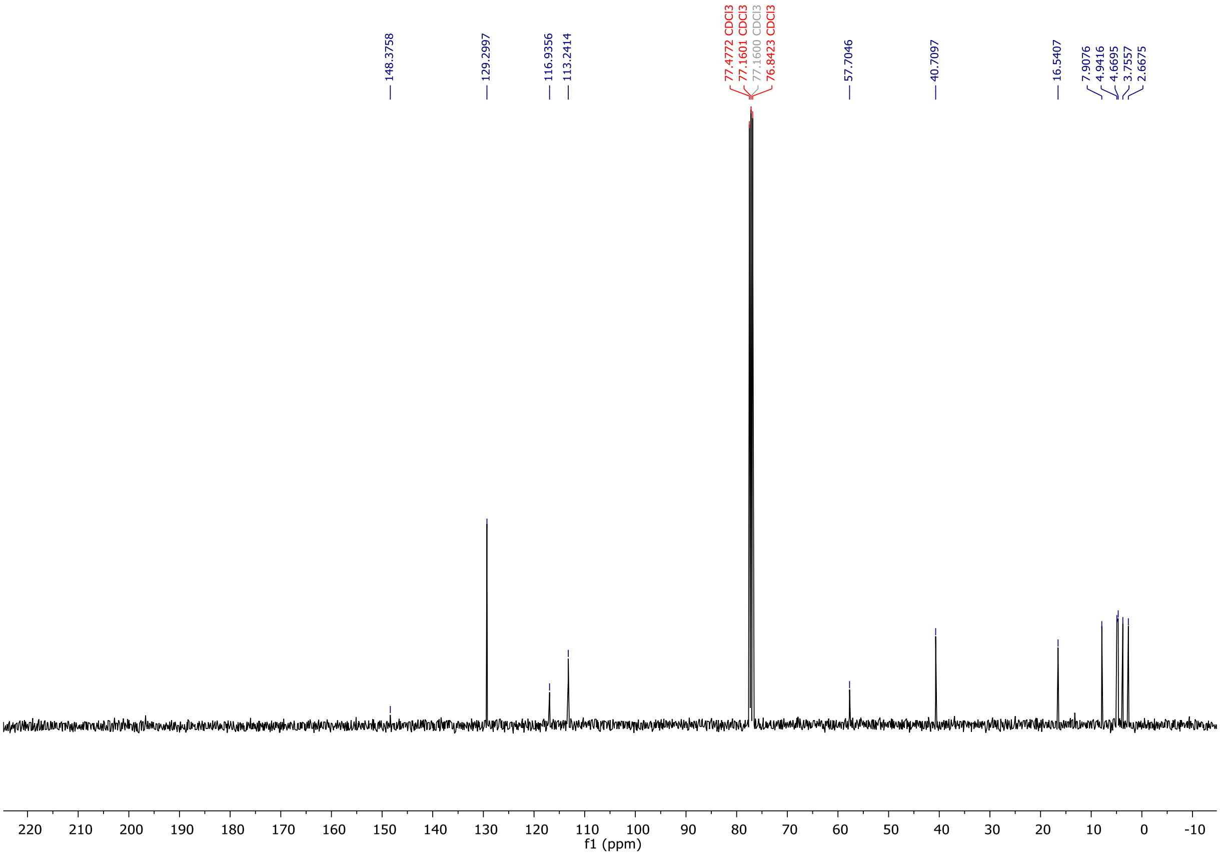

(*rac*)-(*cis*)-*N*-(2-Cyclopropylcyclopentyl)aniline (1p)

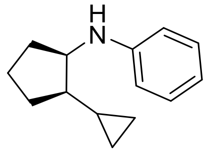

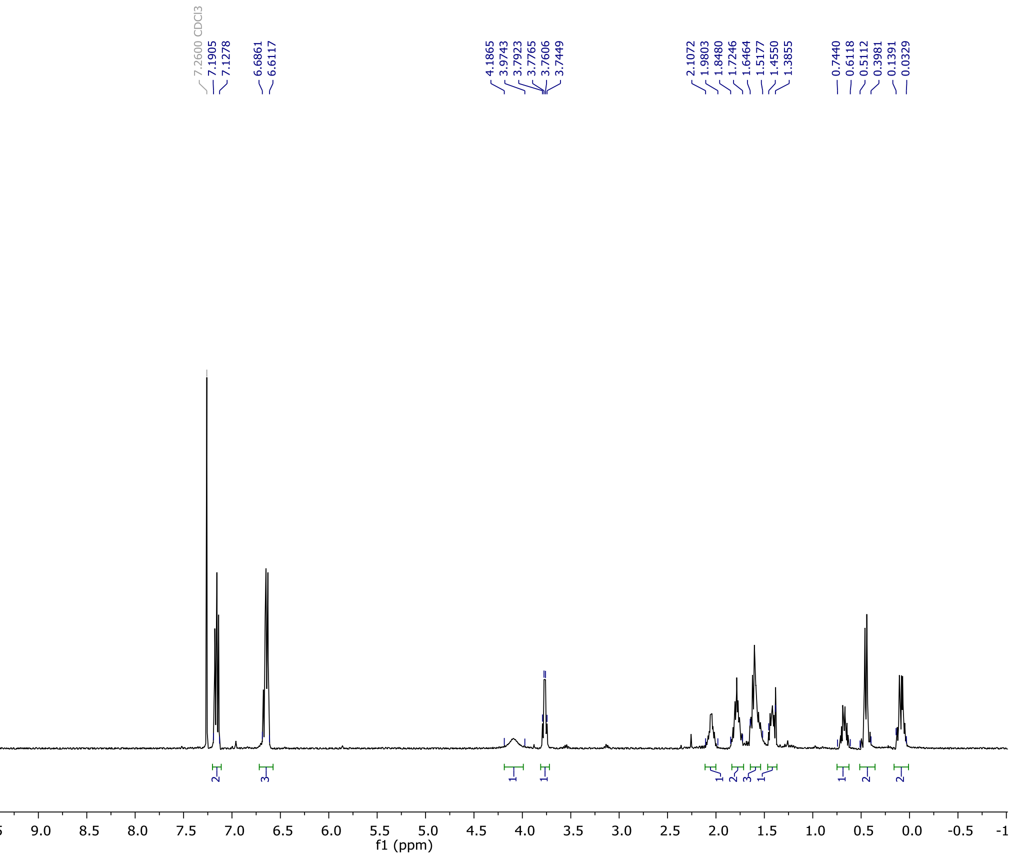

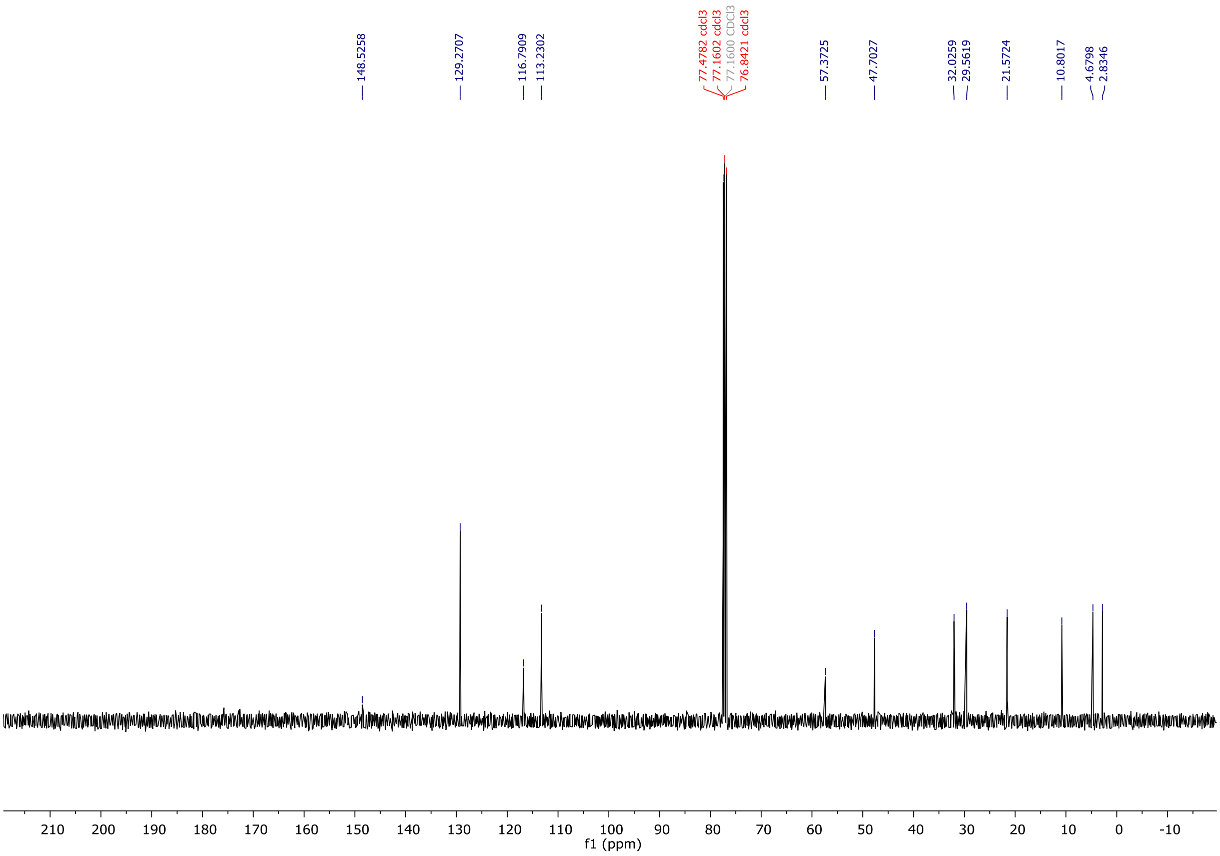

(*rac*)-(*trans*)-*N*-(2-(2-Methylcyclopropyl)ethyl)aniline (1q)

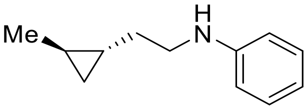

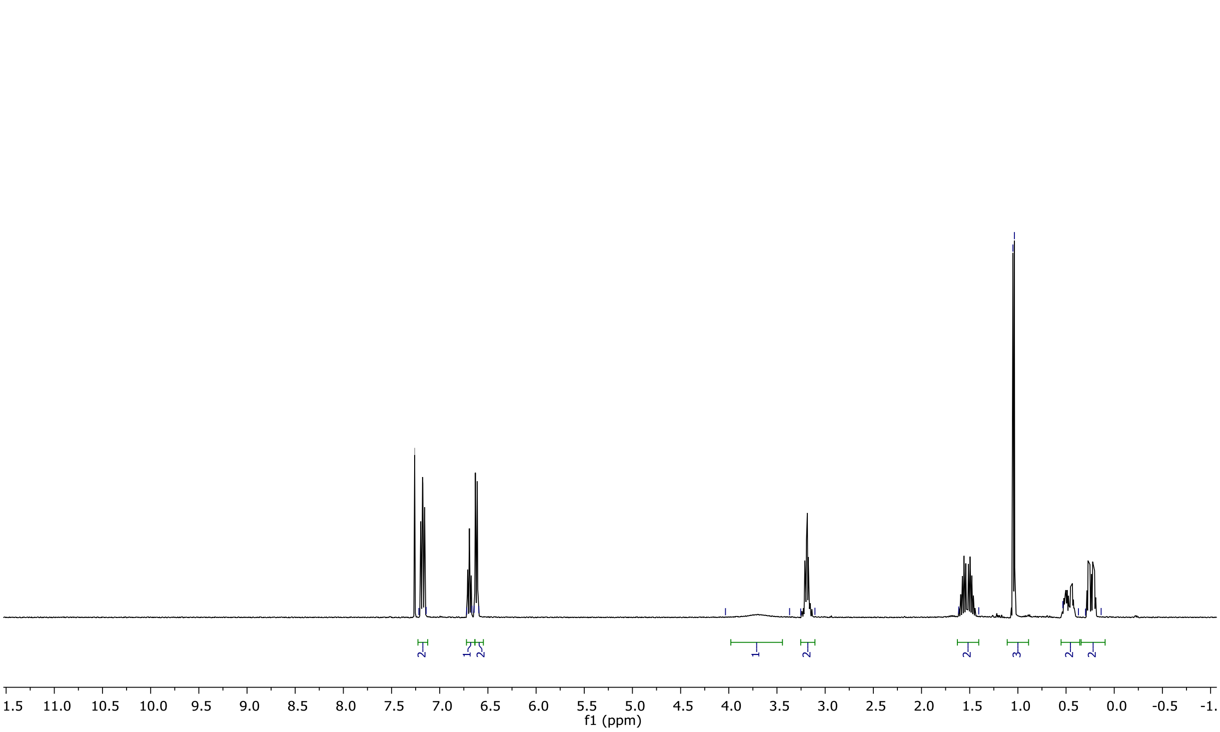

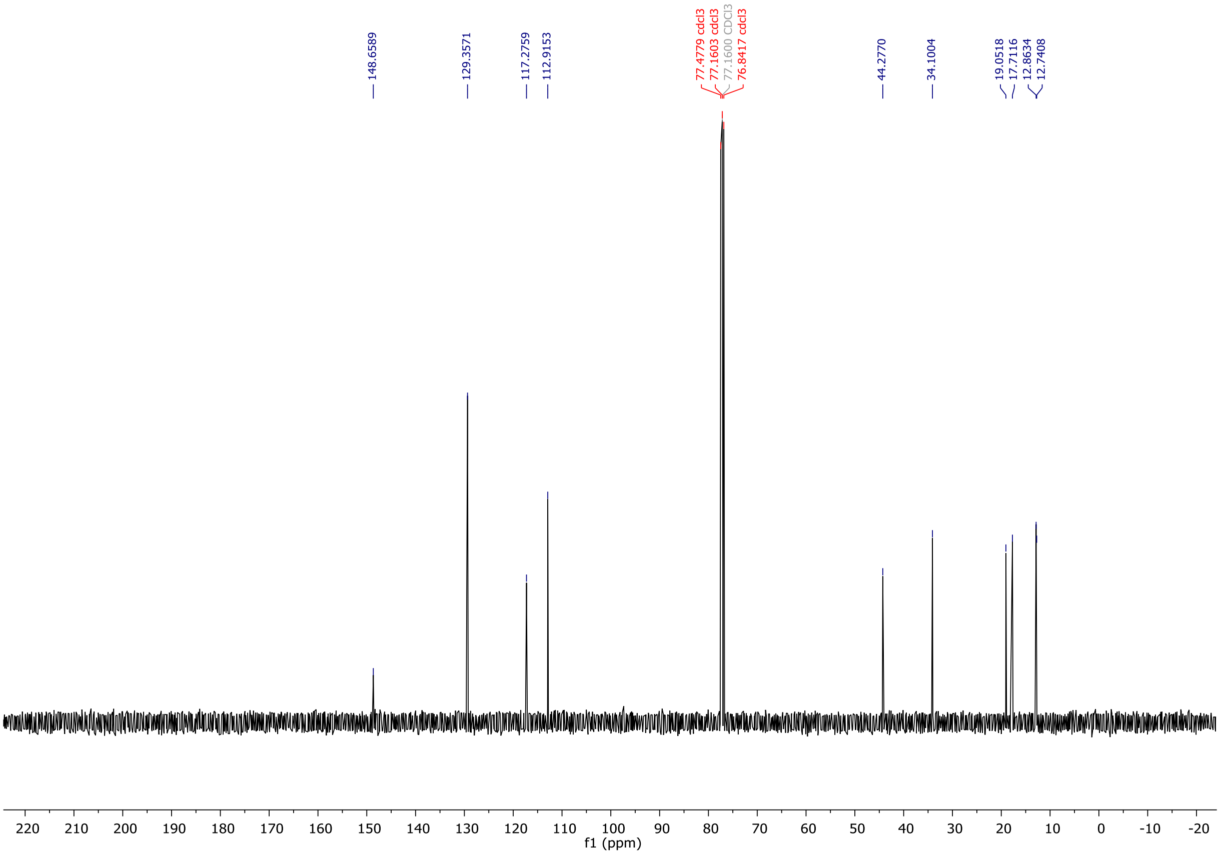

(*rac*)-(*trans*)-*N*-(2-(2-Ethylcyclopropyl)ethyl)aniline (1r)

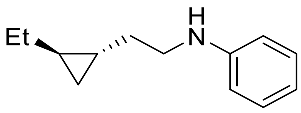

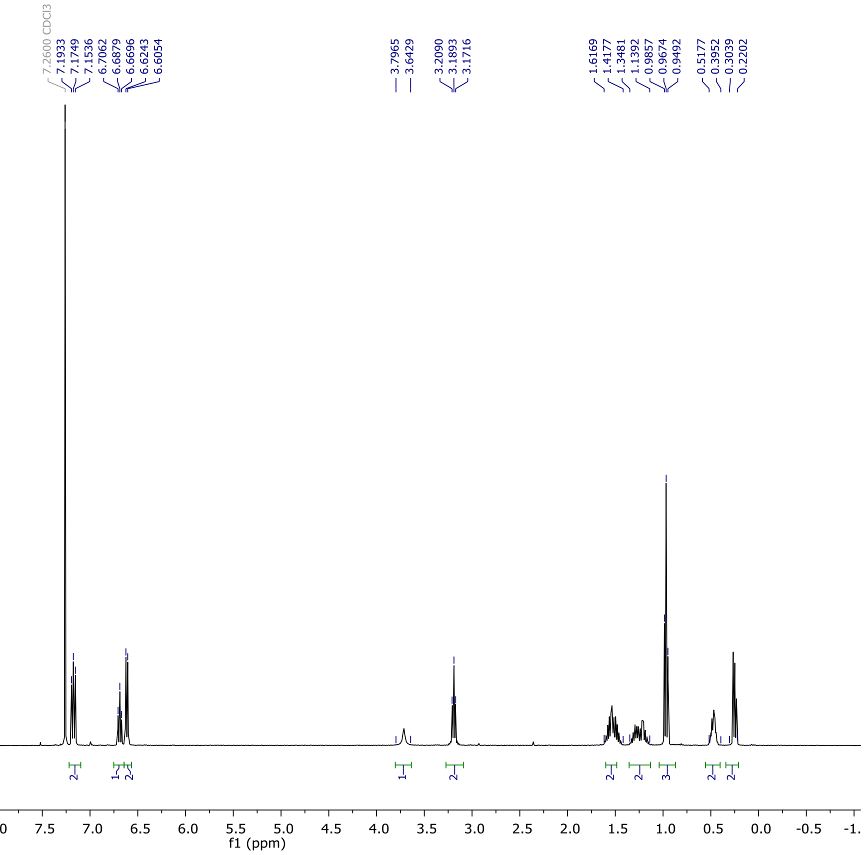

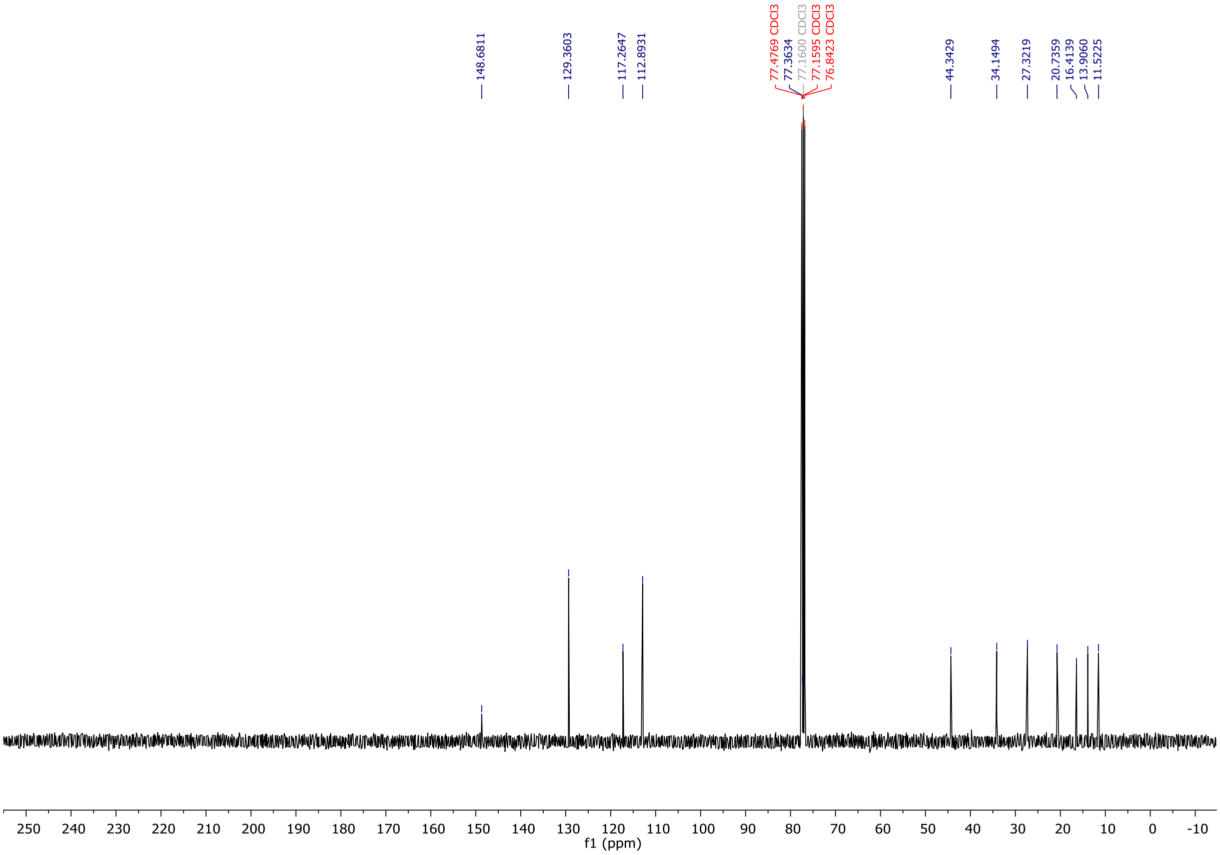

(*rac*)-(*trans*)-*N*-(2-(2-Phenylcyclopropyl)ethyl)aniline (1s)

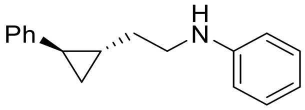

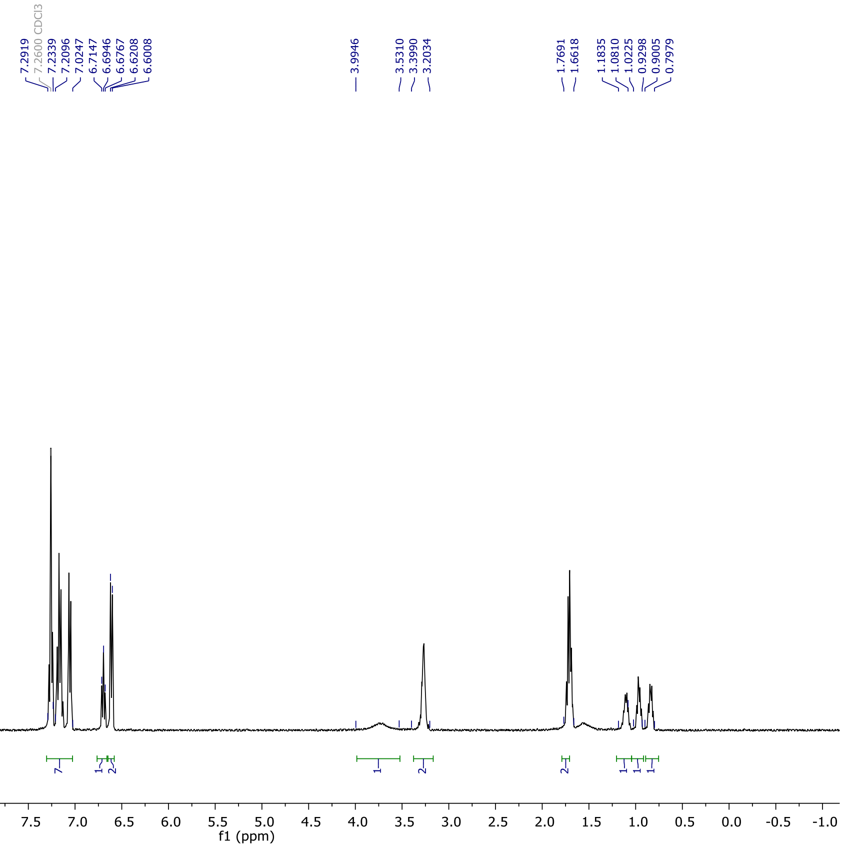

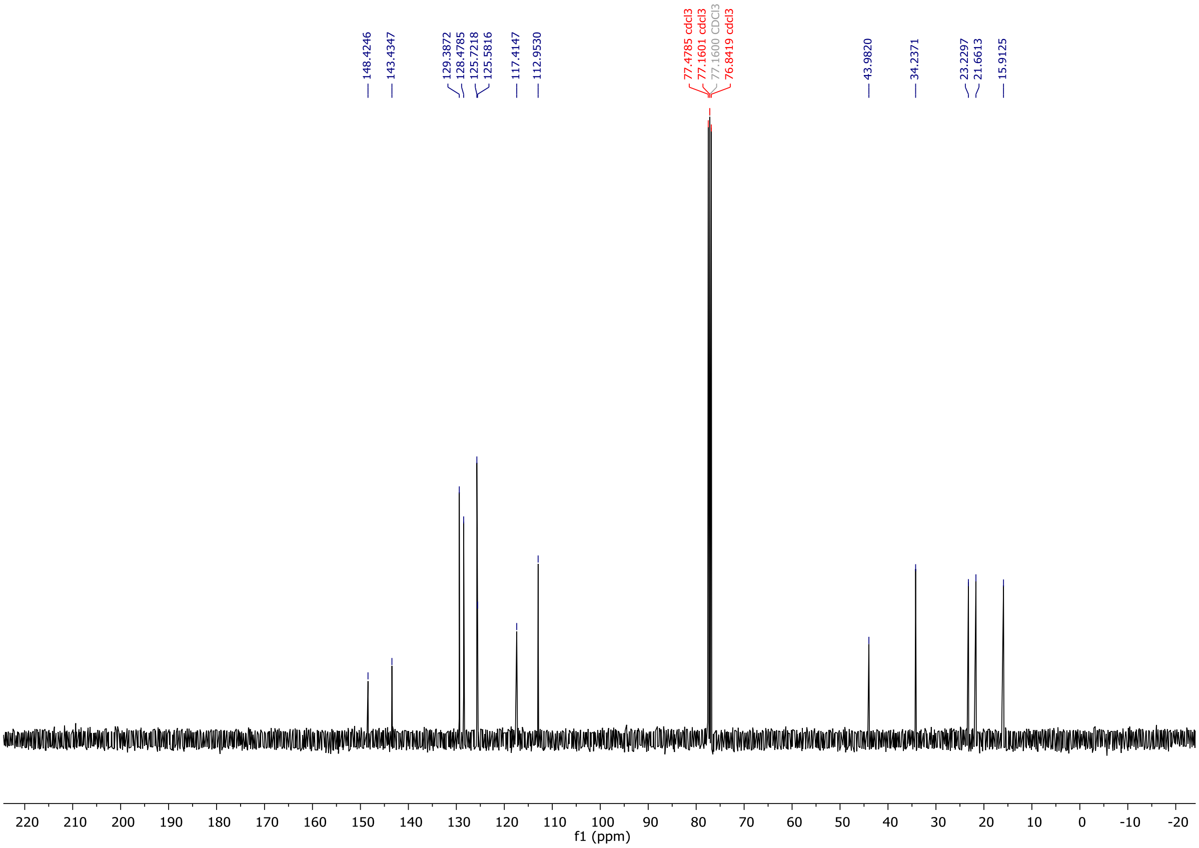

N-(2-(2,2-Dimethylcyclopropyl)ethyl)aniline (1t)

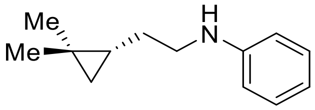

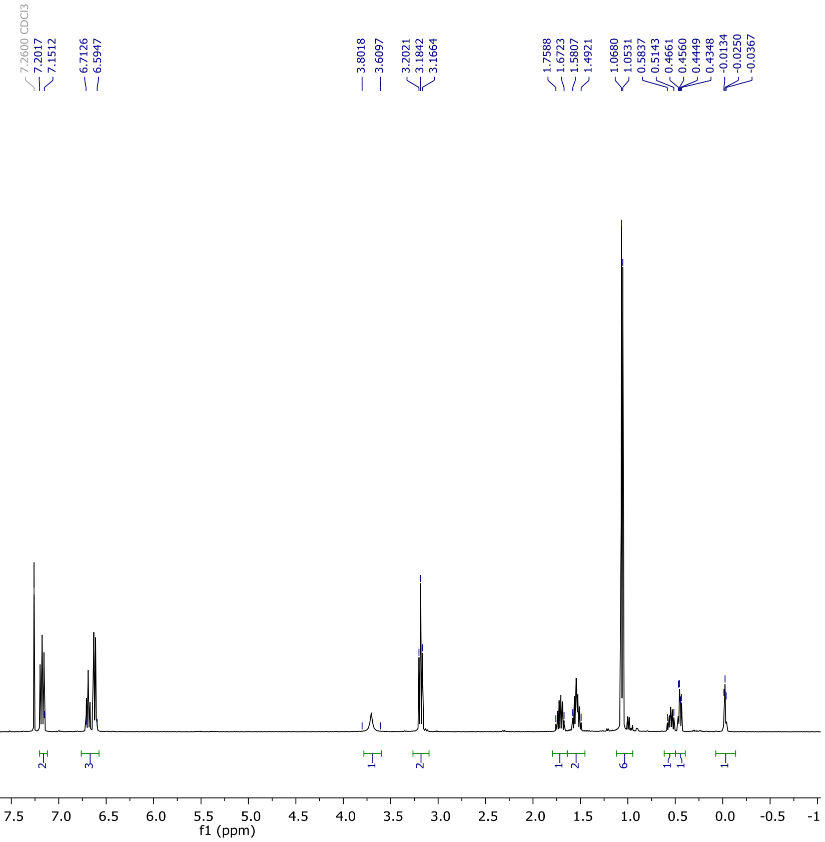

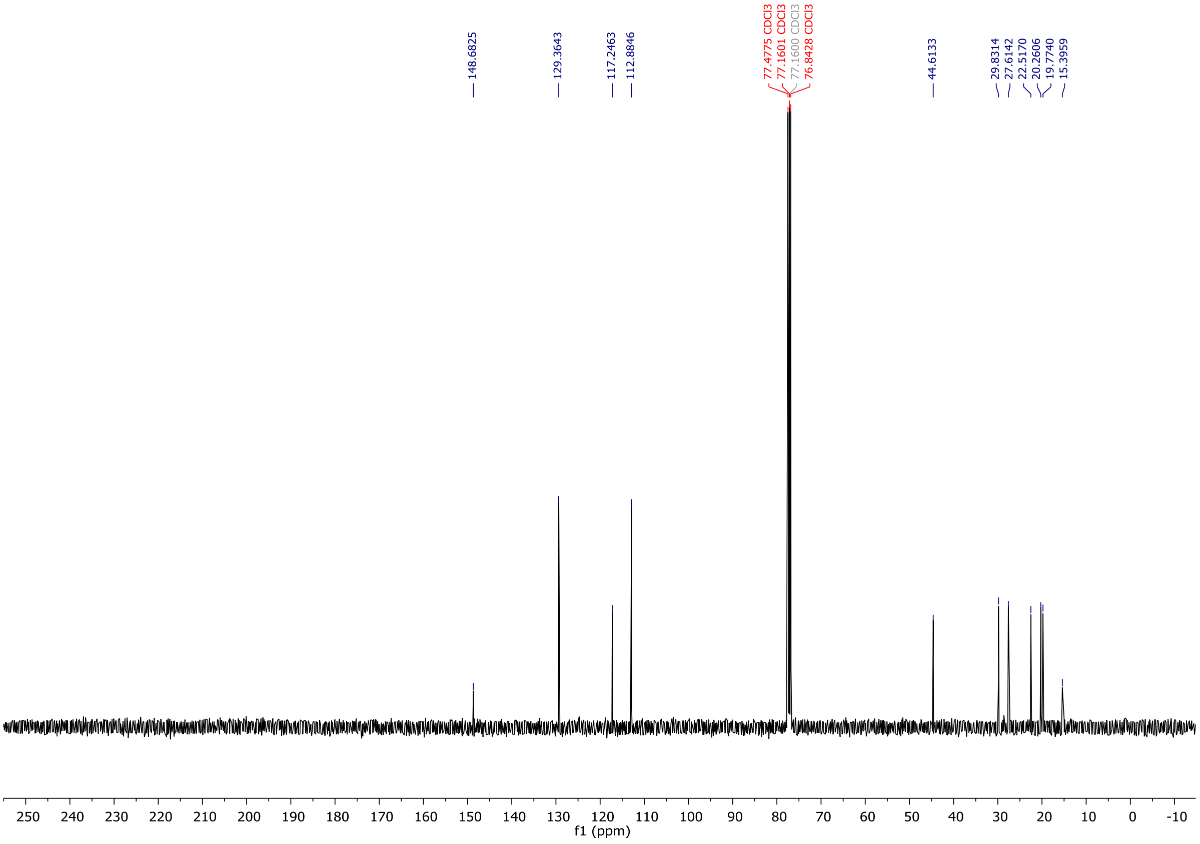

(*rac*)-*N*-((Bicyclo[4.1.0]heptan-2-yl)methyl)aniline (1u)

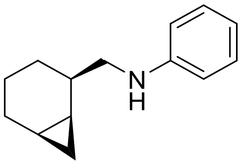

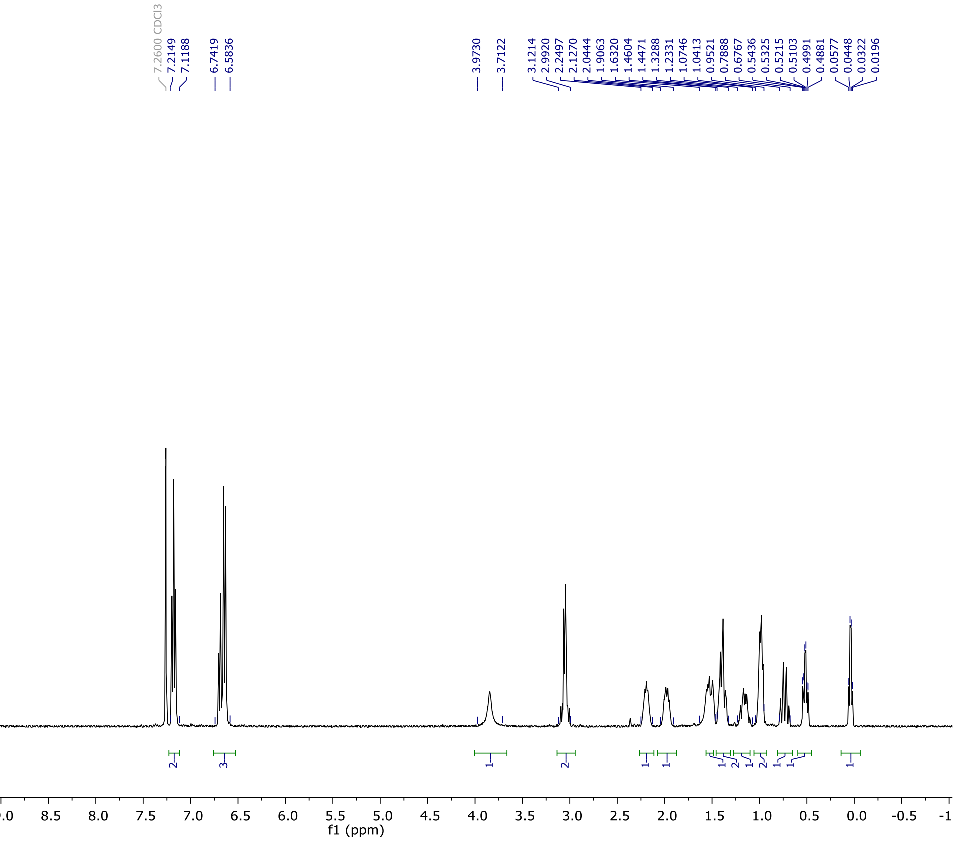

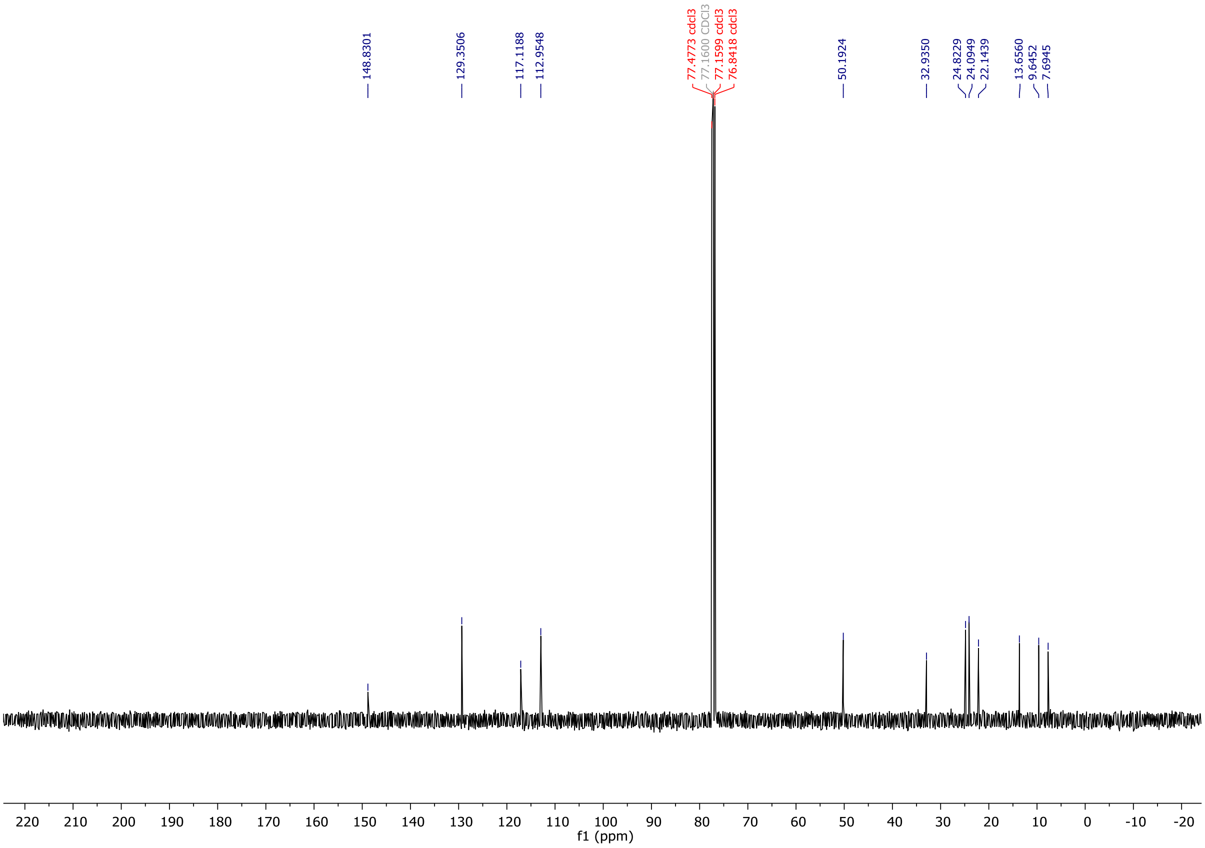

_N_-(2-Cyclopropylbenzyl)-1,1-diphenylmethanamine (1v)

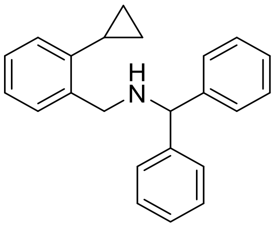

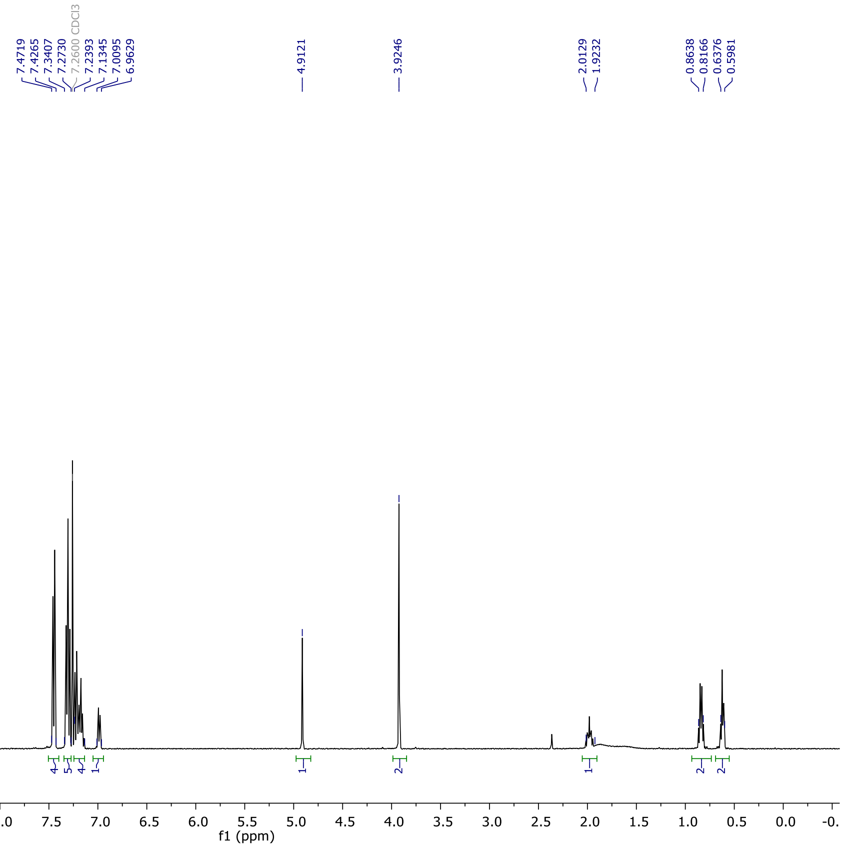

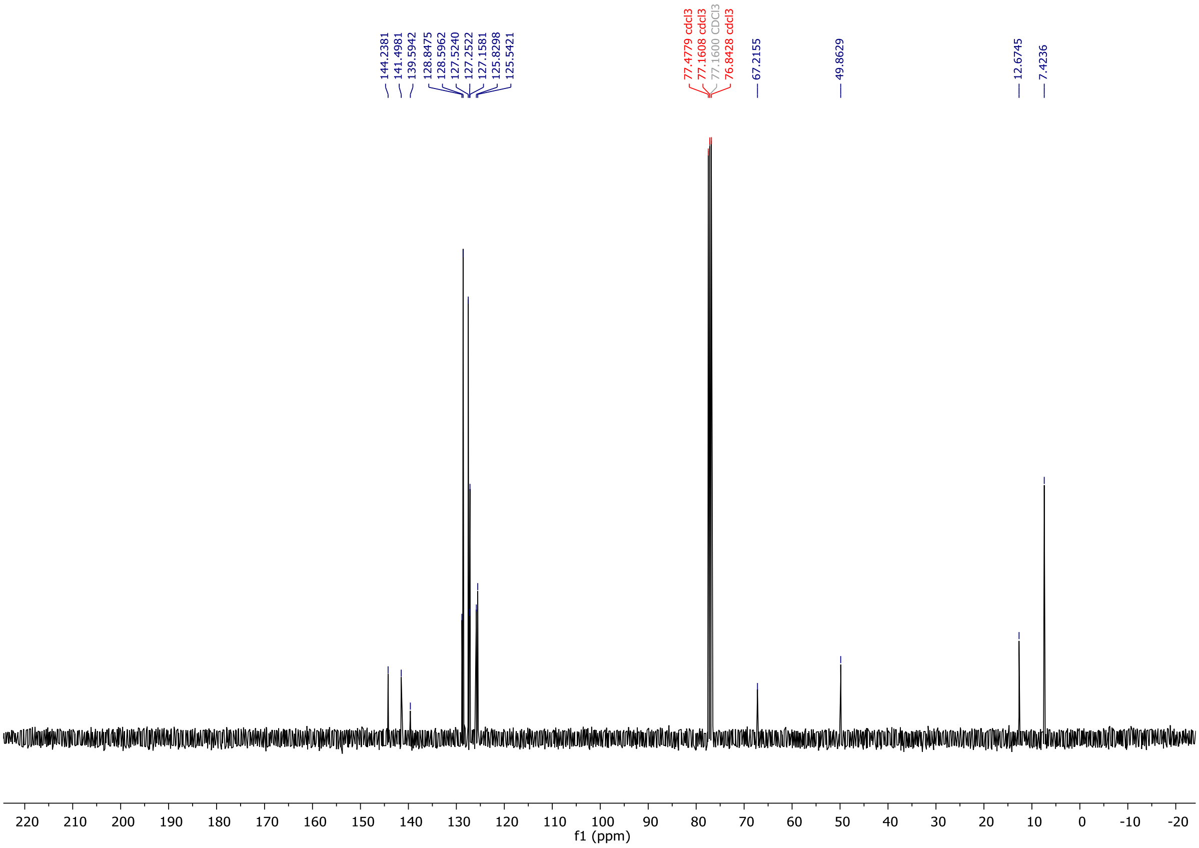

N-(2-Cyclopropyl-5-fluorobenzyl)-1,1-diphenylmethanamine (1w)

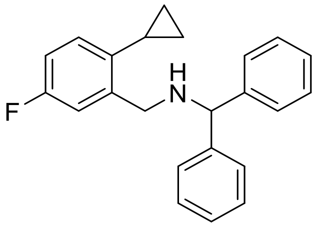

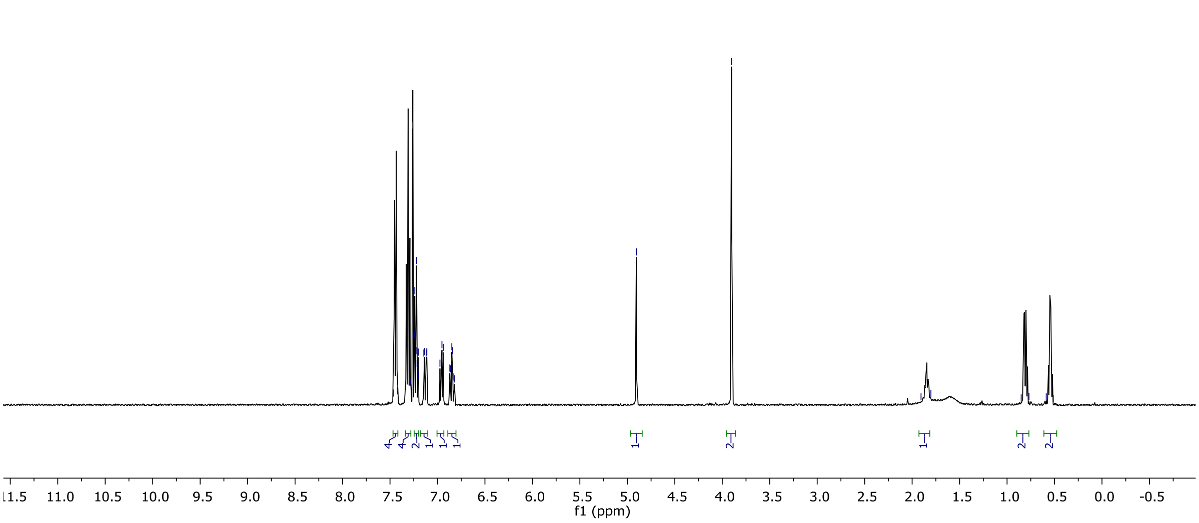

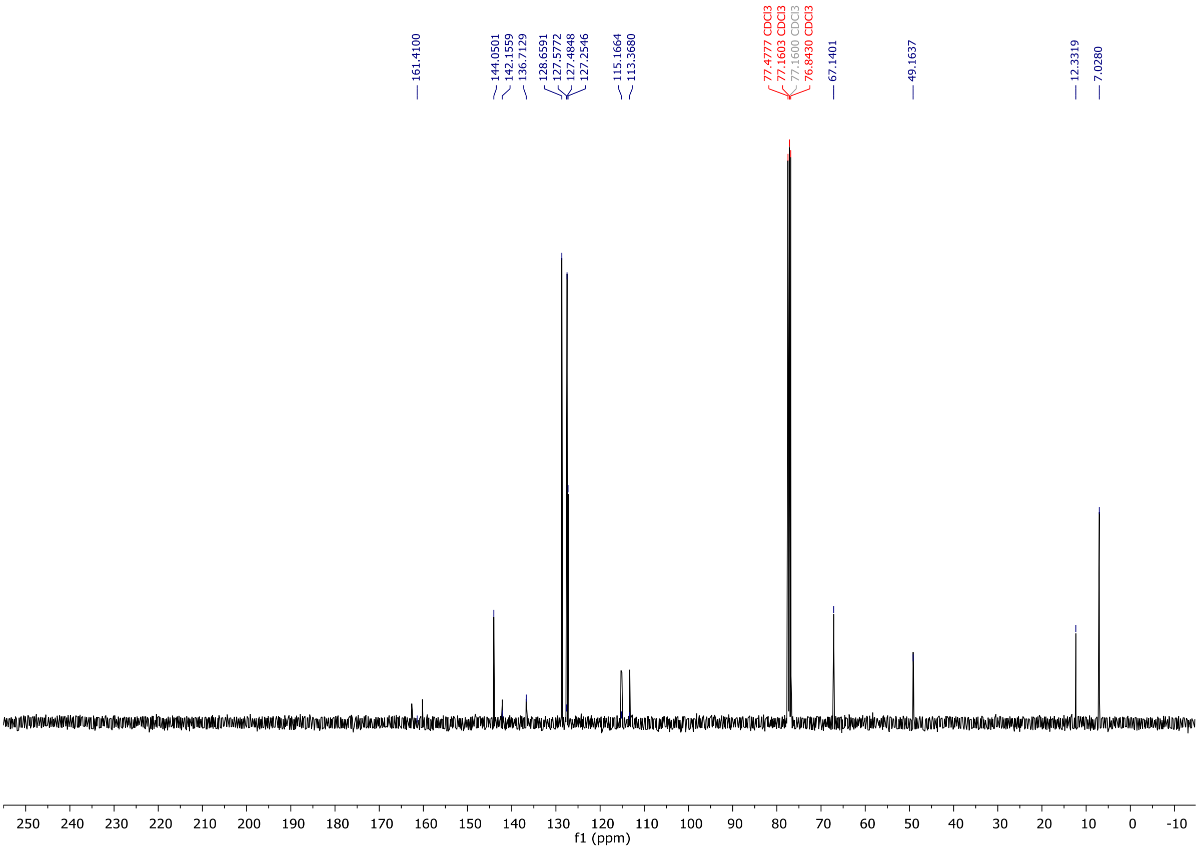

N-(2-Cyclopropyl-5-methoxybenzyl)-1,1-diphenylmethanamine (1x)

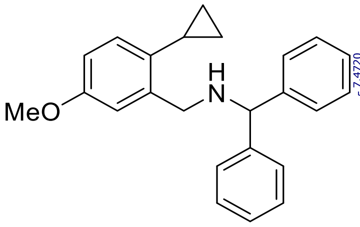

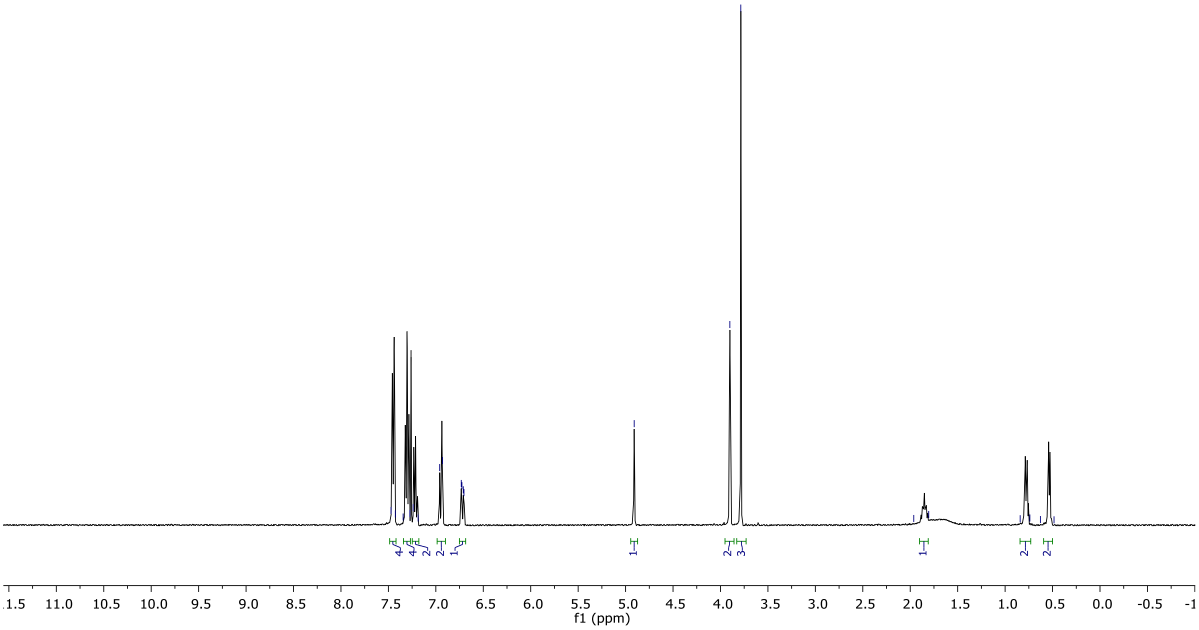

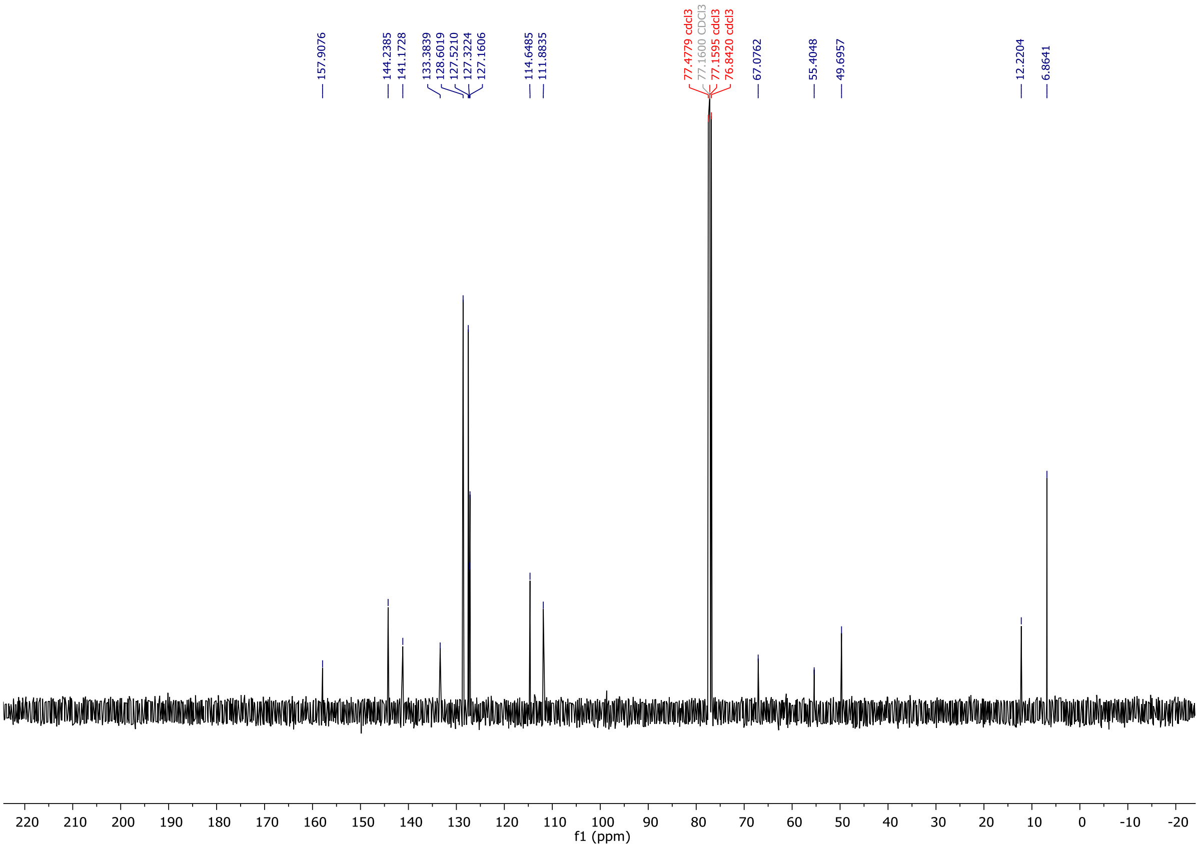

Benzylcyclopropane

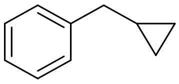

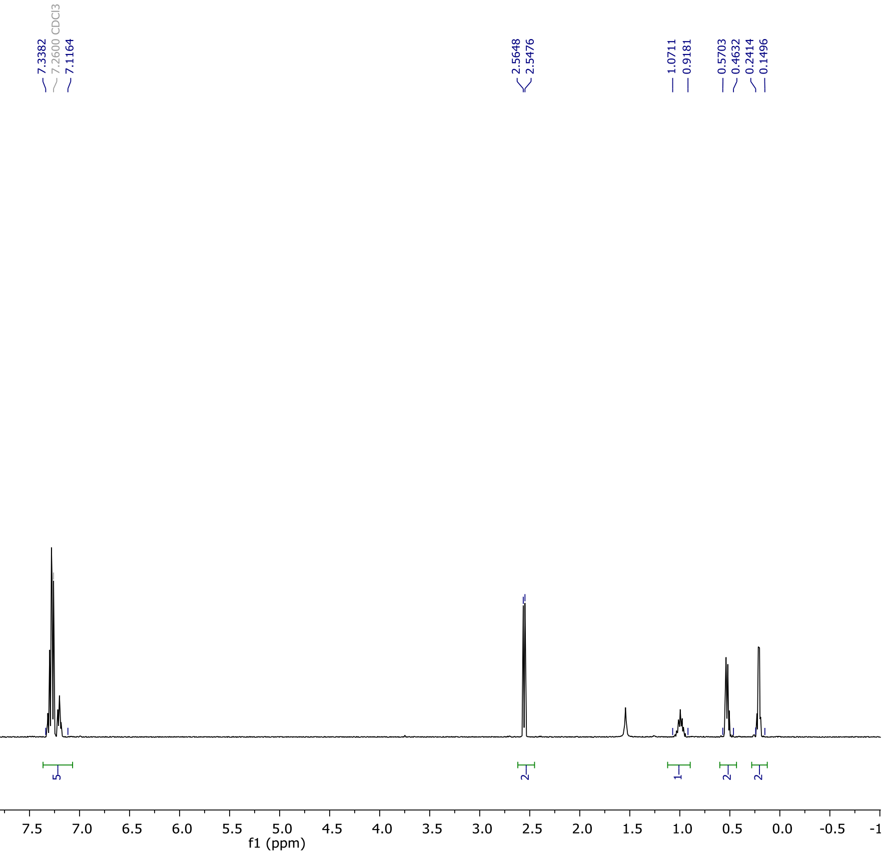

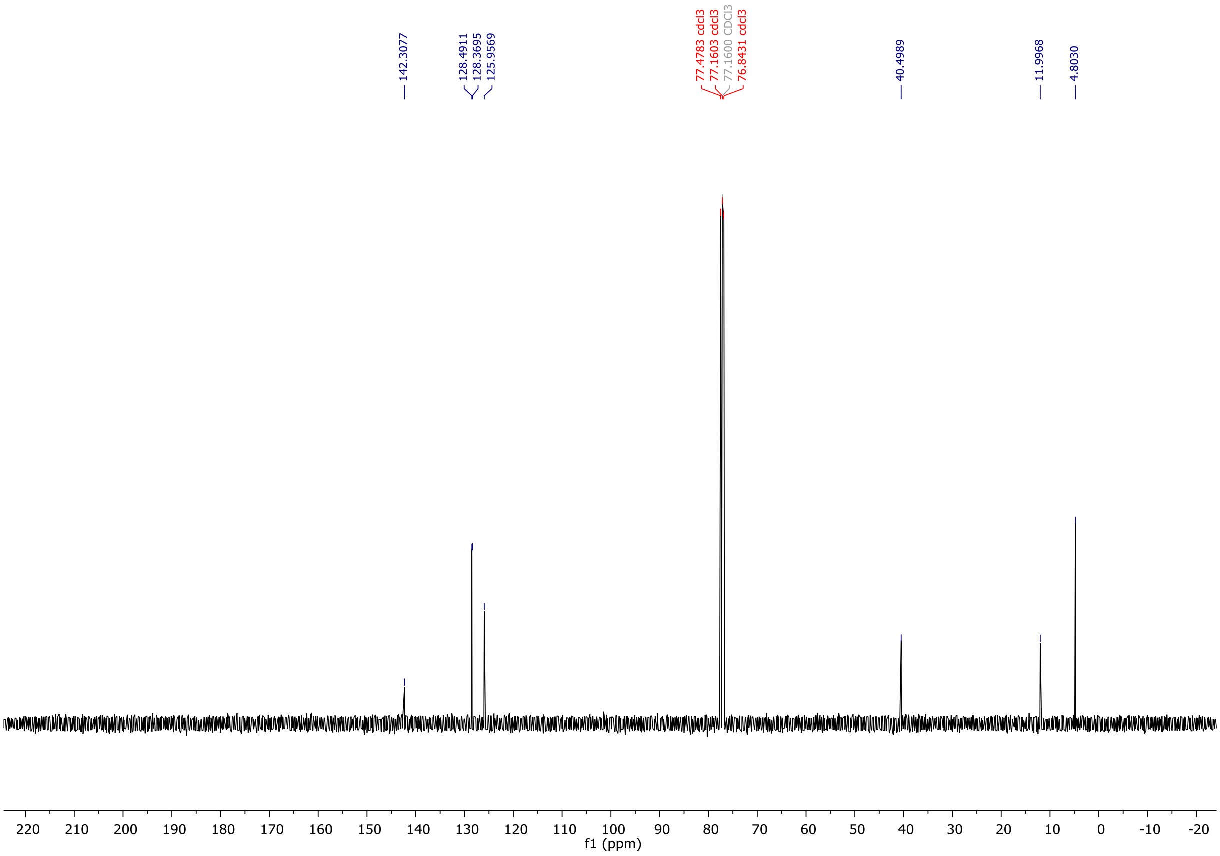

2-(Cyclopropylmethyl)aniline (1y)

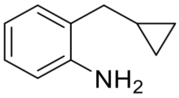

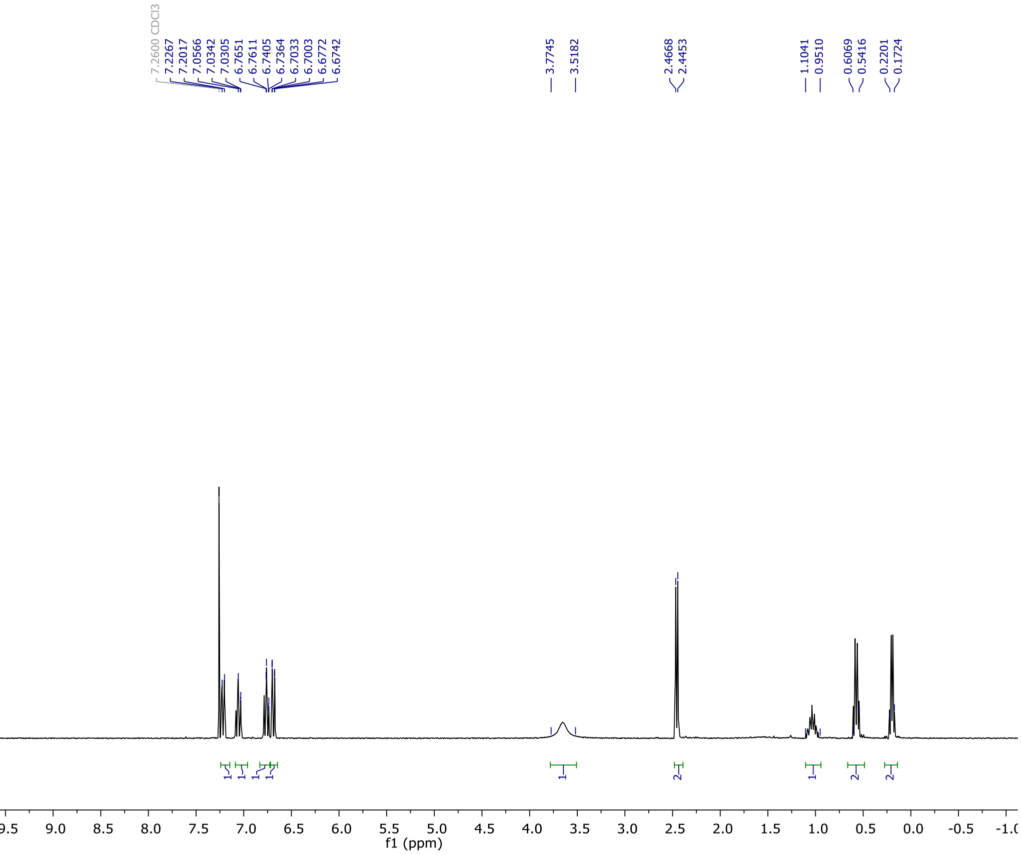

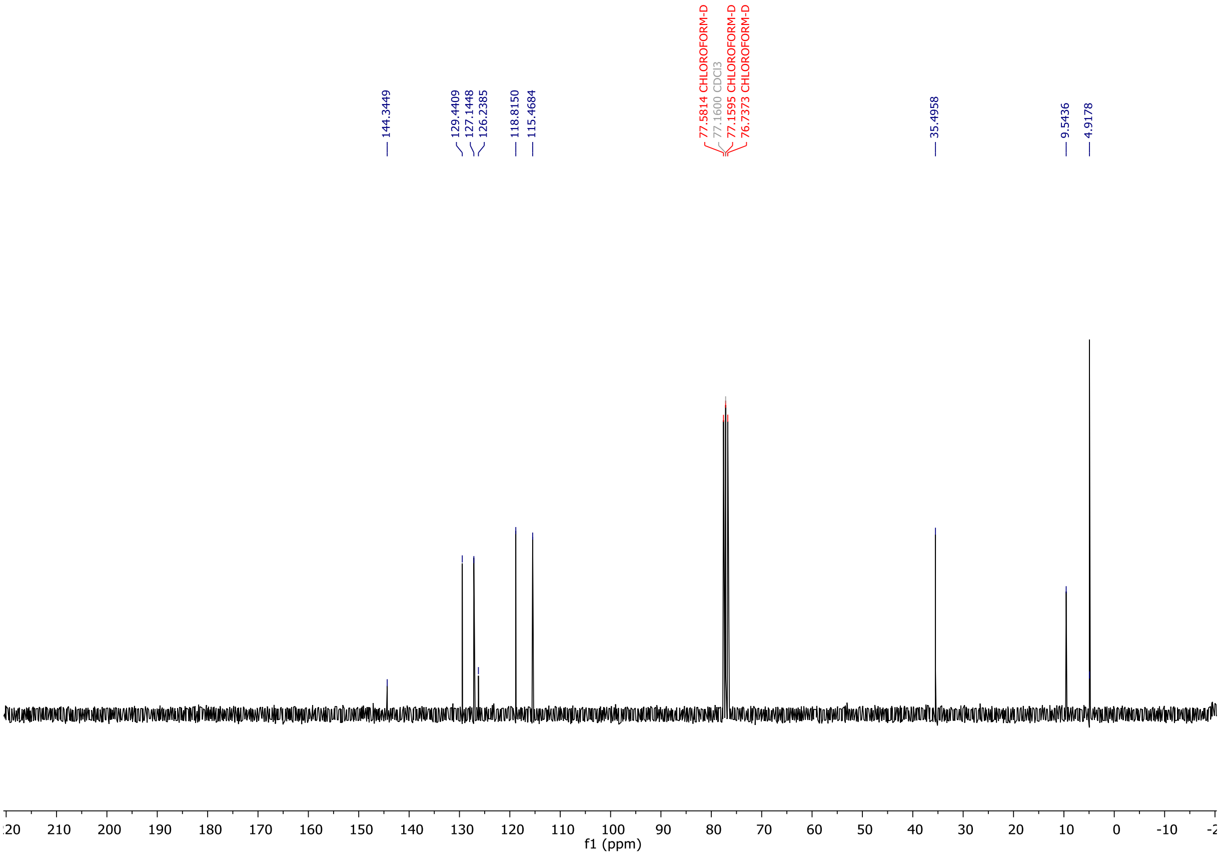

2-(Cyclopropylmethyl)-5-methoxyaniline (1z)

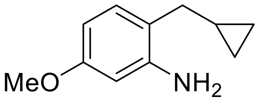

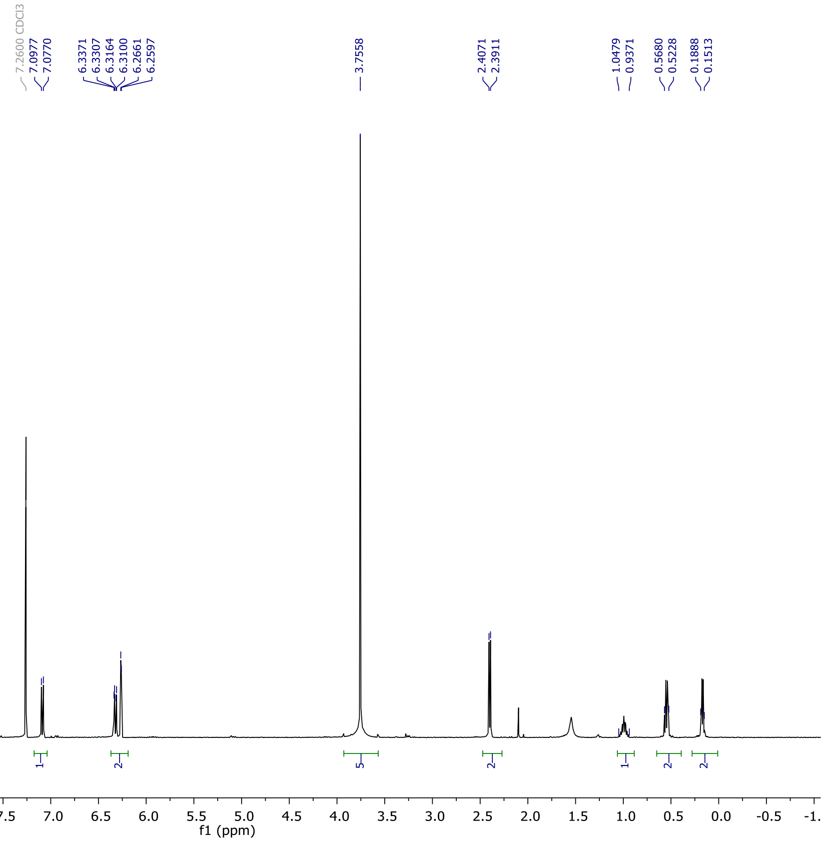

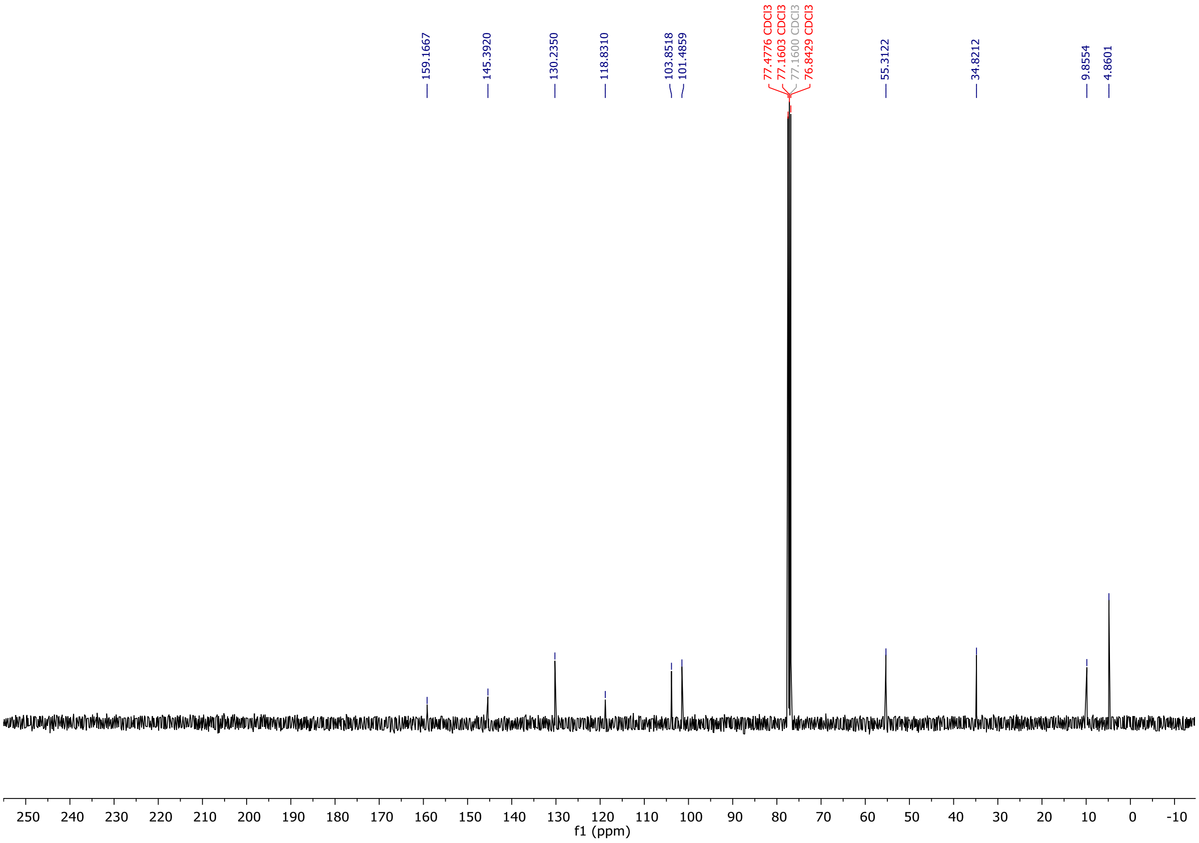

2-(Cyclopropylmethyl)-5-(trifluoromethyl)aniline (1aa)

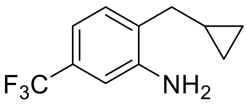

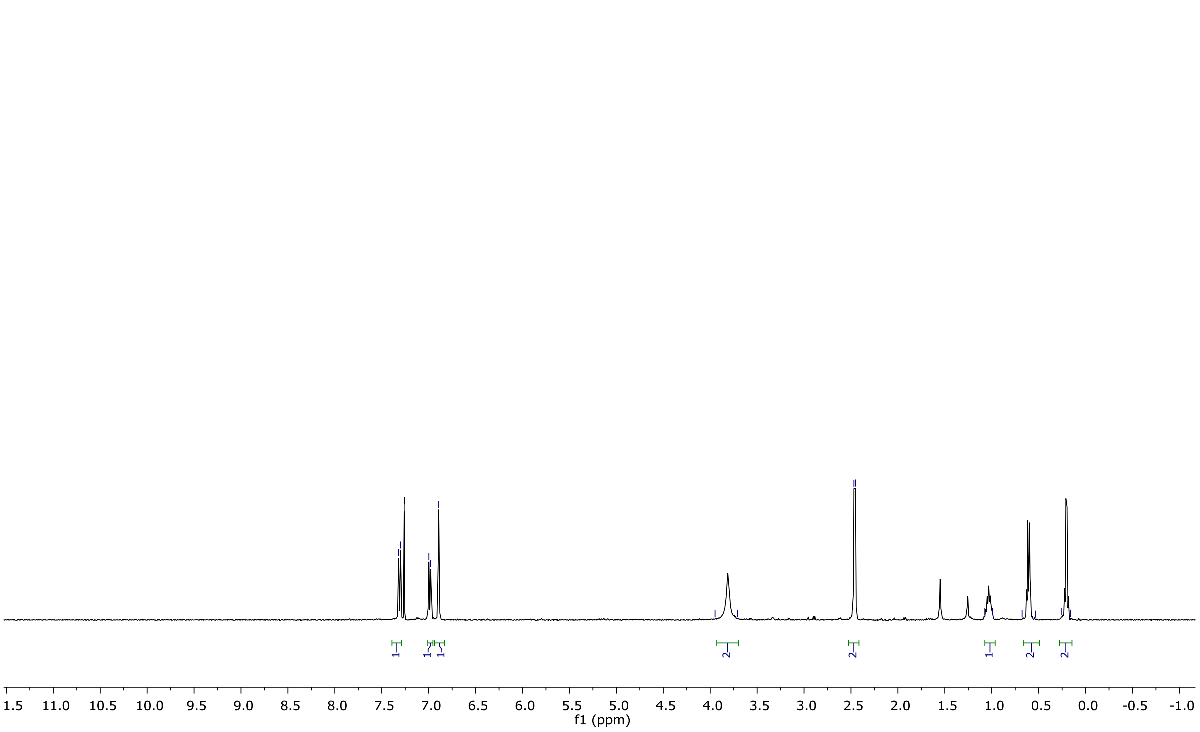

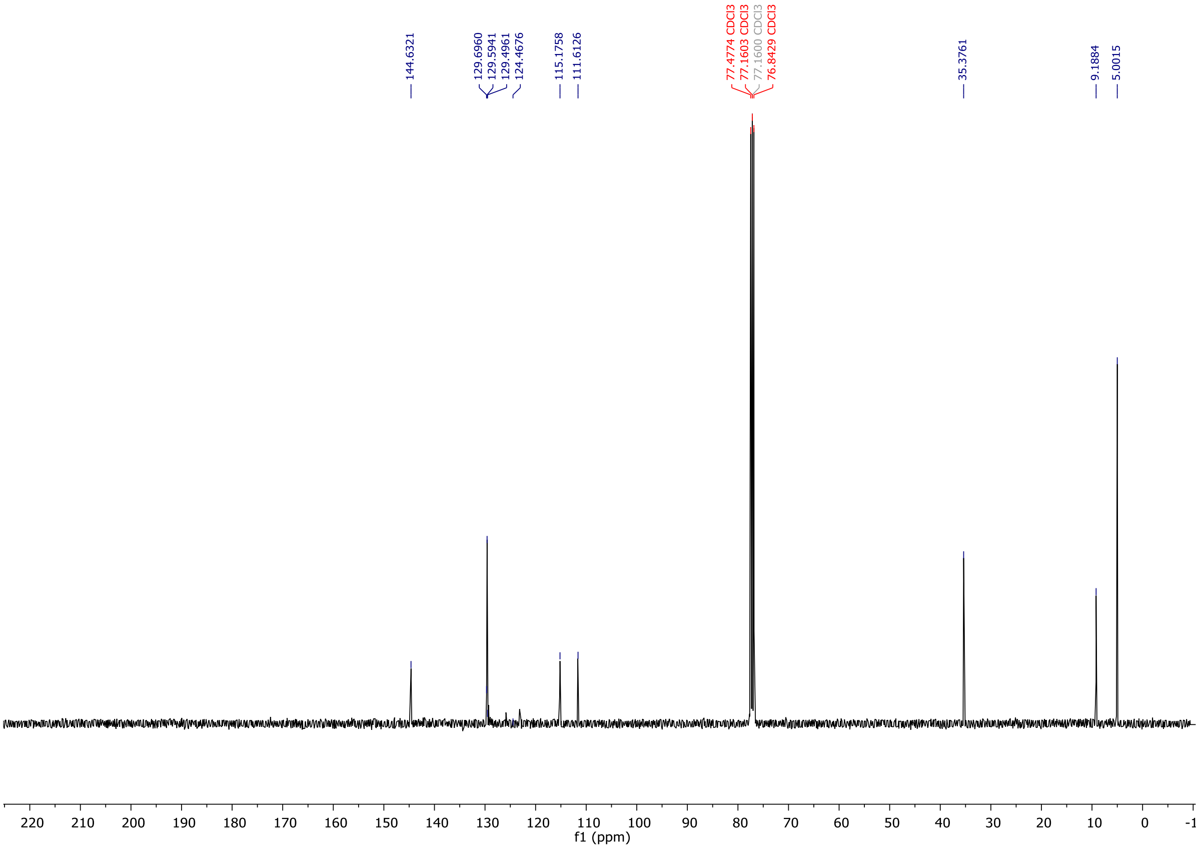

1-Benzyl-1,3-dihydro-2*H*-benzo[*b*]azepin-2-one (2a)

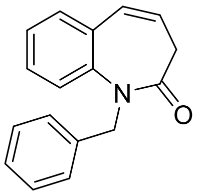

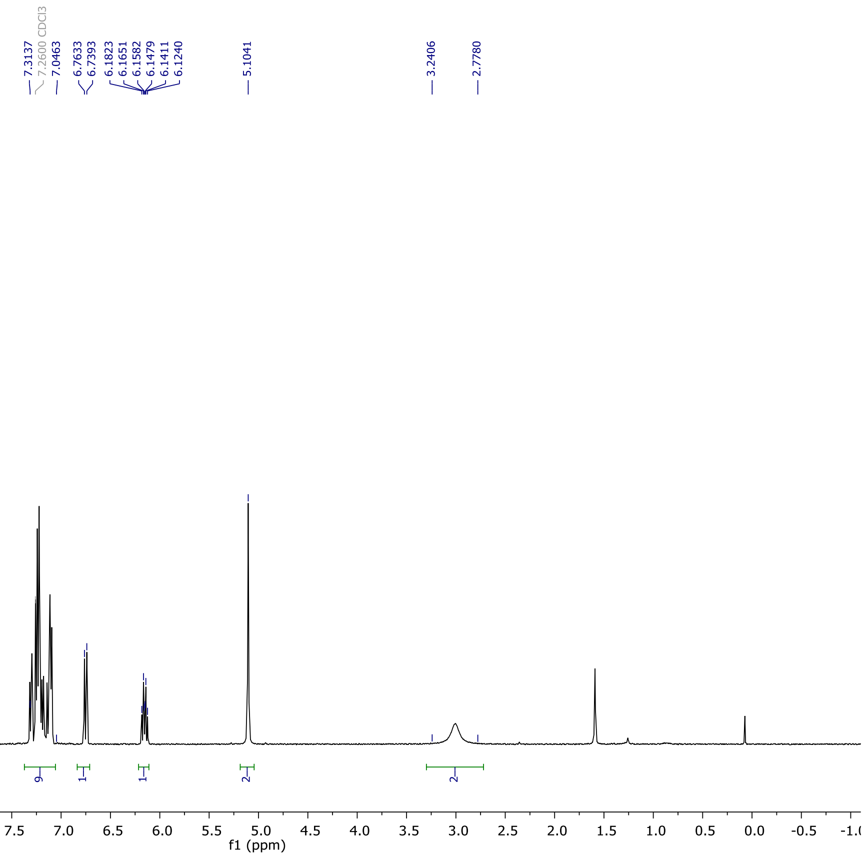

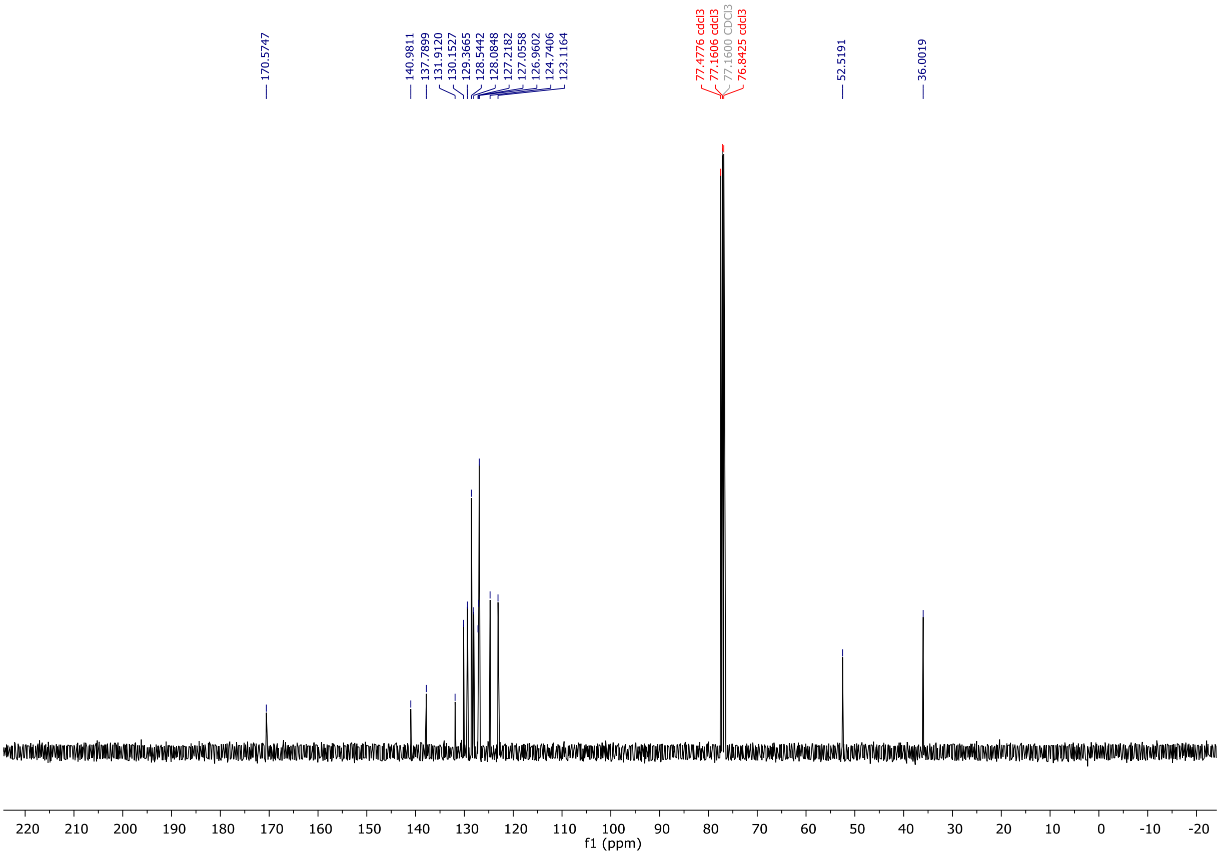

1-Phenyl-1,3-dihydro-2*H*-benzo[*b*]azepin-2-one (2b)

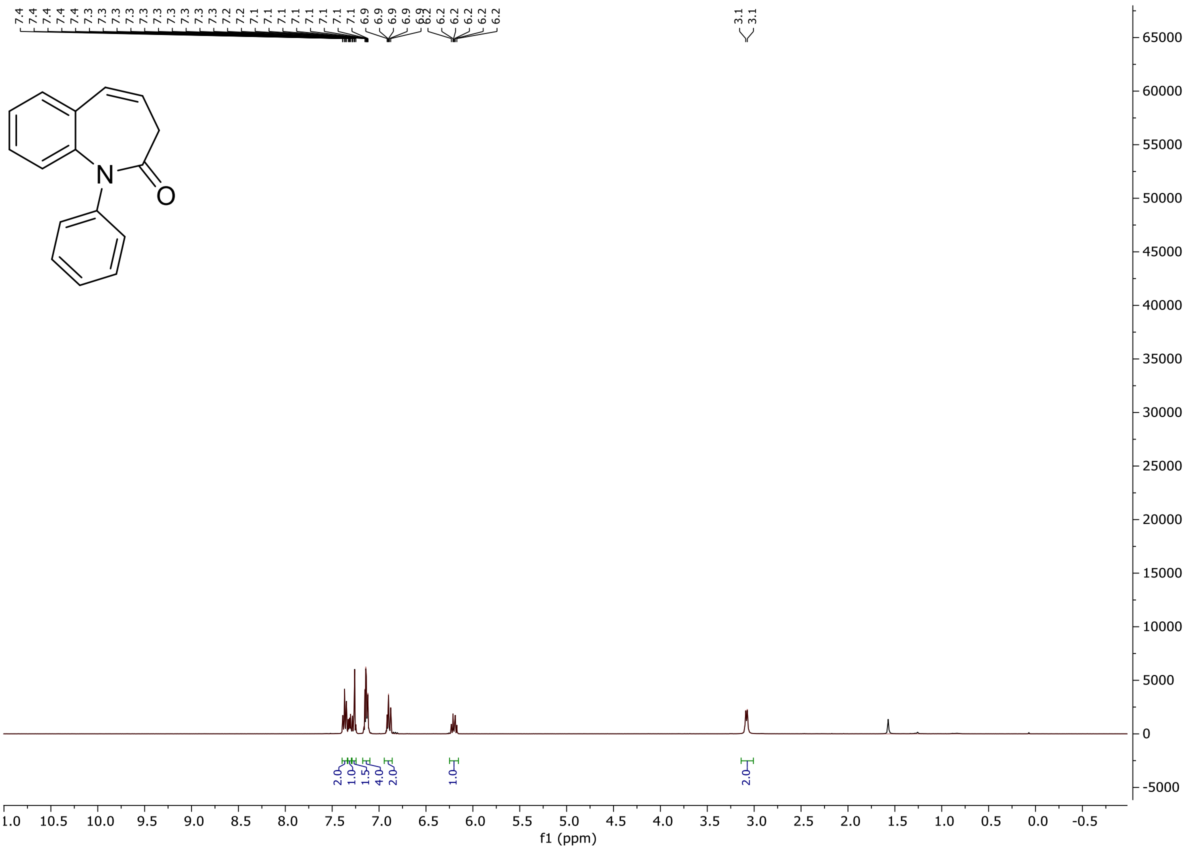

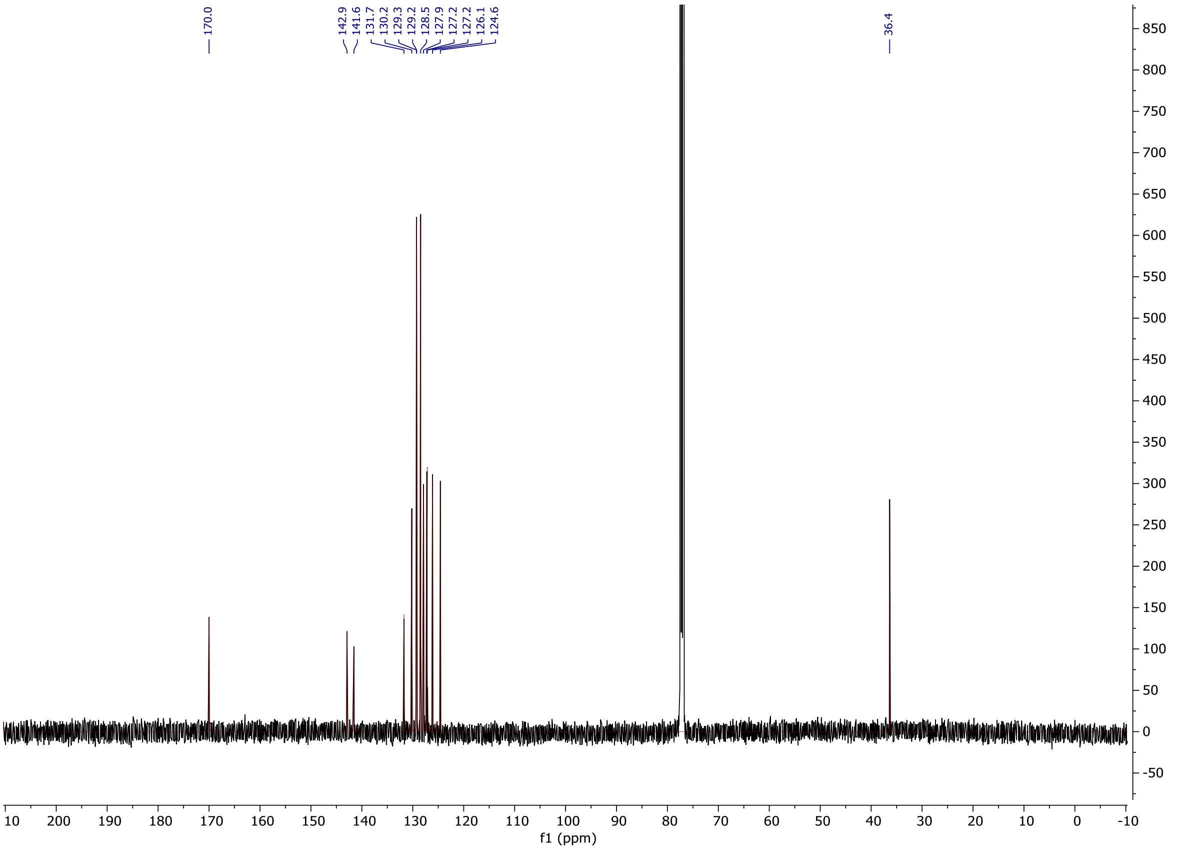

1-(4-Methoxyphenyl)-1,3-dihydro-2*H*-benzo[*b*]azepin-2-one (2c)

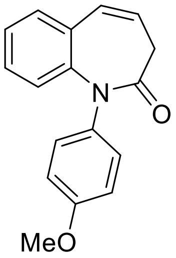

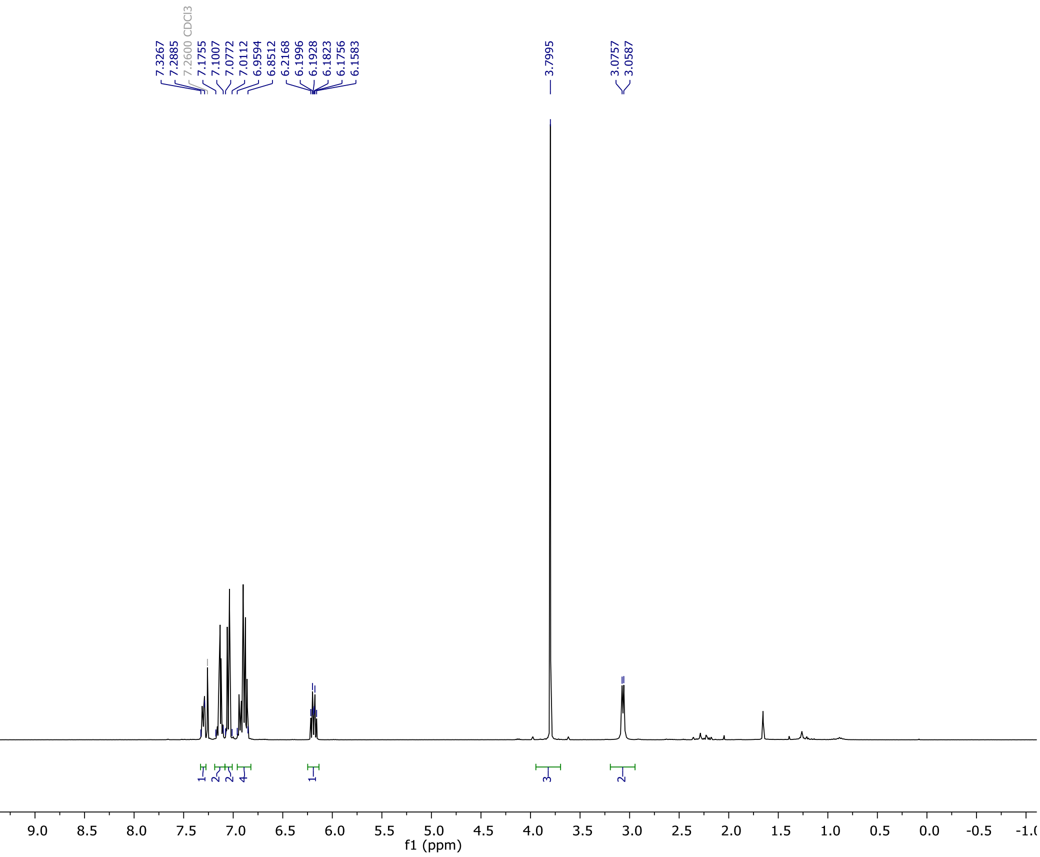

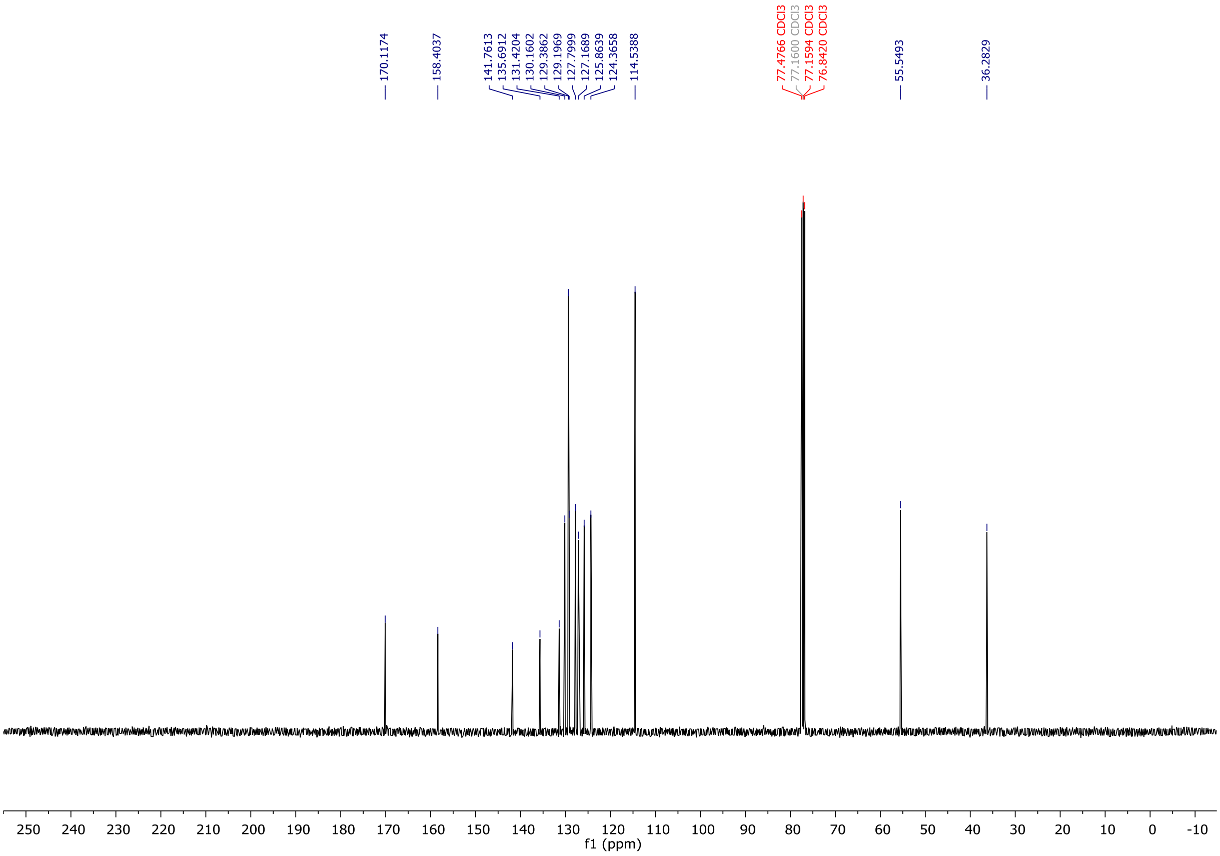

1,3-Dihydro-2*H*-benzo[*b*]azepin-2-one (2d)

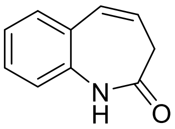

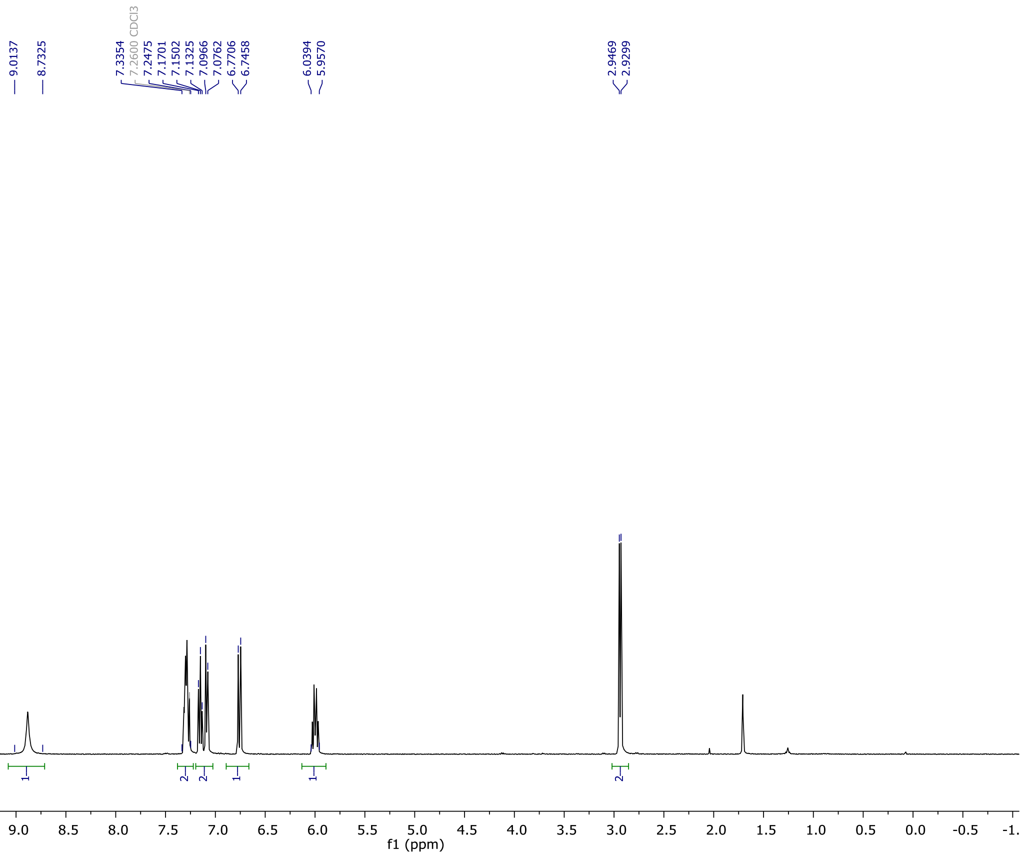

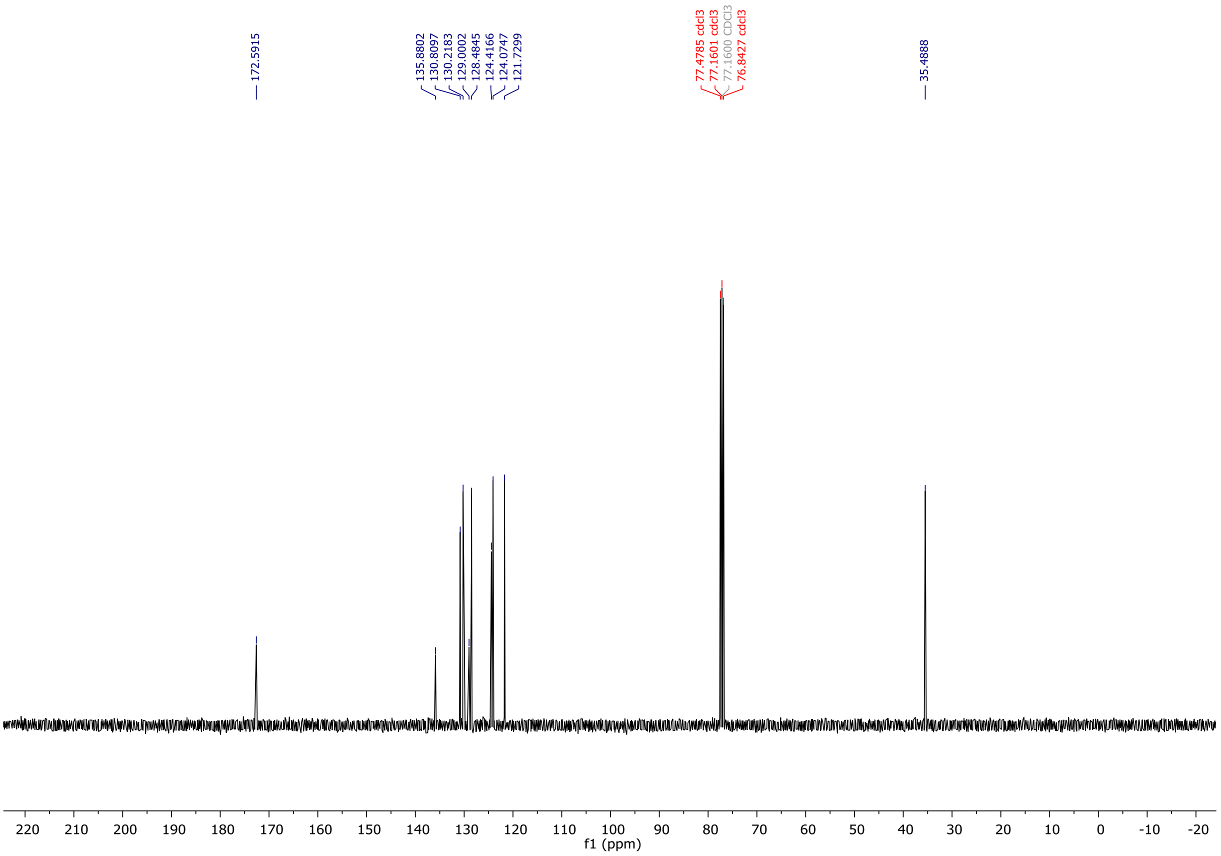

8-Methoxy-1,3-dihydro-2*H*-benzo[*b*]azepin-2-one (2e)

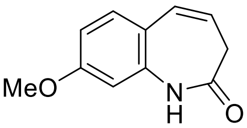

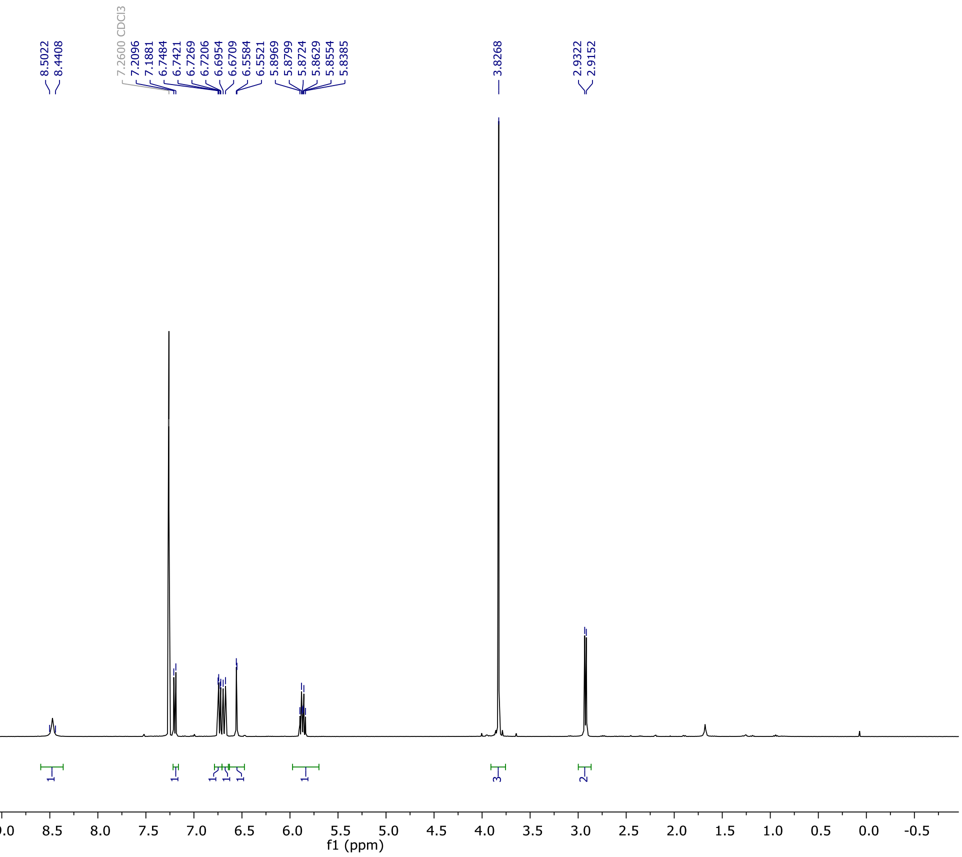

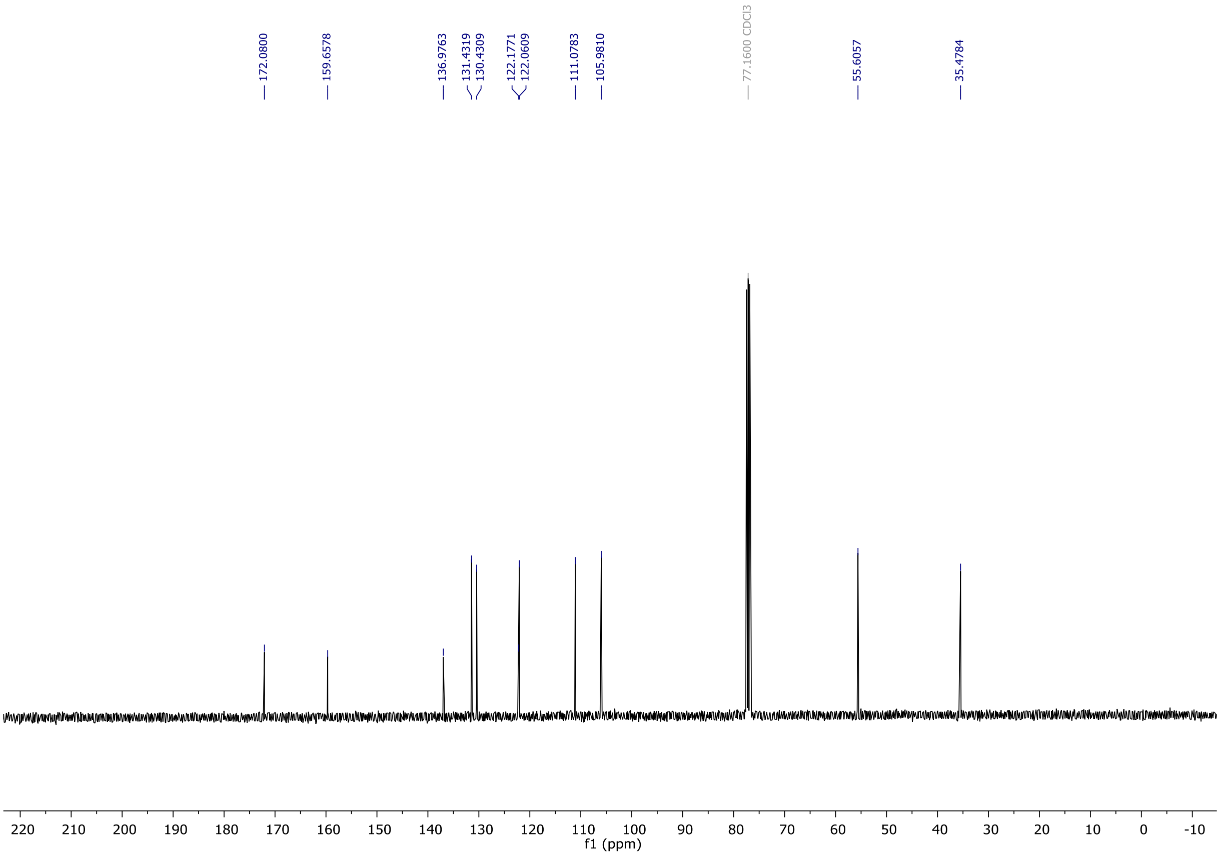

8-(Trifluoromethyl)-1,3-dihydro-2*H*-benzo[*b*]azepin-2-one (2f)

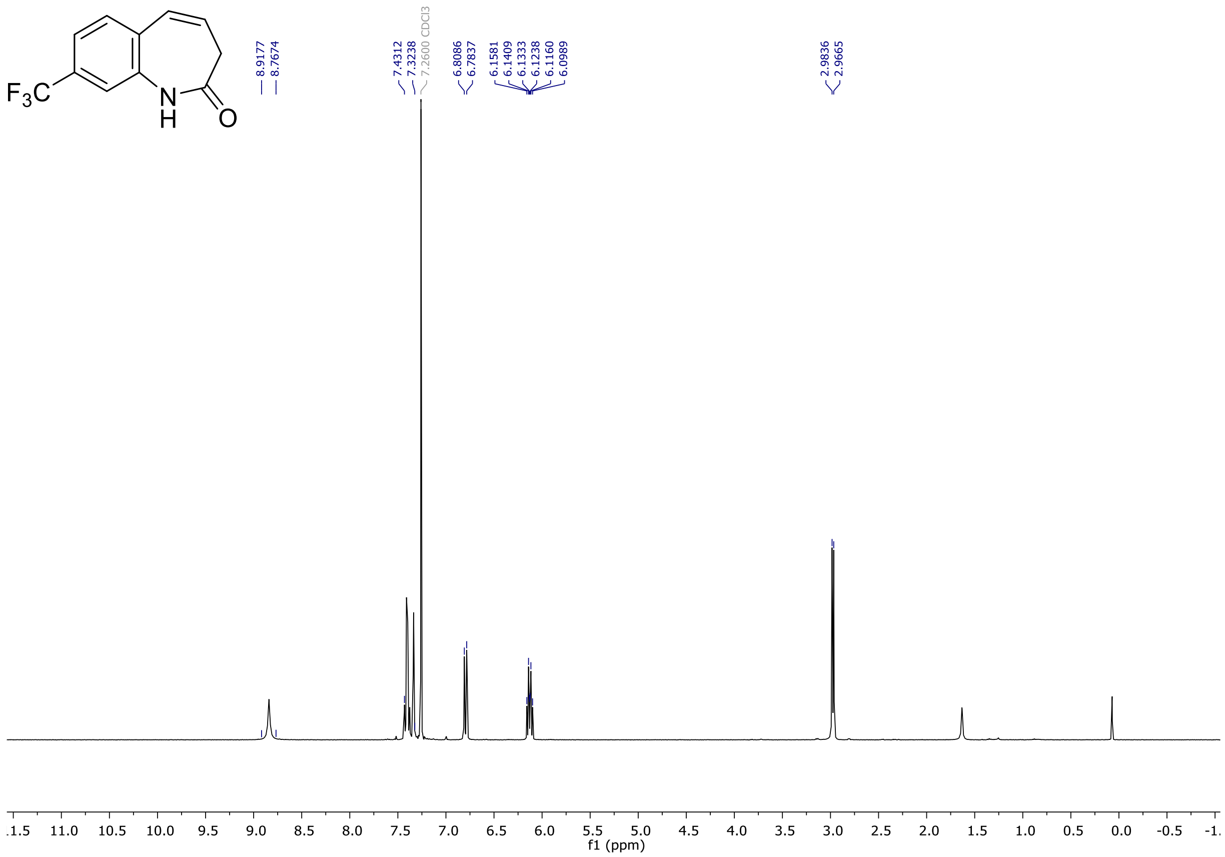

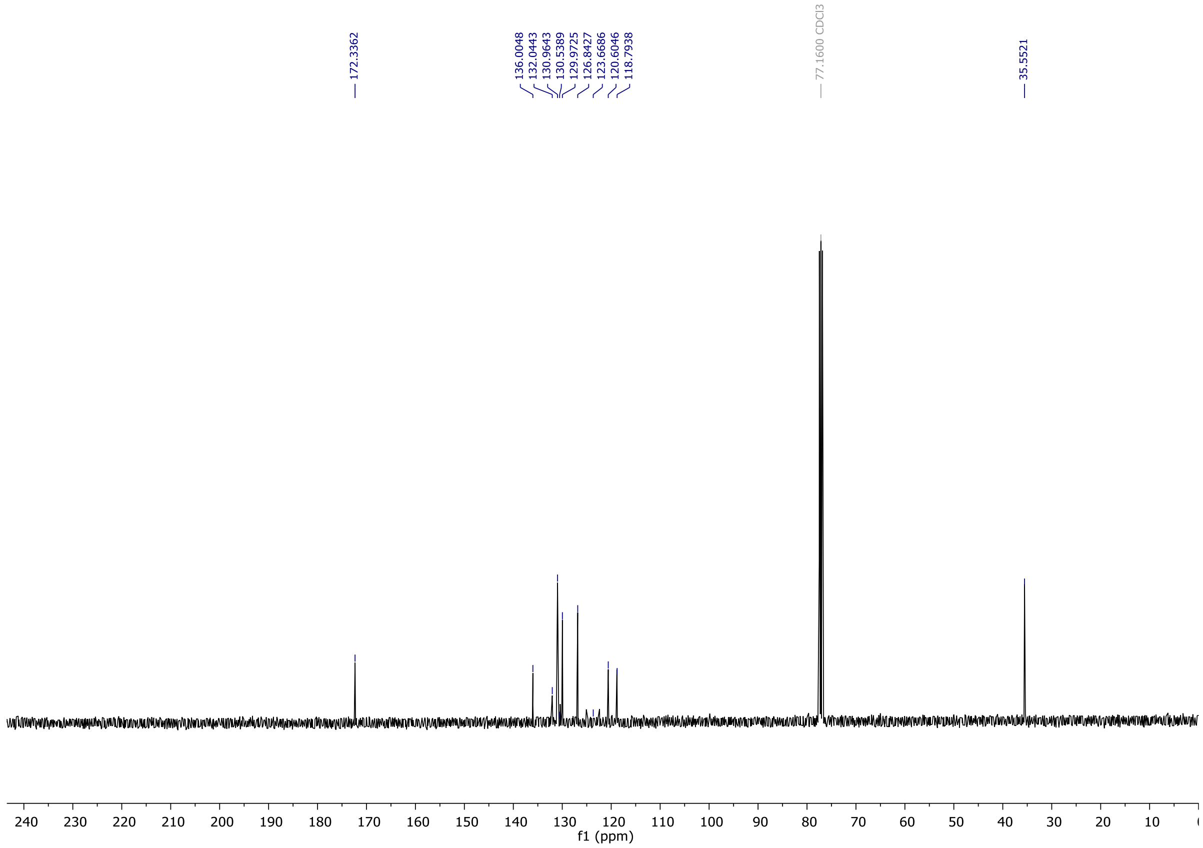

5,7,8,9-Tetrahydro-6*H*-pyrido[3,2-*b*]azepin-6-one (2g’)

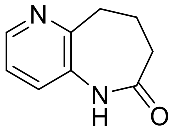

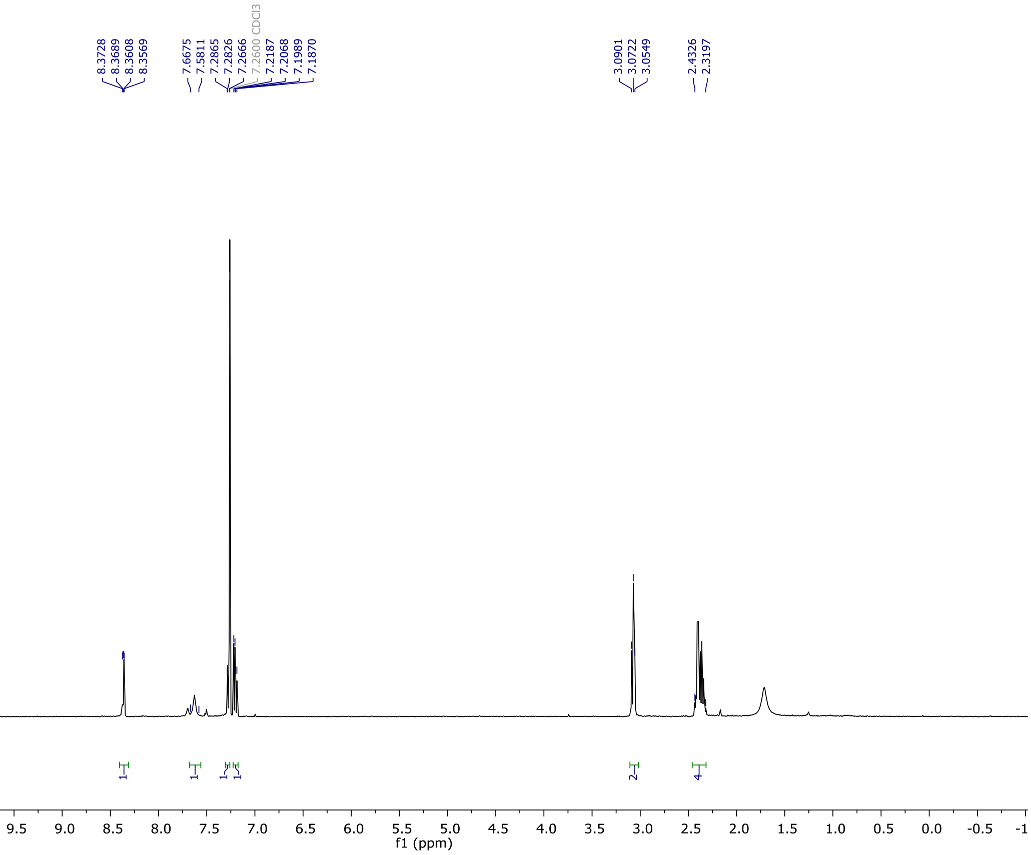

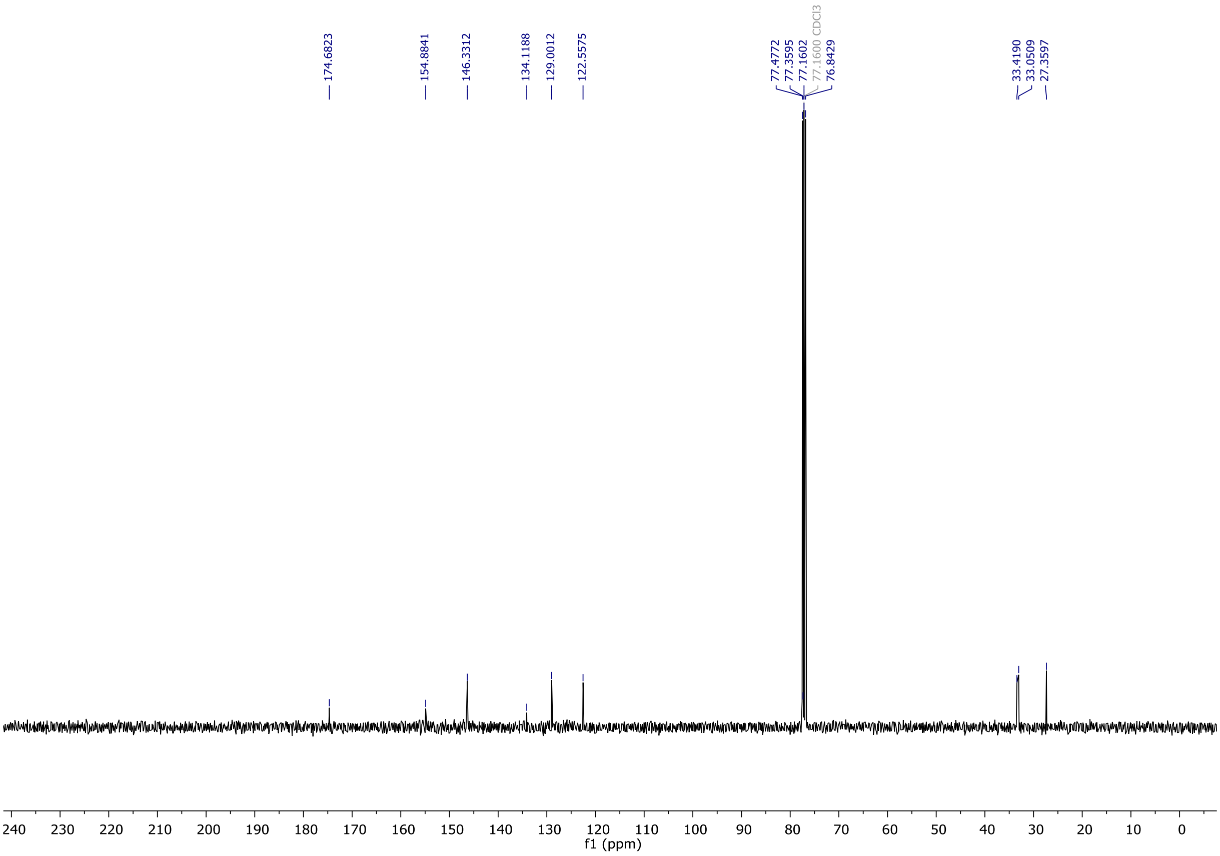

4-Methyl-1,3-dihydro-2*H*-benzo[*b*]azepin-2-one (2h)

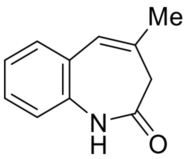

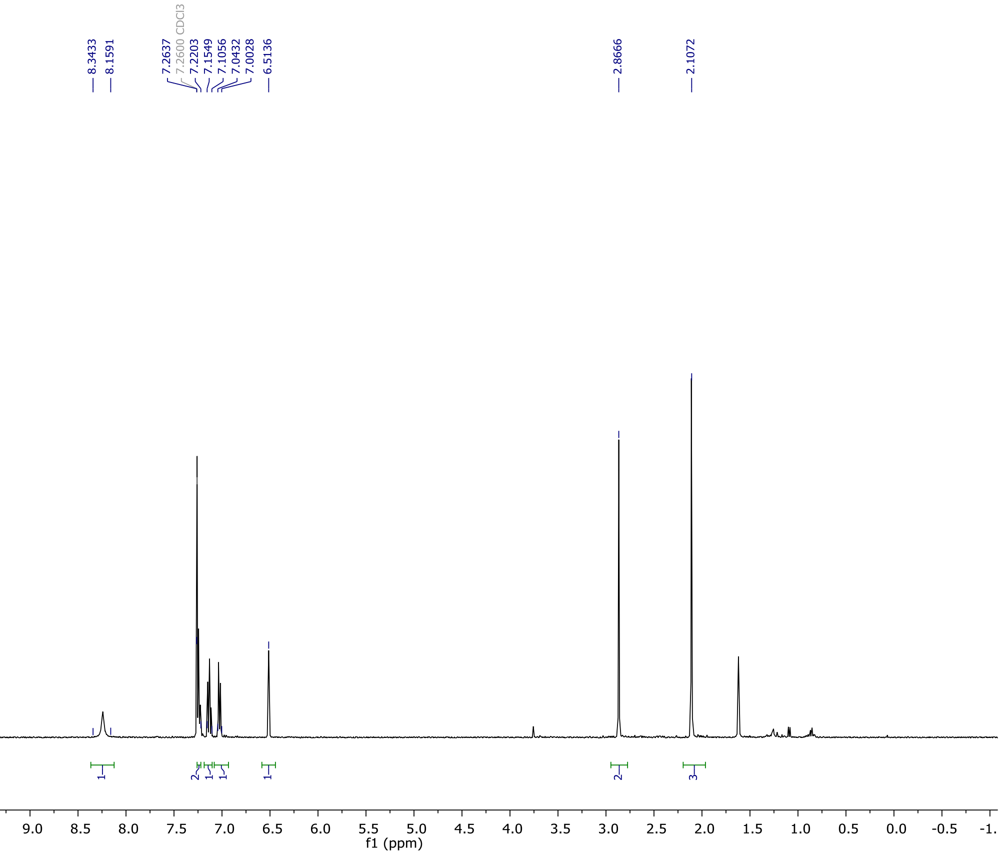

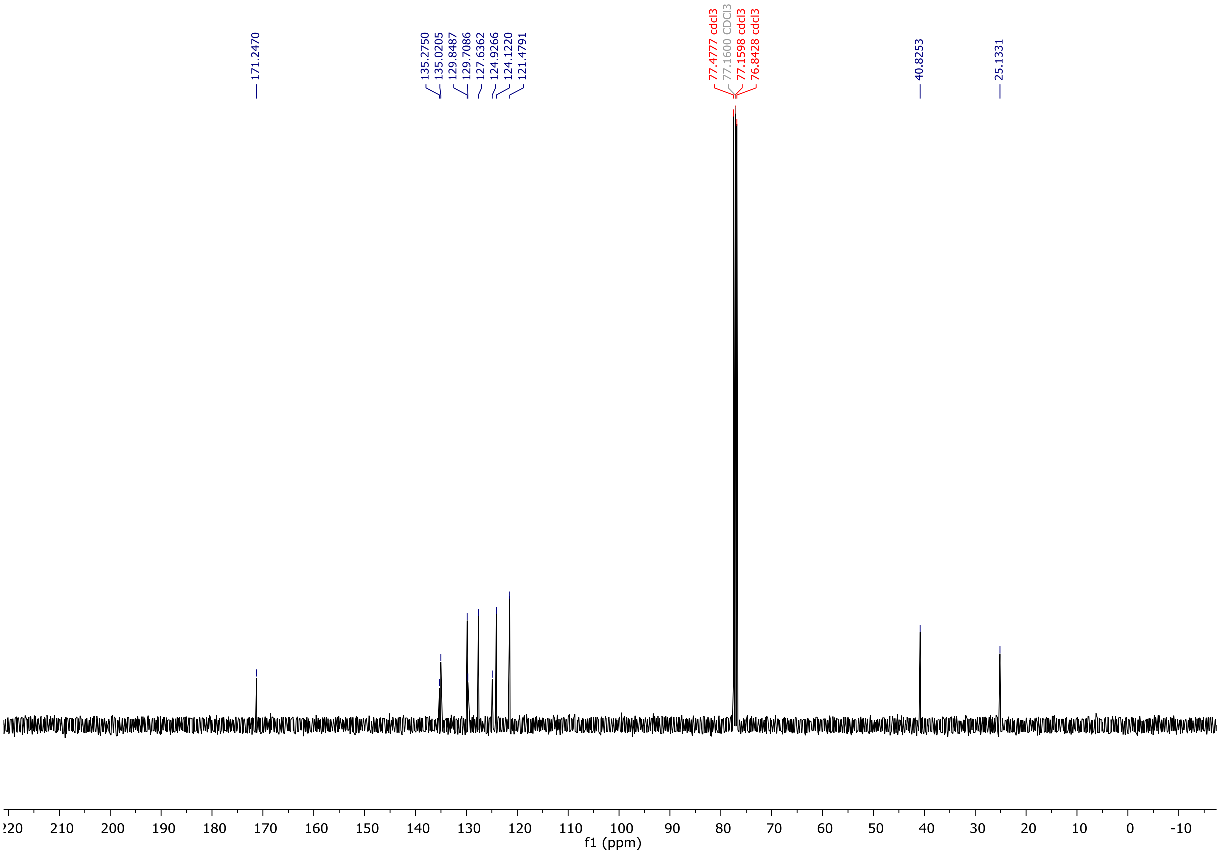

4-Methyl-1,3,4,5-tetrahydro-2*H*-benzo[*b*]azepin-2-one (2h')

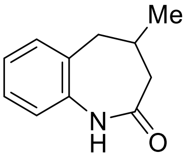

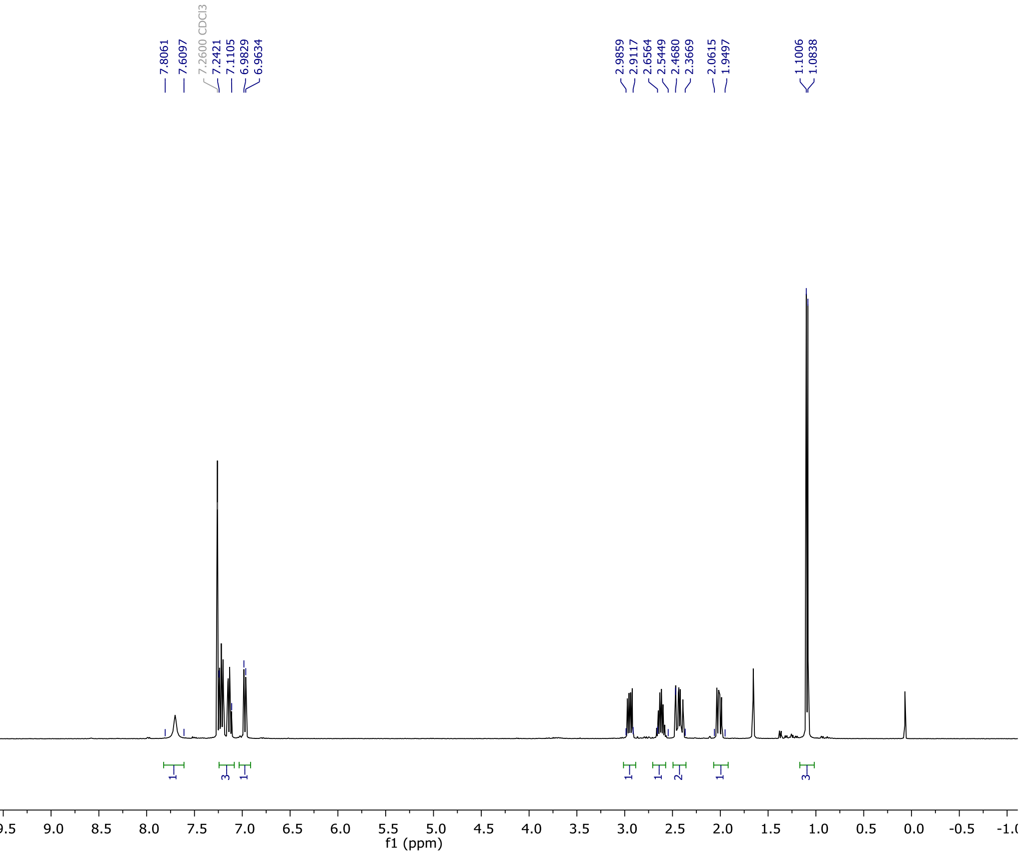

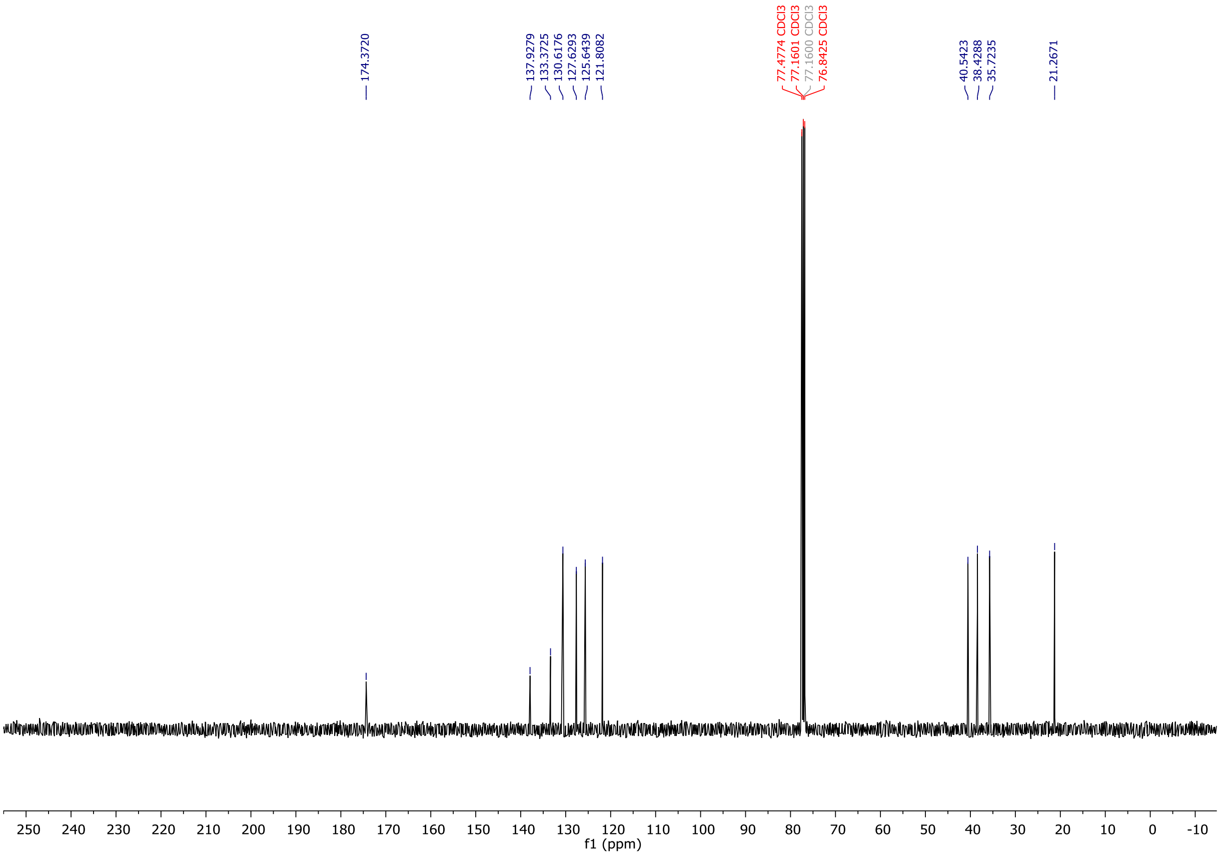

1-Phenyl-1,3,6,7-tetrahydro-2*H*-azepin-2-one (2i)

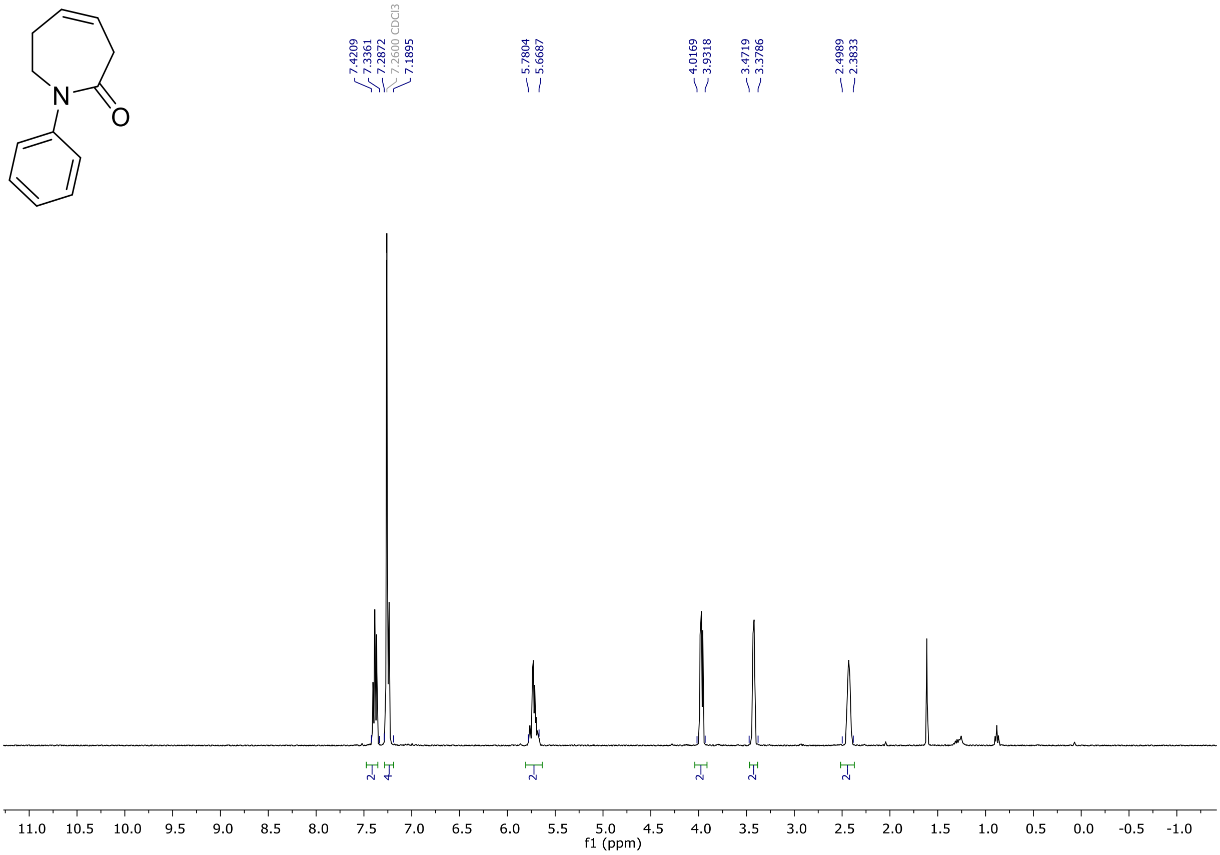

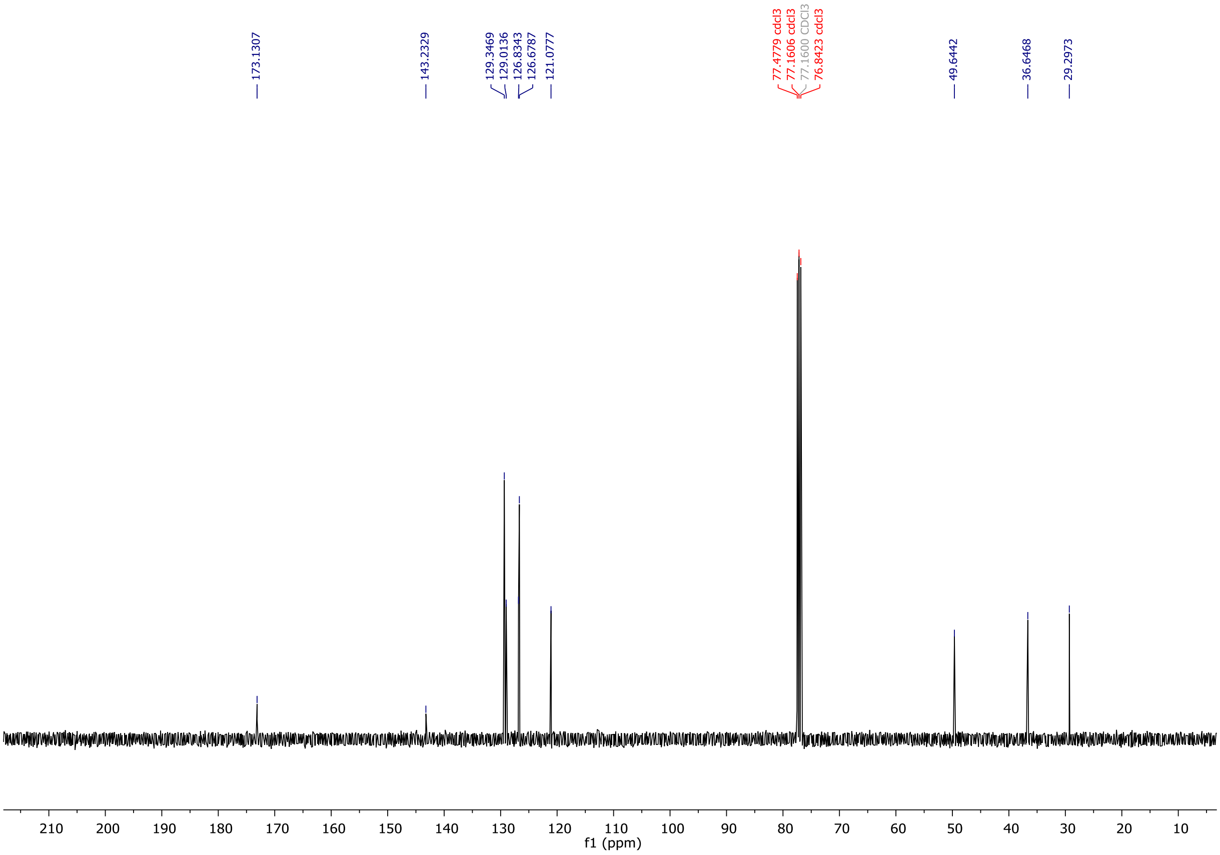

1-Phenylazepan-2-one (2i’)

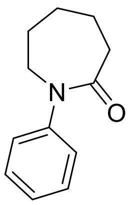

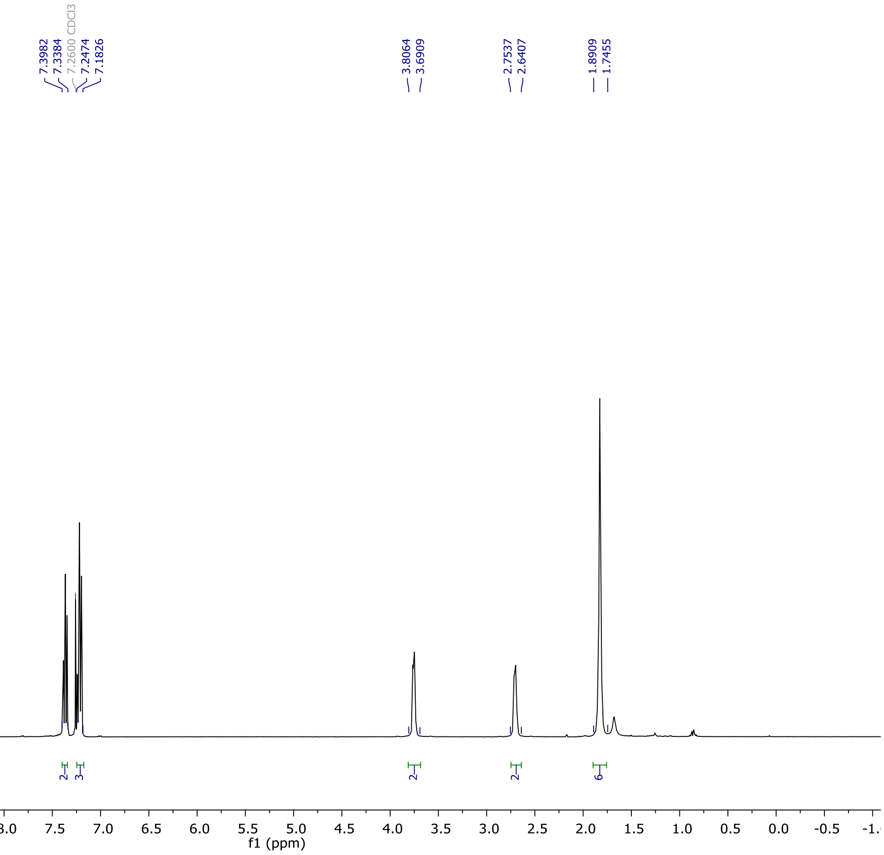

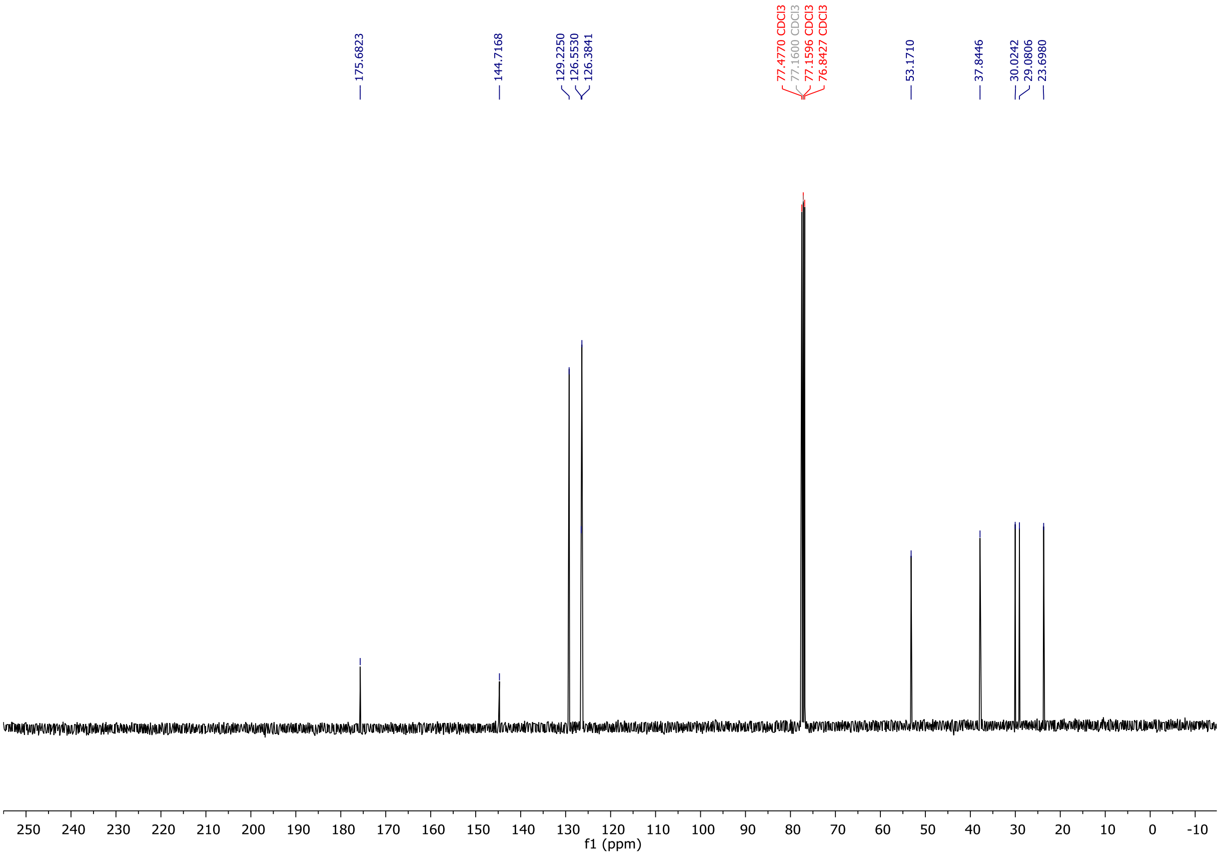

1,6-Diphenyl-1,3,6,7-tetrahydro-2*H*-azepin-2-one (2j)

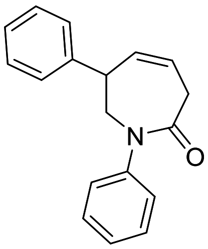

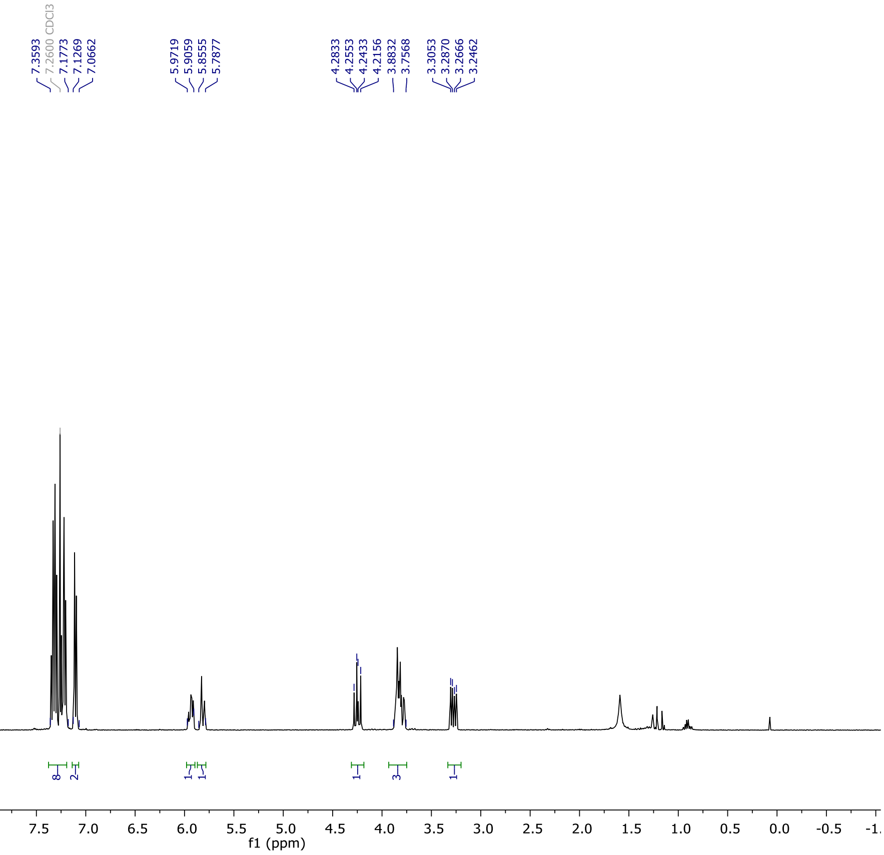

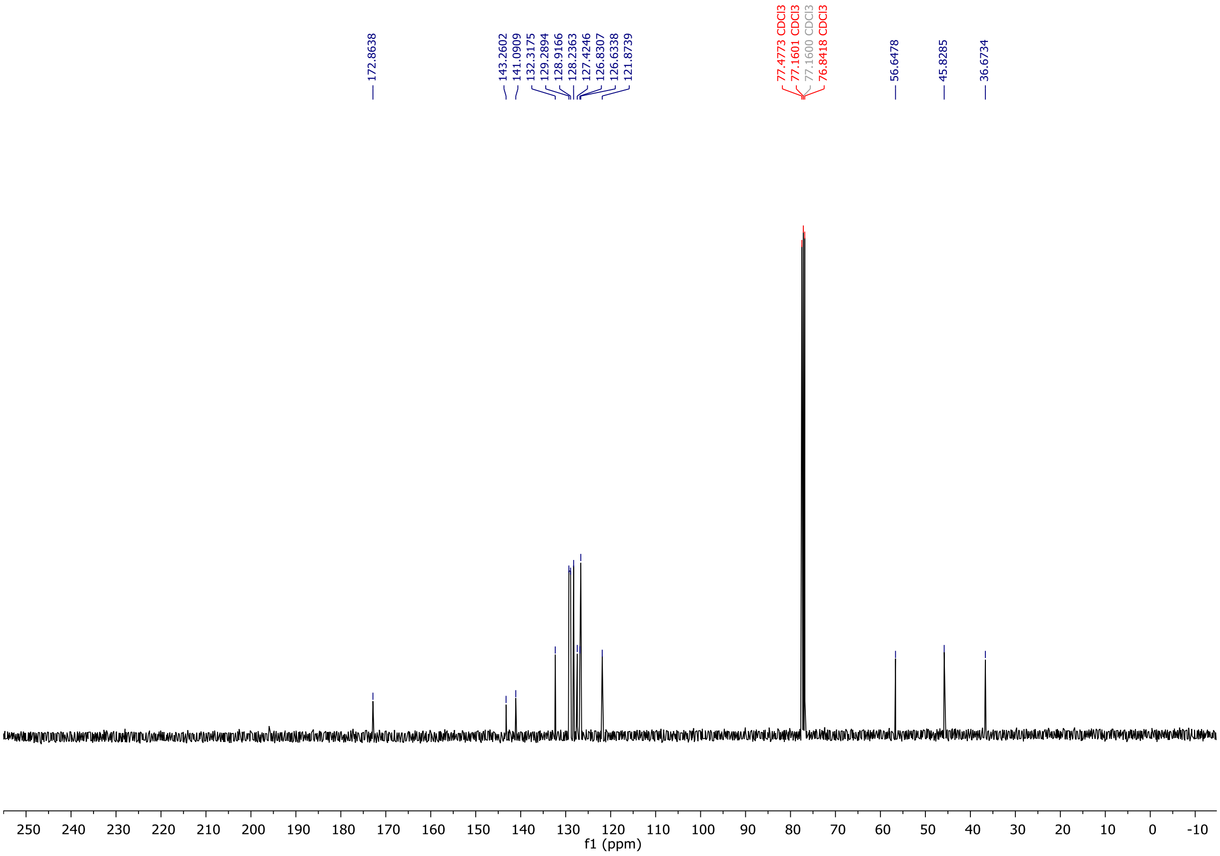

1,6-Diphenylazepan-2-one (2j’)

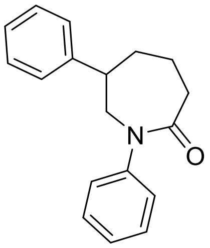

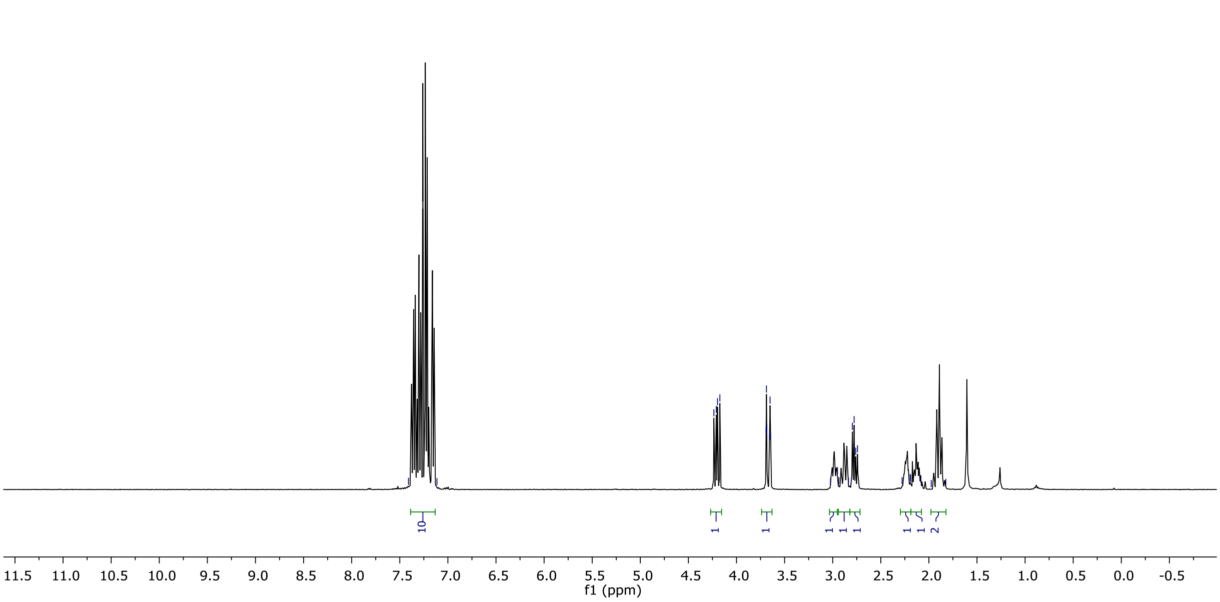

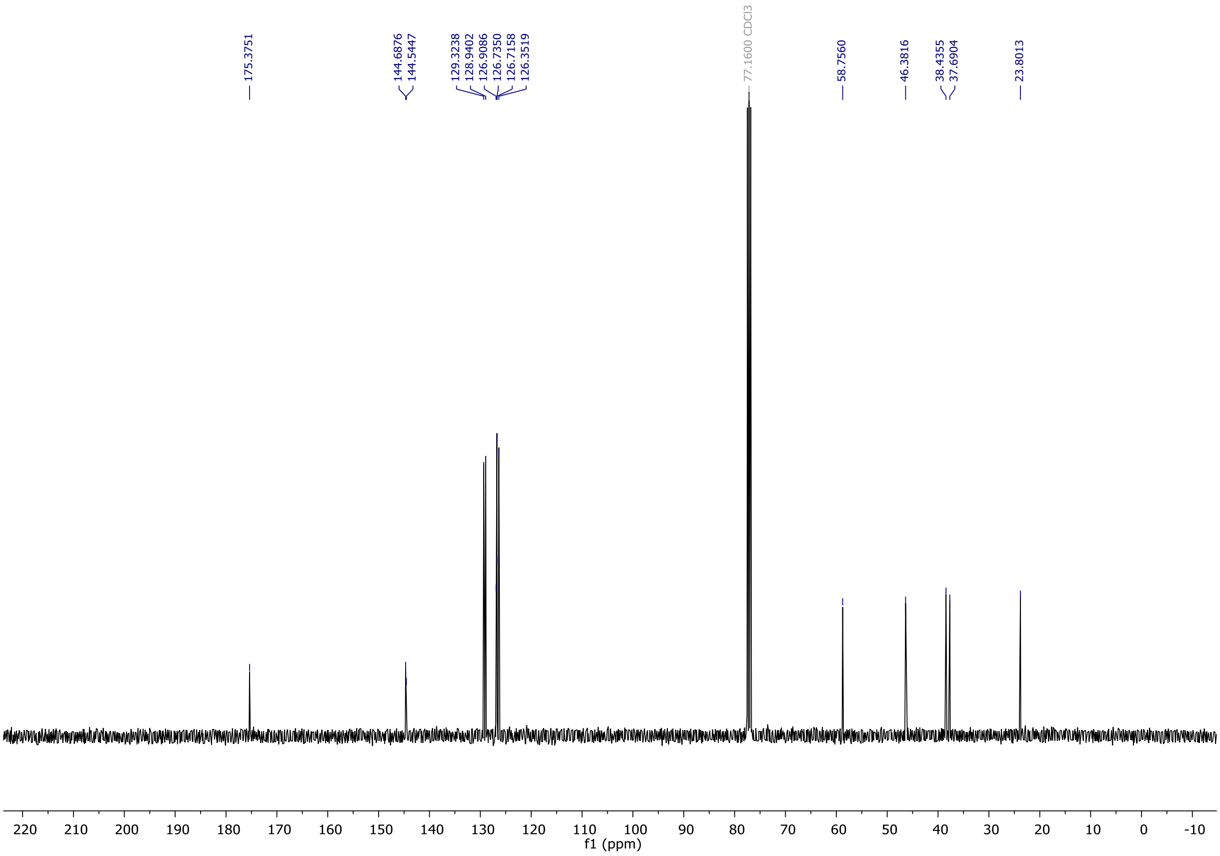

7-Ethyl-1-phenyl-1,3,6,7-tetrahydro-2*H*-azepin-2-one (2k)

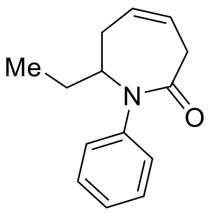

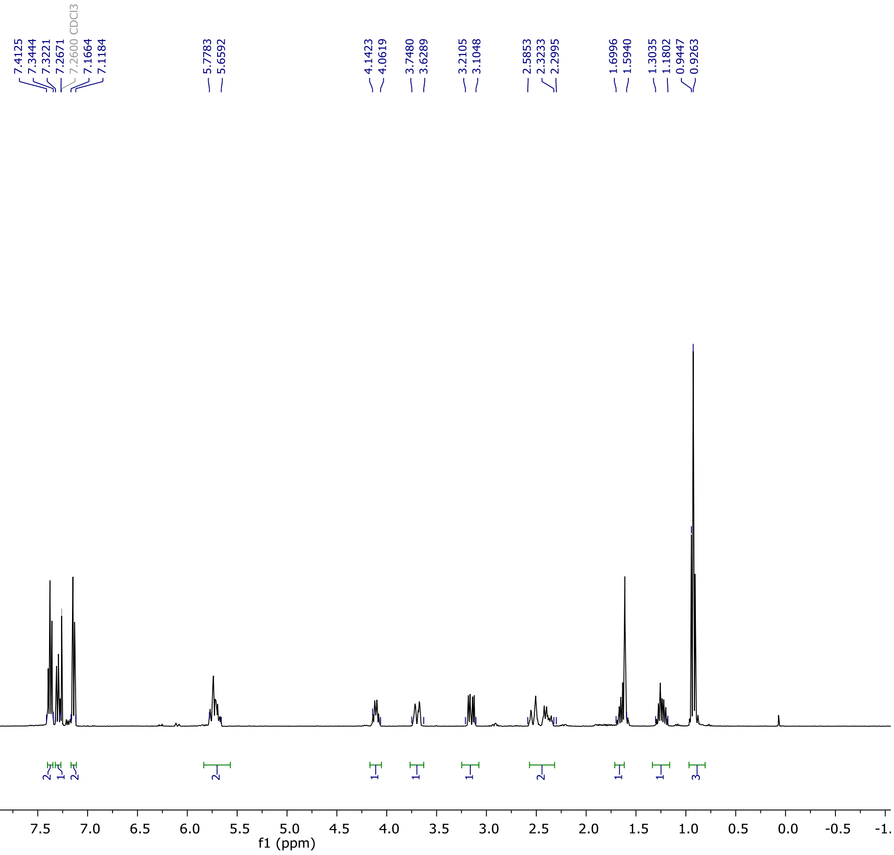

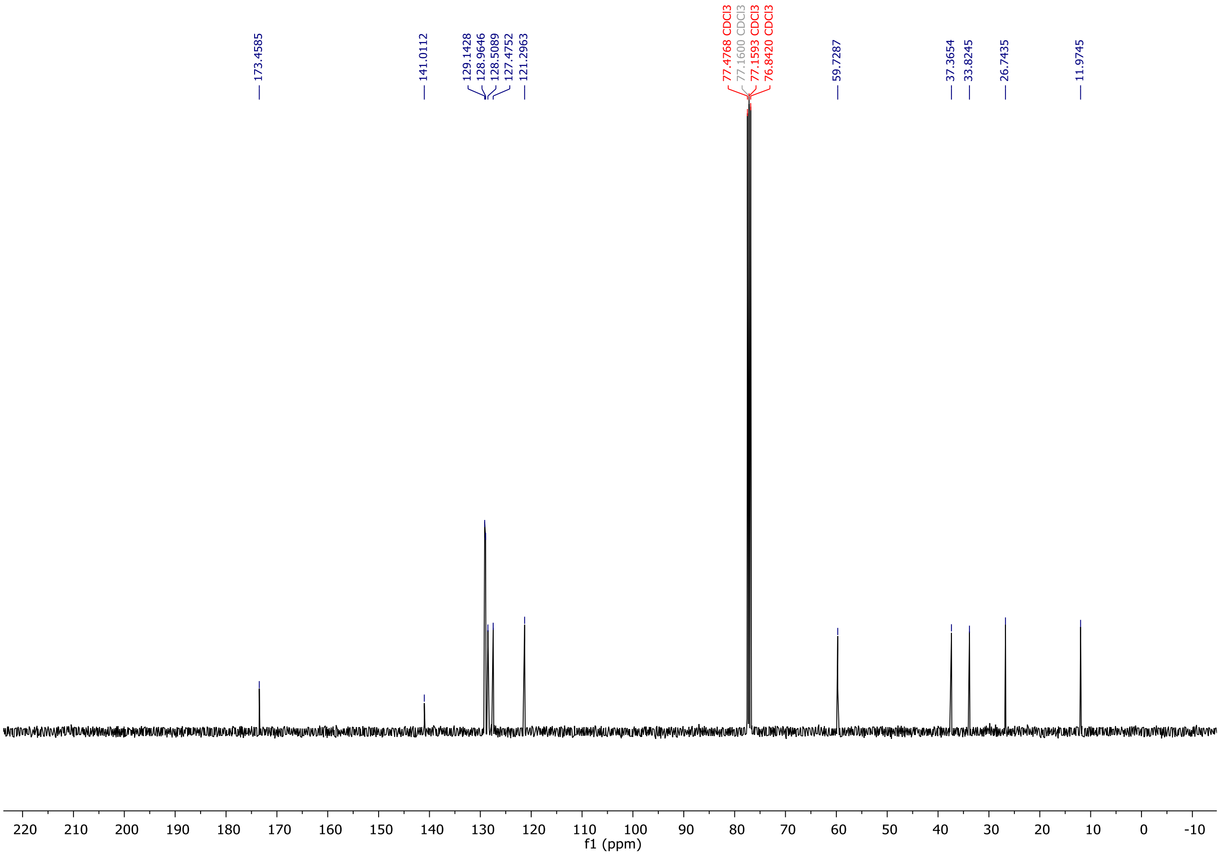

7-Ethyl-1-phenylazepan-2-one (2k’)

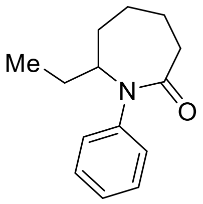

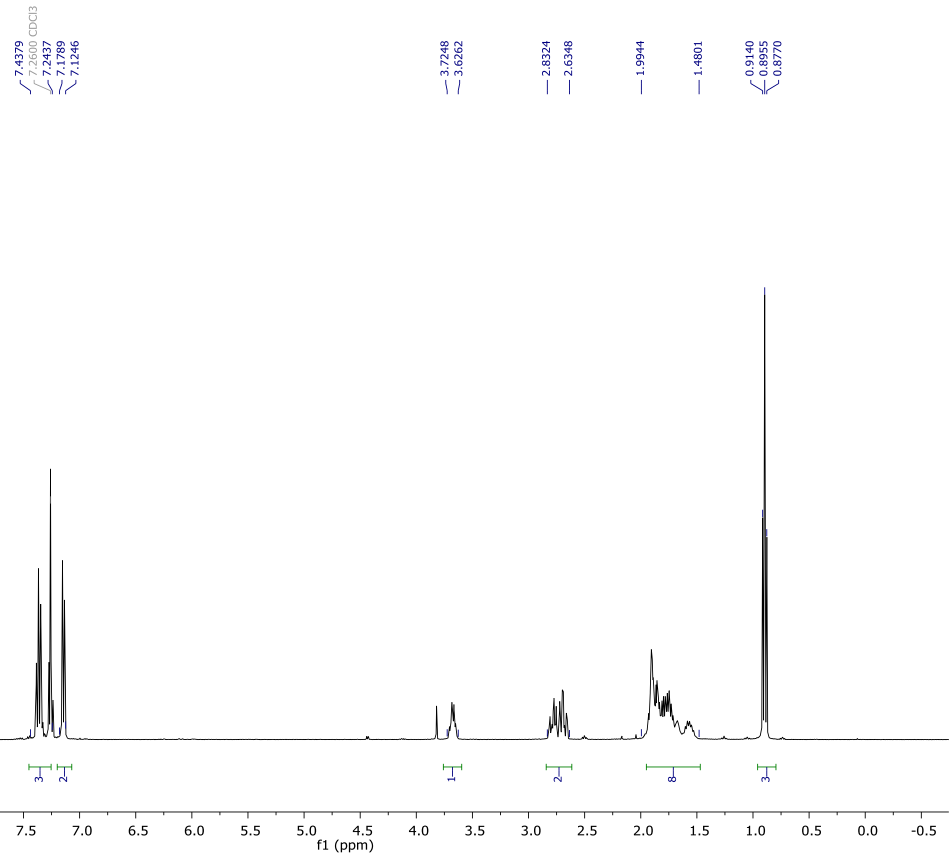

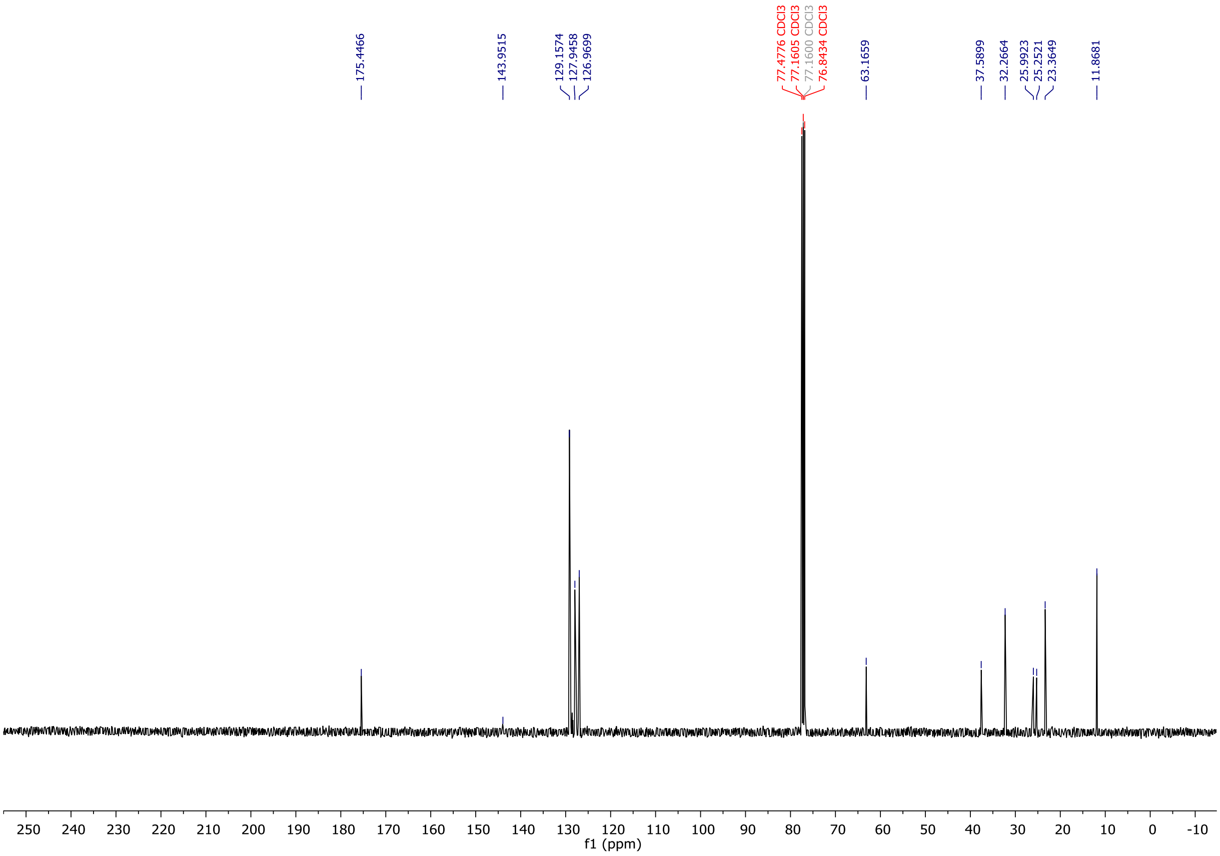

1,7-Diphenyl-1,3,6,7-tetrahydro-2*H*-azepin-2-one (2l)

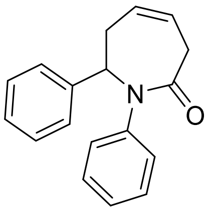

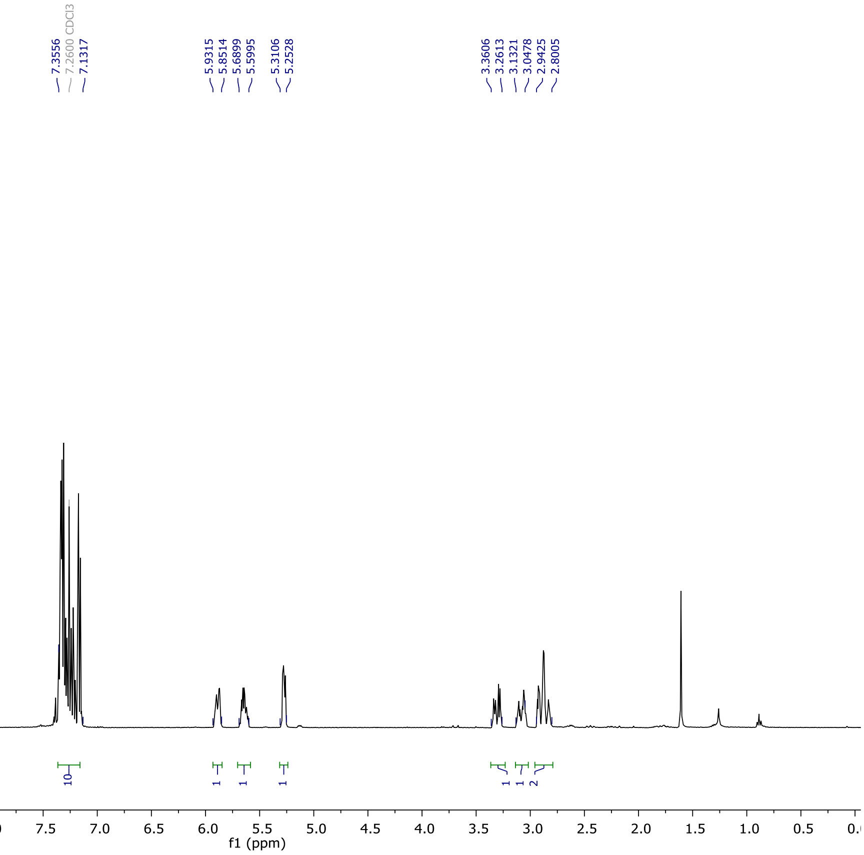

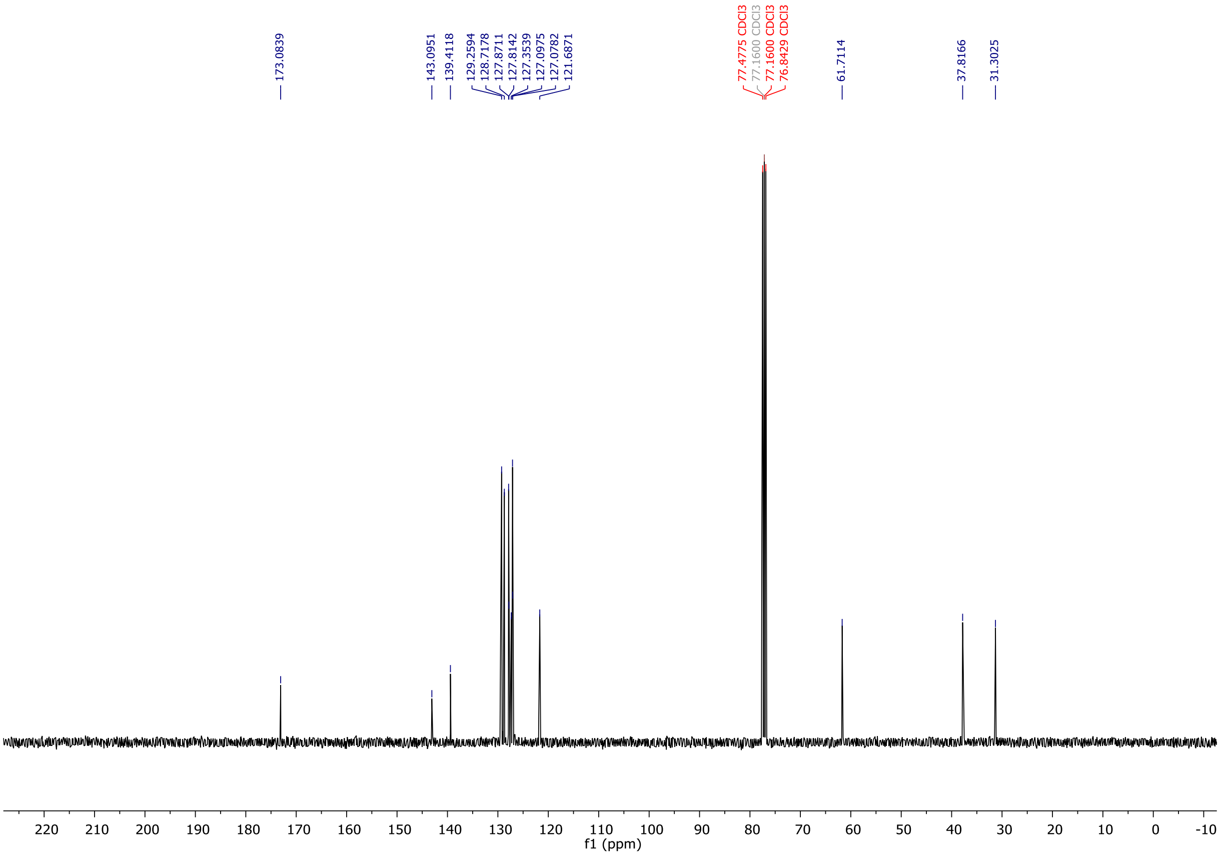

1,7-Diphenylazepan-2-one (2l')

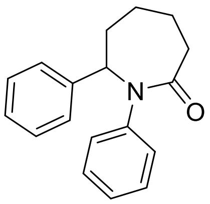

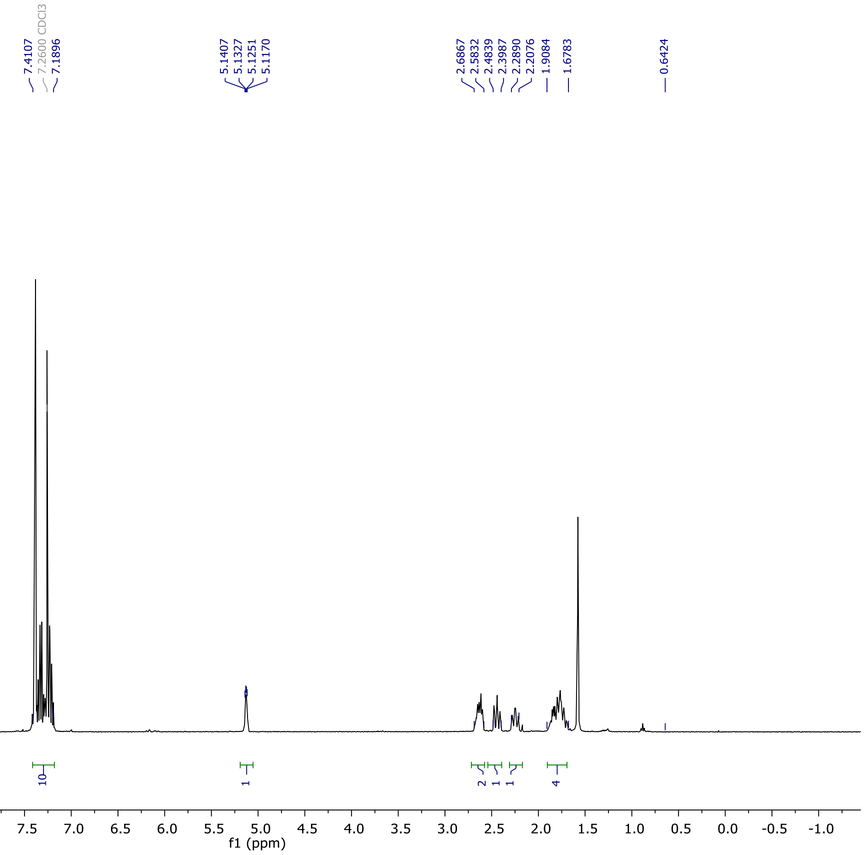

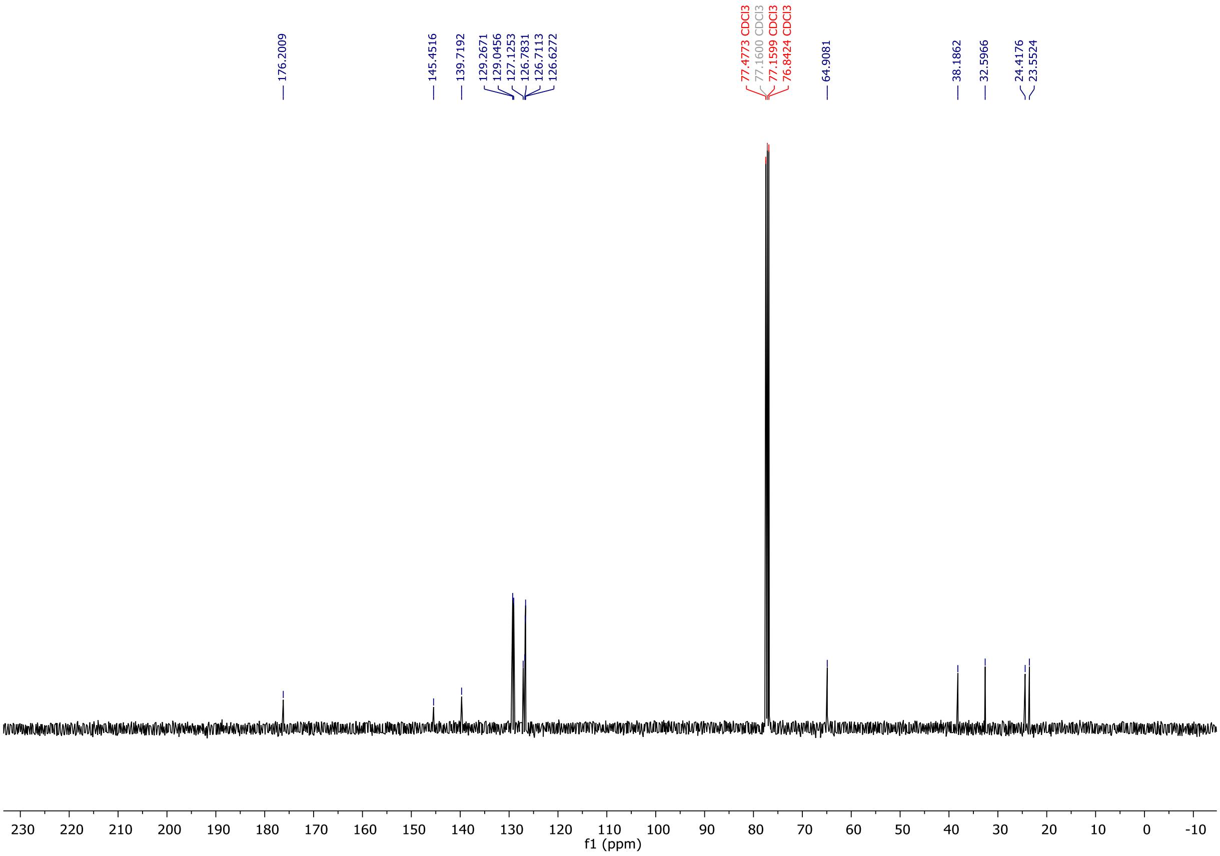

7-(Naphthalen-2-yl)-1-phenyl-1,3,6,7-tetrahydro-2*H*-azepin-2-one (2m)

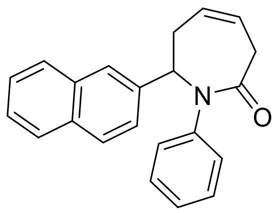

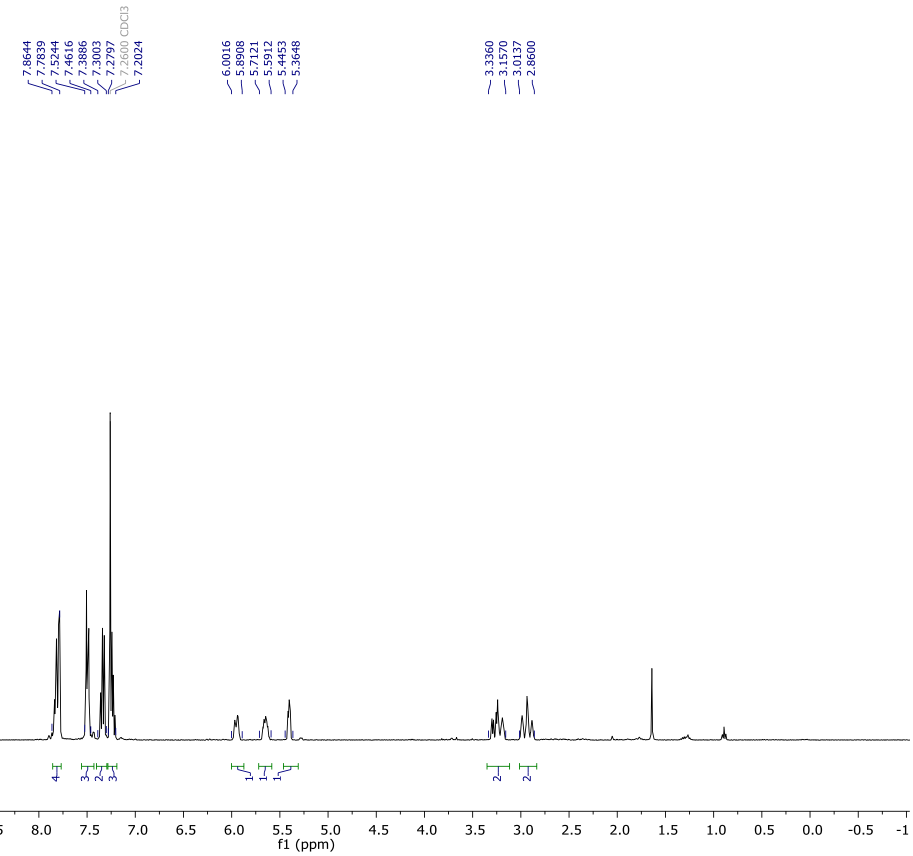

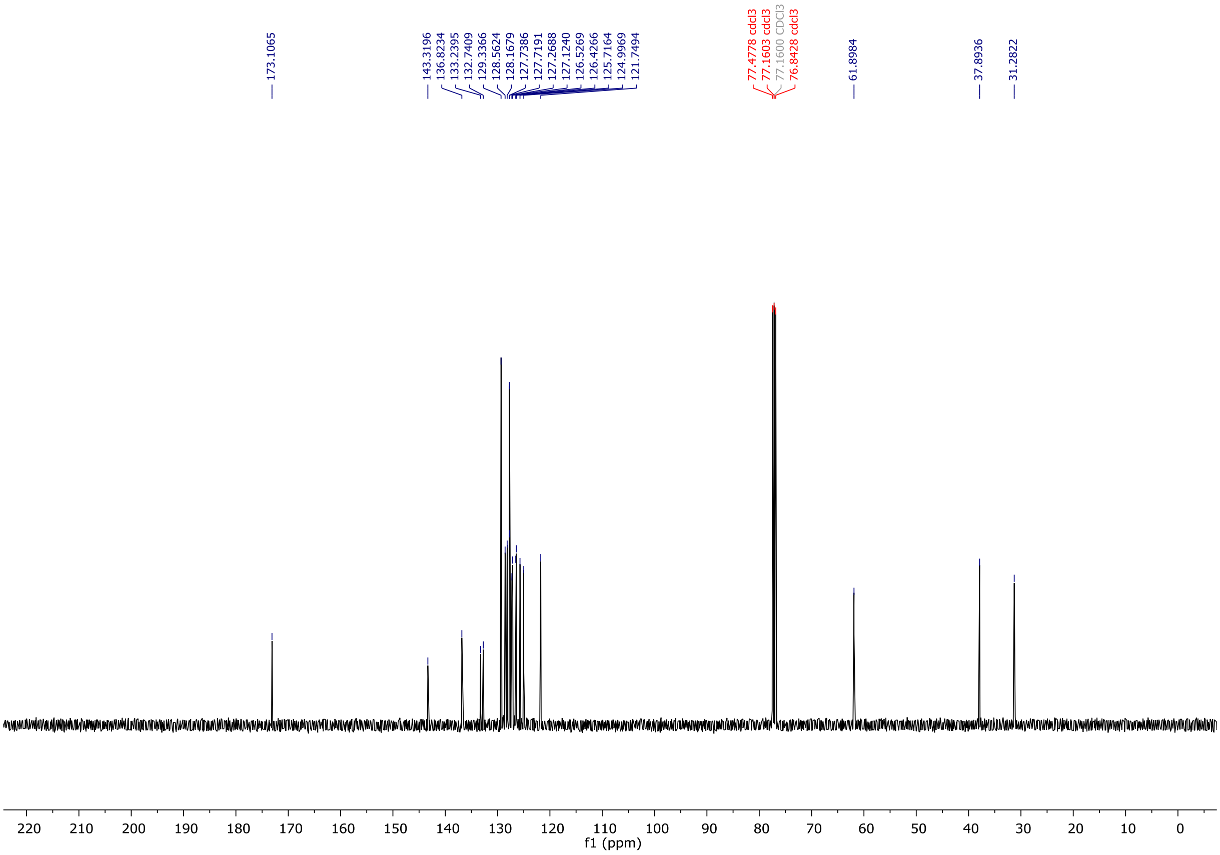

7-(Naphthalen-2-yl)-1-phenylazepan-2-one (2m')

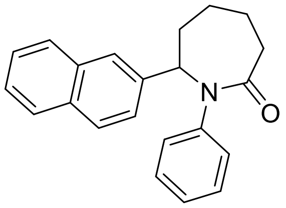

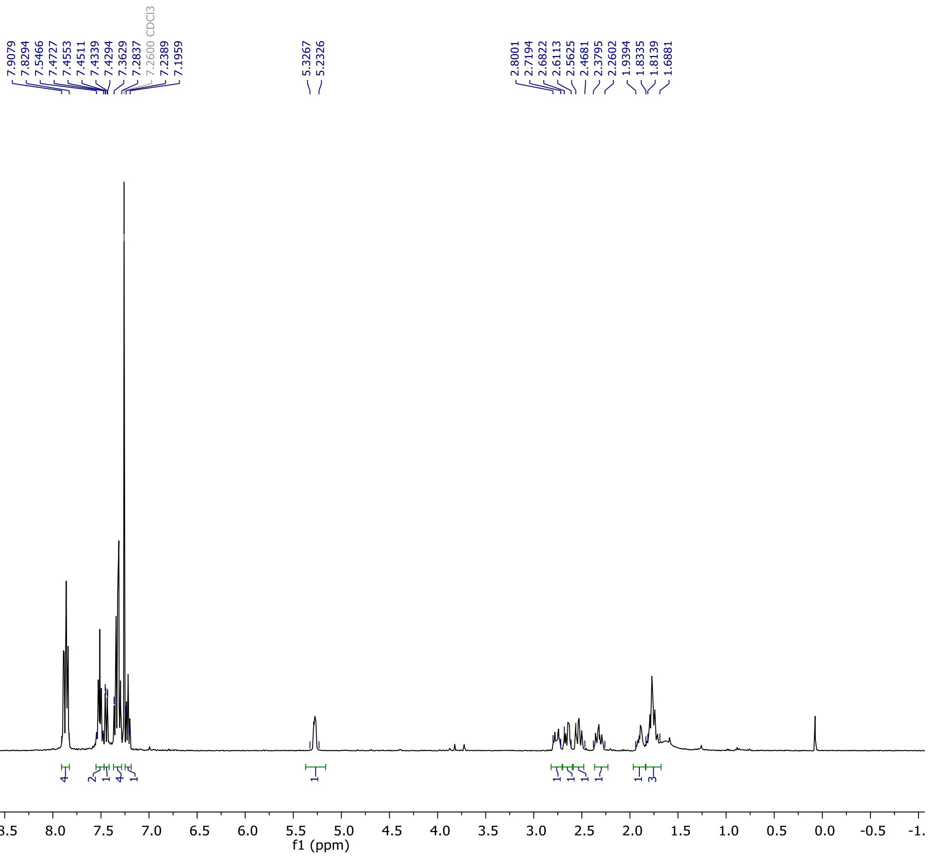

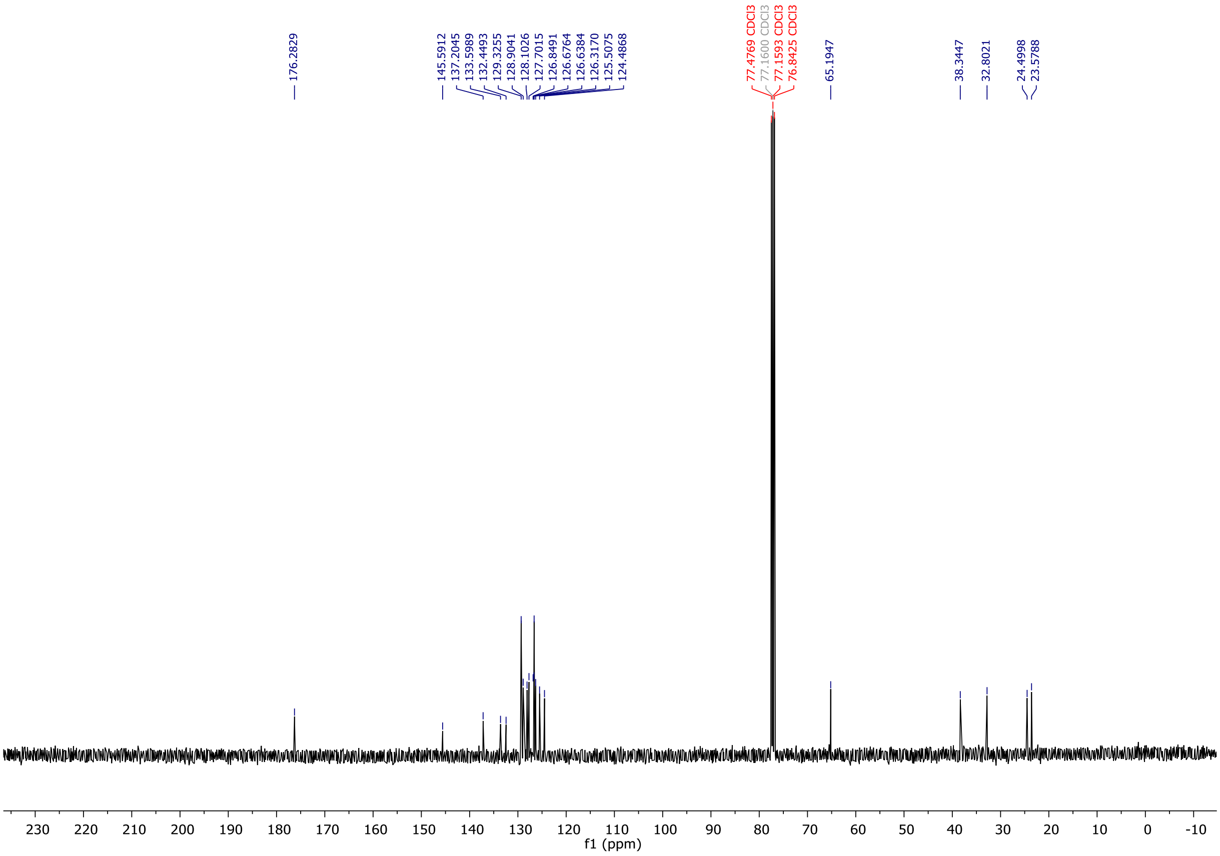

1-Phenyl-7-(thiophen-3-yl)-1,3,6,7-tetrahydro-2*H*-azepin-2-one (2n)

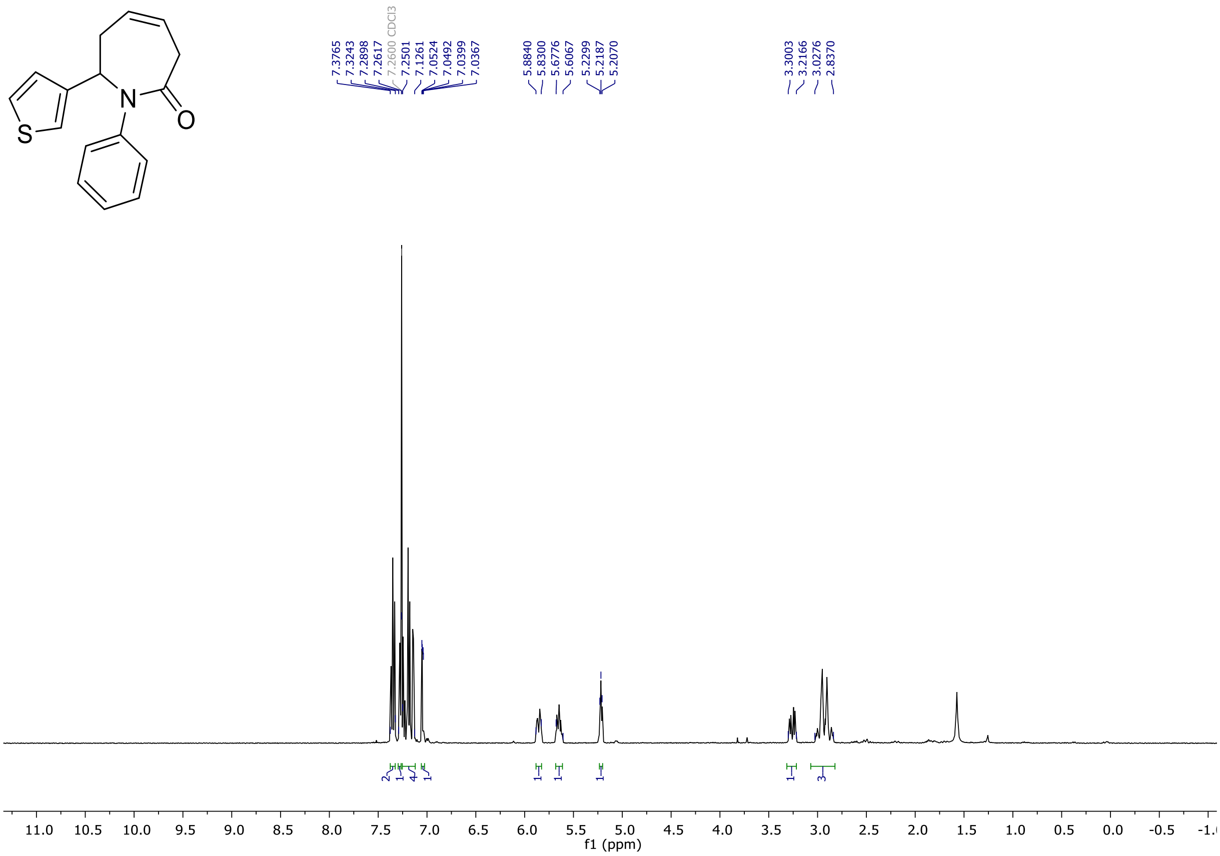

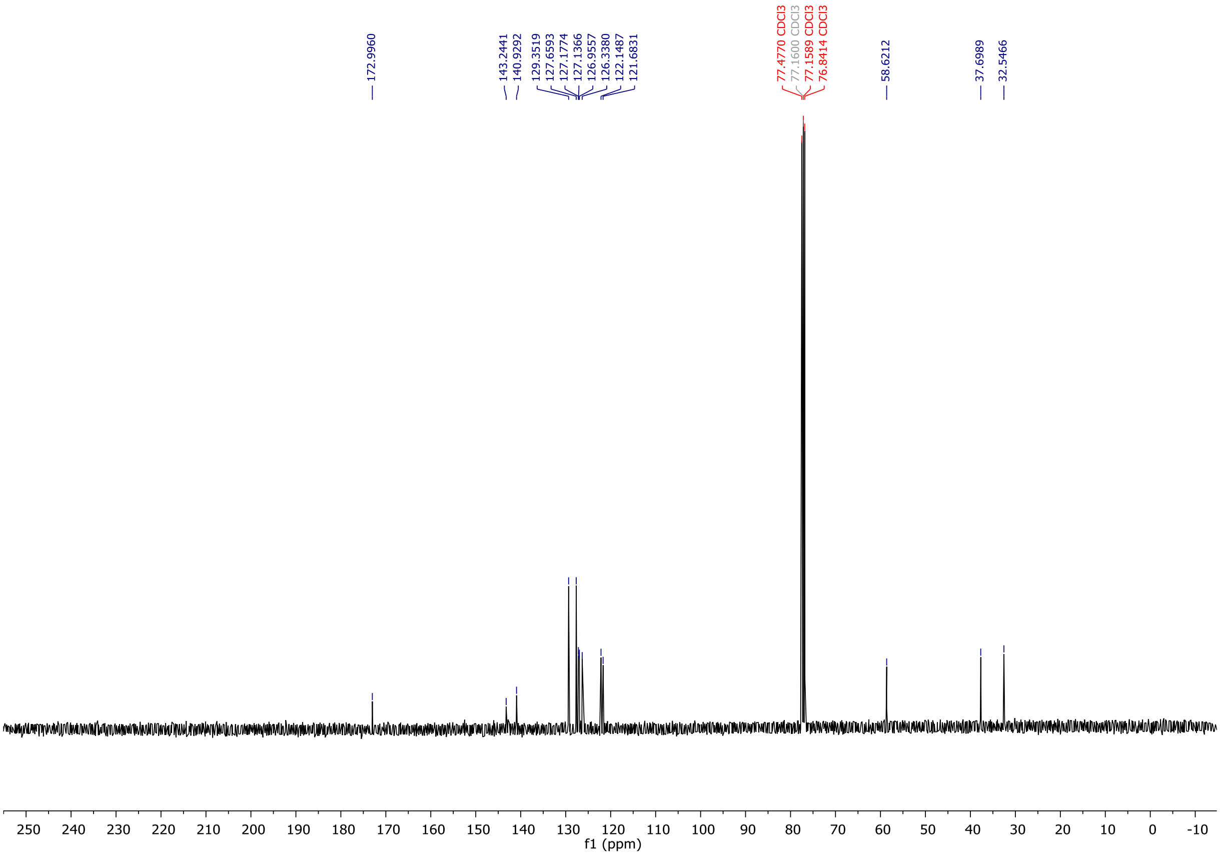

1-Phenyl-7-(thiophen-3-yl)azepan-2-one (2n’)

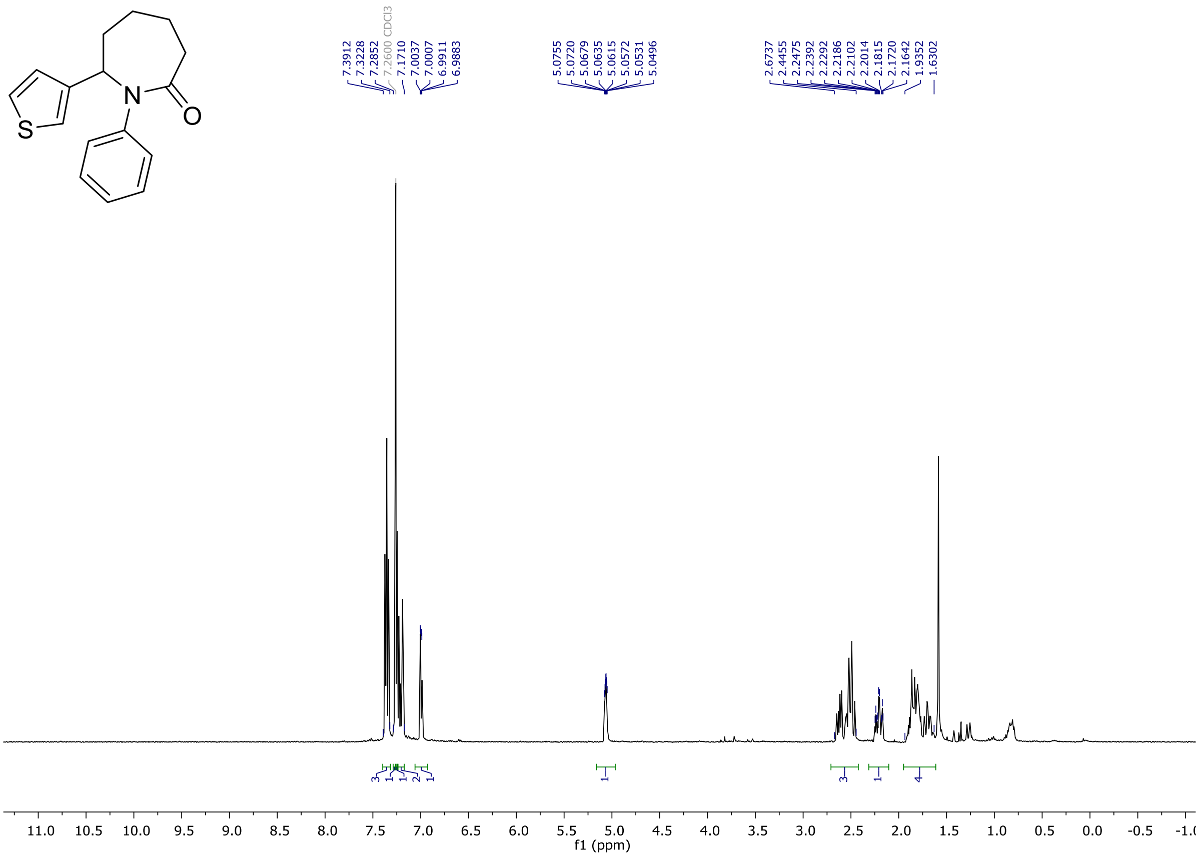

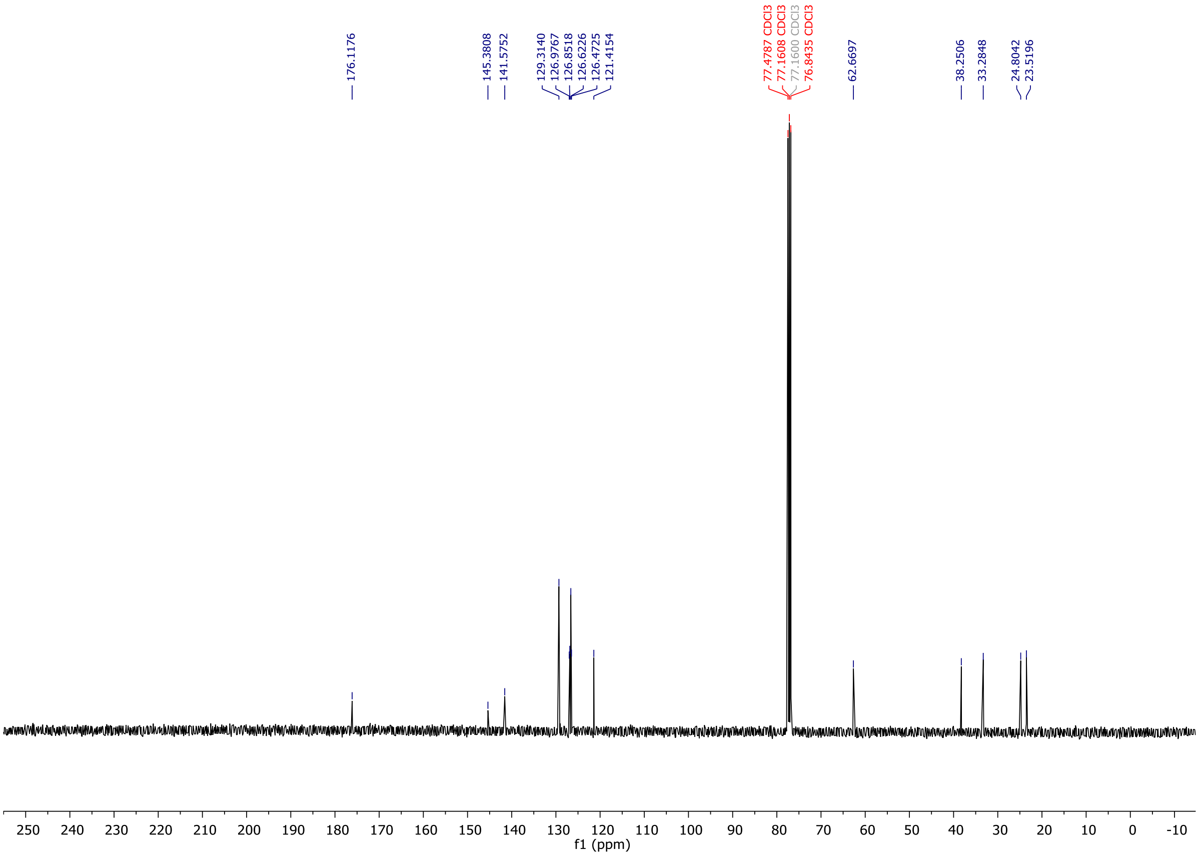

7-Cyclopropyl-1-phenyl-1,3,6,7-tetrahydro-2*H*-azepin-2-one (2o)

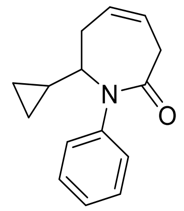

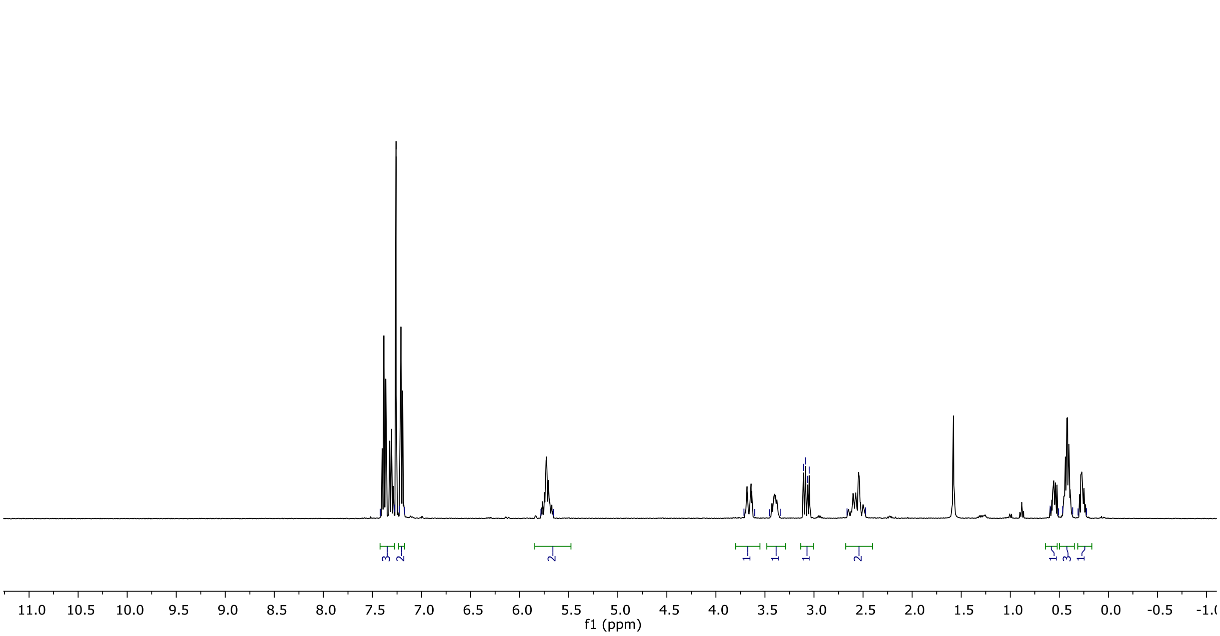

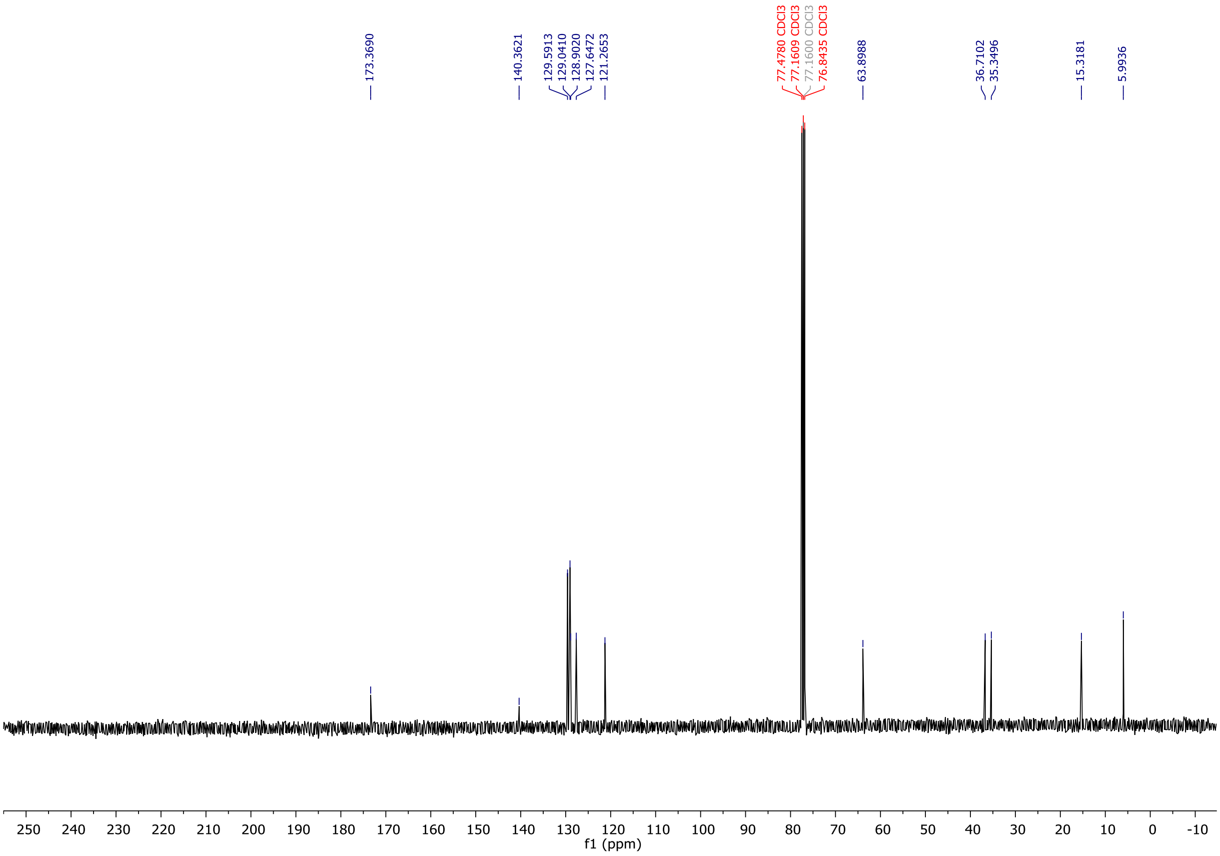

7-Cyclopropyl-1-phenylazepan-2-one (2o')

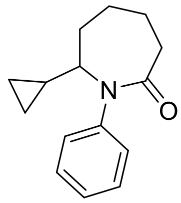

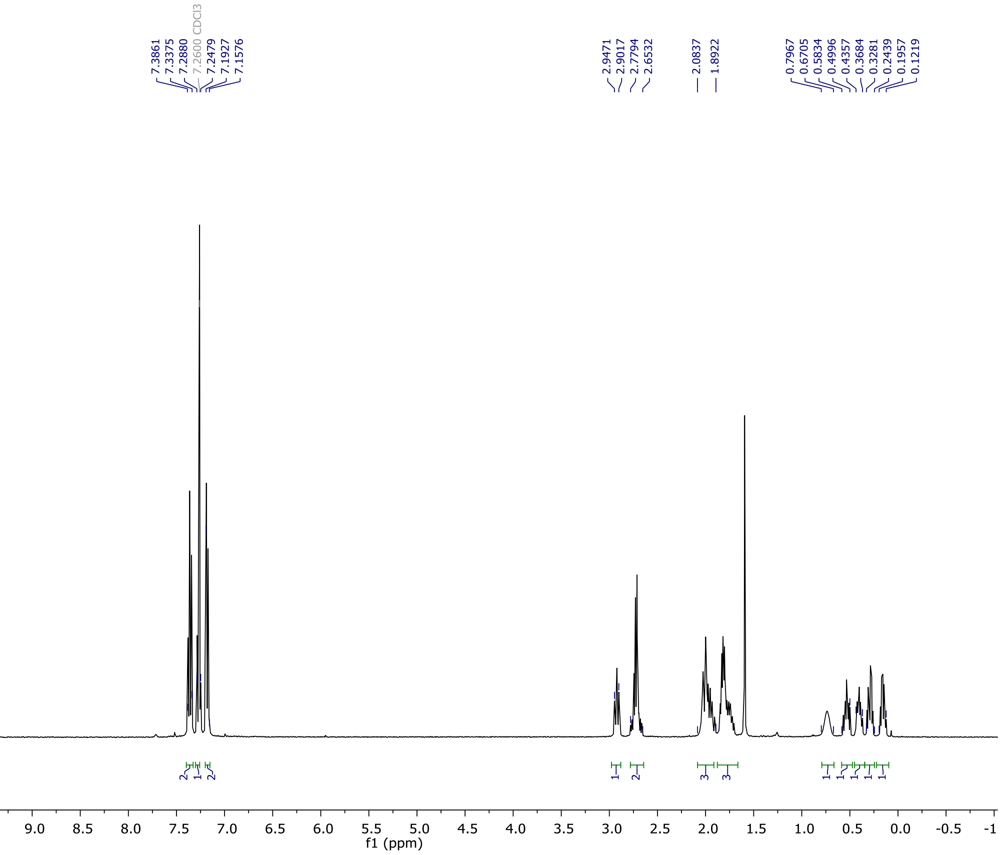

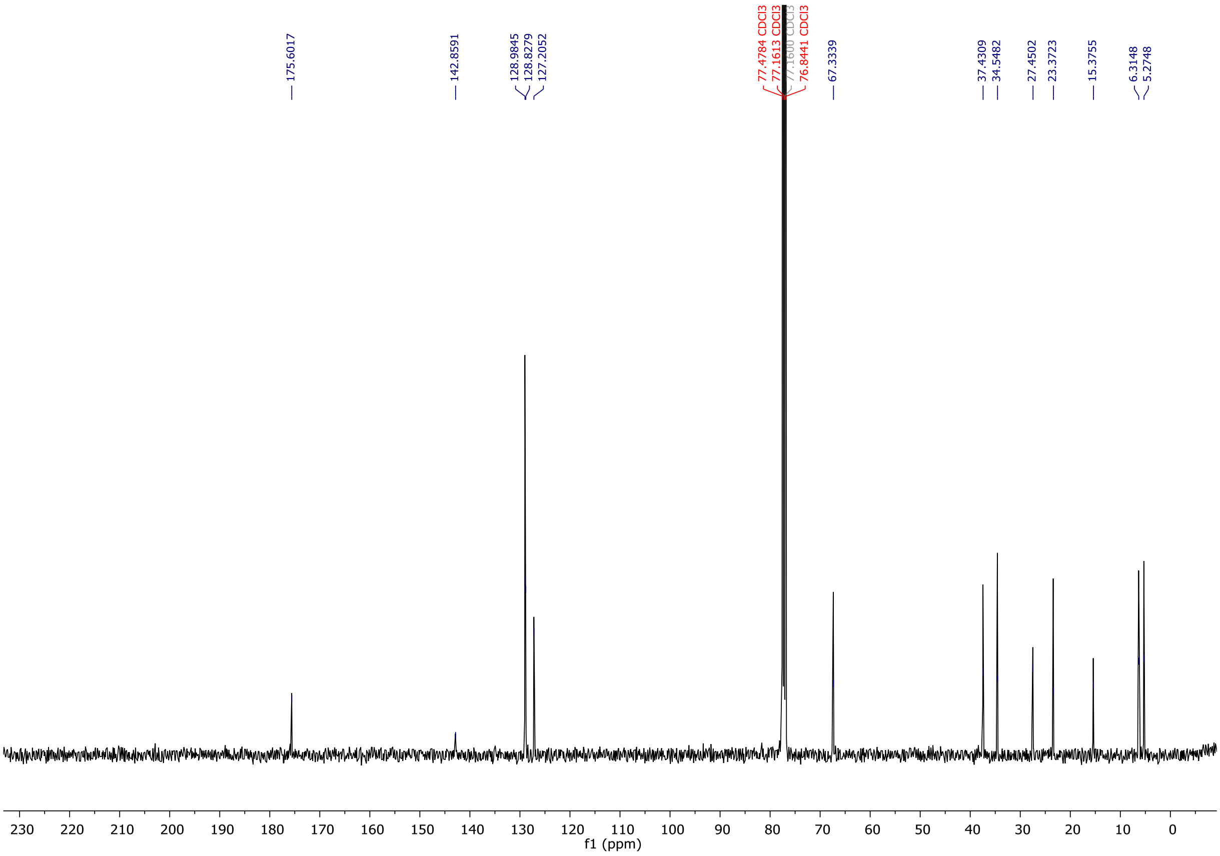

(*rac*)-(*cis*)-1-Phenyl-3,5a,6,7,8,8a-hexahydrocyclopenta[*b*]azepin-2(1*H*)-one (2p)

Mixture of C1-C2 (minor) and C2-C3 (major) isomers.

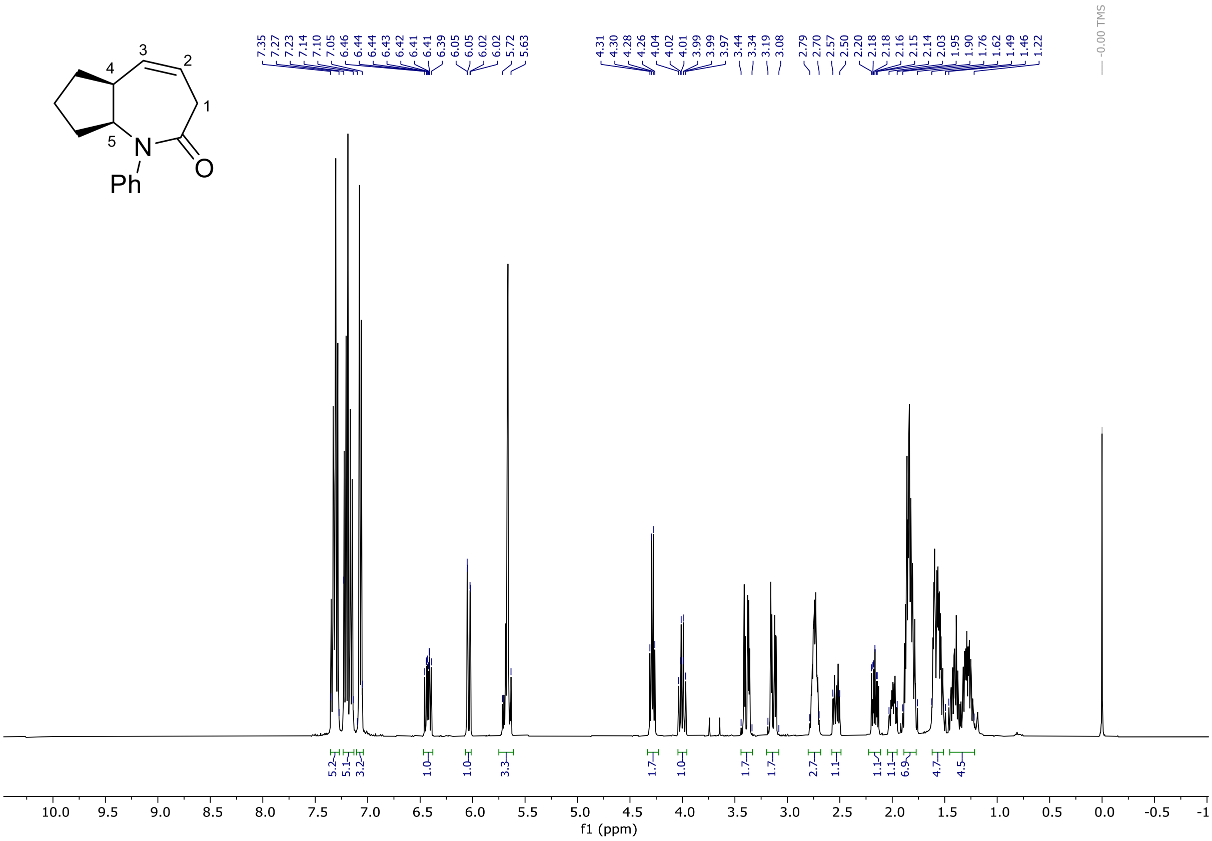

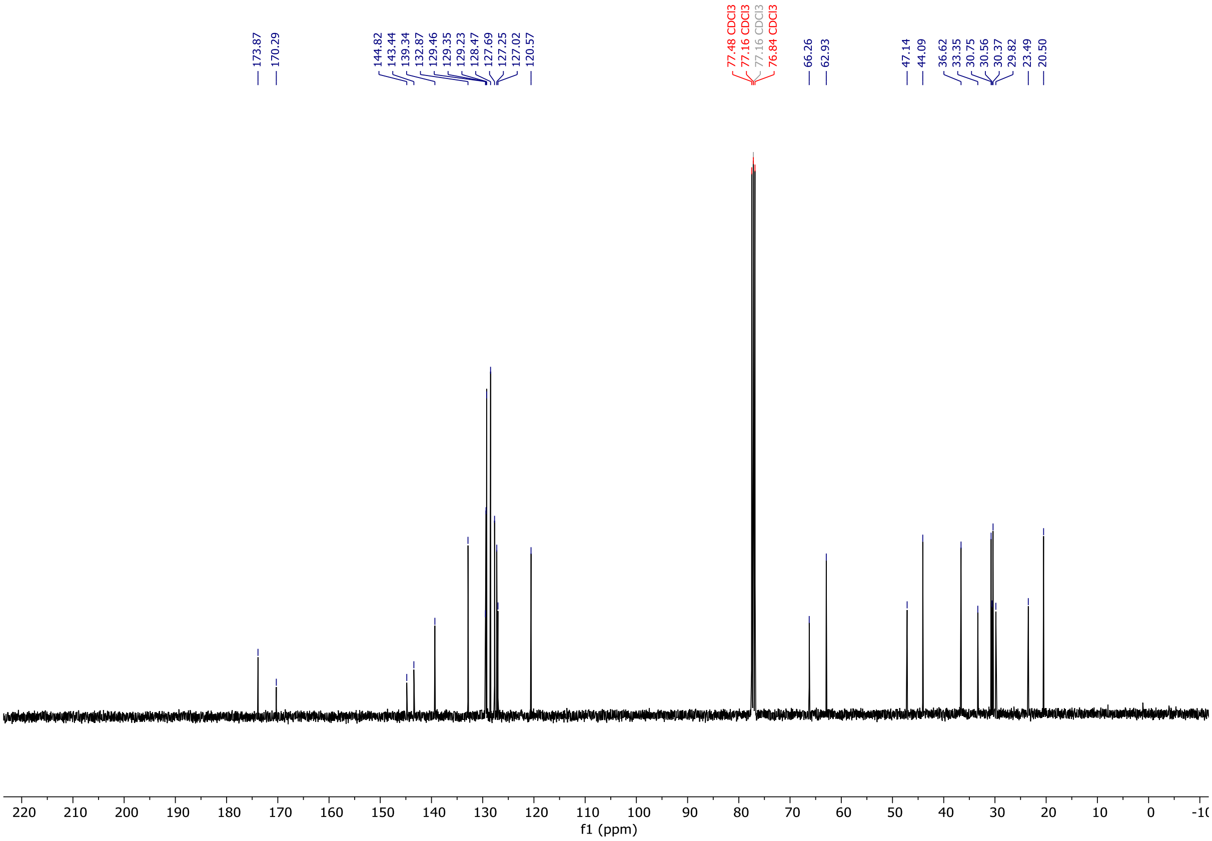

(*rac*)-(*cis*)-1-Phenyloctahydrocyclopenta[*b*]azepin-2(1*H*)-one (2p')

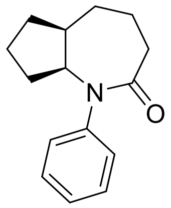

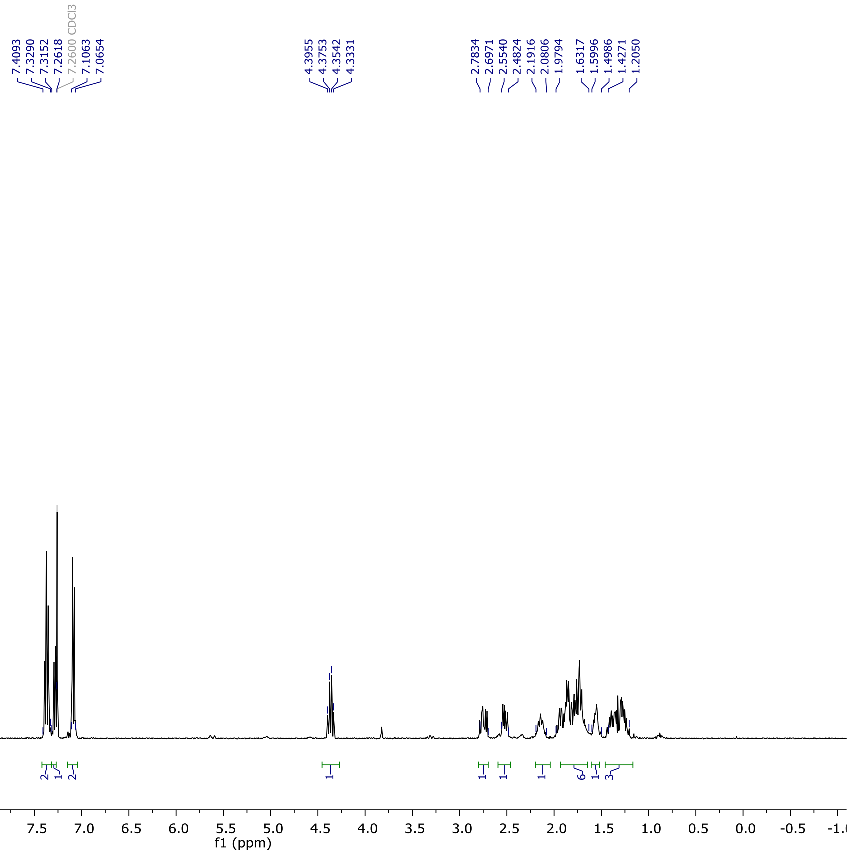

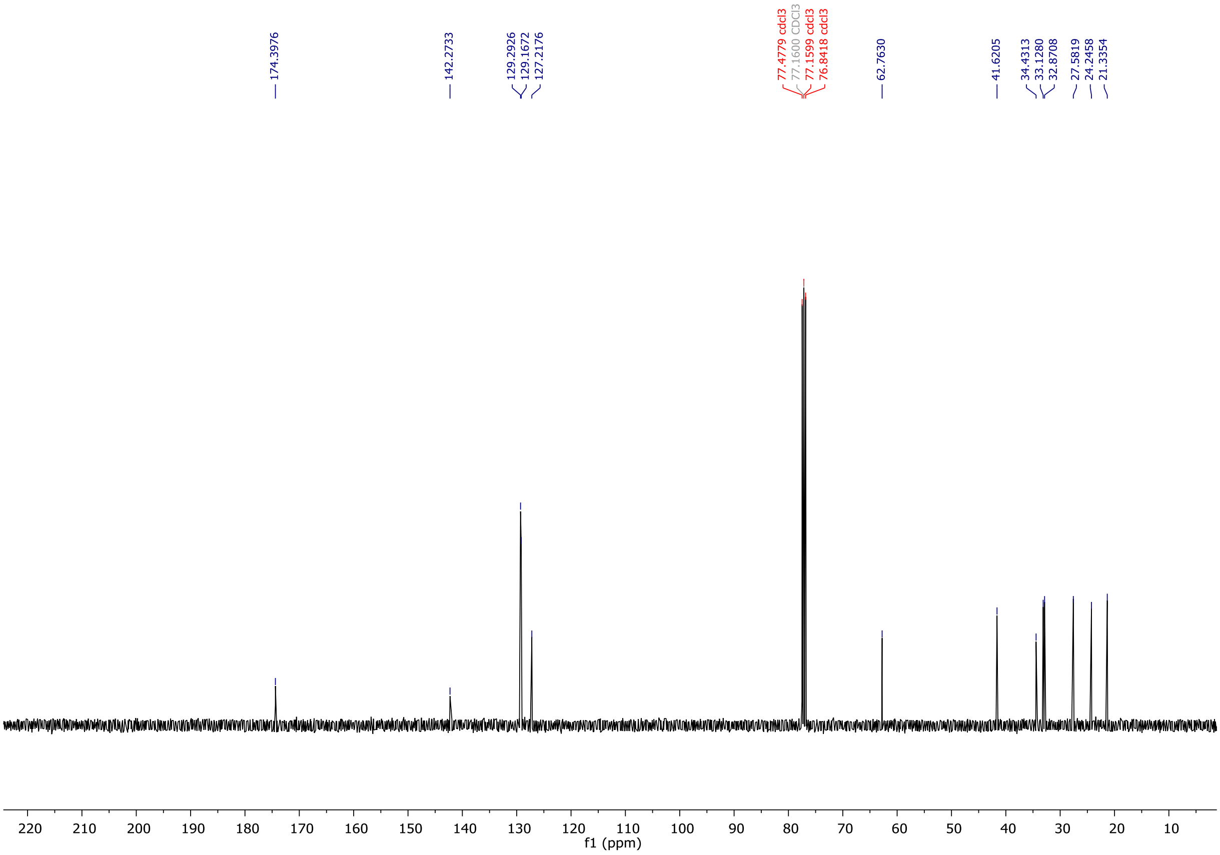

4-Methyl-1-phenyl-1,3,4,5-tetrahydro-2*H*-azepin-2-one (2q)

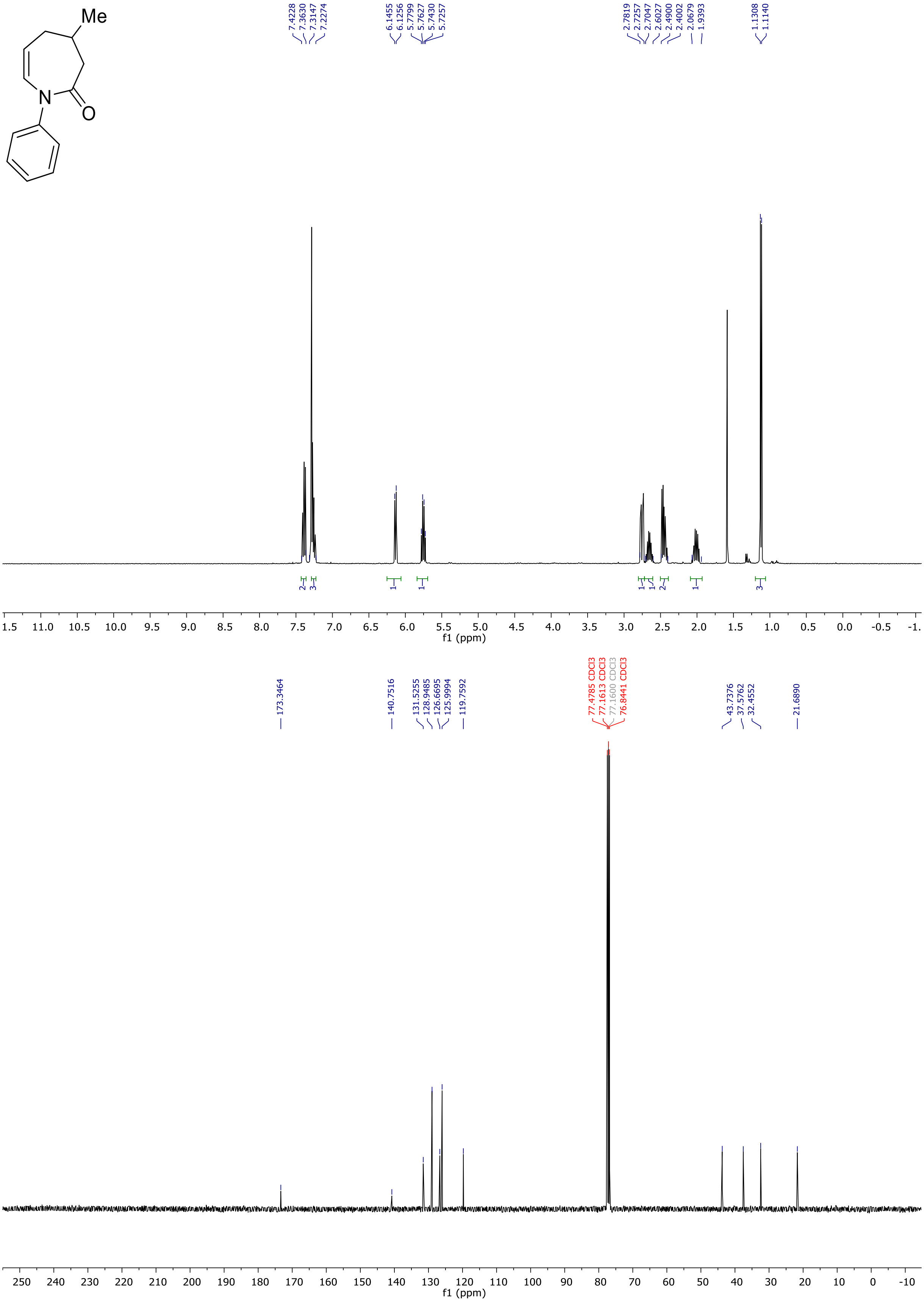

4-Ethyl-1-phenyl-1,3,4,5-tetrahydro-2*H*-azepin-2-one (2r)

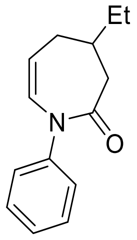

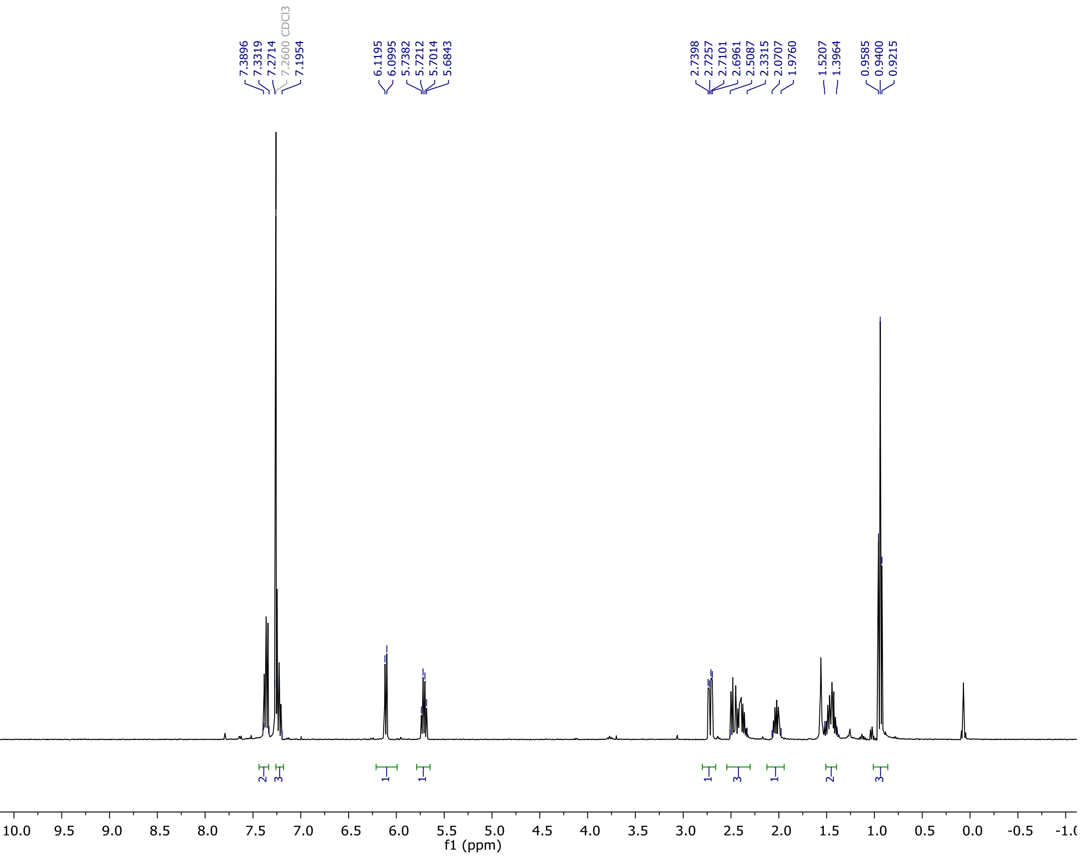

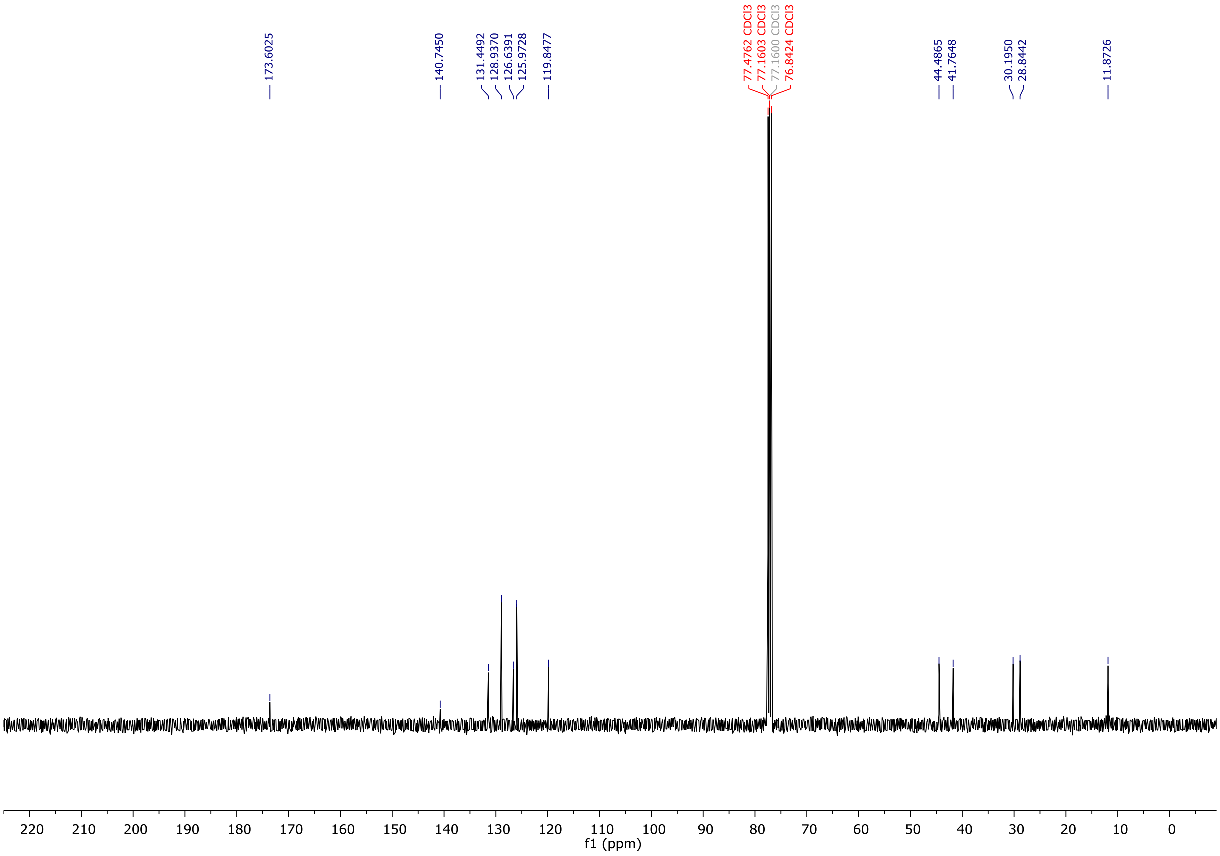

1,4-Diphenyl-1,3,4,5-tetrahydro-2*H*-azepin-2-one (2s)

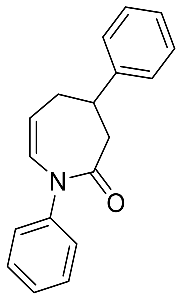

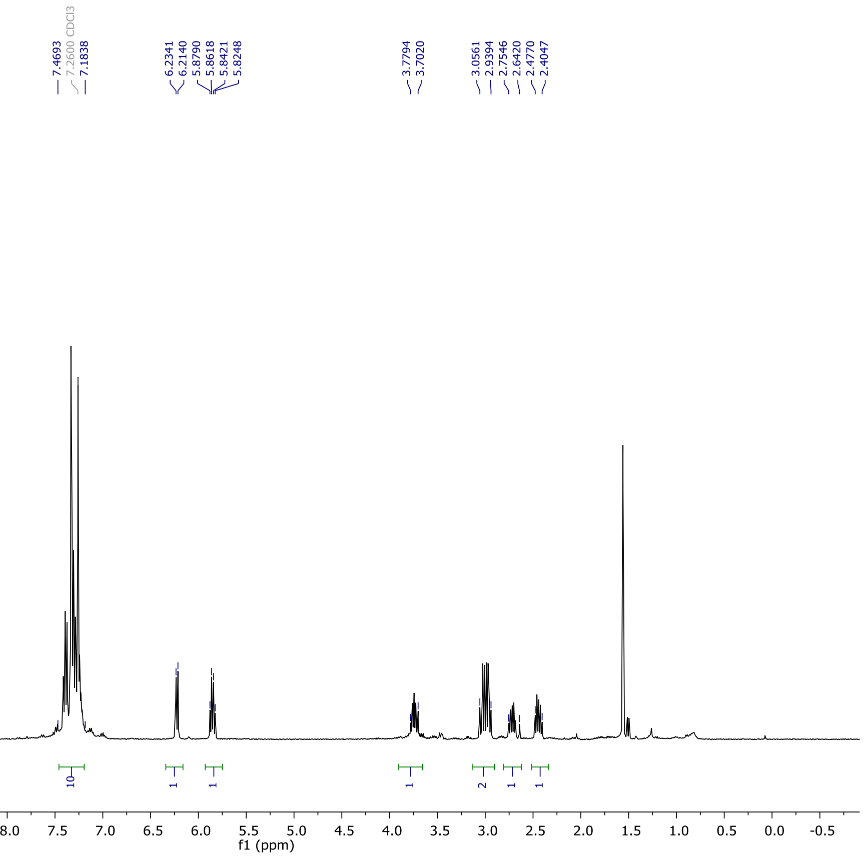

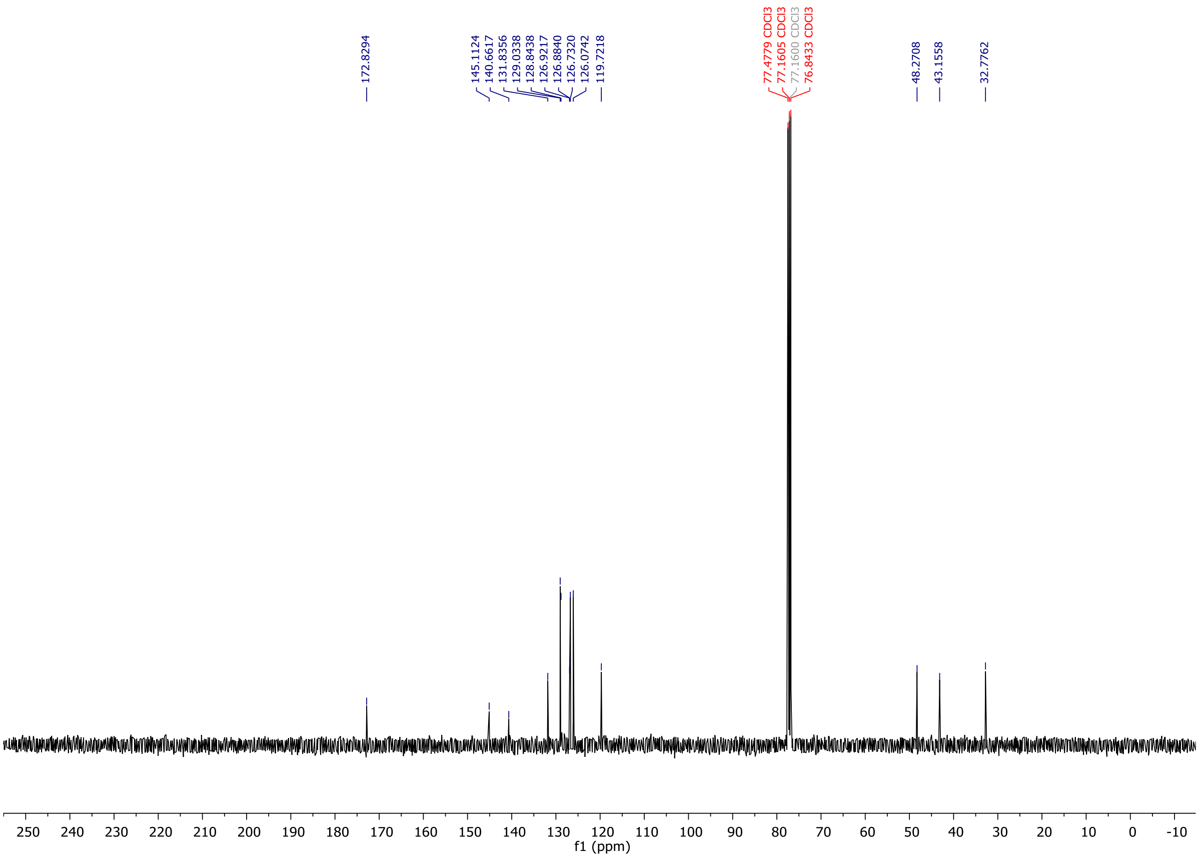

4,4-Dimethyl-1-phenyl-1,3,4,5-tetrahydro-2*H*-azepin-2-one (2t)

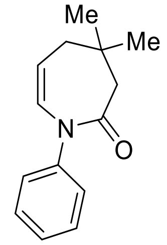

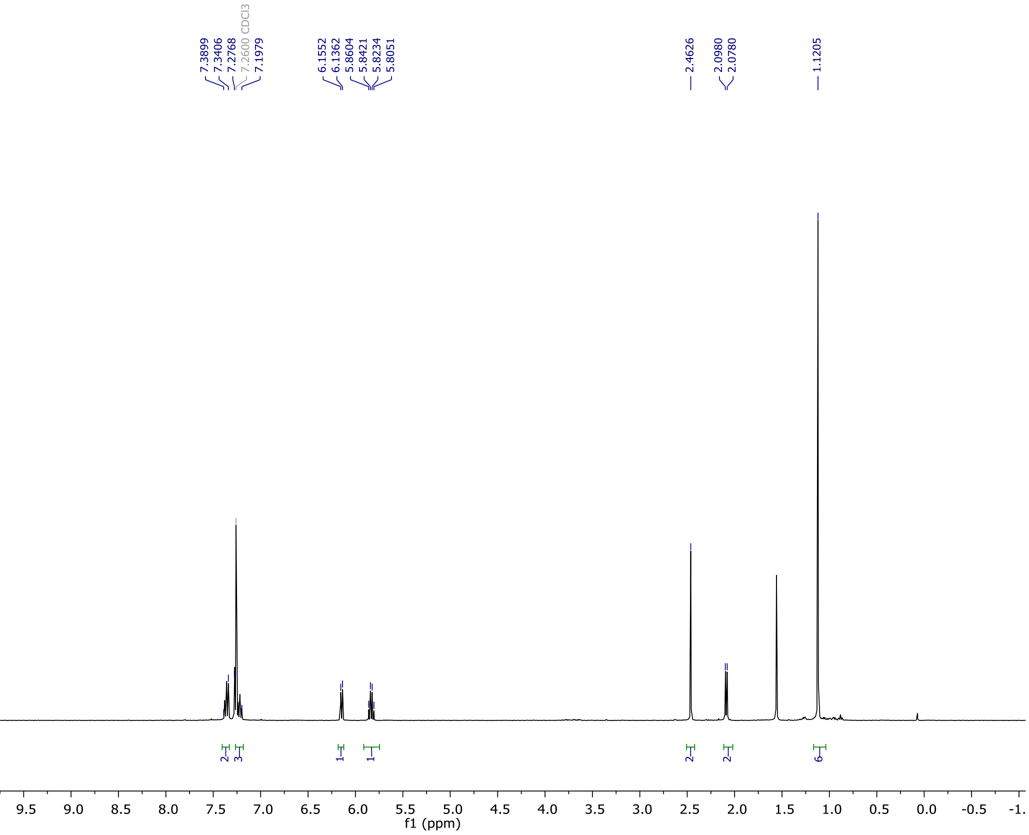

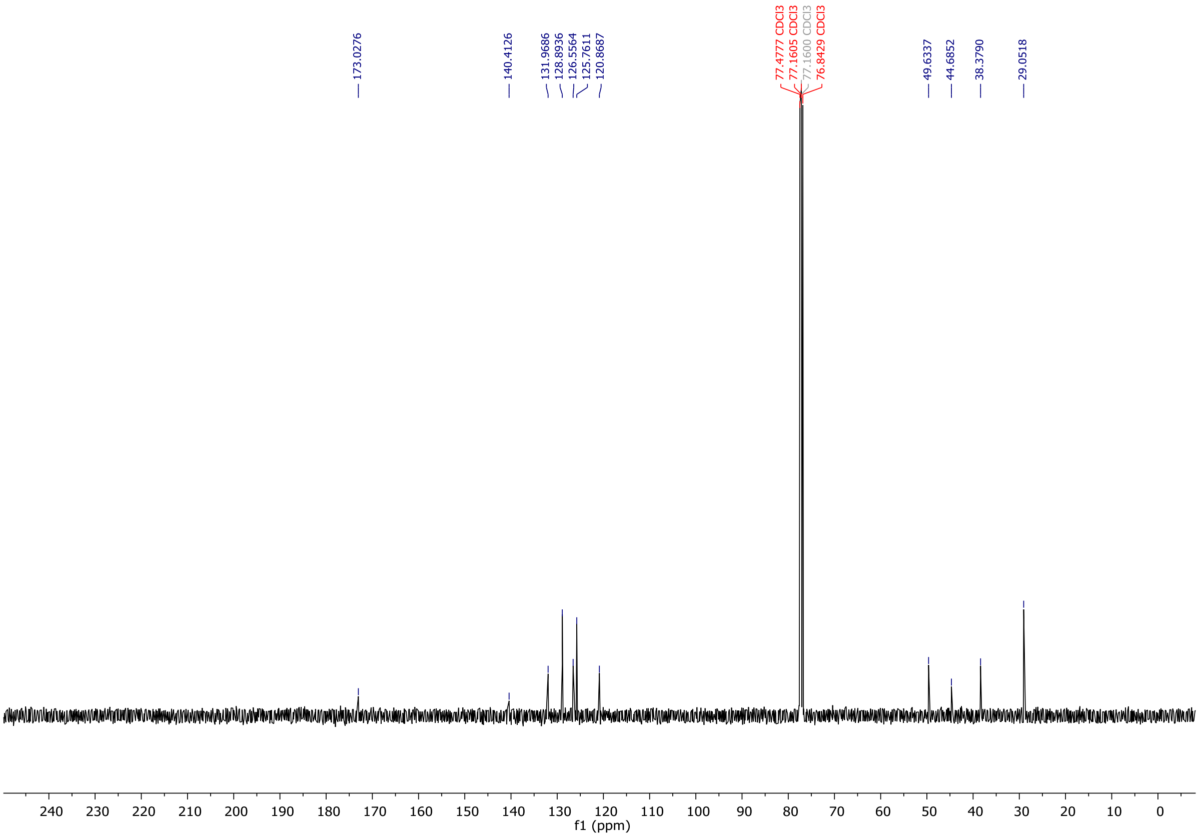

(*rac*)-3-Phenyl-3-azabicyclo[4.3.1]decan-4-one (2u')

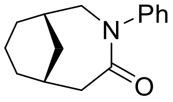

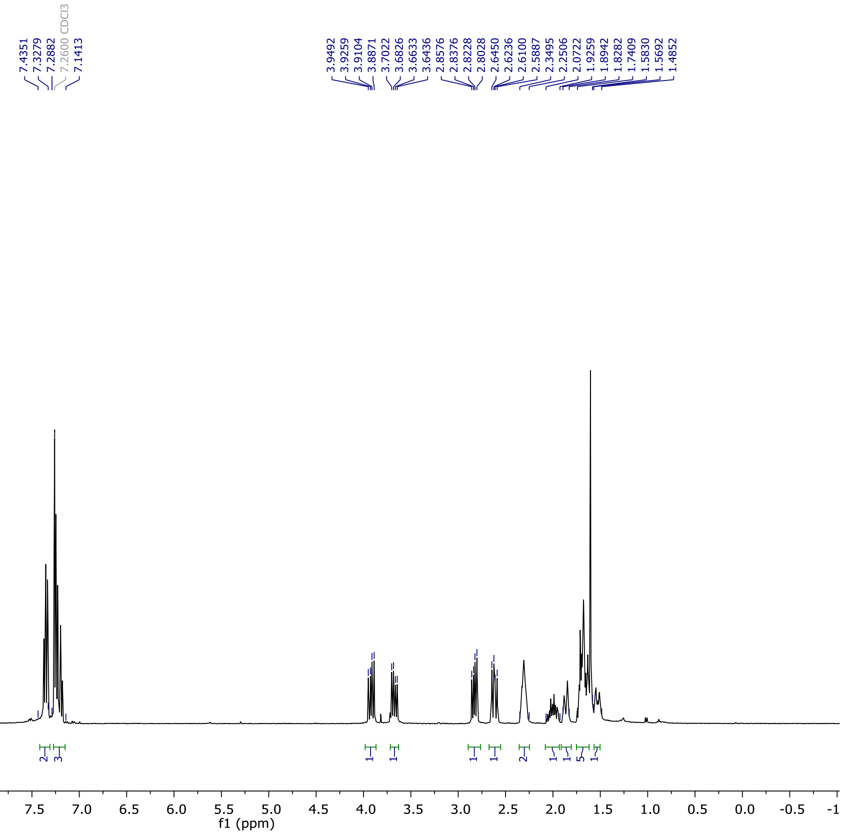

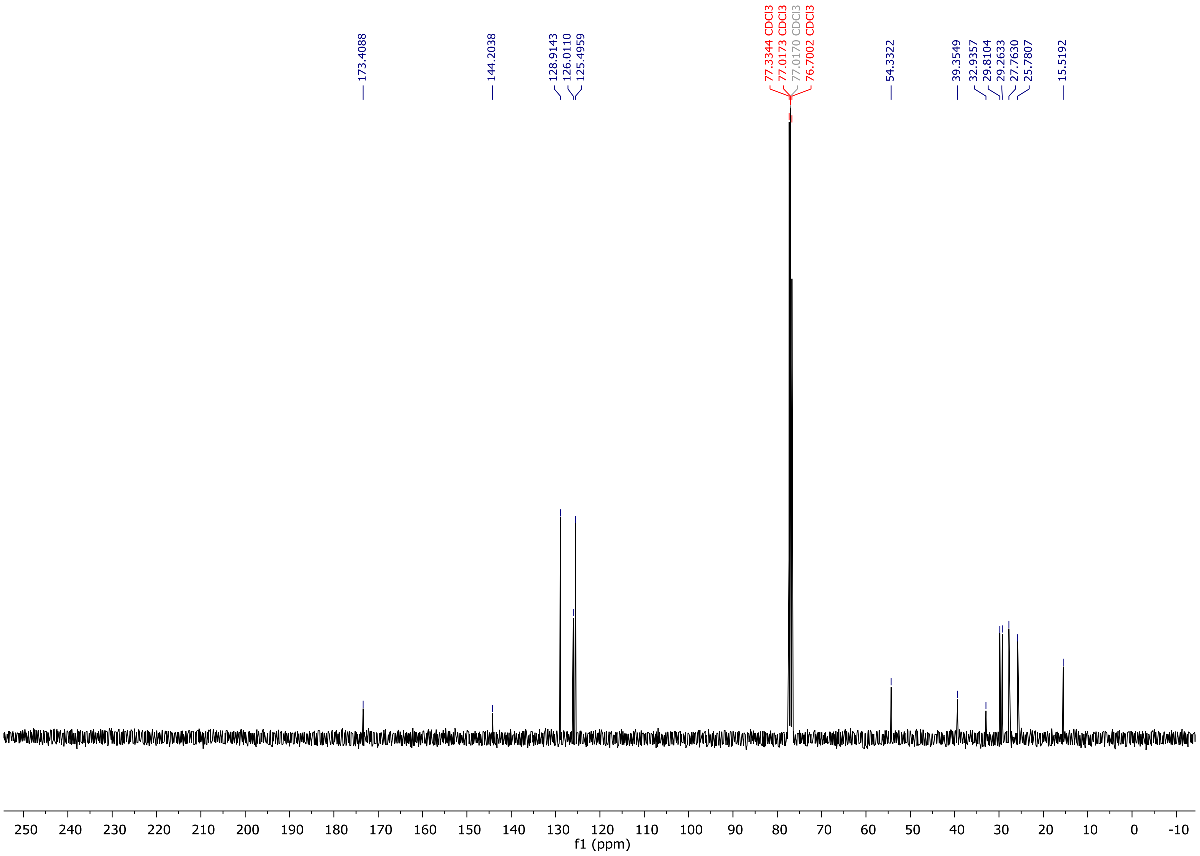

(*Z*)-2-Benzhydryl-1,4-dihydrobenzo[*c*]azocin-3(2*H*)-one (2v)

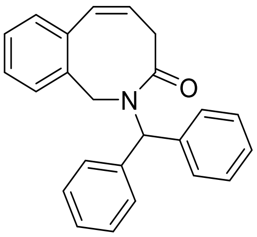

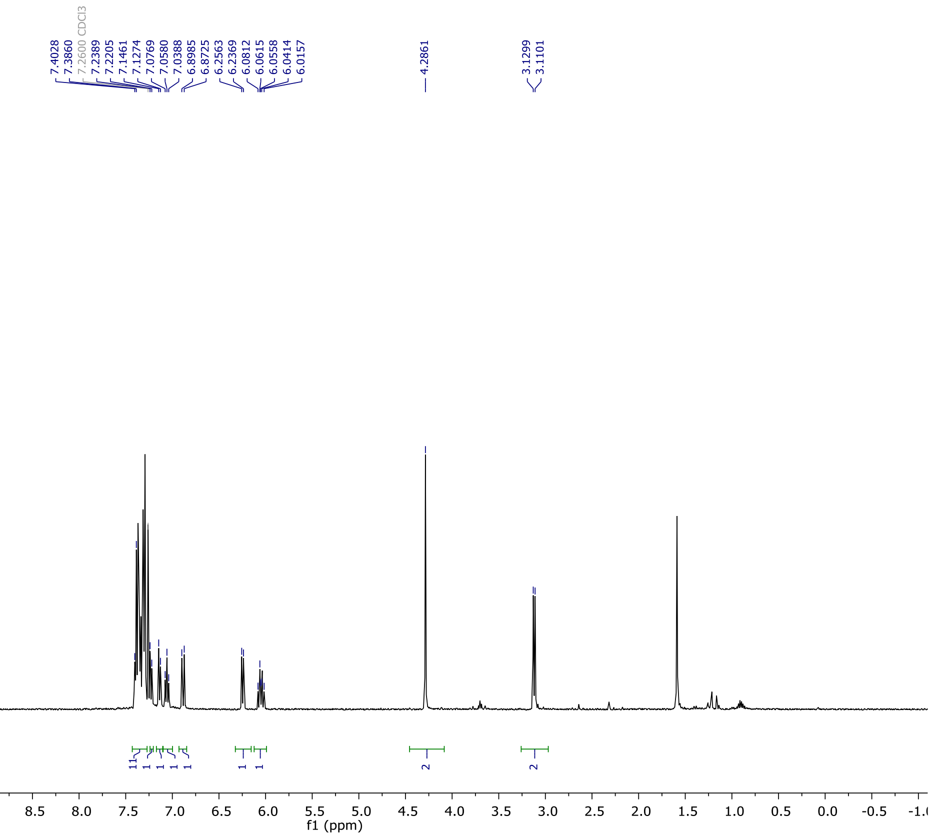

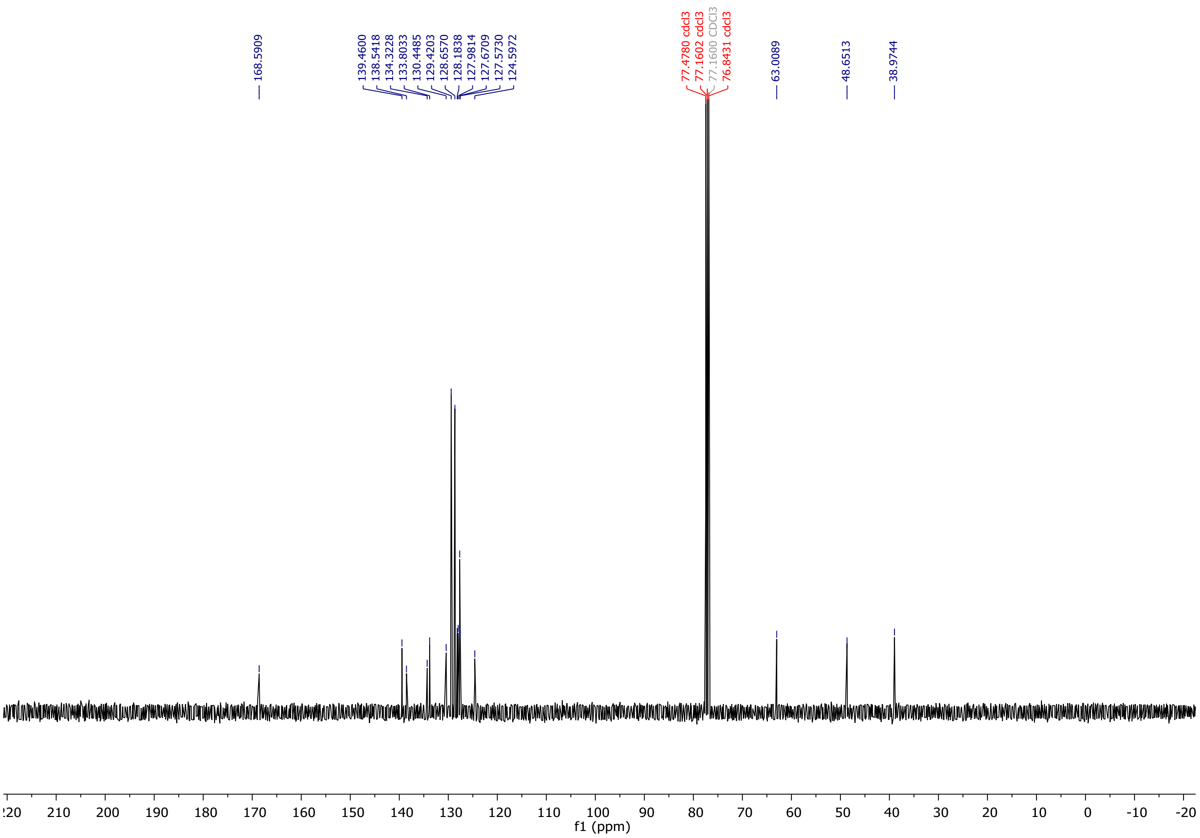

(*Z*)-2-Benzhydryl-9-fluoro-1,4-dihydrobenzo[*c*]azocin-3(2*H*)-one (2w)

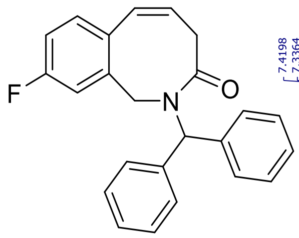

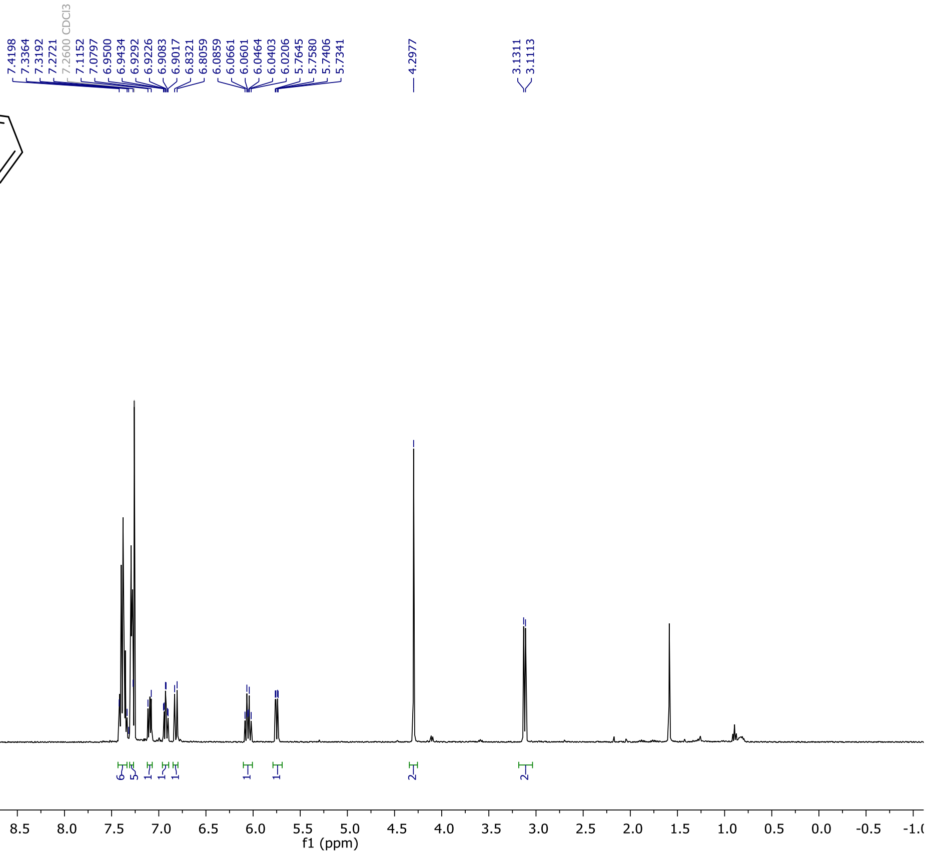

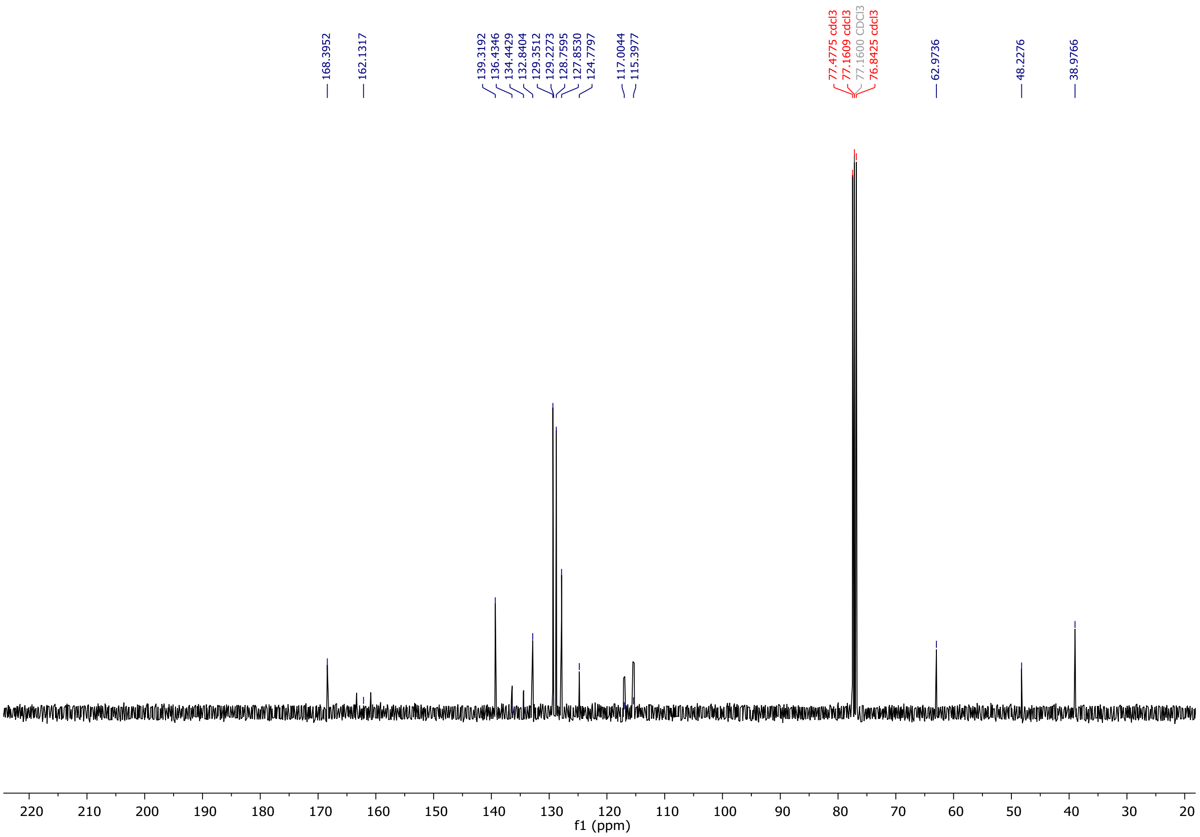

(*Z*)-2-Benzhydryl-9-methoxy-1,4-dihydrobenzo[*c*]azocin-3(2*H*)-one (2x)

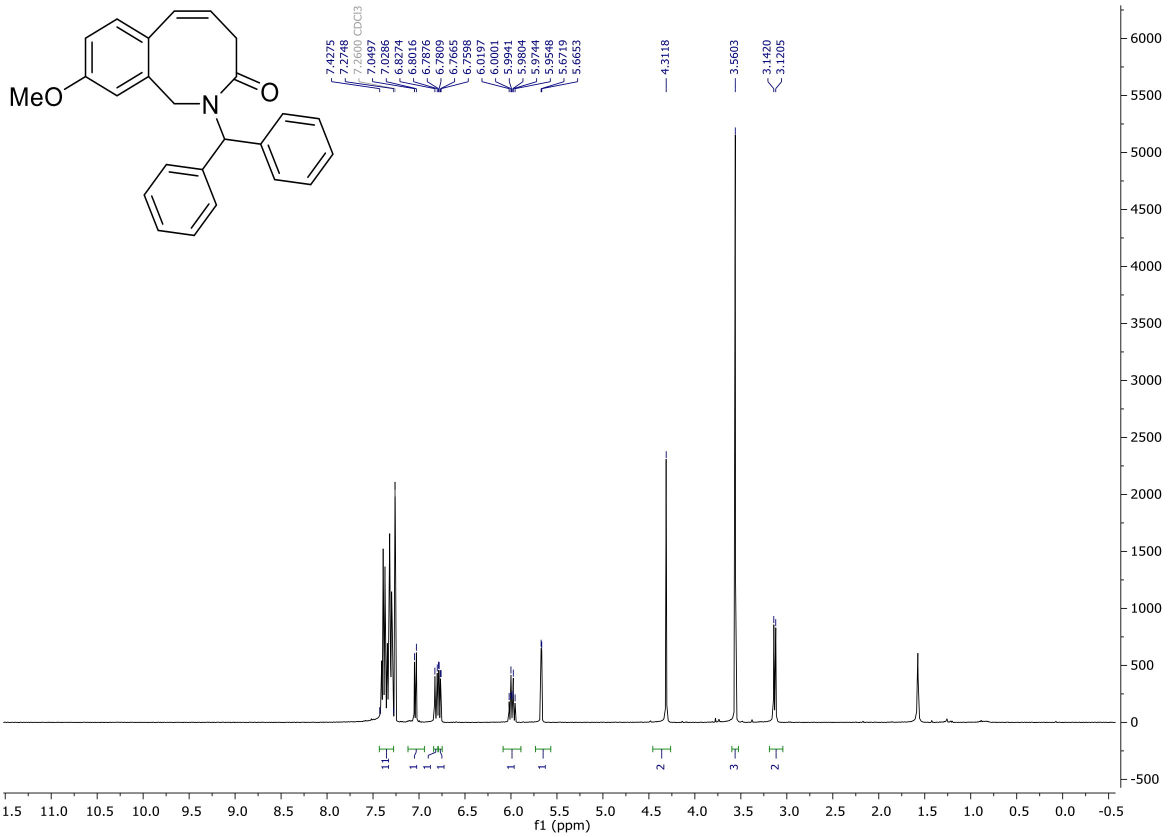

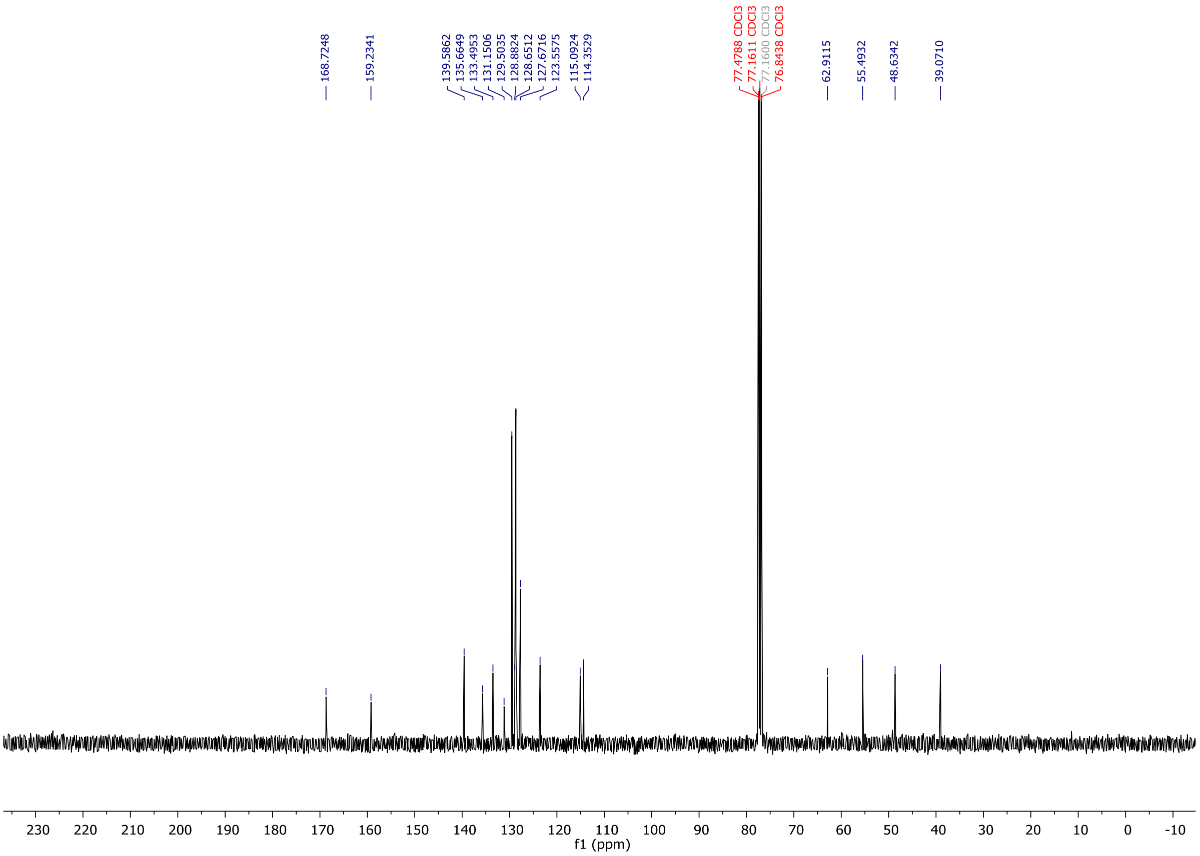

(*Z*)-3,6-Dihydrobenzo[*b*]azocin-2(1*H*)-one (2y)

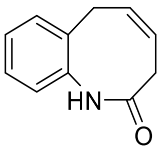

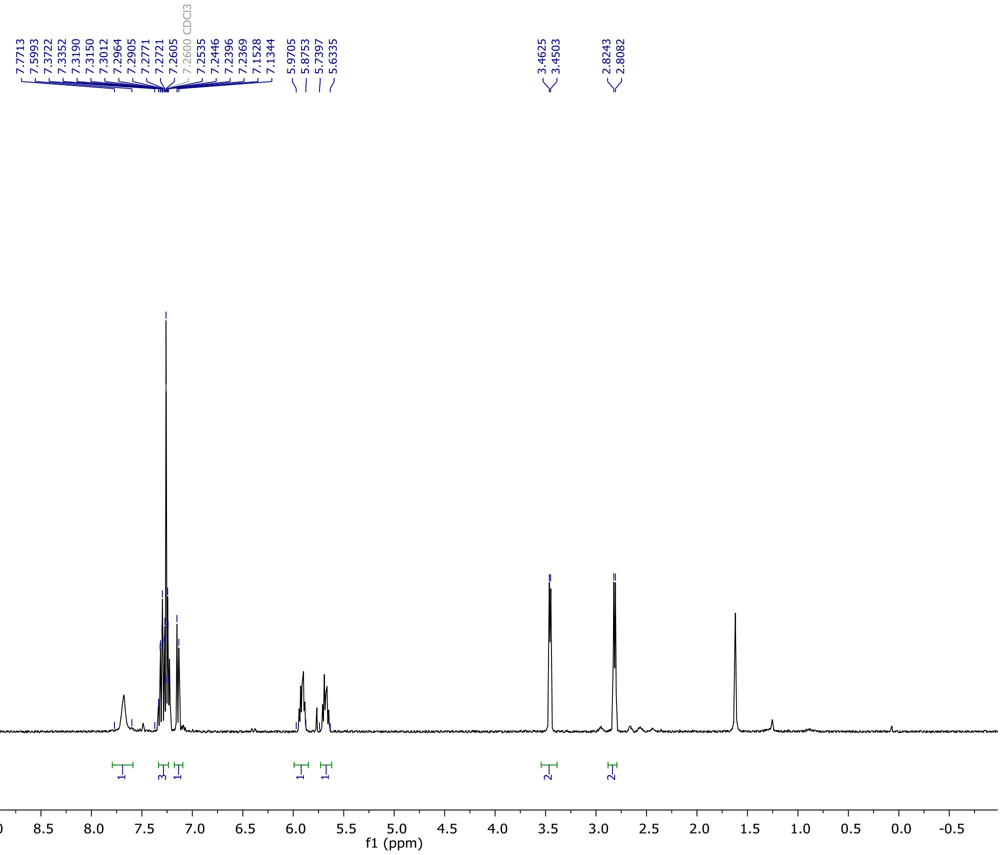

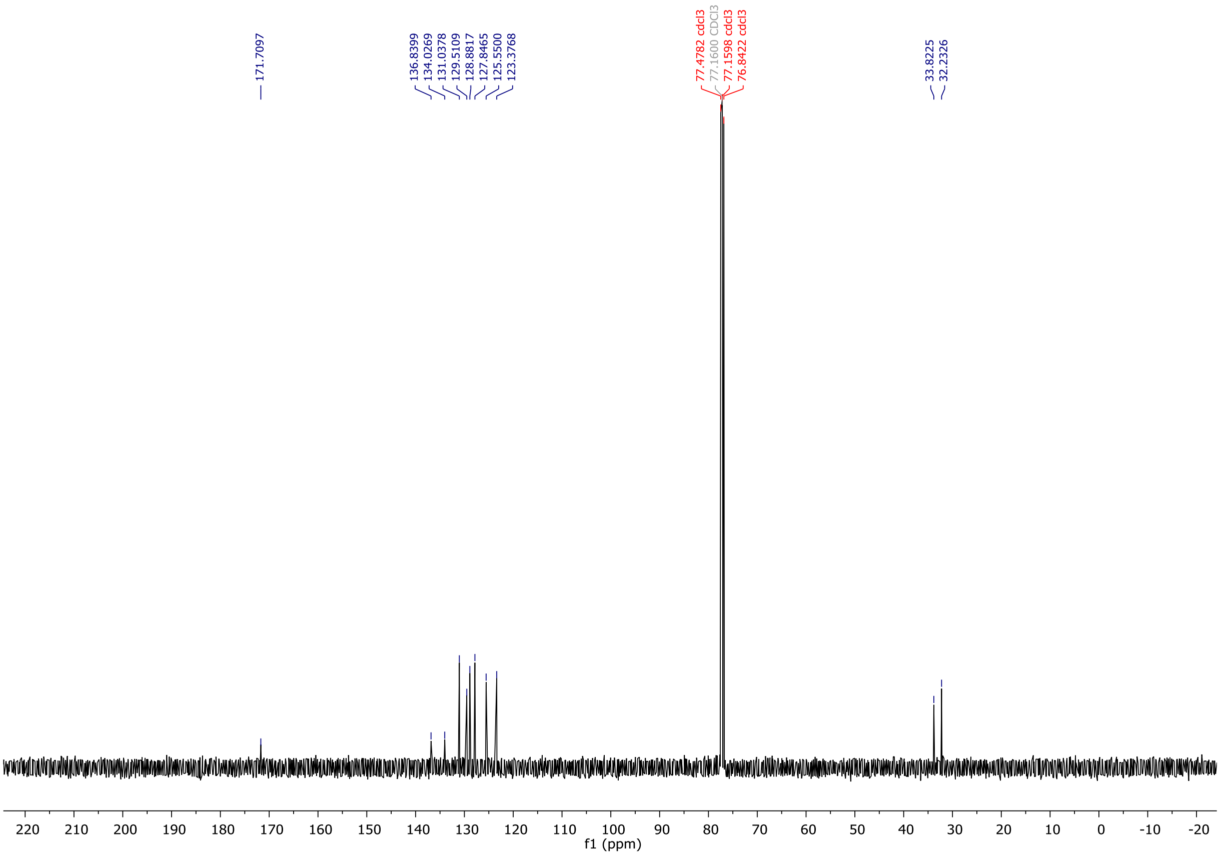

3,4,5,6-Tetrahydrobenzo[*b*]azocin-2(1*H*)-one (2y’)

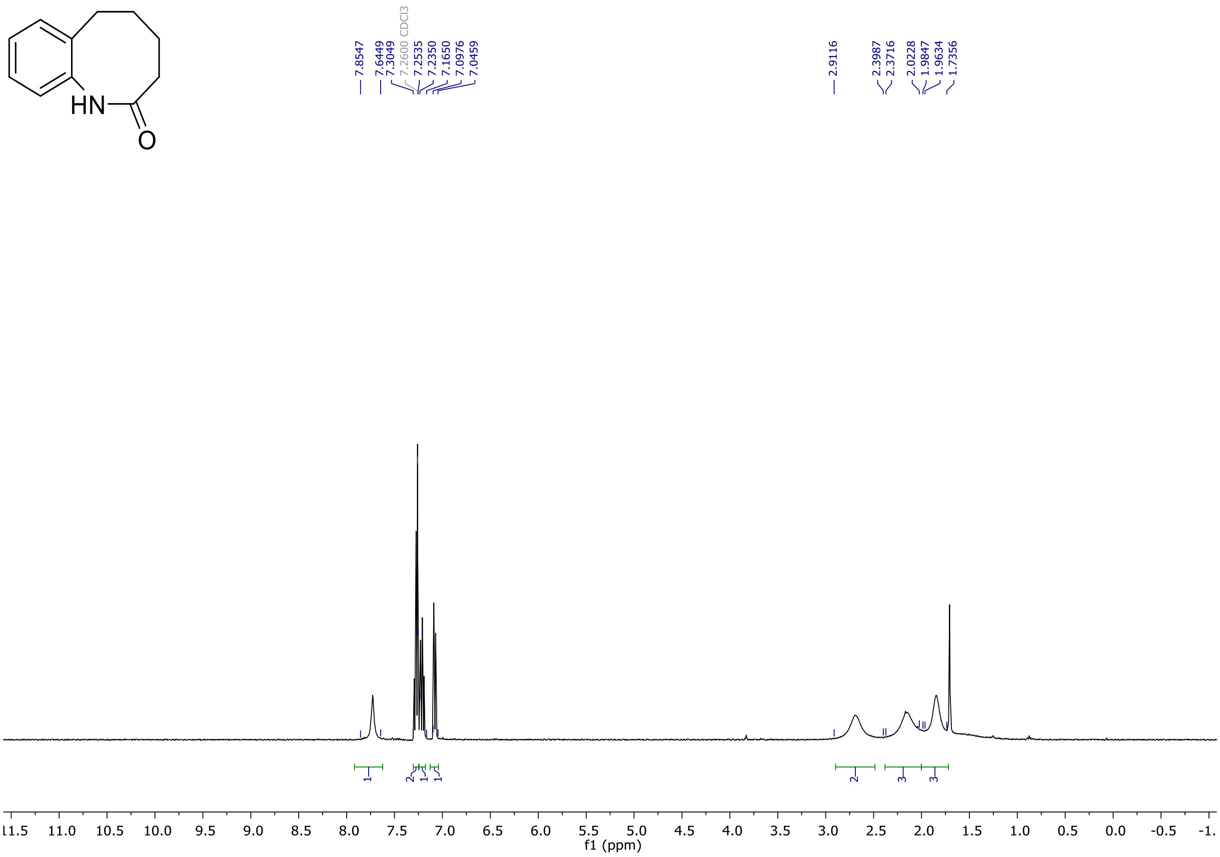

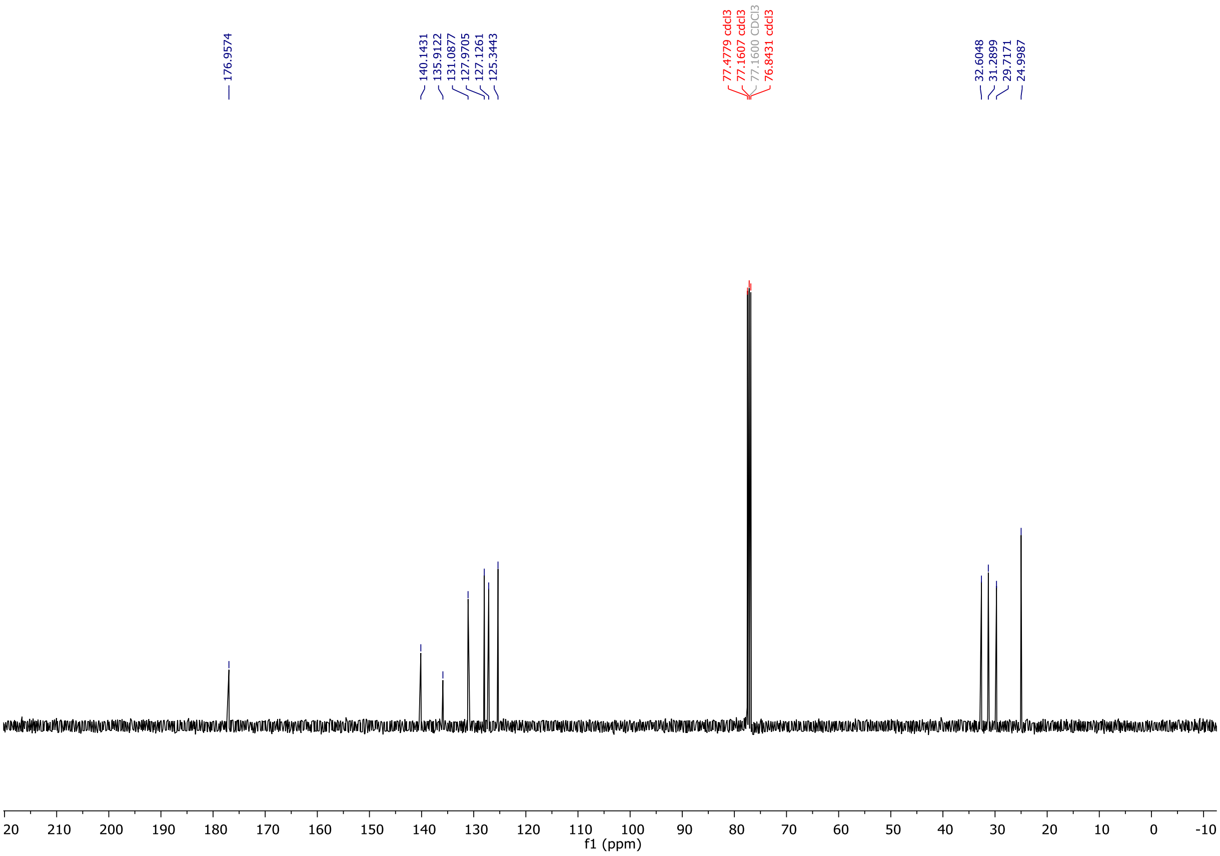

9-Methoxy-3,4,5,6-tetrahydrobenzo[*b*]azocin-2(1*H*)-one (2z')

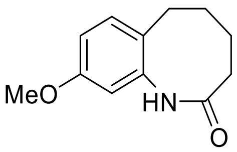

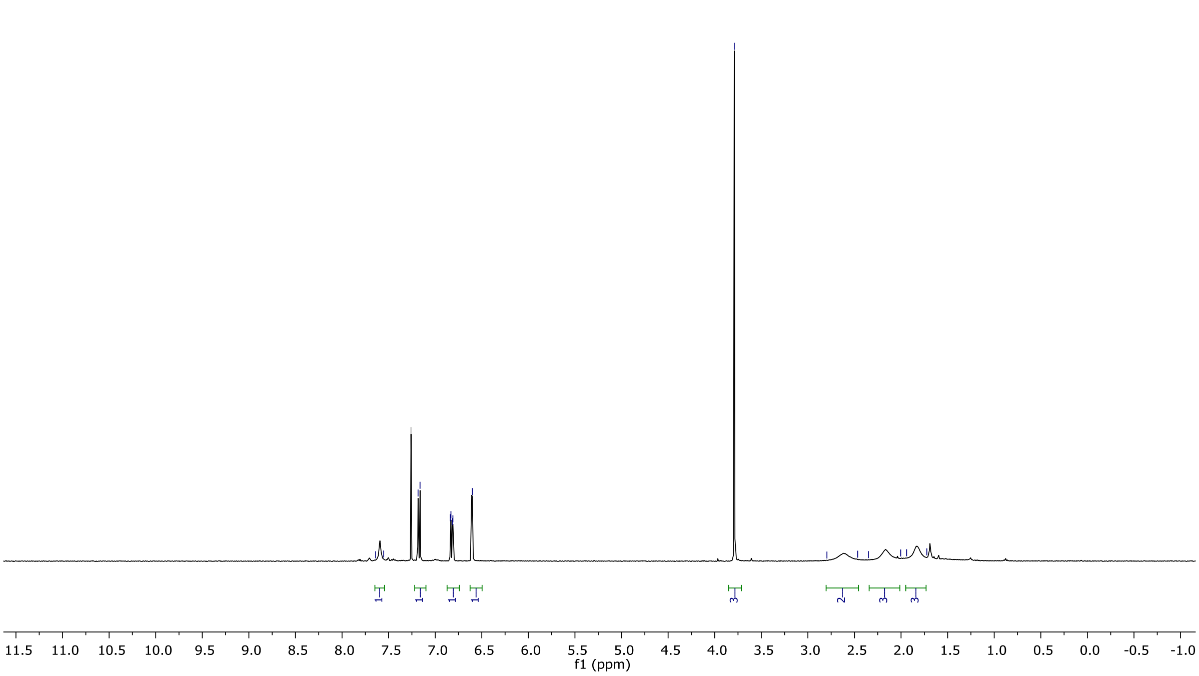

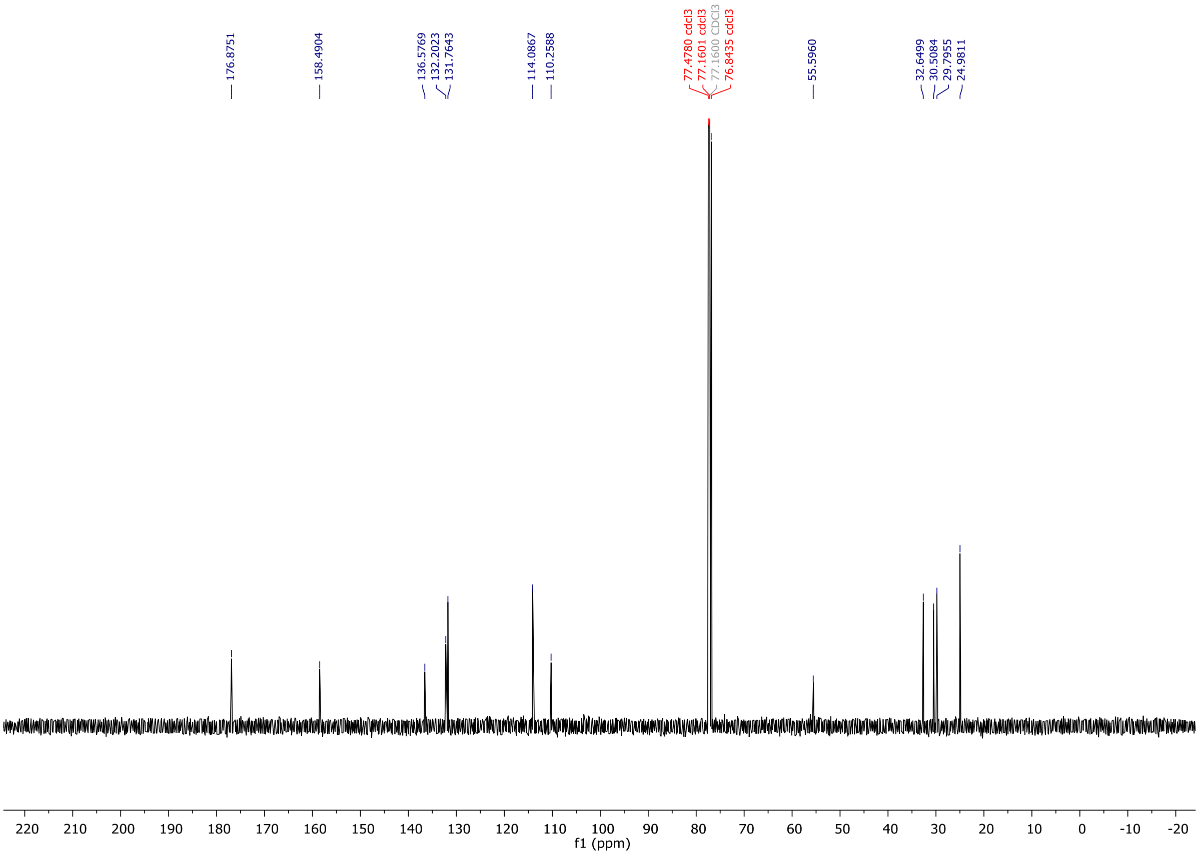

9-(Trifluoromethyl)-3,4,5,6-tetrahydrobenzo[*b*]azocin-2(1*H*)-one (2aa')

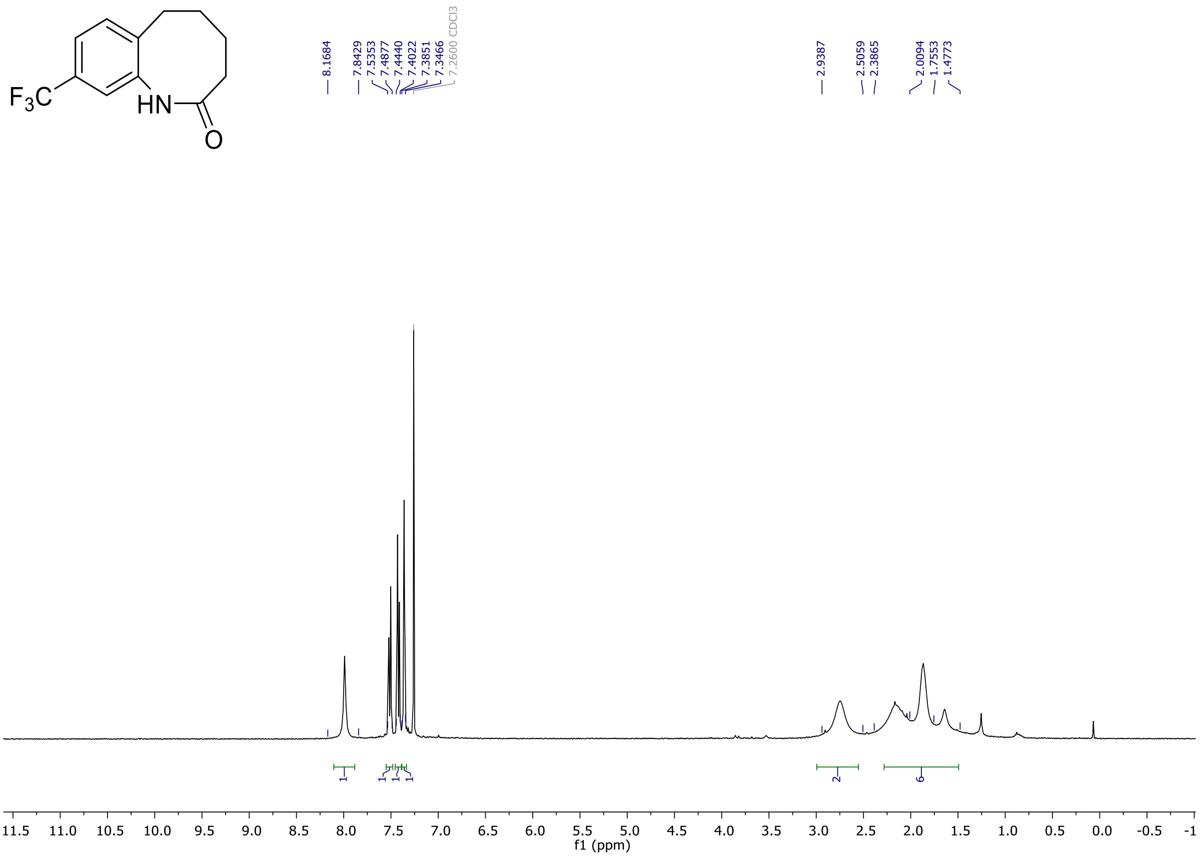

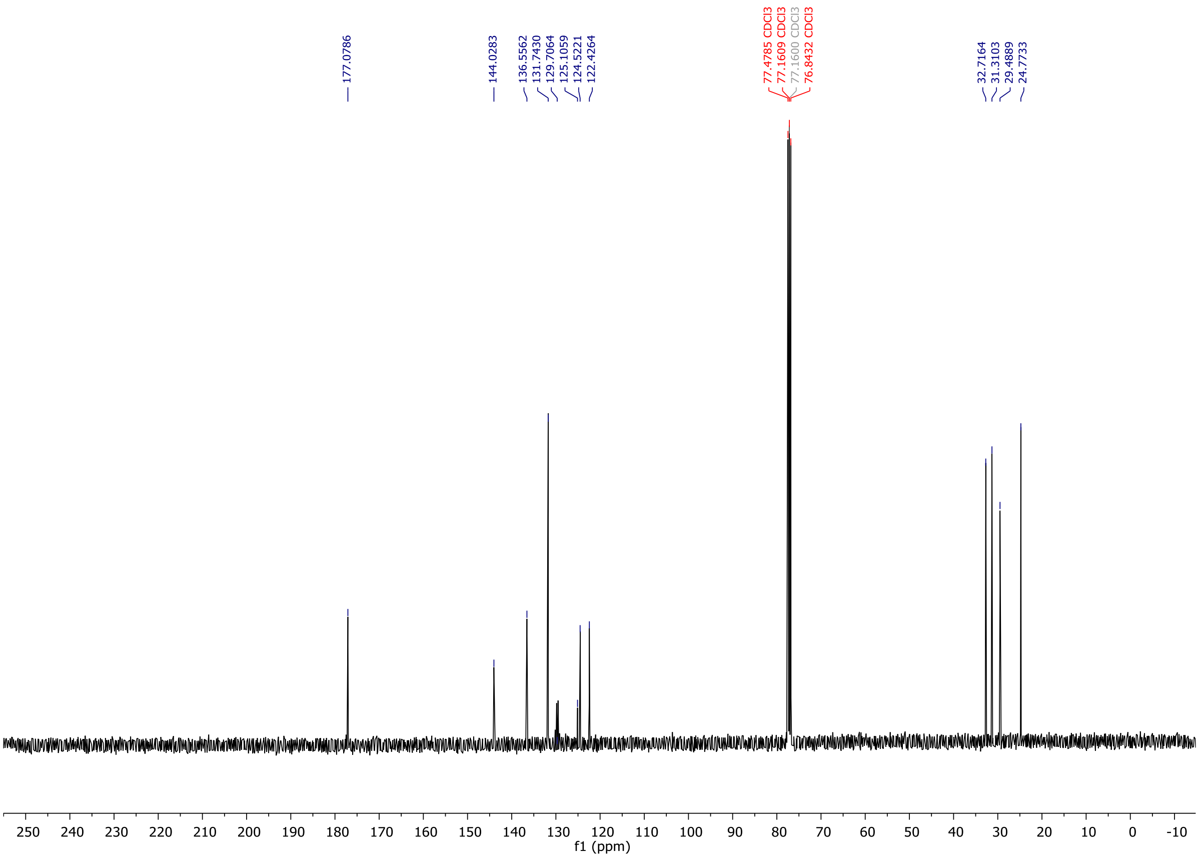

(*E*)-*N*-Phenyl-2-(prop-1-en-1-yl)aniline

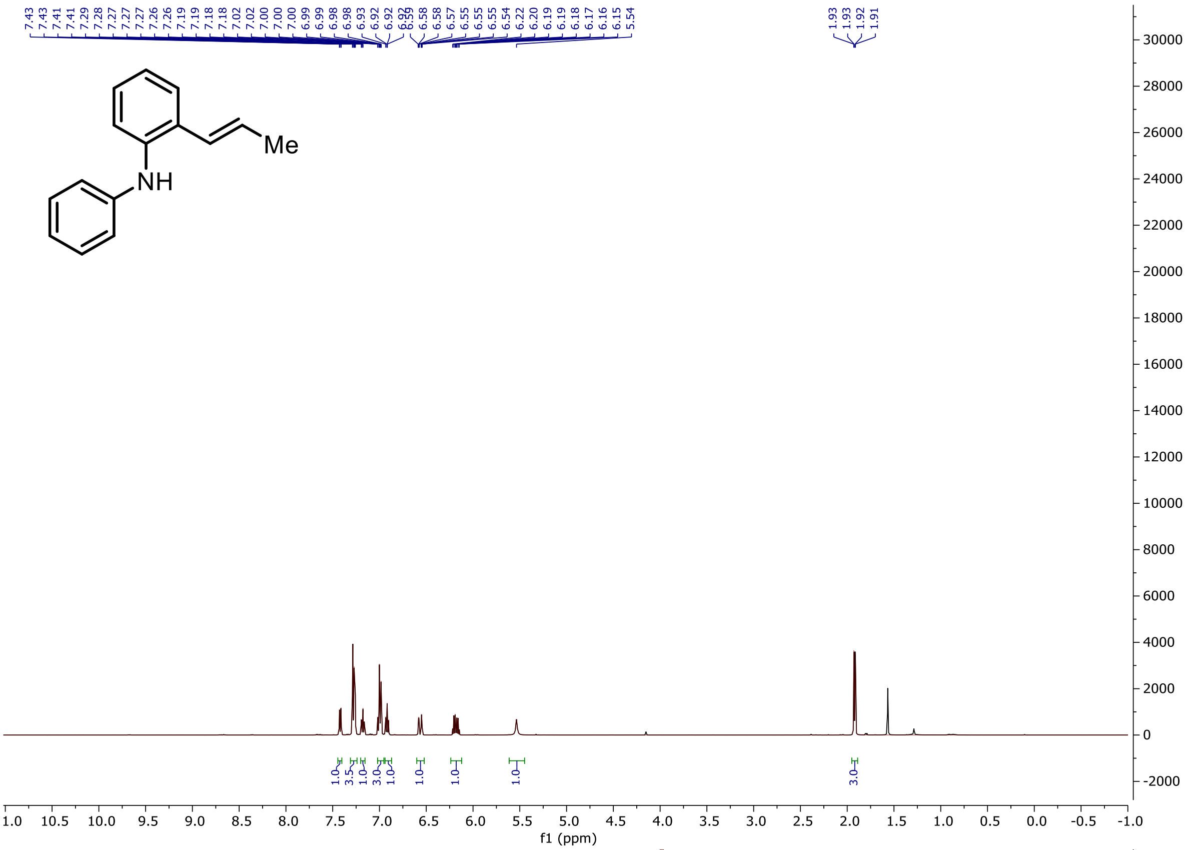

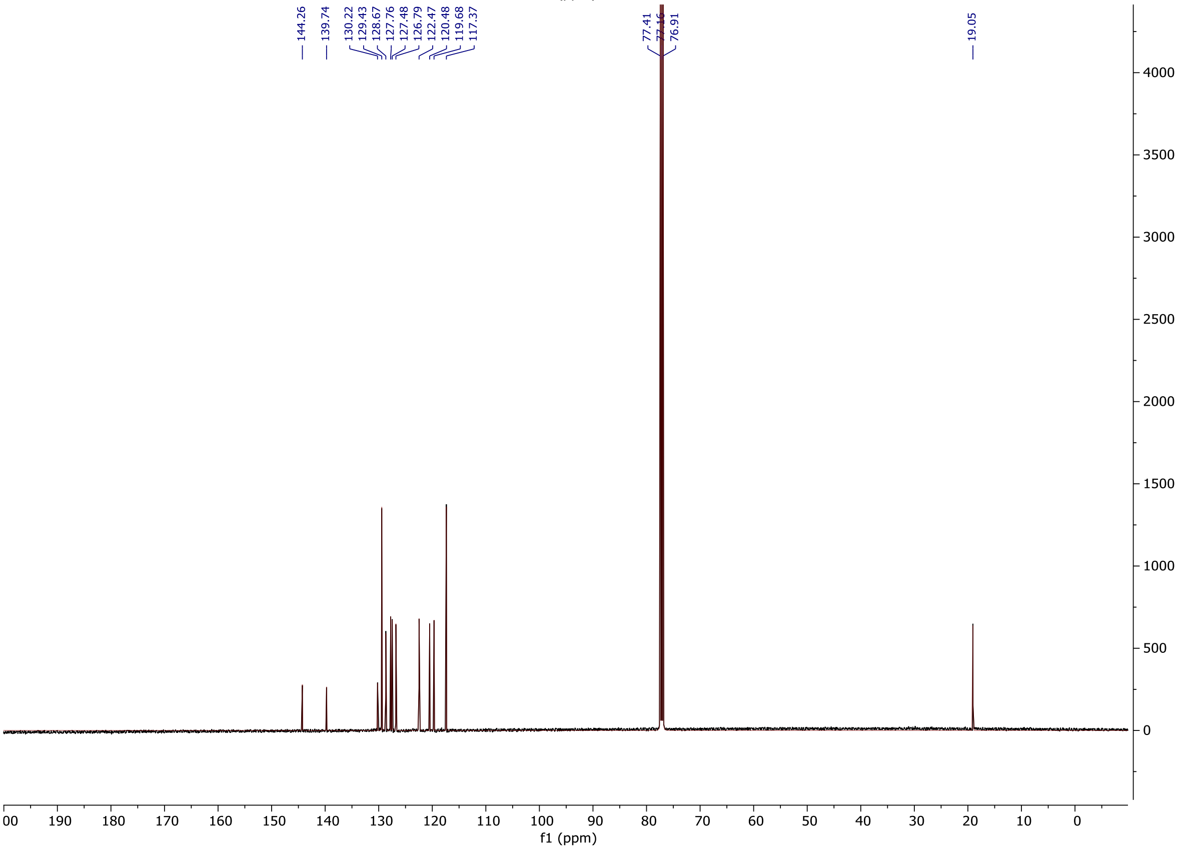

***Deuterio*-dimethyl succinate**

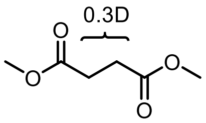

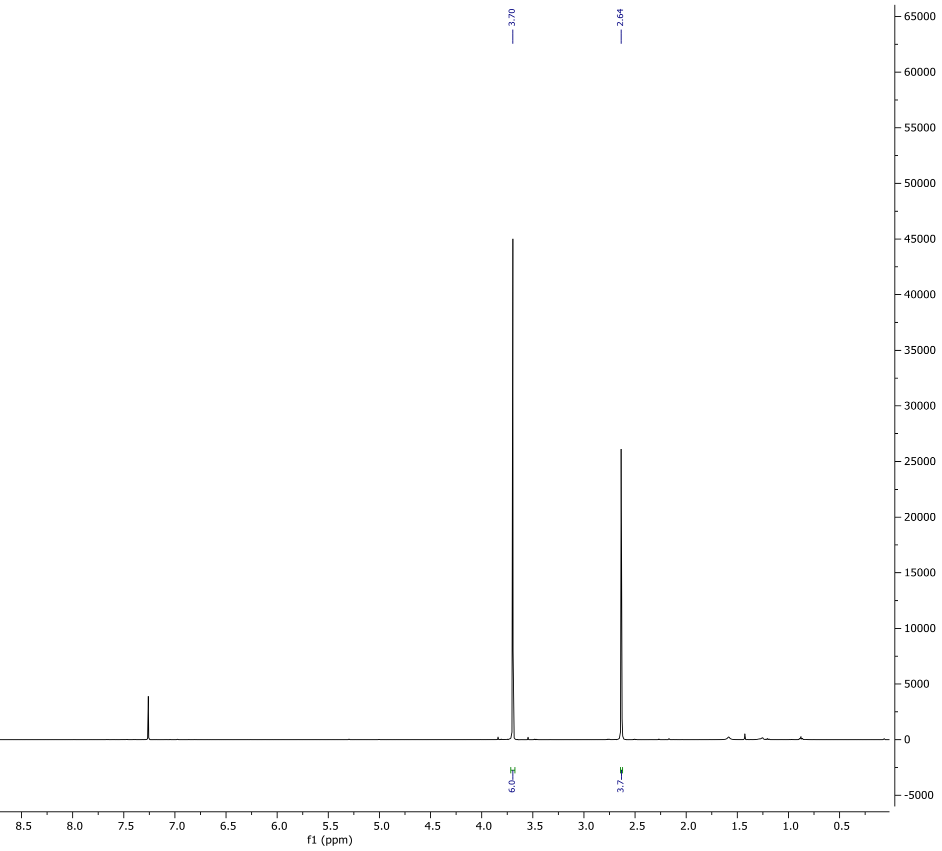

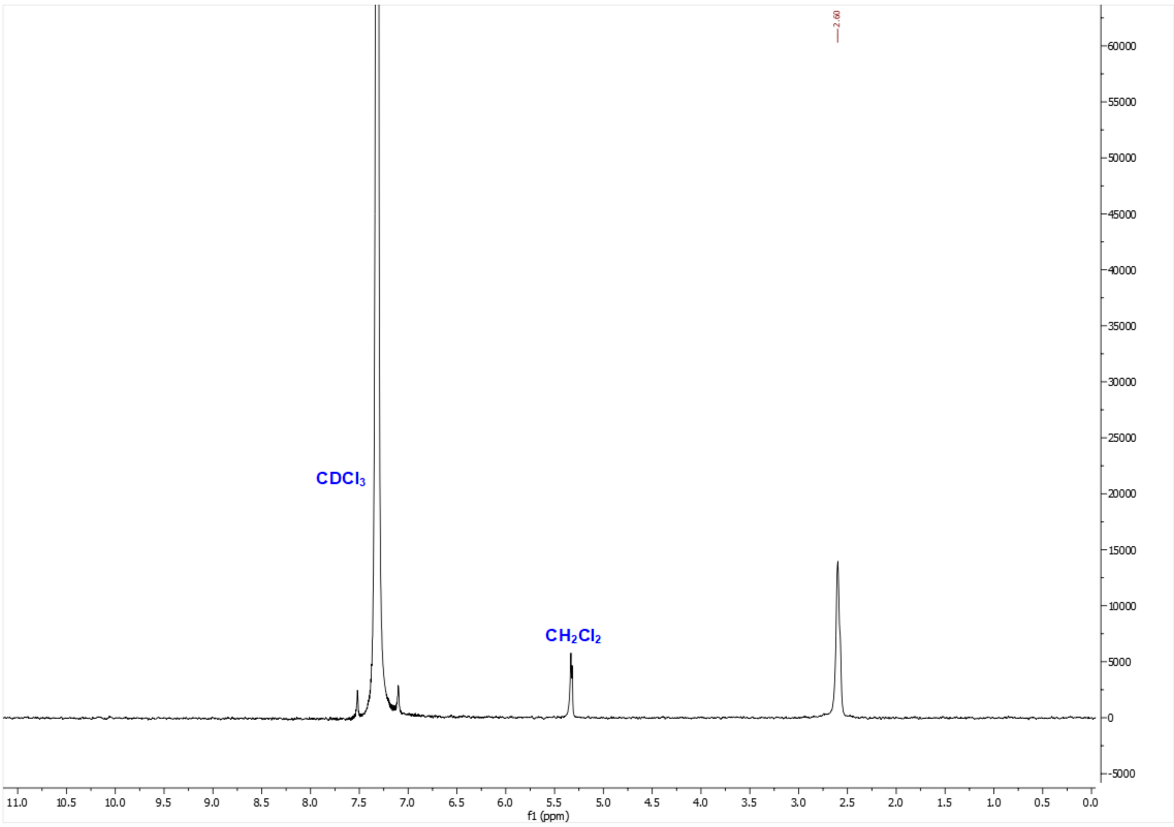

References

1 Ohwada, T.; Kasuga, M.; Shudo, K. *J. Org. Chem.* **1990**, *55*, 2717.
2 Lemhadri, M.; Doucet, H.; Santelli, M. *Synth. Commun*. **2006**, *36*, 121.
3 Scholl, B.; Hansen, H.-J. *Helv. Chim. Acta* **1986**, *69*, 1936.
4 Allen, A. D.; Baigrie, L. M.; Gong, L.; Tidwell, T. T. *Can. J. Chem.* **1991**, *69*, 138.
5 Delhaye, L.; Merschaert, A.; Delbeke, P.; Briône, W. *Org. Process Res. Dev.* **2007**, 11, 689.
6 Close, W. J. *J. Am. Chem. Soc.* **1957**, *79*, 1455.
7 Kumar, G. G. K. S. N.; Laali, K. K. *Org. Biomol. Chem.* **2012**, *10*, 7347.
8 Trofimova, E.V.; Archegov, B.P.; Fedotov, A.N.; Gazzaeva, R. A.; Mochalov, S. S.; Zefirov, N. S. *Chem. Heterocycl. Comp*. **2009**, *45*, 1095.
9 Jössang-Yanagida, A.; Gansser, C. *J. Heterocycl. Chem.* **1978**, *15*, 249.
10 Hu, T.; Li, C. *Org. Lett.* **2005**, *7*, 2035.
11 Witosińska, A.; Musielak, B.; Serda, P.; Owińska, M.; Rys, B. *J. Org. Chem*. **2012**, *77*, 9784.
12 Peng, H.; Li, T.; Yang, H.; Xu, G.; Tang, W. *Org. Biomol. Chem.* **2021**, *19*, 4327.
13 Shaw, M. H.; Melikhova, E. Y.; Kloer, D. P.; Whittingham, W. G.; Bower, J. F. *J. Am. Chem. Soc.* **2013**, *135*, 4992.
14 Miyazaki, Y.; Ohta, N.; Semba, K.; Nakao, Y. *J. Am. Chem. Soc.* **2014**, *136*, 3732.
15 Harmata, M.; Kahraman, M.; Jones, D. E.; Pavri, N.; Weatherwax, S. E. *Tetrahedron* **1998**, *54*, 9995.
16 Jolidon, S.; Hansen, H-J. *Helv. Chim. Acta* **1977**, *60*, 978.
17 Ebule, R.; Mudshinge, S.; Nantz, M. H.; Hammond, G. B.; Xu, B. *J. Org. Chem.* **2019**, *84*, 3249.
18 Durand, D; Lassau, C. *Tetrahedron Lett.* **1969**, *28*, 2329.
19 Dalling, A. G.; Bower, J. F. *Chimia* **2018**, *72*, 595.
20 McCreanor, N. G.; Stanton, S.; Bower, J. F. *J. Am. Chem. Soc.* **2016**, *138*, 11465.
